# Revealing the ecological content of long-duration audio-recordings of the environment through clustering and visualisation

**DOI:** 10.1371/journal.pone.0193345

**Published:** 2018-03-01

**Authors:** Yvonne F. Phillips, Michael Towsey, Paul Roe

**Affiliations:** Science and Engineering Faculty, Queensland University of Technology, Queensland, Australia; University of Auckland, NEW ZEALAND

## Abstract

Audio recordings of the environment are an increasingly important technique to monitor biodiversity and ecosystem function. While the acquisition of long-duration recordings is becoming easier and cheaper, the analysis and interpretation of that audio remains a significant research area. The issue addressed in this paper is the automated reduction of environmental audio data to facilitate ecological investigations. We describe a method that first reduces environmental audio to vectors of acoustic indices, which are then clustered. This can reduce the audio data by six to eight orders of magnitude yet retain useful ecological information. We describe techniques to visualise sequences of cluster occurrence (using for example, diel plots, rose plots) that assist interpretation of environmental audio. Colour coding acoustic clusters allows months and years of audio data to be visualised in a single image. These techniques are useful in identifying and indexing the contents of long-duration audio recordings. They could also play an important role in monitoring long-term changes in species abundance brought about by habitat degradation and/or restoration.

## Introduction

Interpreting long-duration acoustic recordings of the natural environment has become an important technique for ecologists wishing to monitor terrestrial ecosystems. Acoustic recordings have three advantages: 1. *Temporal and spatial cover*: given an adequate source of power, an acoustic sensor can record 24/7, whereas human field observations have obvious logistical constraints; 2. *Persistence*: acoustic data can be stored for later analysis when new techniques become available; 3. *Objectivity*: multiple persons can listen to a recording multiple times to verify content.

The availability of terabytes of acoustic data has spawned a new discipline, *ecoacoustics*, which inherits its theoretical and methodological insights from two existing disciplines, bioacoustics and landscape ecology [1]. Technology has made acoustic recordings easily available, but ecologists are now unable to listen to all the collected audio [2]. Ecoacoustics operates on large temporal and spatial scales. It treats the *soundscape* as a dynamic acoustic environment, both created by and influencing the behaviour of its embedded local fauna [3, 4]. Soundscapes consist not only of animal sounds (biophony) but also geophony (wind, rain, thunder etc.) and anthropophony (technological and human-made sounds) in great variety and combination [3, 4]. Geophony and anthropophony often provide the context for animal vocalisation, as for example bird calls during rain or when a plane is passing over.

The principal analytical tool of ecoacoustics, to date, has been the *acoustic index*, a mathematical function summarizing some aspect of the distribution (through space, time and frequency) of the acoustic energy in a recording. The early ecoacoustics literature investigated the usefulness of various acoustic indices in addressing questions of biodiversity and community [1, 5–8]. Single indices were also used to study daily and seasonal cycles [4, 9, 10]. In this paper, we take the approach that multiple acoustic indices are required to understand a soundscape [2, 11].

The approach of Towsey, Zhang et al. [12] in interpreting long-duration recordings of the environment is by sound visualisation using false-colour spectrograms. These are constructed by mapping three acoustic spectral indices calculated at one-minute resolution to the red, green and blue channels. Long duration, false-colour (LDFC) spectrograms take advantage of the eye’s ability to rapidly process large amounts of information allowing the navigation of an otherwise impenetrable 24-hour recording. A large amount of information can be gained from LDFC spectrograms once one learns how to interpret them. Bird species can be identified (despite the low temporal resolution) if the species continues to call over consecutive minutes. These calls appear as distinctive traces in LDFC spectrograms.

While LDFC spectrograms are a powerful tool for visualising soundscapes, they only achieve a 10^3^−10^5^ reduction in data. The challenge addressed in this paper is to achieve greater data reduction to allow visualisation of longer recordings while retaining ecologically useful information. Our starting point is the work of Sankupellay, Towsey [13], who clustered vectors of acoustic indices derived from 24-hour recordings to achieve a 10^7^−10^8^ data reduction. They interpreted a cluster in *index space* as an *acoustic state*, from which perspective, a soundscape can be described as a sequence of discrete acoustic states. Given a 24-hour recording, Sankupellay, Towsey [13] found that a frequency histogram of the number of times each state occurred within the day is a useful *acoustic signature* for that location and day.

As we demonstrate in this paper, treating a soundscape as a sequence of acoustic states opens new possibilities to visualise, analyse and interpret recordings of the environment. We are concerned with the endeavour to render terabytes of otherwise opaque audio data in an ecologically meaningful way. Two of the significant contributions of this paper are: 1. we describe a method to reduce long-duration recordings of the environment by clustering vectors of acoustic indices; and 2. we describe new methods to visualise long-duration recordings in order to facilitate navigation and analysis. Our task belongs to the research discipline known as *computational auditory scene analysis* (CASA) [14]. In our work, the ‘auditory scenes’ are of the natural environment and the analysis is deliberately biased to the interests of ecologists. In this work, we compare two forest sites with similar vocal species and ecology but having vegetative differences due to rainfall, landform and geology.

## Methods

**Fig 1:**
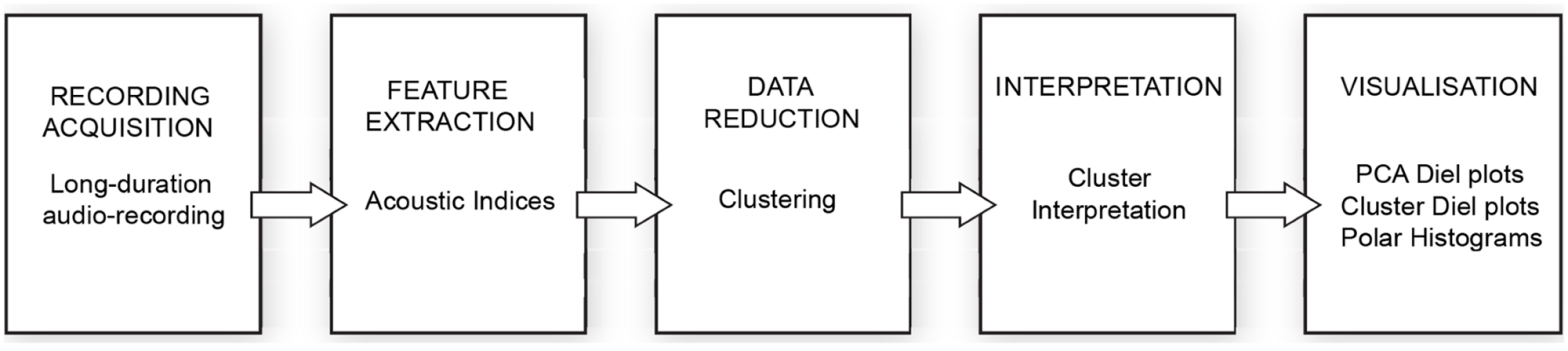
Schematic diagram of the methodology in five steps.

Our methodology can be summarised in a sequence of five steps as shown in Fig 1.

The recordings were made in two State-gazetted national parks, Gympie National Park and Woondum National Park. Both sites are roughly 160 km north of Brisbane, Queensland, Australia and are 25 km apart. The Gympie National Park (26° 3’ S, 152° 42’ E) is vegetated with Spotted Gum (*Corymbia citriodora* subspecies *variegata)* and Grey Gum *(Eucalyptus propinqua*). The open forest site was recently burnt (possibly in 2014) and has very little vegetative groundcover. The Woondum National Park (26° 16’ S, 152° 47’ E) site is a forest of Gympie Messmate (*E*. *cloeziana*), Pink Bloodwood (*C*. *intermedia*) and Grey Gum (*E*. *propinqua*). It is bordered by Flooded Gum (*E*. *grandis)* to the west and by sub-tropical gallery rainforest along a dry creek to the north. For further site details and photos see [15] and S1 File: Site information. From this point, each site will be referred to as the Gympie or Woondum site.

The Gympie site was part of an earlier study [16] and therefore is well characterised. The Woondum site is at a lower elevation and closer to the east coast of Queensland. The regional proximity of the two sites ensures some correlation between their weather patterns, tree species and vocal bird species. However, the Woondum site has higher average rainfall, 1600 mm at nearby Tewantin compared to 1040 mm at Gympie city nearer to Gympie National Park [17, 18]). The Woondum site consequently has a denser scrub understory and a more closed canopy. Frogs were very rarely heard because both sites are on well-drained slopes. The sites are distant from roads and human habitation. Sounds from planes and the occasional motor-bike are present but the sounds from other vehicles are rare.

Recordings were obtained using Song Meter (SM2+) acoustic recorders [19]. A schedule of continuous recording commenced at midnight on the 22^nd^ June 2015 and ended on 23^rd^ July 2016, 13 months in total at each site. Files were saved in WAV format, sampled at 22050 Hz, onto secure digital (SD) cards in 16-bit stereo. Batteries were changed weekly along with the SD cards. Each recording unit was attached to a tree at a height of 1.5 m. The omnidirectional microphones were attached directly to the meters. The final 26-month near-continuous recording consists of 5.7 terabytes of audio files.

Battery changes caused short breaks in the recording. For visualisation purposes, these breaks were inserted as N/A values in order to retain the integrity of the time scale. However, for clustering purposes, these minutes were removed from the data.

### Feature extraction

All recordings were divided into consecutive, non-overlapping, one-minute segments of audio. One-minute of recording is typically used to calculate indices, such as the acoustic complexity index, and it is an appropriate duration to capture the state of a soundscape. Due to intermittent microphone problems, we calculated indices using only one channel. Twelve indices were calculated for each one-minute segment.

The twelve summary acoustic indices used in the clustering are either derived from the wave envelope or the spectrogram. These were calculated using the Audio Analysis Programs software [20]. We use three-letter codes for ease of identifying the indices. For a full description of how each of these indices is calculated, refer to [21]. For the dataset that resulted from the calculation of the summary acoustic indices see S2 File: Gympie Acoustic Indices and S3 File: Woondum Acoustic Indices.

The indices derived from the wave envelope were calculated using the maximum absolute amplitude value in each of 2584 non-overlapping frames (one frame = 512 samples, frame duration = 23.2ms) over the period of one minute. The amplitude envelope was converted to a decibel envelope using dB = 20×log_10_*A*. The first four indices are derived from the waveform envelope converted to decibels.

1 Background Noise (BGN): Calculated as described in Towsey [21]
2 Signal to Noise (SNR) The difference between the maximum decibel value in the decibel envelope and the value of BGN.
3 Events per Second (EVN): A count of the number of acoustic events per second.
4 Activity (ACT): The fraction of noise-reduced decibel envelope which exceeds 3 dB.

The remaining indices were derived from the spectrogram. Each one-minute segment was converted to an amplitude spectrogram by calculating a FFT (with Hamming window) over non-overlapping frames (width = 512). Each spectrum of 256 values (bin width ≈ 43.1 Hz) is smoothed using a moving average filter (width = 3). Fourier coefficients are converted to decibels using dB = 20×log_10_(*A*). Indices 5, 6, and 7 are derived from the noise-reduced decibel spectrogram as described in [21].

5 Low-frequency Cover (LFC): The fraction of spectrogram cells that exceed 3-dB in the low-frequency band (1–1000 Hz) of the noise-reduced decibel spectrogram.
6 Mid-frequency Cover (MFC): As for LFC but in the mid-frequency band (1000–8000 Hz). The mid-band is chosen to capture most of the bird vocalisations but avoid the anthropophony which predominates at low frequencies.
7 High-frequency Cover (HFC): As for LFC but in the high-frequency band (8000–10982 Hz).

Indices 8, 9, and 10 describe three different measures of the distribution of acoustic energy in the mid-band (1000–8000 Hz) of each noise-reduced, one-minute amplitude spectrogram. The noise-reduced amplitude values in the spectrogram are squared to give energy values. To obtain a more intuitive index, we subtract each entropy value from 1.0, to obtain a measure of ‘concentration’.

8 Entropy of the Peaks Spectrum (EPS): measures the degree of ‘concentration’ of the distribution of spectral maxima over one-minute of recording.
9 Entropy of the Average Spectrum (EAS): measures the degree of ‘concentration’ of energy distribution in the mean-energy spectrum derived from a one-minute recording.
10 Entropy of the Spectrum of Coefficients of Variation (ECV): measures the degree of ‘concentration’ of the distribution of values in the normalised energy-variance spectrum.

Indices 11 and 12 are ‘ecological’ acoustic indices, only in the sense that they have been used by ecologists as surrogate measures of species richness.

11 Acoustic Complexity Index (ACI) is obtained from the amplitude spectrogram using the method in Pieretti, Farina [22]. It measures the amplitude fluctuations across frequency bins and is designed to be indicative of the extent of biophony in a one-minute audio segment.
12 Cluster Count: the number of distinct spectral clusters in the mid-frequency band of a one-minute segment of recording. Calculated as described in Towsey [21]. This index attempts to measure the amount of internal acoustic structure within the mid-band where bird calls predominate [23].

Note that four of the above acoustic indices are derived from the waveform and six are derived from the mid-band (1000–8000 Hz) where bird calls are expected to predominate. This reflects the important contribution of bird calls to biophony at the two recording sites. We did trial other acoustic indices but found that they were highly correlated with at least one of the above indices (*R*^2^>0.7). No pair of the above twelve summary indices was correlated above the 0.7 threshold. The values for each index were normalised between the 1.5 and 98.5 percentiles.

Feature extraction reduced 5.7 terabytes of recording files to a final dataset consisting of 1,141,147 feature vectors, each having twelve normalised acoustic indices. There were 773 unrecorded minutes; 241 due to battery changes and 532 due to file corruption. An additional 4320 minutes (three full days, 28–30 October 2015) were removed before the final clustering due to the failure of both left and right microphones in the Gympie National Park recording unit.

### Clustering 26 months of acoustic data

The dataset was clustered using a hybrid method. For a full description refer to Phillips and Towsey [15]. The method involves the use of *k*-means followed by hierarchical clustering. The approach was designed to take advantage of the strengths of each clustering algorithm. K-means is fast and can be used on large datasets, but it delivers different results depending on initialization. Hierarchical clustering does not scale well but is deterministic once a distance metric is selected. Our hybrid clustering method consists of three steps:

1. Partition the total dataset using k-means clustering (*kmeans* in R stats package [24]) into a large number (k1) of clusters. We explored values of *k*1 from 15,000 to 27,500 in steps of 2500. K-means partitions a set of feature vectors (in our case, vectors of 12 acoustic indices) such that each vector is assigned to the cluster having the nearest cluster centroid (where the distance measure is Euclidian). This version of k-means is initialised with a random selection of *k* data instances and terminates when there is no change in cluster content [25].
2. Cluster the series of the *k*1 cluster centroids from step 1 using hierarchical clustering (*hclust* in the R *stats* package [24]). Cut each hierarchical tree to produce *k*2 clusters. We trialled a range of *k*2 values from 10 to 100 in steps of 5. We used agglomerative clustering (or ‘bottom-up’ approach) in which each feature vector starts in its own cluster. Pairs of most similar clusters are merged sequentially eventually resulting in one cluster containing all the data.
3. Assign each vector in the dataset to the nearest of the *k*2 cluster centroids from step 2 using k-nearest-neighbour (*knn* in the R *class* package [26]). K-nearest-neighbour assigns each vector in the dataset to a *k*2 cluster, again by using Euclidean distance. For more detail on these steps see [27].

The optimum value for *k* (or in our case the optimum values for *k*1 and *k*2) is typically determined by use of a clustering index such as the Dunn index [28] or Silhouette index [29]. These indices measure the discreteness and tightness of the resulting clusters but they are agnostic to the clustering application. In our case, these indices yielded ambiguous results. We therefore developed an ‘error’ function to optimise *k*1 and *k*2 that attempted to measure how well a cluster set partitioned the biophony in our recordings [15]. Our intention was to achieve a clustering result that maximised information of ecological interest.

### Optimising *k*1 and *k*2

The hybrid-clustering algorithm requires the optimisation of two parameters (*k*1 and *k*2) to minimise our measure of clustering error. We consider our choice of error criterion to be an important contribution of this paper. Our choice rests on two assumptions:

1. That the biophony in two 24-hour recordings (rain and wind free) will be more similar, the closer the two recording sites are in space and the closer the two recording days are in time. Conversely, differences in vocal species and their calling behaviour (and therefore differences in biophony) will increase with increasing seasonal and landscape separation.
2. That the *acoustic signatures* (calculated according to the method of Sankupellay, Towsey [13]) of two 24-hour recordings having similar biophony will be closer than the acoustic signatures of two recordings having dissimilar biophony. Given a clustering run that yields *N* clusters, an *acoustic signature* is an N-bar histogram, each bar of which is the count of times that a member of that cluster occurs within a 24-hour period.

These two assumptions are supported by the results of Sankupellay, Towsey [13] who derived an acoustic signature to summarise the acoustic content of a 24-hour day. Note: Sankupellay, Towsey [13] use the term “acoustic fingerprint” rather than “acoustic signature”. They found that 24-hour acoustic signatures derived from recordings made at the same site are more similar than acoustic signatures of recordings from different sites.

To make use of this result, we selected six days of recording from each of our two sites. The days were carefully chosen to be wind and rain free, that is, to maximize the content of biophony and minimise the content of geophony. Each group of six days was divided into two sets of three days separated by 30 days (Table 1). The ability of a clustering result to produce acoustic signatures that segregated these twelve days into four groups of three days became our measure of clustering ‘effectiveness’ or ‘utility’ and was used to optimize the values of *k*1 and *k*2.

**Table 1:** Summary of twelve-day dataset, 6 days from each of the two sites.

	Gympie NP site	Woondum NP site
Mid-winter	30 July 2015 (day 1) 31 July 2015 (day 2) 1 Aug 2015 (day 3)	30 July 2015 (day 7) 31 July 2015 (day 8) 1 Aug 2015 (day 9)
Early-spring	31 Aug 2105 (day 4) 1 Sept 2015 (day 5) 4 Sept 2015 (day 6)	31 Aug 2015 (day 10) 1 Sept 2015 (day 11) 4 Sept 2015 (day 12)

For a given clustering result that produces *N* clusters, each of the twelve days in Table 1 was converted to an *acoustic signature* (normalized N-bar histogram). These twelve acoustic signatures were then clustered hierarchically (*hclust* in the R stats package, distance metric = ward.D2) to produce a 12-leaf dendrogram.

According to the above reasoning, a ‘good’ clustering result will produce clusters (and therefore *acoustic signatures*) that divide the twelve days into four groups of three (as in Table 1). We formulated an ‘error’ index, the *intra-three-day-distance* (I3DD), which quantifies the extent to which each 12-leaf dendrogram (see Results section) groups the days as expected. The calculation of the I3DD value is based on the maximum heights linking pairs of the three-day groups within the 12-leaf dendrograms. For details refer to Phillips and Towsey [15]. Smaller I3DD values indicate greater intra-group integrity, that is, a sharper demarcation of the twelve days into four groups of three.

### Cluster significance

An understanding of the acoustic content of the clusters and their ecological significance was determined using the following four methods:

i Listening to a random sample of 20 one-minute recordings from each cluster.
ii Plotting the temporal distribution of clusters: 24-hour histograms of cluster occurrence are likely to reveal cluster content. For example, clusters dominant around dawn suggests their content is morning bird chorus. Clusters dominant at evening suggests insect chorus.
iii Mapping the cluster medoids onto two dimensions using a Sammon projection (*sammon* function in R MASS package [26]; *pam* function in R cluster package [30]). This facilitated visualisation of the relationships between the different acoustic clusters. A Sammon projection maps data from a high-dimensional space (twelve in our case) to a lower dimensional space (two in our case) while preserving the relative inter-point distances. A medoid is the cluster member closest (in Euclidian sense) to the centroid of the cluster. The advantage is that, in an irregular cluster, one is certain to select as a cluster representative, an instance that exists.
iv Comparing cluster medoids using radar plots: The values of the twelve normalised acoustic indices in each cluster medoid indicate which indices are important in defining the cluster.

## Results

### Clustering results

**Fig 2:**
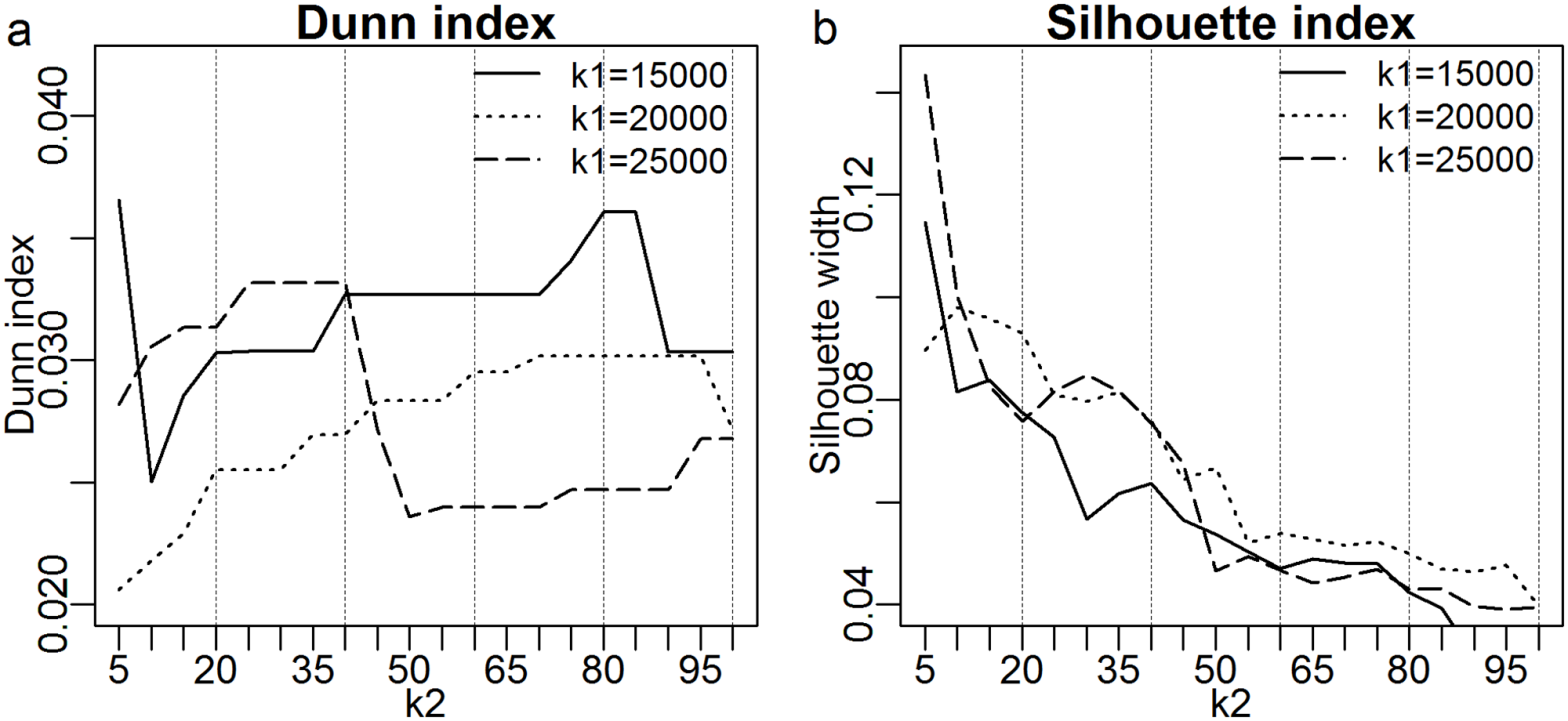
The (a) Dunn and (b) Silhouette index calculated on the result of step 2 in the hybrid method (see [15]).

The Dunn index indicated that *k*1 = 15000 and *k*2 = 5 or 80 was the best combination of parameter values (Fig 2a). There is a great difference between 5 and 80 clusters, and experience suggests that 5 clusters is too few and 80 is unnecessarily many for discretising environmental sound. By contrast, the Silhouette index implied that the data could not be well clustered at all (Fig 2b). The maximum Silhouette value of 0.14 at five clusters was well below the 0.25 threshold, usually interpreted to imply that there is “no substantial structure” in the data [31 p.343]. Because these indices did not provide clear optimum values for *k*1 and *k*2, we turned to the formulation of the I3DD ‘error’ function derived from acoustic signatures of days having maximum biophony.

**Fig 3:**
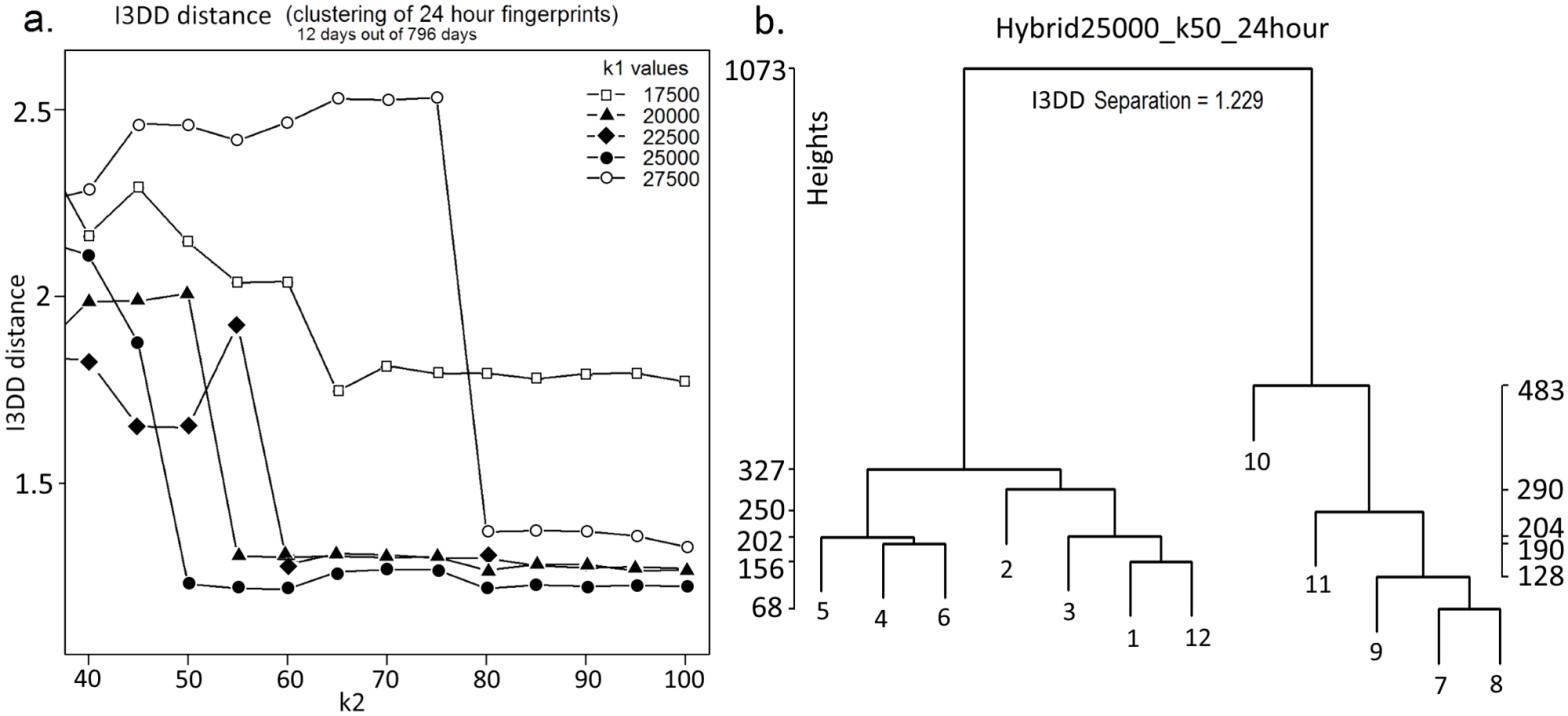
(a) I3DD ‘error’ versus k1 and k2 for the hybrid clustering method on the 26-month dataset. (b) Dendrogram for the hybrid run (k1 = 25000, k2 = 50). The I3DD error measures how well the four groups of three days are separated in the dendrogram.

The parameter values based on the I3DD index [15] are given in Fig 3a. Each of the five curves in the figure reveals a minimum acceptable value for *k*2 given the value of *k*1. We selected the value of *k*2 = 60, being the minimum value of the curve, *k*1 = 25,000. A dendrogram for the twelve acoustic signatures for one of the clustering runs is shown in Fig 3b. Note that the dendrogram has two main branches corresponding to the sites of Gympie (days 1 to 6) and Woondum (days 7 to 12). Only day 12 (4^th^ September) is ‘misplaced’ in the tree.

### Cluster interpretation

Having obtained sixty acoustic clusters, the next step was to determine the acoustic content of each cluster using the four methods outlined in the Methods sub-section “Cluster significance”.

#### 1. Listening to a sample from each cluster

Twenty one-minute recordings were randomly sampled from each of the sixty clusters, yielding a total of twenty hours of recording. Each minute of audio was listened to via the Ecosounds Acoustic Workbench [32]. This website records annotations for future reference. Systematic listening to the samples revealed that there were seven major sources of acoustic events: (1) Quiet, (2) Wind, (3) Birds, (4) Orthoptera (crickets and grasshoppers), (5) Homoptera (cicadas), (6) Rain and (7) Planes.

Forty-two of the sixty clusters or 73.4% of the 1,141,147 one-minute segments were dominated by a single sound source (or in the case of quiet clusters, the absence of a clearly identifiable source). Assigning an acoustic class label to these clusters was relatively straight forward (Table 2). Note that a *dominant* sound source does *not* mean the *only* sound source. Insects sounds, for example, were present in many recordings but were not necessarily the dominant sound source, especially when birds and wind were also present. A further eleven clusters or 19.3% of the one-minute segments contained two or more dominant sound sources. The remaining seven clusters or 7.3% of the one-minute segments were difficult to label. These clusters contained inconsistent and infrequent combinations of acoustic events having low amplitude but were not quiet enough to be labelled ‘Quiet’.

**Table 2:** The 60 acoustic clusters grouped into classes according to their dominant acoustic sources. This table summarises the information obtained by listening to 1200 minutes (20 hours) of recording (for further information see S4 File: Sample minutes and Phillips [33].

Class Label	Number of clusters assigned class label	% of total minutes assigned class label		Class description: The dominant kinds of acoustic event
		Gympie NP	Woondum NP	
ONE DOMINANT SOUND SOURCE				
Quiet	9	20.6%	26.2%	Absence or near absence of sounds belonging to all other classes below.
Birds	8	20.1%	14.4%	Bird calls or songs throughout.
Wind	10	15.8%	15.3%	Sounds associated with air movement.
Orthoptera	3	9.1%	6.3%	Insects sounds excluding those from cicadas.
Cicadas	6	5.1%	5.5%	Cicada sounds dominant over other classes.
Rain	4	1.7%	5.6%	Rain sounds.
Planes	2	0.9%	0.4%	Low frequency sounds due to airplanes, motorbikes or other vehicles.
TOTALS	(42)	(73.3%)	(73.7%)	Cumulative Totals
TWO OR THREE DOMINANT SOUND SOURCES				
Orthoptera/Birds	2	7.8%	10.2%	Insects and birds equally vocal.
Cicadas/Birds/ Wind	2	5.1%	2.6%	Cicadas, birds and wind equally evident.
Rain/Birds	3	1.6%	3.9%	Rain and birds equally evident in two of these clusters and more rain in the third.
Birds/Quiet	1	2.0%	1.3%	Low rate of bird calls in most minutes with a quiet background.
Birds/Planes	1	2.1%	0.3%	Birds & planes.
Orthoptera/Wind	1	0.3%	0.9%	Mostly distant insects in quiet background or wind.
Wind/Planes/ Orthoptera	1	0.5%	0.1%	Mostly moderate wind, some planes & insects.
TOTALS	(11)	(19.4%)	(19.3%)	Cumulative Totals
INCONSISTENT SOUND SOURCES				
Wind/ Birds	1	1.5%	1.9%	Inconsistent: Either birds & wind or Orthoptera & wind.
Light rain/ Orthoptera	1	0.9%	2.3%	Inconsistent: Light rain or Orthoptera.
Birds/Planes/ Orthoptera	1	2.0%	0.5%	Inconsistent: Birds, Orthoptera or Planes.
Quiet/Planes	1	1.5%	0.7%	Inconsistent: Mostly quiet with distant planes.
Wind/ Cicadas	1	1.1%	0.7%	Inconsistent: Wind &/or cicadas.
Quiet/ Birds Orthoptera	1	0.3%	0.6%	Inconsistent: Orthoptera &/or birds.
Birds/Wind	1	0.1%	0.3%	Inconsistent: Birds or wind.
TOTALS	(7)	(7.4%)	(7.0%)	Cumulative Totals

The nine clusters assigned to the *Quiet* class had an absence of, or very little, sound from other classes. Recordings assigned to the *Plane* class (two clusters) were found also to include thunder, but planes remained the most frequent ‘loud’ event. *Wind* clusters were the most difficult to characterise because there was a gradation from high wind into silence. All samples from the three *Bird* morning chorus clusters (37, 43 and 58) had birds as the dominant sound source. Random samples from the eight Bird clusters (a total of 160 samples) indicated that birds were the dominant sound source in 92% of those recordings (see S4 File: Sample minutes). The remaining 8% of *Bird* recordings contained significant contributions from insects or other source in addition to bird calls. To see the cluster list refer to [34].

#### 2. Temporal distributions of the clusters

**Fig 4:**
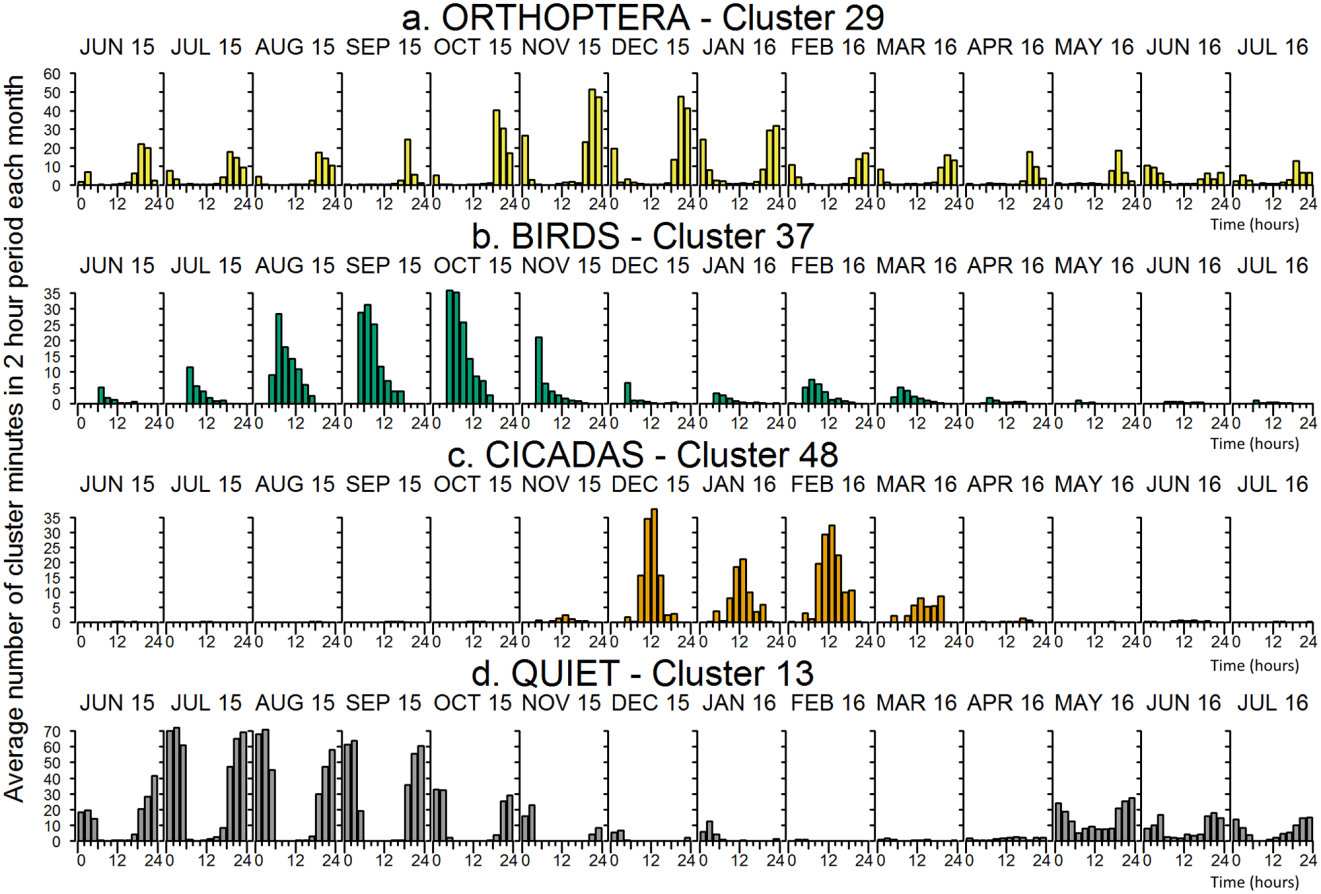
Two-hour plots showing the average number of minutes per month (June 2015 to July 2016) in each two hour period throughout the day at Woondum National Park (See S5 File: Cluster statistics for two-hour plots of each of the 60 clusters).

Acoustic communities calling in the day differ from those calling at night [35]. Therefore, it is expected that the temporal distribution of a cluster will indicate its acoustic class. For example, clusters containing quiet or orthoptera are expected to occur mostly at night; bird clusters should mostly occur during the day; and rain and wind clusters could occur at any time but will vary seasonally. Two methods were chosen to display the temporal distribution of the 60 clusters in order to reveal daily and seasonal patterns: histograms and rose plots.

i **Histogram plots**: The average number of times that one-minute instances belonging to a cluster occurs in each two-hour period during the day in each month is shown in Fig 4. The chart for each month contains twelve bars (one for each 2-hour period) and the average is calculated over the total number of days in that month.

Fig 4 displays the 24-hour distribution of four acoustic clusters (#29, 37, 48 and 13) by month, through the year. The distribution of a cluster over a 24-hour cycle or seasonal cycle gives a strong indication of its acoustic content. For example, cluster 29 labelled “*Orthoptera*” has its maximum occurrence in the summer months during the evening and early morning and its minimum occurrence during winter. This is consistent with the known calling behaviour of Orthoptera at these locations. Cluster 37 is at a maximum during spring mornings and almost absent in winter months, consistent with it being labelled *bird morning chorus*. Cluster 48 occurs only during the summer months and is dominant towards middle of the day, consistent with its label, *midday cicada calls*. As is to be expected, the *quiet* cluster 13 dominates at night, but of interest is that it is almost absent during summer months.

**Fig 5:**
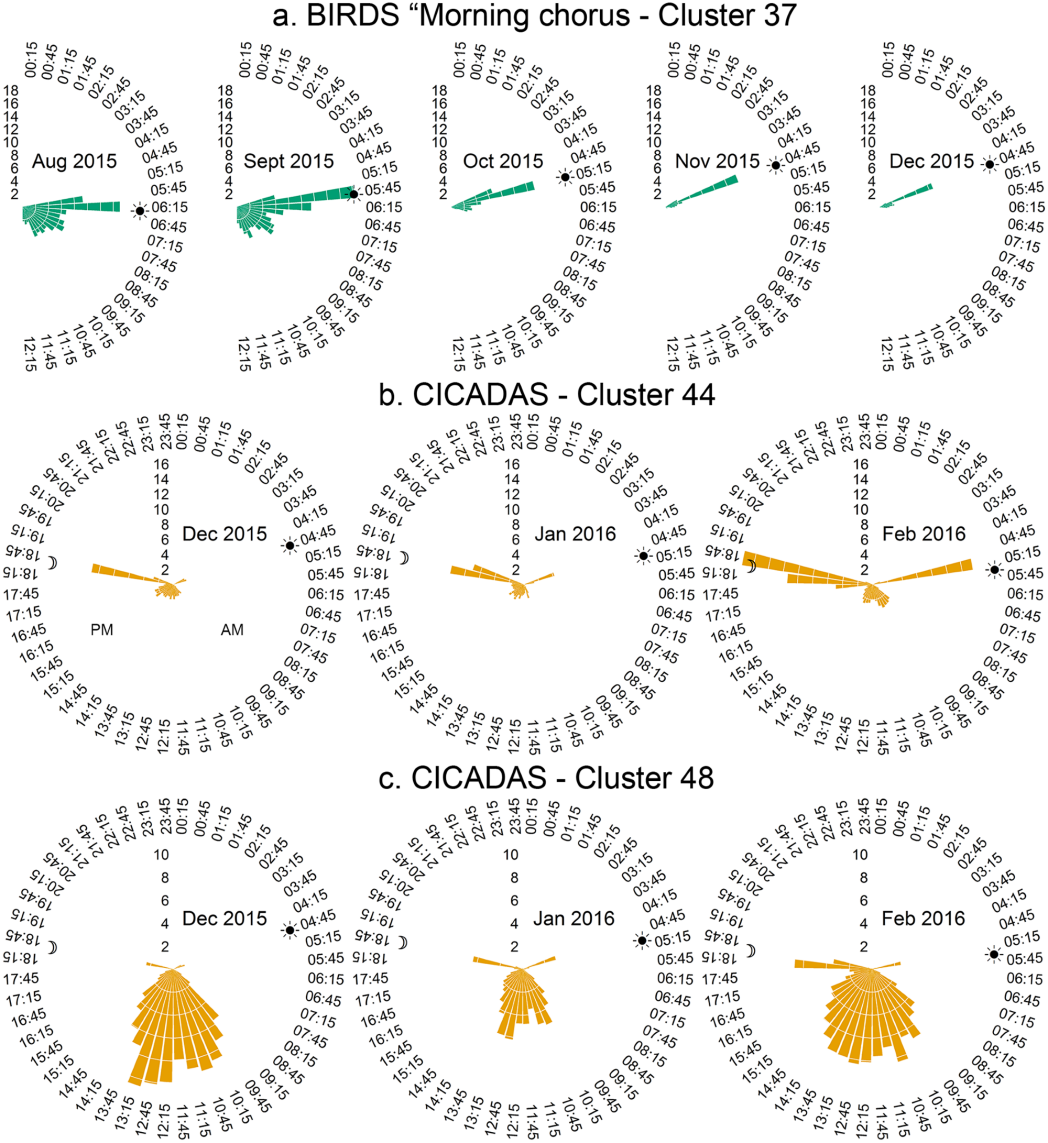
Rose Plots of the average number of cluster minutes per half-hour in each month. (a) BIRDS (Cluster 37) at Gympie NP. (b) and (c) CICADAS (Clusters 44 and 48) at Woondum NP. The sun and moon symbols mark the time of sunrise and sunset on the 15^th^ day of each month. Rose plots produced using code adapted from [37].

It should be noted that the scale on the y-axis on each of the plots is different. The maximum possible number of minutes in the period is 120 minutes (2 hours). Cluster 13 exceeds or approaches an average of 70 minutes in the two-hour period before midnight during July and August. This indicates that Cluster 13, which is one of several ‘quiet’ clusters, occupies at least 50% of the time during the periods around midnight during winter. Cluster 37 a ‘bird’ cluster occupied 30% of the 2-hour period between 4 and 6 am during October 2015 indicative of the bird dawn chorus. For the temporal distributions of each of the 60 clusters, see S5 File: Cluster statistics.

ii **Rose plots**: We used rose plots to display the average number of times that a cluster instance appears in each half-hour period of the day. In this case, the rose plot contains 48 ‘petals’ per 24-hour cycle and the average is calculated over all the days in the month. These rose plots give a more fine-grained view of cluster distribution than the above histograms. The morning chorus cluster 37 (Fig 5a) for example, is seen to occur mostly in the half-hour before sunrise. The peak of this cluster synchronises with the time between civil-dawn and sunrise over these months. In addition, the average number of ‘morning chorus’ minutes is at a maximum in September corresponding to the height of the breeding season for birds common to this area [36].

Cluster 44 (Fig 5b) captures a dawn-dusk cicada chorusing, which, during February 2016 occupied 45% (on average) of the temporal sound space at dawn and 58% at dusk. The intensity of the cicada chorus during these periods disrupts bird and other insect chorusing. Cluster 48 at Woondum NP (Fig 5c) captures cicada midday calling which also dominates the temporal soundscape at that time.

To summarise, the temporal distribution of cluster occurrences through 24-hours of a day and through seasons of the year, is consistent with the labels given to them by listening to small samples from each cluster.

#### 3. Sammon map

**Fig 6:**
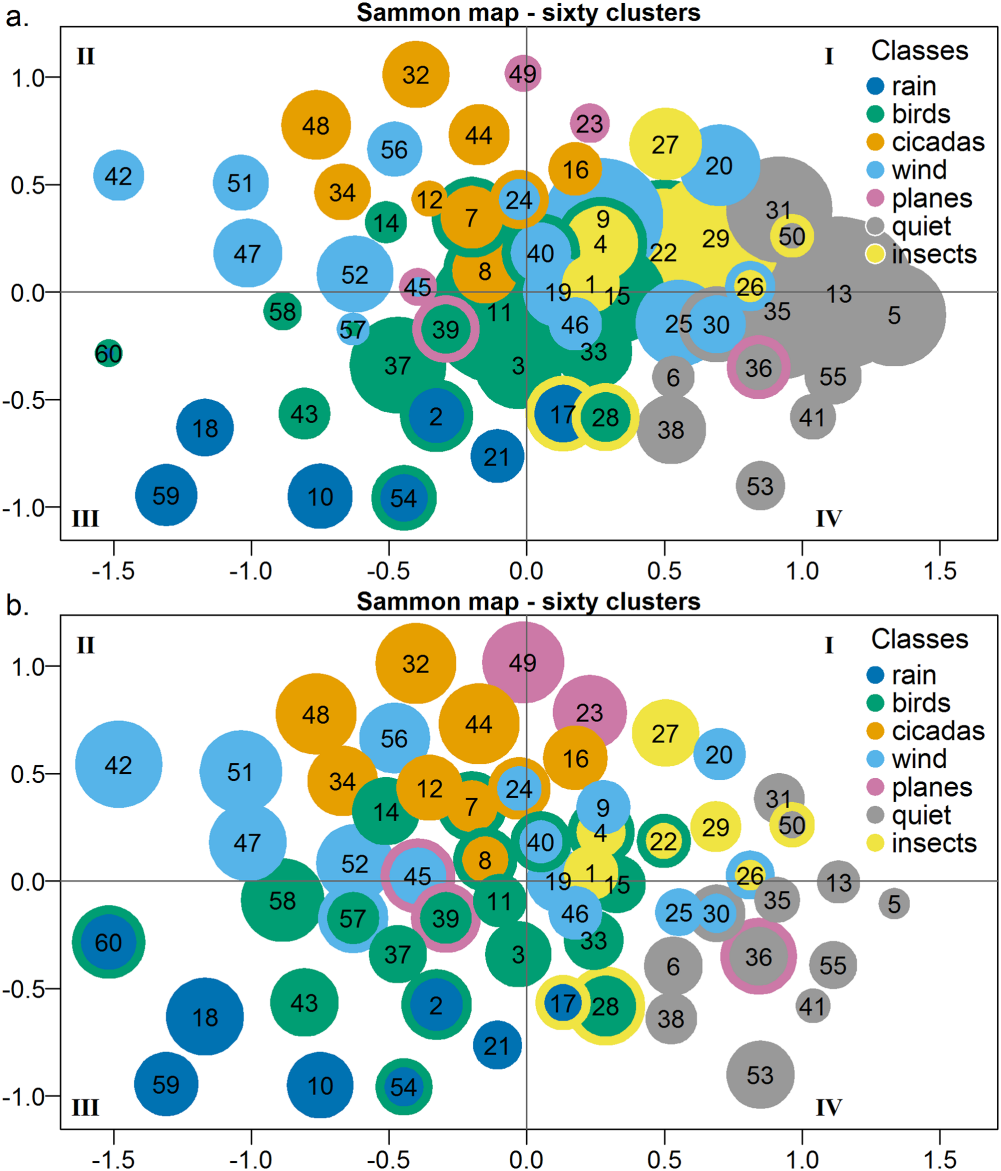
(a) Sammon map of the cluster medoids. (Drawn using R plotrix package [38].) Circle diameters in top image (6a) are proportional to the number of instances in each cluster. (b) Circle diameters in the bottom image (6b) are proportional to the Euclidian distance from the cluster medoid that encompasses 90% of the cluster instances. The colour codes have been selected from a colour blind pallet [39].

A Sammon map of the 60 cluster medoids (Fig 6) projects the medoids onto a two-dimensional surface while preserving relative inter-medoid distance. In the top map (Fig 6a) the circle radius indicates the relative cluster size (number of one-minute instances in each cluster) and the circle colour indicates the dominant acoustic class. Circles with borders in a different colour indicate co-dominant sound sources. In the bottom map (Fig 6b), circle radius indicates the relative radius of the cluster hypersphere that encompasses 90% of cluster instances, centred on the cluster medoid. Note that those clusters containing fewer instances tend to lie on the periphery of the map (Fig 6a), and they also tend to occupy a relatively larger volume of feature space (Fig 6b). Clusters 60, 58, 42, 45 and 53 are examples.

The distribution of clusters in the Sammon maps tends to confirm the class labels assigned by listening to the relatively small number of sampled recordings. Clusters containing the same dominant sound source are generally grouped together within the Sammon map. Clusters containing codominant sound sources tend to sit between clusters of the equivalent single sound sources. The exceptions to this general observation are the wind and plane clusters.

The ‘quiet’ clusters are on the right side of the map whereas the loudest are to the left, suggesting that the x-axis distributes clusters according to amplitude. The largest bird clusters are distributed around the centre of the Sammon map away from the extremes of other acoustic clusters. The exceptions to this rule are the loud, morning chorus clusters (43 and 37) which are located to the high-amplitude, left-side of the map (Fig 6). The diagonal from bottom-left-to top-centre of the Sammon map appears to distribute clusters on bandwidth–the *Cicada*, *Orthoptera* and *Plane* clusters tend to contain narrow bandwidth events, while rain events (bottom-left) are broadband, due to percussive effects of rain drops hitting the recording box.

#### 4. Comparison of cluster medoids

**Fig 7:**
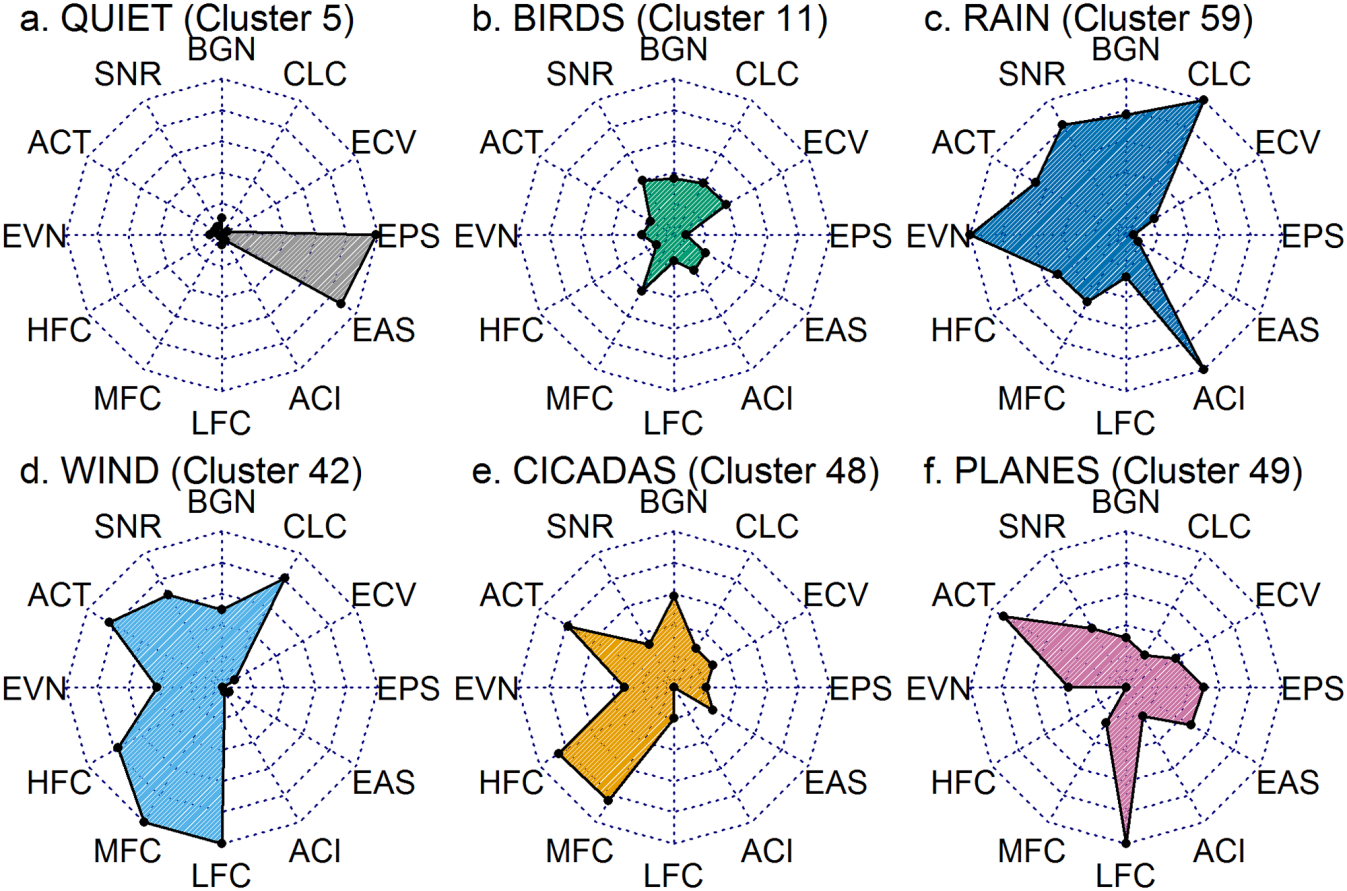
Radar plots of the medoids of some important acoustic clusters. Produced using the R package ‘fmsb’ [40]. For more radar plots see S5 File: Cluster statistics.

As a final confirmation of cluster content, the cluster medoids can be illustrated using Radar Plots (Fig 7), which facilitate cluster interpretation in terms of the original acoustic indices. Here we have selected six clusters representative of major classes of acoustic event. It is also helpful to correlate these radar plots with the corresponding cluster location in the Sammon Map (Fig 6).

The medoid values (Fig 7a) of Quiet’ cluster 5 (located on the right side of the Sammon Map, Fig 6) show high values of Entropy of Peaks Spectrum (EPS) and Entropy of Average Spectrum (EAS). In our treatment of the entropy indices, a high value indicates ‘concentration’ of energy. Typically, in an extended period of quiet audio there will be one brief click or chirp that contains most of the acoustic energy and hence a high relative energy ‘concentration’, despite the audio containing low total acoustic energy.

The medoid of cluster 11, the largest of the bird clusters, is located near the centre of the Sammon Map and many of its indices fall in the middle range (Fig 7b). Cluster 59, the heavy rain cluster at the bottom left of the Sammon Map (Fig 6) has high values across Background Noise (BGN), SNR, Acoustic Complexity Index (ACI), Events (EVN) and Cluster counts (CLC) (Fig 7c) indicating a very complex and dynamic soundscape. Wind cluster 42 (Fig 7d) and other wind clusters at the top left of the Sammon map have broadband content (high values for LFC, MFC and HFC) and low values for the entropy indices (EAS, EPS and ECV) implying that the acoustic energy is distributed through both frequency and time.

Cicada cluster 48 has high and mid-frequency content and high value for Activity (ACT) due to the continuous nature of cicada chorusing (Fig 7e). By way of contrast, the Plane cluster 49 (Fig 7f) has low frequency content only, but also a high value for ACT due to continuous character of plane noise as it passes overhead, because ACT is a measure of the fraction of time the decibel envelope is above 3 dB. See S5 File: Cluster statistics for radar plots of the medoid values of each of the 60 clusters.

### Visualisation of the year-long recordings

This section describes the results of three visualisation techniques used to display the acoustic state sequences obtained from the clustering. The three techniques are diel plots, polar histograms and PCA plots.

#### 1. Diel plots

**Fig 8:**
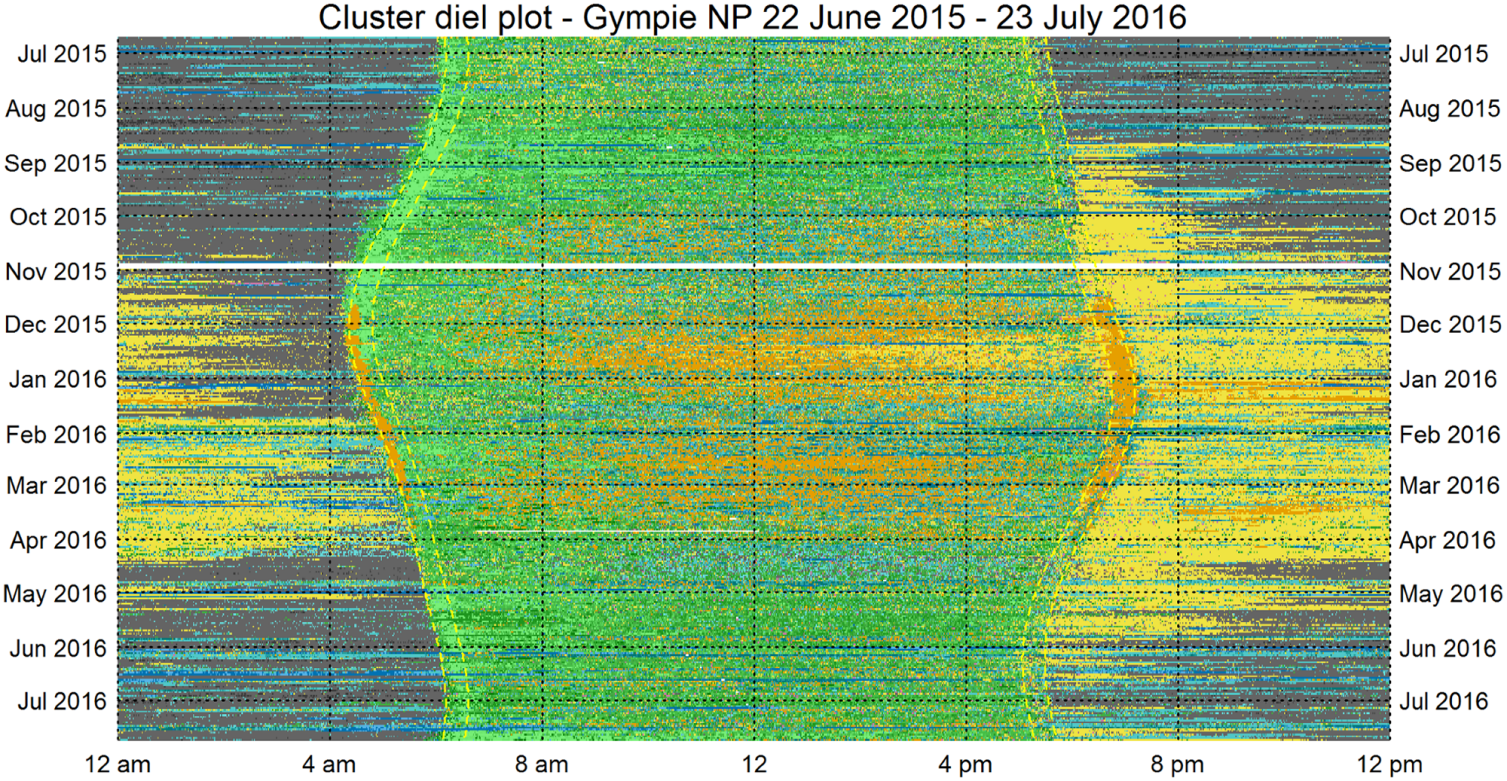
Diel plot for 13 months of audio recording obtained from Gympie NP. The dotted yellow lines mark the civil dawn, sunrise, sunset and civil sunset from left to right. The colour interpretation for these images is: Green–birds, Yellow–Orthoptera, Orange–Cicada, Light blue–Wind, Dark Blue–Rain, Grey–Quiet, Pink–Planes.

**Fig 9:**
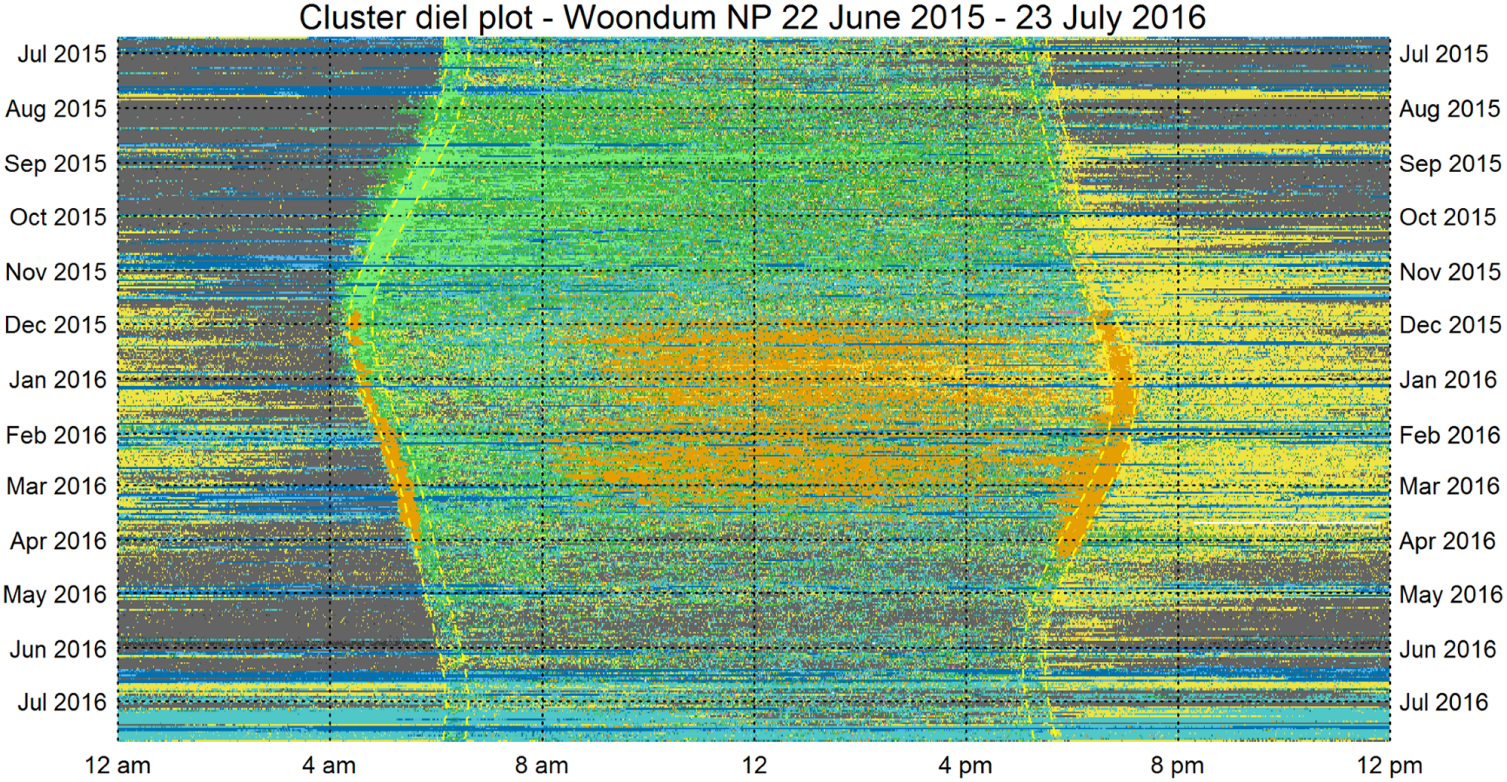
Diel plot for Woondum NP displaying the acoustic states across 398 days.

We visualised the thirteen months (398 days) of continuous acoustic recording using diel plots (Figs 8 & 9). Each cluster represents a discrete acoustic state of duration one-minute. Thus, once meaningful class labels are assigned to the clusters, an entire day of recording can be represented as a sequence of 1440 acoustic states. Each diel plot has 1440 columns (one column for each minute from midnight to midnight) and 398 rows (one for each day, starting from the top with 22 June 2015, the day after winter solstice). For visualisation, it is helpful to colour-code the clusters according to the similarity of their acoustic content. We continue to use the same colour-code used for the Sammon plots in Fig 6, except that the two morning chorus clusters (37 and 43) are now coloured lighter green to distinguish them from the other bird clusters. These images represent a 10^**6**^ data reduction and yet retain important ecological information.

Note that it is possible to locate the starts and ends of rain periods (dark blue bands) to the nearest minute without the use of rain recording equipment. Note also, that it would be possible to estimate rainfall volume from the sound intensity heard in each ‘rain’ cluster. From August to November 2015, the ‘morning chorus’ clusters (37 & 43, shown in a lighter green) coincide with the period between civil dawn and sunrise. The white bands indicate unrecorded minutes, due to battery changes, file corruption or minutes removed because of microphone problems (28-30^th^ October 2015 at Gympie NP).

Cicada chorusing (in orange) commenced in late November when it interrupts the bird morning chorus. When cicadas ceased their morning chorus around March 2016, an uninterrupted bird morning chorus resumed. Cicada chorusing is also prominent in the middle of most summer days. Orthopteran chorusing (in yellow) occurs predominantly at evening and night during late spring, summer and early autumn. A particular advantage of these diel plots is that visual comparisons can be made between the two sites (Gympie–Fig 8, and Woondum–Fig 9). At a glance it can be seen that the cicada chorusing is more prominent at Woondum, but Orthopteran chorusing is more prominent at the Gympie site.

#### 2. Polar histograms

**Fig 10:**
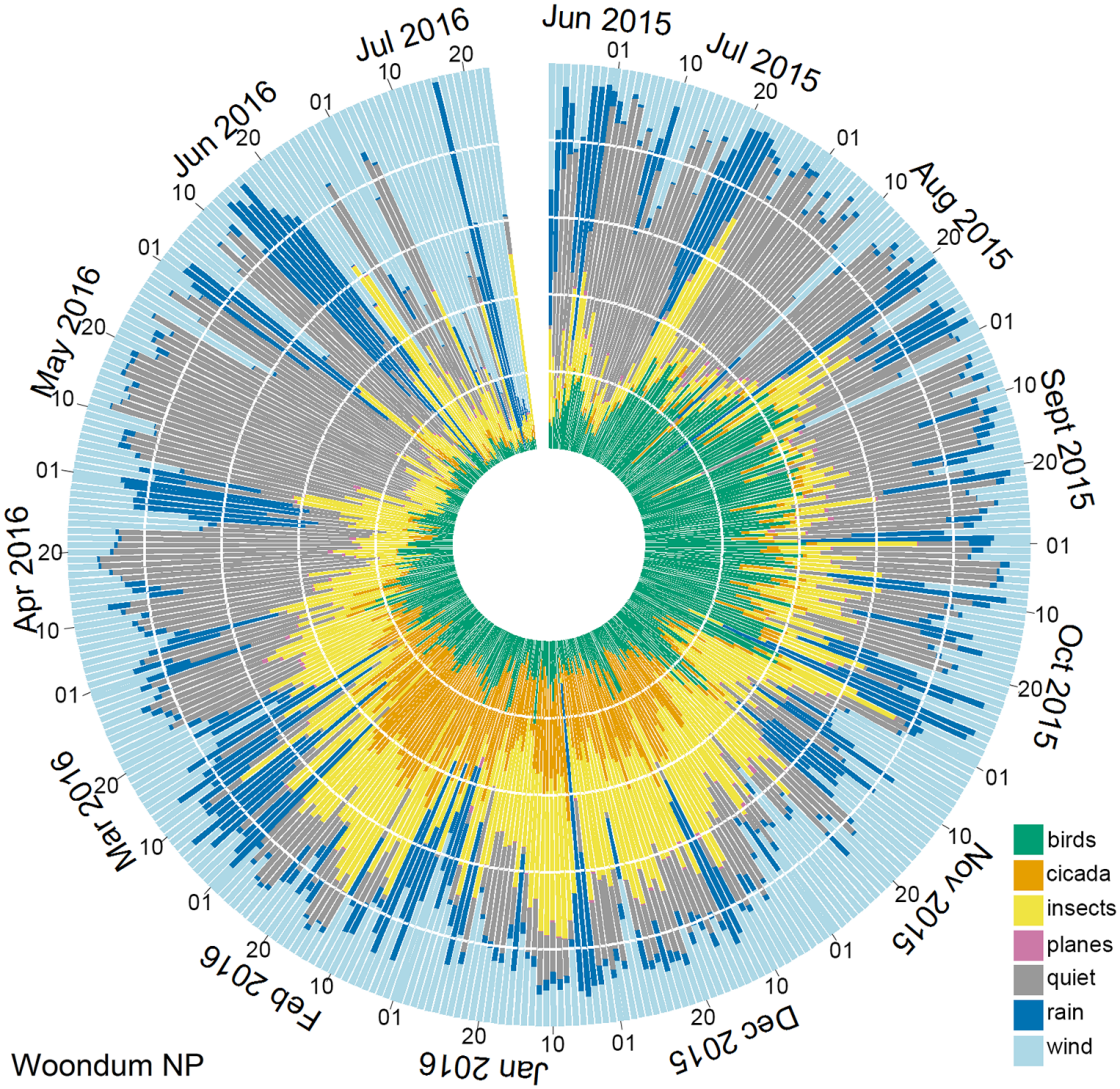
Polar Histogram of 13 months of recording (22 June 2015 to 23 July 2016) at Woondum. The plot was produced using R code adapted from [37]. For the polar histogram from the Gympie site see S5 File: Cluster statistics.

Polar histograms (Fig 10) were employed to display the proportion of minutes per day in each acoustic class over a 13-month period. It provides a useful overview of broad-scale changes in the soundscape with season. The dominance of birds in spring and of insects and cicadas in summer is easily observed. The polar histogram reveals an association between rain periods (dark blue prominent in the histogram) and Orthopteran activity (yellow).

**Fig 11:**
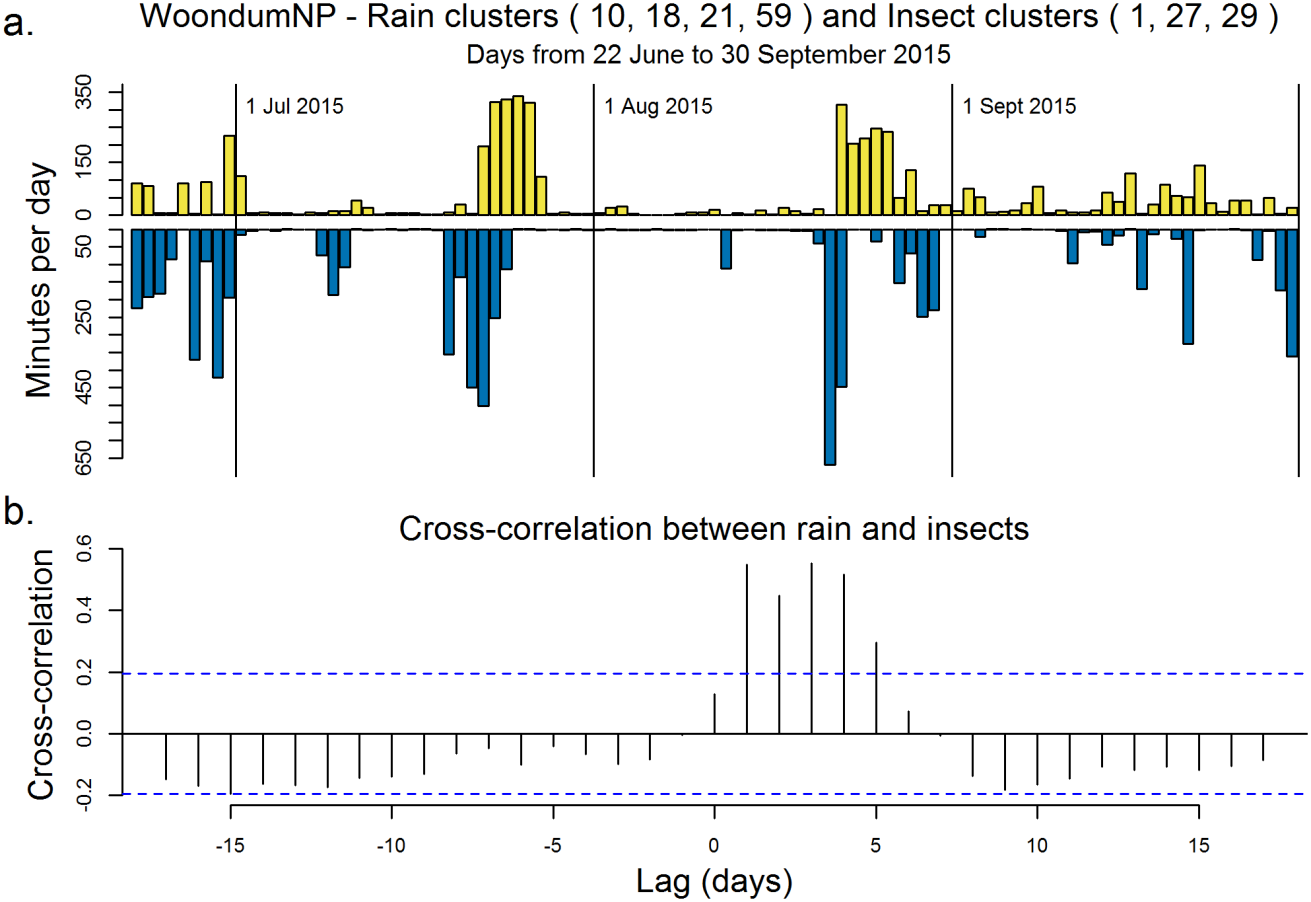
Relationship between the minutes of rain and the subsequent minutes of insects. (a) The number of minutes per day (over 100 days) of ‘insects’ (clusters 1, 22, 17, 27 combined) and ‘rain’ (clusters 10, 18, 54, 59 combined) at Woondum National Park. (b) Cross-correlation between the ‘rain’ and ‘insect’ classes versus the lag in days. The dotted lines give the 95% confidence interval.

The association between rain and insects is further explored in the back-to-back histogram (Fig 11a). This plot clearly reveals a lag of about one day between the onset of rain and an increase in subsequent Orthopteran calling (Fig 11b). This association is most evident in winter months. In summer months (December to February), Orthoptera dominate the soundscape regardless of rain. Polar histograms have additional uses, for example, to identify a succession of days with minimal wind and rain.

### Cluster cycles

**Fig 12:**
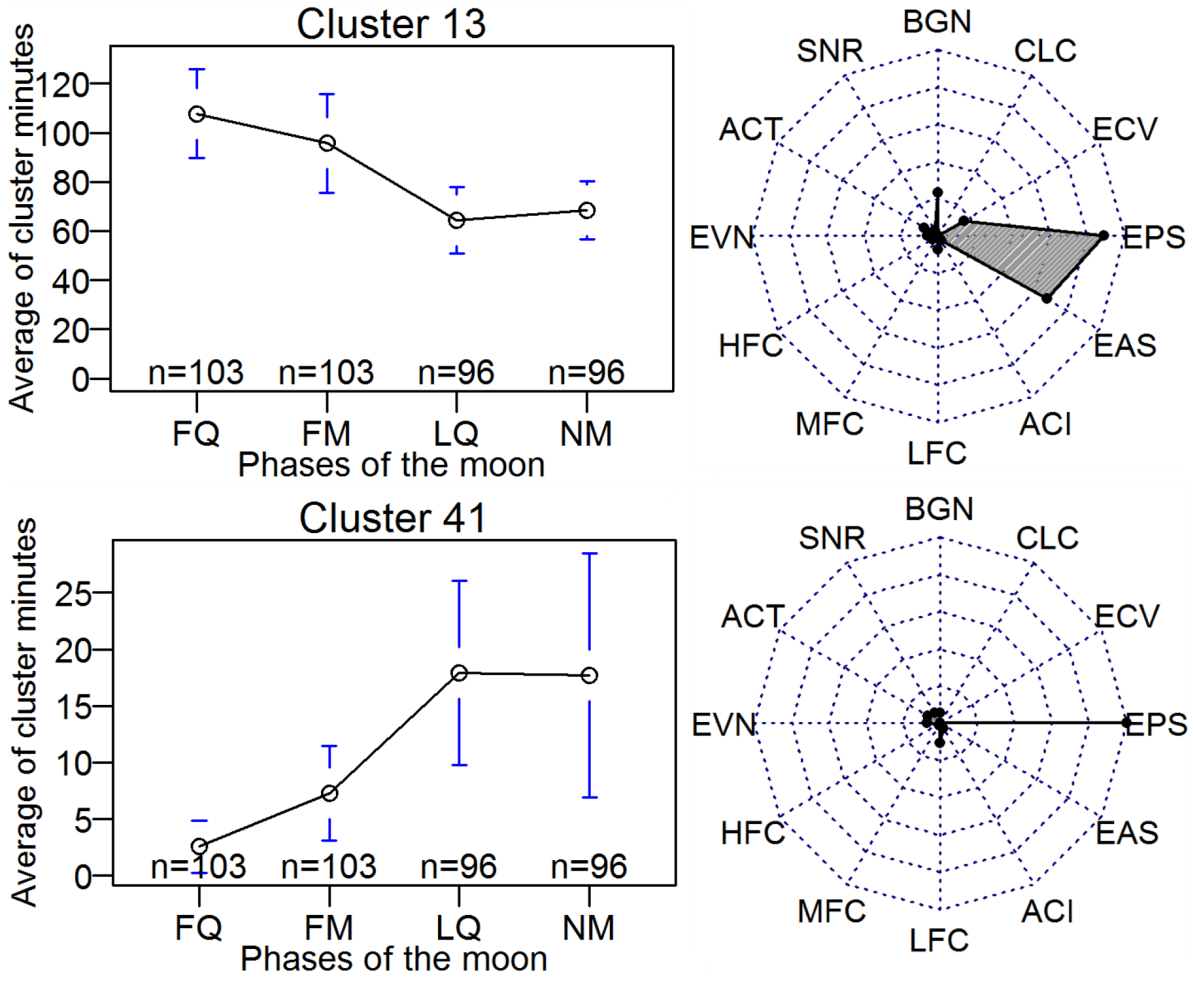
Left side: The average number of times in a 24-period that cluster 13 (top) and cluster 41 (bottom) are present during the lunar cycle. (Averages are over the 13 lunar months of the study period.) Right Side: Radar plots illustrating relative index values for the medoids of the corresponding clusters.

**Fig 13:**
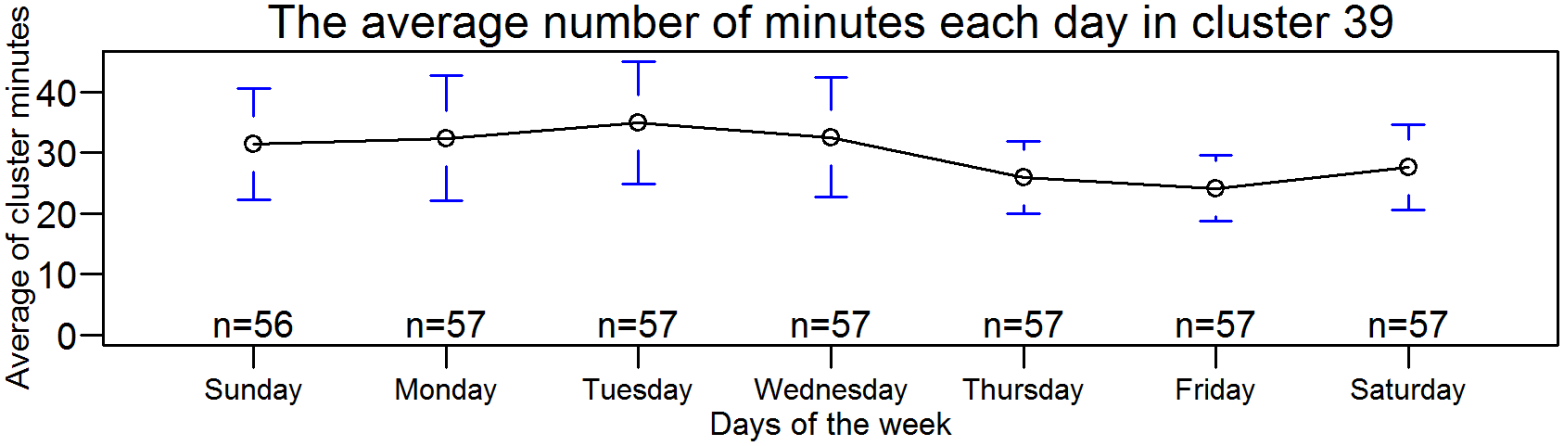
The average number of times cluster 39 is present in a 24-period versus days of the week. (Averages are over 56 weeks of the study period.) Cluster 39 was previously identified as containing co-dominant bird and plane sounds.

In addition to the expected annual and 24-hour cycles of some cluster distributions, such as observed in Fig 4, we also discovered two clusters having a lunar cycle (clusters 13 and 41, Fig 12) and one cluster having a weekly cycle (cluster 39, Fig 13). Cluster 41 was the quietest of the clusters labelled Quiet and occurred more frequently in the last quarter of the lunar cycle. By contrast, cluster 13, the largest of the Quiet clusters, was pre-dominant in the first-quarter. The radar plots (right side, Fig 13) indicate that cluster 13 had higher values for EAS and BGN, consistent with slightly elevated levels of insect sound to be heard in cluster 13 compared to cluster 41 recordings. Lunar periodicity of insect life cycle and behaviour has been noted for a number of species [41].

Of the 60 acoustic states/clusters, only cluster 39 exhibited a seven-day distribution cycle, with a minimum on Friday (Fig 13). Cluster 39 was previously identified as containing co-dominant bird and plane sounds. We did not attempt to quantify the number of airplane passes but note that the recording sites were relatively close to long-distance flight paths.

### Challenges faced

As might be expected over the course of 13 months of recording, problems were encountered with microphone deterioration and recording quality. This was most evident following periods of heavy rain. The microphones were covered with a foam wind-sock provided by the manufacturer and were subject to wind, rain and sun. We did not protect our microphones from exposure by using a roof or silicon spray. The recordings were made in stereo and fortunately, there was only one short period when both microphones failed simultaneously. One microphone on each recorder was changed halfway through the recording year.

We attempted to automate the detection of microphone degradation by comparing zero-crossing rates and other indices for left and right channels. These tests worked some of the time but not consistently. In the end, we listened to recordings at regular intervals, rejected a degraded channel and, for consistency, all acoustic indices were recalculated using only the unaffected channel.

**Fig 14:**
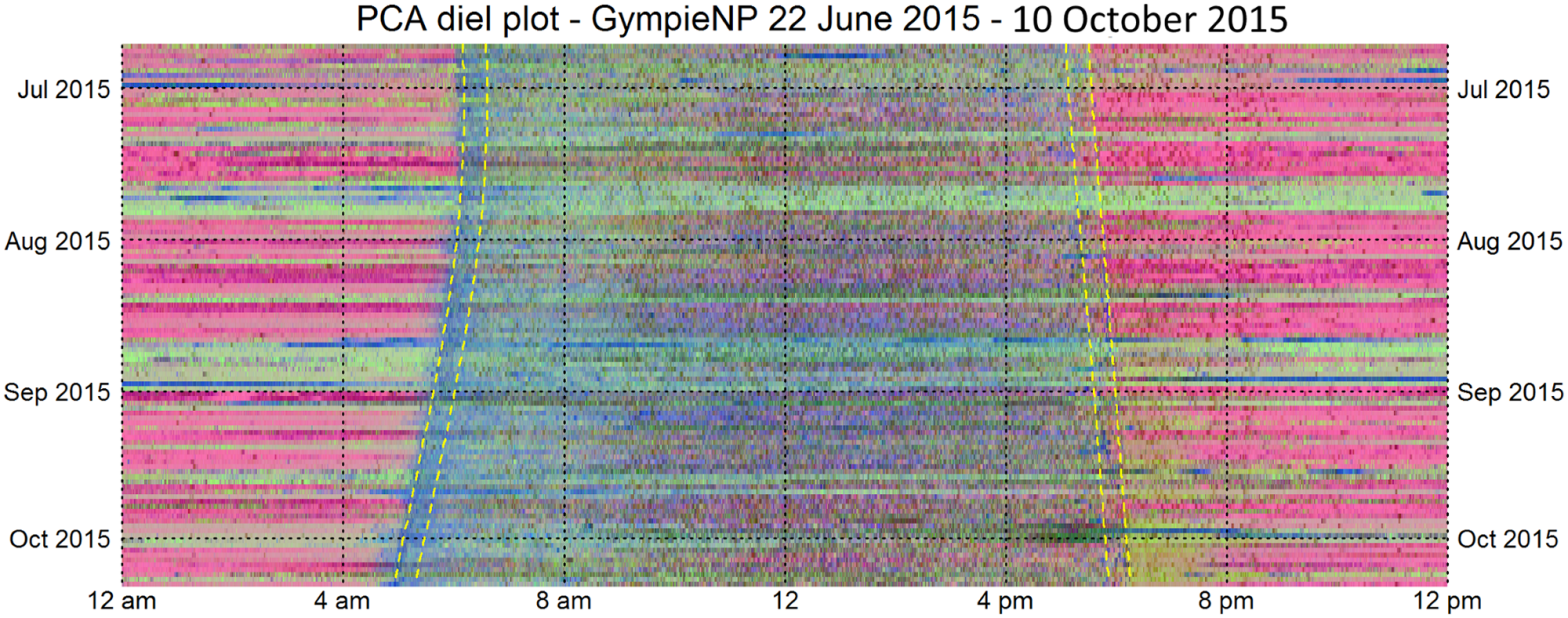
PCA diel plot illustrating the first one hundred and eleven days (22 June 2015 to 10 October 2015) of acoustic recording at Gympie NP. The civil-dawn, sunrise and sunset, and civil sunset times are marked in yellow.

We did however develop a useful technique to *visualise* the effects of microphone degradation using coloured diel plots derived from Principal Components Analysis (PCA). The left and right channels were averaged before calculating the acoustic indices. PCA was performed on vectors of acoustic indices normalised in [0, 1]. The first, second and third principle components were rescaled to full colour range [0, 255] and these values were mapped to the red, green and blue channels of a diel plot (Fig 14). As with the previously described diel plots, the resulting image is 1440 pixels wide representing twenty-four hours (midnight to midnight) and one row per day starting from the top. These PCA diel plots show light horizontal banding corresponding to periods where one of the microphones was degraded. Note that these degraded periods were typically preceded by heavy rain (shown in dark blue).

PCA diel plots are a fast technique for detecting recording problems because they highlight the basic structure of a recording without the need to cluster feature vectors. This PCA diel plot (Fig 14) shows the morning chorus in blue, insect chorusing in yellow and afternoon thunderstorms during late September in dark green. Obviously, these colours will change depending on the data and choice of acoustic indices and the choice of which of the three principal component coefficients are mapped to which channel.

## Discussion

Audio recordings of the environment are an increasingly important technique to monitor biodiversity and ecosystem function. Despite the fact that not all animals vocalise, the major animal taxa which do vocalise, birds, frogs and insects, turn out to be important indicator species. While the acquisition of long-duration recordings is becoming easier and cheaper, the management, analysis and interpretation of the audio remains a significant research issue.

The key issue addressed in this paper is how to reduce environmental audio for visualisation, yet retain ecologically meaningful content. We have described a clustering technique which reduces audio data by six to eight orders of magnitude, yet retains ecologically relevant information. We have also described a number of visualisation techniques that assist interpretation of environmental audio. We discuss our contributions under five headings.

### 1. What do acoustic indices mean?

Acoustic indices were introduced some ten years ago as a tool to assist ecological investigation of species diversity [42]. At first, it appeared as if these were a new kind of index, that opened up new possibilities for ecological investigation. Developments in the field over subsequent years were not well informed by the established fields of signal processing and machine learning. Acoustic indices were assumed to have singular ‘ecological’ relevance. We would assert that there is no fundamental difference between an acoustic index (as developed by ecologists) and an acoustic feature (such as used in speech processing). The principle difference is the time scale over which the features are typically calculated (seconds or minutes versus milliseconds, a gap of some three orders of magnitude). All the acoustic indices used in this paper (or variants of them) have been used as acoustic features by other (bioacoustic) researchers for a variety of machine learning tasks, such as bird call recognition. The one exception to this is the ACI index, which appears not to be a useful acoustic feature for recording segments less than about 15 seconds duration and which has no theoretical roots in the signal processing literature. Yet the published literature on this index suggests that it is useful in the *ecoacoustic* context.

One of the contributions of this paper is to link ecoacoustics research to the long tradition of signal processing and machine learning and to promote the idea that soundscape processing requires the extraction of multiple acoustic features (indices), just as speech processing requires the extraction of multiple acoustic features. The important difference is in the time scale, because soundscape phenomena can have a temporal scale of days, months or even years.

### 2. Efficient clustering of environmental audio

We have described a two-step hybrid clustering technique which first uses *k*-means clustering to reduce audio data (in the form of 1,141,147 vectors of acoustic indices) to a large number (*k*1) of small clusters, followed by a second, hierarchical clustering step, which combines the *k*1 clusters into a smaller number (*k*2) of clusters that partition major sources of biophony (birds, insects, cicadas) and geophony (wind and rain). This technique attempts to take advantage of the best properties of the two algorithms. We note that, following calculation of 26 months of acoustic indices (on an HPC), the subsequent clustering steps described in this paper were performed on a standard laptop (16 GB RAM, 2.1 GHz Intel^®^ Core TM i7). For the R code used to perform the hybrid clustering and the visualisations in this paper see https://github.com/QutEcoacoustics/plos-visualization-paper.

We consider another important contribution of this paper to be the method by which *k*1 and *k*2 were optimised to produce ecologically meaningful clusters. The standard Dunn index gave ambiguous results and the Silhouette index indicated that the data did not contain sufficient structure to justify clustering.

Our method depends on the observation that *acoustic signatures* (in the form of histograms of cluster frequency over a 24-hour period) offer informative summaries of one day of recording [13]. Here, “informative” means that days having similar biophony yield more similar signatures than days having dissimilar biophony. We chose twelve days of recording (two sites × two times of year × three almost consecutive days) as the basis for an ‘error’ function to obtain the optimum values of *k*1 and *k*2. It is worth noting that, even though no clustering run ever produced an ‘ideal’ partitioning of the twelve days into four groups of three (day 12 was always incorrectly grouped; see Fig 3b), this ‘target’ nevertheless yielded a satisfactory clustering result.

### 3. Cluster interpretation

Listening to the audio was the obvious first choice to determine the acoustic content of the 60 clusters. However, this is a very time-consuming task. Even a small sample of just 20 minutes from each cluster required more than 20 hours of listening. We therefore adopted three additional methods for confirmation of cluster content.

First, was the examination of the temporal distributions of the clusters (Figs 4 and 5). These provided supporting evidence that clustering had ‘captured’ the natural daily and seasonal cycling of the vocalising taxa. For example, the daily peak in bird calling during the morning chorus evident in cluster 37 (Fig 5a) or the seasonal patterns of the orthopthera, birds and cicadas (Fig 4).

Second, was an examination of the distribution of the clusters in the Sammon map where it was found that clusters containing similar acoustic sources were located close to each other. The main exceptions to this were the wind clusters, which is not unexpected because wind can vary from strong sustained gusts to a gentle breeze. Strong wind clusters (42, 47 and 51) were located to the left (high amplitude) side of the Sammon map, whereas gentle wind clusters (e.g. 25, 46) were located to the right (low-amplitude) side of the map.

Given the low Silhouette scores (Fig 2b), the question arises as to whether clustering is an appropriate technique to compress and categorise acoustic recordings of the environment. Clustering does not guarantee ‘meaningful’ clusters, particularly where the underlying data distribution is continuous or near-continuous. Clustering is most appropriate when there is an underlying ‘structure’ in the data set, even if partially hidden by noise. We expect rain and wind to have continuous distributions from light to heavy or weak to strong. Clustering in these cases is likely to impose structure on essentially continuous distributions, which is consistent with the low Silhouette scores.

On the other hand, clusters 44 (dawn & dusk cicadas) and 29 (night time Orthopteran chorusing) have precise temporal distributions (Figs 4 & 5) that are consistent with the expected calling behaviour of those taxa. In other words, these clusters do have a discrete ecological interpretation which justifies the clustering process. In addition, the eight ‘bird’ clusters, which together constitute an ecologically discrete group, are clustered together towards the centre of the Sammon map.

It is interesting that almost 75% of the total soundscape over 13 months at the two sites was dominated by a single sound source at any one time (Table 2), divided approximately equally between ‘quiet’, ‘wind’, ‘birds’ and the remaining four classes. While the decision as to whether a one-minute recording contained a dominant source versus two or more equal sound sources was based on the listening experience of one of the authors (YP), there are logical reasons why this might be so. Silence is imposed by cold winter conditions at night and windy conditions tends to reduce the frequency of bird vocalisations. Nevertheless, one might have expected more bird calling activity in conjunction with orthoptera and cicadas. The abundance of acoustic states with a dominant sound source is perhaps a demonstration of the temporal partitioning of the soundscape between the major taxa of vocalising animals as proposed by the *acoustic niche hypothesis* [43]. Cicada choruses, for example, can be so loud as to exclude other vocal taxa from the soundscape. But tempering this interpretation is the observation that Orthoptera were calling in most of the one-minute segments most of the time–but they were not always a dominant or co-equal sound source.

As a final comment on the appropriateness of clustering environmental audio, we note that only seven clusters out of the 60 (clusters 17, 24, 28, 36, 40, 50 and 57 containing some 7% of one-minute segments) had inconsistent acoustic content. We interpret these cluster as “falling between the clustering cracks”. For example, cluster 57 falls between Wind cluster 52 and Morning Chorus cluster 37. Cluster 50 falls between Quiet cluster 31 and Insect cluster 29 (see Fig 6).

To summarise the above observations, recordings of the environment contain acoustic attributes that appear to vary continuously, but also attributes that suggest the existence of discrete acoustic sound sources. Our results indicate that there was sufficient discretely-structured biophony in our recordings for acoustic clustering to be a useful technique. And even the imposition of discrete structure onto continuous distributions (as in case of rain and wind) appears to be a useful device for visualising recordings of the environment.

Finally, we suggest that another practical application for clustering of environmental recordings is to remove recording segments dominated by geophony and anthropophony. These are usually removed manually before analysis [35] but this can be a very time-consuming process. Acoustic clustering has the potential to automate the removal of unwanted portions of a recording at a resolution of one-minute. Alternatively, the identification of rain and wind can lead to directly to important observations. In our work, the identification of rain clusters led to an immediate observation of an apparent relationship between rainfall and subsequent insect chorusing in winter months (Fig 11).

Although anthropogenic sound was not a significant sound source in our recordings, studies of the effect of anthropogenic sounds on animal calling behaviour [44, 45] may benefit from clustering to locate acoustic states due to biophony and anthropophony.

### 4. Visualisation

We consider another useful contribution of this paper to be visualisation techniques that are vital for the interpretation of long-duration acoustic recordings. The 13-month diel plots (Figs 8 and 9) have multiple uses. They could be used for navigation in a user-interface where clicking on a pixel takes the user to the corresponding one-minute spectrogram at standard scale. They could be used, like the polar plots in Fig 10, to provoke questions and prompt further analyses. We believe that comparing year-long diel plots over multiple years could be an important technique to monitor habitat degradation, habitat restoration, changes in species abundance and the effects of climate change. Such changes can be subtle, as for example, where there is no immediate loss of species but there are changes to the underlying ecological processes due to shifts in breeding and migration times [46]. Our visual techniques could detect differences between two natural woodland sites only 25 km apart, even though many of the tree species and dominant vocal species are common to both sites. In such cases, a comparison of species lists would not necessarily reveal differences.

### 5. Recording protocols

One of the little mentioned issues in published research on audio recordings of the environment is degradation of microphone performance. However, Blumstein et al. [47] comment on the need to protect microphones from rain and humidity. Since most studies manually remove recordings containing wind and rain, we can assume that the same filtering process will remove recordings degraded for other reasons. If long duration recordings of the environment are to become an accepted methodology, it will be necessary to develop protocols for microphone care. A simple protection measure would be a roof over the recording unit sufficient to protect the foam wind-socks from direct rain. The percussive effects of rain on the roof can be minimised with a layer of foam. It would be helpful to have two sets of microphones so that these can be alternated when batteries and SD card are changed. While these measures add to recording cost, it should be remembered that replacing degraded microphones is also a significant cost, not to mention the very real risk of total data loss. Labs that are doing a lot of recording should consider setting up standard tests for microphone integrity. We are aware of one Australian company that is working on producing such a testing unit. It is worth noting that in the work reported here, the built in channel redundancy of having stereo channels, saved many days of recording.

## Conclusion

We have described a two-step hybrid clustering technique which greatly compresses audio data while retaining ecologically relevant acoustic information. The method reduced 26 months of recorded audio to a set of 60 acoustic clusters, most of which could be given a discrete interpretation in terms of a dominant sound source: biophony, geophony and anthropophony. Once acoustic data is clustered, many analytical and visual techniques can be applied, to facilitate navigation of long-duration recordings of the environment.

There are at least two major future directions for this work. The first is to determine to what extent the clustering results obtained from one site can be used to classify acoustic data from an unrelated site. This would greatly extend the generality of our technique. The second direction is to develop new acoustic features/indices that highlight various soundscape features. We have begun to do this by developing indices derived from spectral ridges and spectral peak distributions. But there is certainly much more that can be done here. The objective would be to extract acoustic features (indices) that are appropriate to the ecological questions being asked. This in turn, will depend on the sound sources in the recording, which indices are used in combination and the clustering technique. For example, in our case, six of the twelve acoustic features were extracted from the mid-frequency band (1000–8000 Hz) because we knew beforehand that bird and insect vocalisations were the most important biophony at both sites. However, if frog chorusing was expected to be an important component of biophony, then additional relevant features could be extracted from the low frequency band.

Finally, the question arises as to whether the cluster optimisation technique described in this paper could be used with other types of data that may not be time-series or acoustic. Our objective was to bias clustering towards separating acoustic events that are discrete (e.g. insect versus bird vocalisations) even where these are embedded in other acoustic events having continuous distributions (e.g. rain, wind and degrees of silence). Such data is clearly complex and continuous distributions are not usually considered a candidate for clustering. However, we had terabytes of acoustic recording and some form of data reduction by quantisation was essential. To determine a suitable cluster number, we measured performance on a useful sub-task. By analogy, our clustering method might be extended to process datasets that contain discrete data objects embedded in diffuse data objects. An example might be content description of landscape images, where some of the image content is discrete (e.g. people and animals) and some is diffuse (sky and cloud). Our method could certainly be adapted to audio recordings of other soundscapes (natural or artificial) containing complex combinations of discrete and continuous acoustic events.

## Supporting information

S1 File Site information. Site photographs and a list of bird species found at each site.
(PDF)

S2 File Gympie acoustic indices. The summary acoustic indices calculated on one-minute segments of the Gympie National Park recording.
(CSV)

S3 File Woondum acoustic indices. The summary acoustic indices calculated on one-minute segments of the Woondum National Park recording.
(CSV)

S4 File Sample minutes. Links to the 1200 minutes used to help determine the cluster contents.
(PDF)

S5 File Cluster statistics. Complete sets of the temporal distribution plots, radar plots, polar histograms for each of the 60 clusters.
(PDF)
