# Supplementary material for: Revealing the ecological content of long-duration audio-recordings of the environment through clustering and visualisation
Source: PLoS One. 2018 Mar 1;13(3):e0193345. doi: 10.1371/journal.pone.0193345 (PMC5832236; doi:10.1371/journal.pone.0193345)
Supplement: S1 File — Site photographs and a list of bird species found at each site. (PDF) [file pone.0193345.s001.pdf]

S1 Site Information

The following pages contain two sections:

Section A: Site photographs of the two sites (photographs taken by Y. Phillips).

Section B: Species List – listing of bird species which have been annotated in the recordings at each site.

Section A: Site details

Contents: Site details includes photographs of the site.

Notes:

1. Gympie is 160 km north of Brisbane in Queensland, Australia.
2. Gympie National Park (gazetted in 2006) previously a State Forest and Forest Reserve has an area of 1768 hectare [1]. The recording site within the National Park has an elevation of 225 m and a distance of 45 km from the coastline.
3. Woondum National Park (gazetted in 2009) approximately 20 km south-east of Gympie was previously a State Forest and Forest Reserve has an area of 4001 hectares [2]. The recording site has an elevation of 118 m and is 28 km from the coastline.

Photos from Gympie National Park https://doi.org/10.4225/09/5a7ce77feb9e9

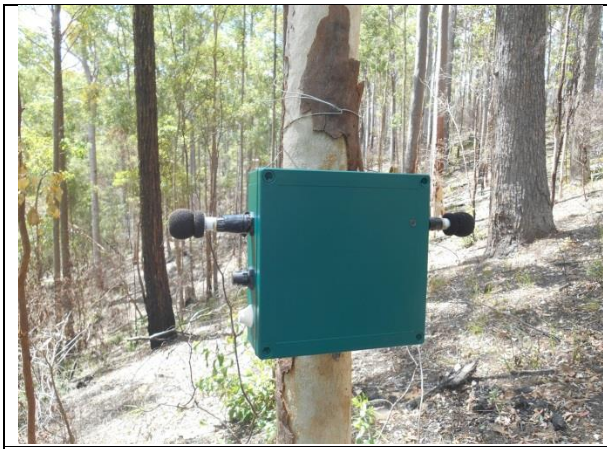

SM2+ recorder in situ

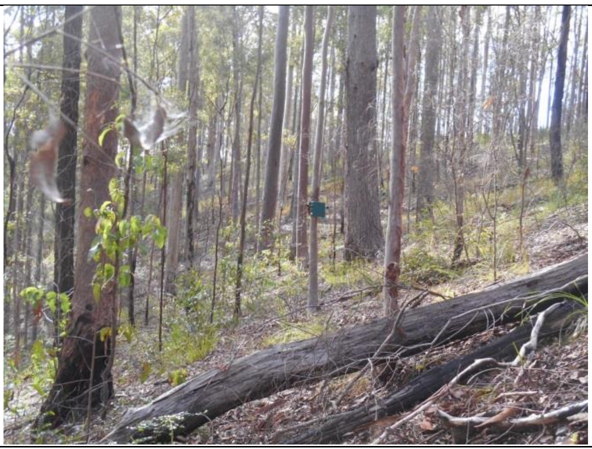

Vegetation structure

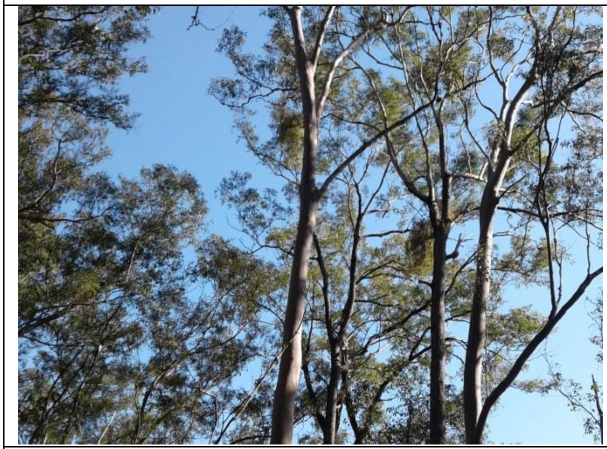

Tree Canopy with Mistletoe spp.

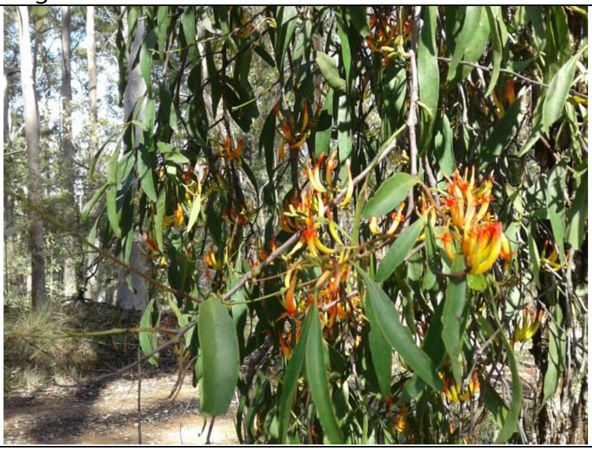

Mistletoe species

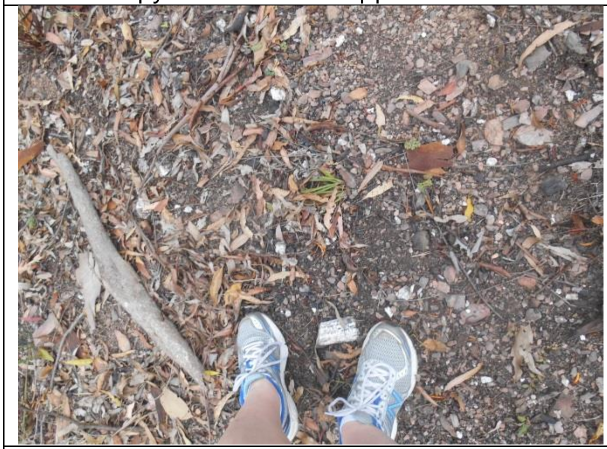

Groundcover

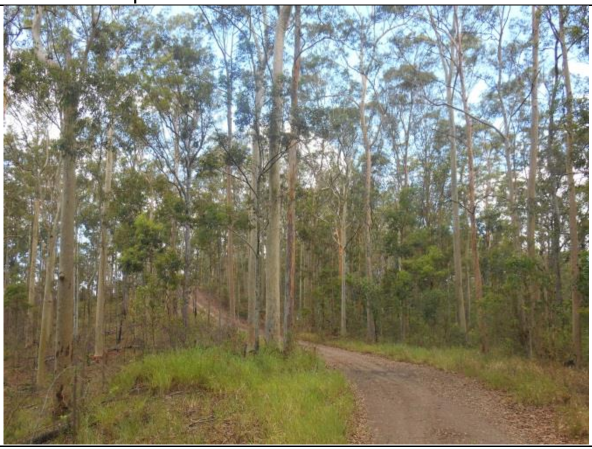

Vehicle access

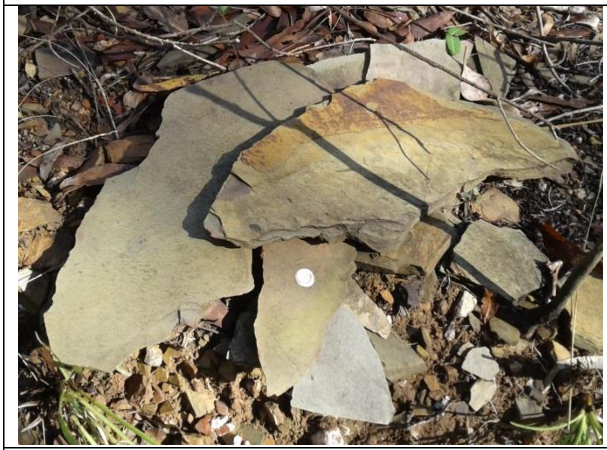

Geology – Sedimentary (metamorphosed)

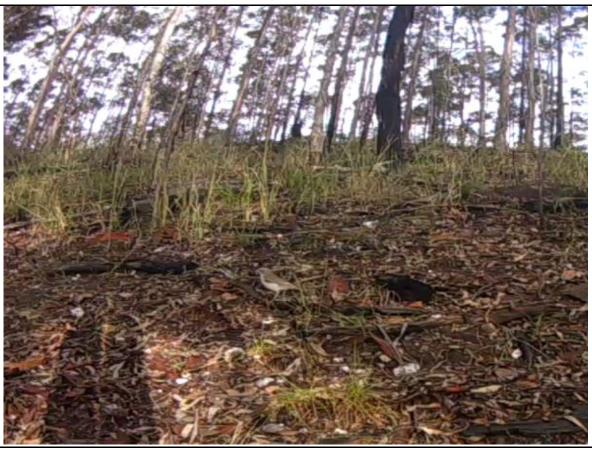

Grey shrike-thrush captured on a wildlife camera

Photographs from Woondum National Park https://doi.org/10.4225/09/5a7ce77feb9e9

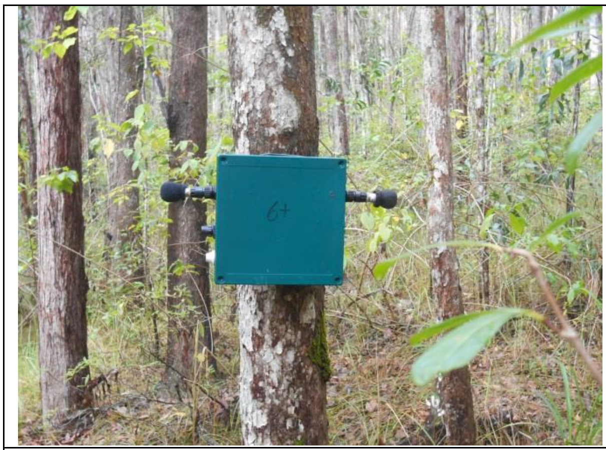

SM2+ recorder in situ

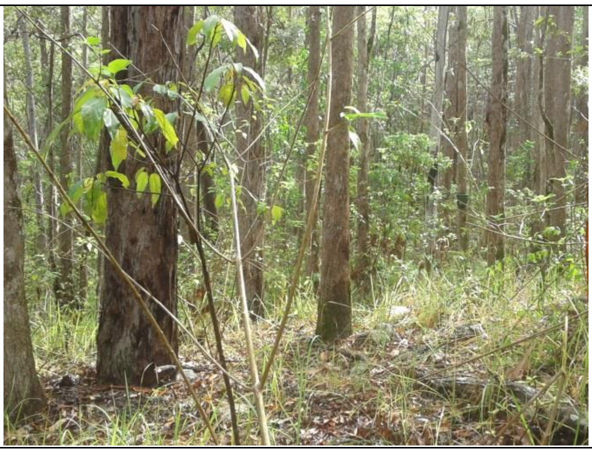
Vegetation structure

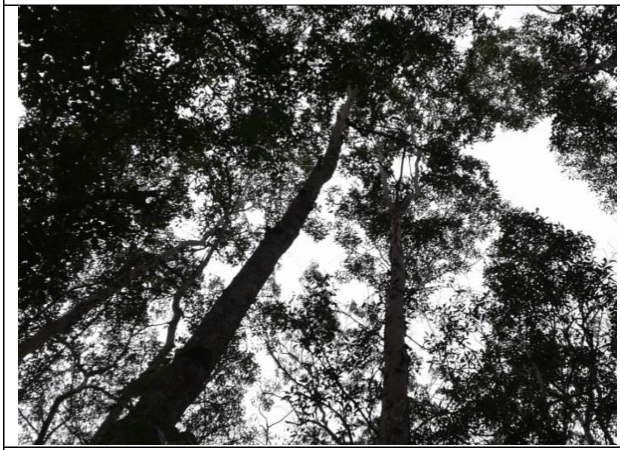
Tree canopy

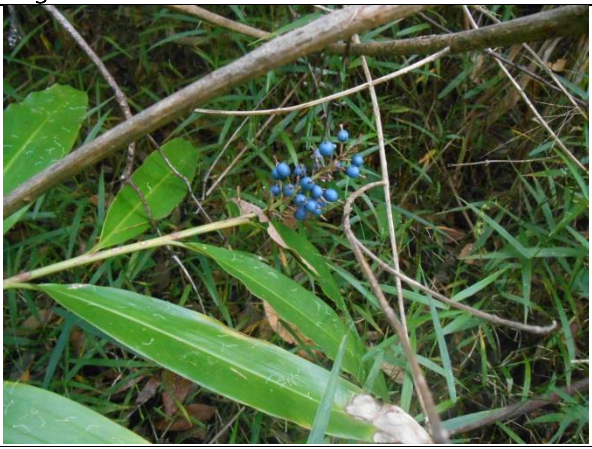
Native ginger (*Alpinia caerulea*)

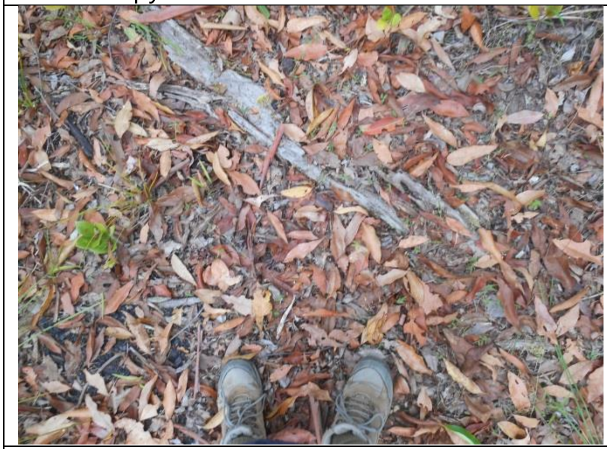
Groundcover

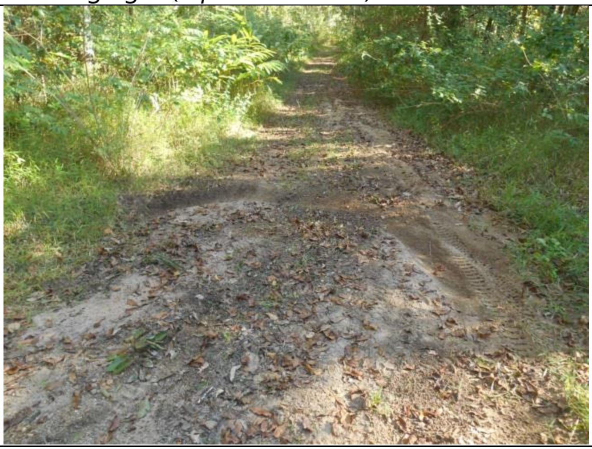
No vehicle access is permitted.

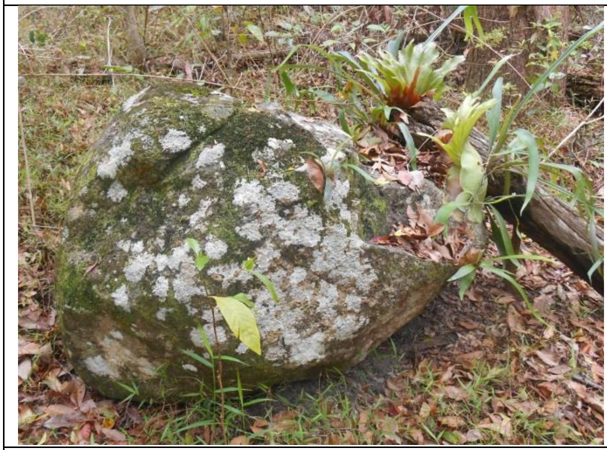
Geology – Granite

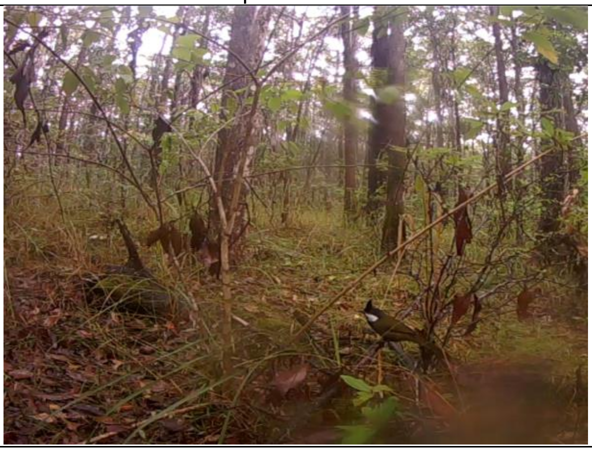
Eastern Whipbird captured on a wildlife camera

Section B: Bird Species

Bird species at Gympie and Woondum National Park sites by Order and Family

Order	Family [3]	Species Names (Gympie NP)	N	Species Names (Woondum NP)	N	Number in common	TOTAL
Caprimulgiformes	Aegothelidae	Australian Owlet-Nightjar	1	Australian Owlet-Nightjar	1	1	1
Caprimulgiformes	Caprimulgidae	White-throated Nightjar	1		0	0	1
Caprimulgiformes	Podargidae	Tawny Frogmouth	1		0	0	1
Columbiformes	Columbidae	Brown Cuckoo-Dove<br>Peaceful Dove<br>Wonga Pigeon<br>Topknot Pigeon ⊙<br>Bar-shouldered Dove ⊙<br>Emerald Dove ⊙<br>White-headed Pigeon	7	Brown Cuckoo-Dove<br>Peaceful Dove<br>Wonga Pigeon<br>Woompoo Fruit Dove<br>White-headed Pigeon<br>Superb Fruit-Dove	6	4	9
Coraciiformes	Halcyonidae	Laughing Kookaburra	1	Laughing Kookaburra	1	1	1
Coraciiformes	Meropidae	Rainbow Bee-eater	1	Rainbow Bee-eater	1	1	1
Cuculiformes	Cuculidae	Fan-tailed Cuckoo<br>Brush Cuckoo<br>Shining Bronze Cuckoo<br>Channel-billed Cuckoo	4	Fan-tailed Cuckoo<br>Brush Cuckoo<br>Shining Bronze Cuckoo<br>Channel-billed Cuckoo<br>Eastern Koel<br>Little Bronze-Cuckoo	6	4	6
Galliformes	Megapodiidae	Australian Brush Turkey	1	Australian Brush Turkey	1	1	1
Passeriformes	Acanthizidae	White-browed Scrubwren<br>Large-billed Scrubwren<br>Brown Gerygone<br>White-throated Gerygone	4	Large-billed Scrubwren<br>Brown Gerygone<br>Brown Thornbill	3	3	4
Passeriformes	Artamidae	Pied Butcherbird<br>Grey Butcherbird<br>Pied Currawong<br>Australian Magpie	4	Pied Currawong<br>Australian Magpie	2	2	4

Order	Family [3]	Species Names (Gympie NP)	N	Species Names (Woondum NP)	N	Number in common	TOTAL
Passeriformes	Campephagidae	Black-faced Cuckoo-shrike<br>White-bellied Cuckoo-shrike<br>Cicadabird<br>Varied Triller	4	Cicadabird<br>Varied Triller	2	2	4
Passeriformes	Cinclosomatidae	Eastern Whipbird	1	Eastern Whipbird	1	1	1
Passeriformes	Climacteridae	White-throated Treecreeper	1	White-throated Treecreeper<br>Red-browed Treecreeper	2	1	2
Passeriformes	Corcoracidae	White-winged Chough	1		0	0	1
Passeriformes	Corvidae	Torresian Crow	1	Torresian Crow	1	1	1
Passeriformes	Dicaeidae	Mistletoebird	1	Mistletoebird	1	1	1
Passeriformes	Dicruridae	Spangled Drongo<br>Spectacled Monarch<br>Black-faced Monarch<br>Leaden Flycatcher<br>Magpie-Lark<br>Grey Fantail<br>Rufous Fantail<br>Willie-wagtail	8	Spangled Drongo<br>Spectacled Monarch<br>Leaden Flycatcher<br>Grey Fantail<br>Rufous Fantail	5	5	8
Passeriformes	Estrildidae		0	Red-browed Finch	1	0	1
Passeriformes	Maluridae	Variegated Fairy-wren	1	Variegated Fairy-wren	1	1	1
Passeriformes	Meliphagidae	Scarlet Honeyeater<br>White-throated Honeyeater<br>Lewin's Honeyeater<br>Noisy Miner<br>Eastern Spinebill<br>Noisy Friarbird<br>Yellow-faced Honeyeater	7	Scarlet Honeyeater<br>White-throated Honeyeater<br>Lewin's Honeyeater<br>Yellow-faced Honeyeater<br>Noisy Friarbird	5	5	7
Passeriformes	Oriolidae		0	Olive-backed Oriole<br>Australian Figbird	2	0	2
Passeriformes	Orthonychidae		0	Australian Logrunner	1	0	1

Order	Family [3]	Species Names (Gympie NP)	N	Species Names (Woondum NP)	N	Number in common	TOTAL
Passeriformes	Pachycephalidae	Rufous Whistler<br>Golden Whistler<br>Grey Shrike-thrush<br>Eastern Shrike-tit	4	Rufous Whistler<br>Golden Whistler<br>Grey Shrike-thrush<br>Little Shrike-thrush	4	3	5
Passeriformes	Pardalotidae	Spotted Pardalote<br>Striated Pardalote	2		0	0	2
Passeriformes	Petroicidae	Eastern Yellow Robin<br>Rose Robin	2	Eastern Yellow Robin	1	1	2
Passeriformes	Pittidae	Noisy Pitta	1	Noisy Pitta	1	1	1
Passeriformes	Ptilonorhynchidae	-	0	Green Catbird	1	0	1
Passeriformes	Zosteropidae		0	Silvereye	1		1
Psittaciformes	Psittacidae	Scaly-breasted Lorikeet<br>Rainbow Lorikeet<br>Little Lorikeet<br>Australian King Parrot<br>Pale-headed Rosella	5	Rainbow Lorikeet<br>Little Lorikeet<br>Australian King Parrot<br>Crimson Rosella	4	4	5
Strigiformes	Strigidae	Southern Boobook<br>Powerful Owl	2	Southern Boobook	1	1	2
Strigiformes	Tytonidae	-	0	Sooty Owl	1	0	1
TOTALS	30	**GYMPIE NP TOTAL**	**66**	**WOONDUM NP TOTAL**	**57**	**44**	**79**

⊙ Birds seen at site but not yet found (annotated) in the recording

References

1. Queensland Government. Gympie National Park Management Statement2013; (30 December 2012). Available from: http://www.npsr.qld.gov.au/managing/plans-strategies/statements/pdf/gympie.pdf.
2. Queensland Government. Woondum National Park Management Statement2013. Available from: http://www.npsr.qld.gov.au/managing/plans-strategies/statements/pdf/woondum.pdf.
3. Simpson K, Day N. Field Guide to the Birds of Australia. Camberwell, Australia: Penguine Group; 2004.
