# Supplementary material for: Revealing the ecological content of long-duration audio-recordings of the environment through clustering and visualisation
Source: PLoS One. 2018 Mar 1;13(3):e0193345. doi: 10.1371/journal.pone.0193345 (PMC5832236; doi:10.1371/journal.pone.0193345)
Supplement: S4 File — Links to the 1200 minutes used to help determine the cluster contents. (PDF) [file pone.0193345.s004.pdf]

S2 Sampled minutes

This file describes the contents of the 1200 minutes, 20 randomly sampled minutes, 10 mintues from each site from each of the 60 clusters (refered to in the PLoS ONE article) used to determine the contents of each cluster. The hyperlinks display the sampled minute in 30-second segments on the Ecosounds website [1] using the Acoustic Workbench software [2].

Ecosounds (www.ecosounds.org) is a website that facilitates the management, access, visualization, and analysis of environmental acoustic data. This software, named the Acoustic Workbench, is open source and available from GitHub (https://github.com/QutBioacoustics/). The website is run by the QUT Ecoacoustics Research Group to support bioacoustics and ecoacoustics research. Registration can be made through: https://www.ecosounds.org/my_account/sign_up

To reference the audio data accessed through the hyperlinks in this document: Phillips, Y. (2016) *The Cooloola Project* [Continuous Acoustic Recordings]. Gympie, Australia. Retrieved March 17, 2017, available from Ecosounds: https://www.ecosounds.org/projects/1029

Cluster #	Brief description	Cluster #	Brief description	Cluster #	Brief description
1	INSECTS	21	LIGHT RAIN	41	VERY QUIET
2	LIGHT RAIN AND BIRDS	22	INSECTS AND BIRDS	42	STRONG WIND
3	BIRDS (LOW RATE OF CALLING)	23	PLANES, MOTORBIKES, THUNDER	43	BIRDS - MORNING CHORUS
4	INSECTS AND BIRDS	24	WIND AND/OR CICADAS[IC]	44	LOUD CICADAS
5	FAIRLY QUIET	25	WIND	45	WIND AND PLANES
6	MOSTLY QUIET	26	INSECTS AND WIND	46	WIND
7	CICADAS AND BIRDS AND WIND	27	INSECTS	47	VERY STRONG WIND
8	CICADAS AND BIRDS AND WIND	28	BIRDS AND/OR INSECTS OR PLANES[IC]	48	CICADAS
9	MODERATE WIND	29	INSECTS	49	PLANES (INCLUDING THUNDER)
10	LIGHT TO MODERATE RAIN	30	BIRDS AND QUIET	50	QUIET AND INSECTS AND BIRDS[IC]
11	BIRDS	31	QUIET	51	STRONG WIND
12	CICADAS	32	CICADAS	52	WIND
13	QUIET	33	BIRDS	53	MOSTLY QUIET
14	BIRDS	34	CICADAS	54	MODERATE RAIN AND BIRDS
15	BIRDS	35	QUIET	55	QUIET
16	CICADAS	36	QUIET AND/OR PLANES[IC]	56	WIND
17	LIGHT RAIN OR INSECTS[IC]	37	BIRDS - MORNING CHORUS	57	BIRDS OR WIND[IC]
18	MODERATE RAIN	38	QUIET	58	LOUD BIRDS
19	MODERATE WIND	39	BIRDS AND PLANES	59	MODERATE RAIN
20	MODERATE WIND	40	WIND AND/OR BIRDS OR INSECTS[IC]	60	RAIN/BIRDS

[IC] Inconsistent

Cluster 1 – Gympie National Park

INSECTS (6444 minutes) The dominant sound sources are provided in bold text.

date_ time	Hyperlinks	Insects	Planes	Birds	Rain	Thunder	Wind
20160620_ 011500	https://www.ecosounds.org/listen/354630?start=4485	No	No	No	**Light rain**	No	Moderate wind
20160224_ 201500	https://www.ecosounds.org/listen/353485?start=24200	**Insect between 4.5 and 6 kHz**	No	No	No	No	No
20160224_ 214200	https://www.ecosounds.org/listen/353461?start=5071	**Insect between 4.5 and 6 kHz**	No	No	No	No	No
20151228_ 134100	https://www.ecosounds.org/listen/353815?start=558	**Insects between 6 and 8.5 kHz**	No	Cicadabird and others	No	No	No
20160103_ 091700	https://www.ecosounds.org/listen/353902?start=9068	Insects above 6.5 kHz	**Loud plane**	Pied Currawong, Cicadabird	No	No	No
20160604_ 021700	https://www.ecosounds.org/listen/354536?start=8217	**Insects between 4.8 and 5.5 kHz**	No	No	Very light rain	No	Slight wind
20151112_ 200700	https://www.ecosounds.org/listen/352861?start=23718	**Insects between 4 and 6 kHz**	No	No	No	No	Slight wind
20151109_ 210100	https://www.ecosounds.org/listen/352863?start=2617	**Insects between 4 and 5.2 kHz**	No	No	No	No	Slight to Moderate wind
20151012_ 193400	https://www.ecosounds.org/listen/354704?start=21745	**Insects between 4 and 5.5 kHz and 9.5 kHz**	No	No	No	No	No
20150702_ 071400	https://www.ecosounds.org/listen/269832?start=1684	No	No	**Lewins honeyeater, Torresian Crow, Pied Currawong, Scarlet Honeyeater, Rufous Whistler, Spotted Pardalote**	No	No	No

Cluster 1 – Woondum National Park

INSECTS (3249 minutes) The dominant sound sources are provided in bold text.

date_ time	Hyperlinks	Insects	Planes	Birds	Rain	Thunder	Wind
20160228_ 185900	https://www.ecosounds.org/listen/353961?start=20214	**Insects at 2 kHz and between 4.5 and 7.5 kHz**	No	No	No	No	Slight wind
20160206_ 004500	https://www.ecosounds.org/listen/353354?start=2696	**Insects between 4.5 and 7 kHz**	No	No	No	No	Slight to moderate wind
20160316_ 223200	https://www.ecosounds.org/listen/353611?start=8065	**Insects between 4.8 and 7 kHz**	No	No	No	No	Moderate wind
20160107_ 042900	https://www.ecosounds.org/listen/353954?start=16136	**Insects between 2.8 and 5 kHz**	No	Yellow-faced Honeyeater	No	No	Slight wind
20151106_ 185200	https://www.ecosounds.org/listen/352812?start=19215	**Insect at 0.9 kHz and between 4 and 7 kHz**	No	No	No	No	Moderate wind
20151106_ 201500	https://www.ecosounds.org/listen/352812?start=24195	**Insects between 5 and 7.2 kHz**	**Loud plane**	No	No	No	No
20160504_ 174800	https://www.ecosounds.org/listen/354300?start=15375	**Insects between 2.6 and 3.1 and between 5 and 6.5 kHz**	Moderately loud plane	No	No	No	No
20151124_ 152100	https://www.ecosounds.org/listen/352969?start=6558	No	Moderately loud plane	**Eastern Yellow Robin piping**	Very light rain	No	Moderate wind
20151202_ 202200	https://www.ecosounds.org/listen/353025?start=261	**Insects between 4.5 and 6.5 kHz**	**Loud plane**	No	No	No	No
20151031_ 065000	https://www.ecosounds.org/listen/352780?start=245	No	No	**Yellow-faced Honeyeater, Mistletoebird , Eastern Whipbird**	No	No	Moderate wind

Cluster 2 – Gympie National Park

LIGHT RAIN AND BIRDS (7091 minutes) The dominant sound sources are provided in bold text.

date_ time	Hyperlinks	Insects	Planes	Birds	Rain	Thunder	Wind
20160309_ 193000	https://www.ecosounds.org/listen/353517?start=21499	Insect between 4.9 and 5.5 kHz	No	No	**Light rain**	No	No
20150930_ 055300	https://www.ecosounds.org/listen/333279?start=21175	No	No	**Lewin's Flycatcher, Leaden flycatcher, Eastern Whipbird, Mistletoebird, Brown Cuckoo-dove, White-throated Honeyeater, White-throated Treecreeper**	No	No	Moderat e wind
20150821_ 201600	https://www.ecosounds.org/listen/331658?start=24262	**Insect between 3.4 and 4.4 kHz**	No	No	**Light to moderate rain**	No	No
20151130_ 062500	https://www.ecosounds.org/listen/352988?start=23097	Insects above 6 kHz	**Loud plane**	**Rufous Whistler, Eastern Whipbird, White-throated Treecreeper, Crested Shrike-tit**	No	No	No
20151116_ 055900	https://www.ecosounds.org/listen/352898?start=21538	No	No	**Lewin's Honeyeater, White-throated Treecreeper, Rufous Whistler**	No	No	Moderat e wind
20160429_ 035200	https://www.ecosounds.org/listen/354245?start=13911	No	No	No	**Light rain**	No	No
20160615_ 034500	https://www.ecosounds.org/listen/354634?start=13497	No	No	No	**Light rain**	No	Slight wind
20160130_ 033100	https://www.ecosounds.org/listen/353276?start=12656	**Insects between 4 and 6 kHz**	No	No	**Light rain**	No	Moderat e wind
20160604_ 102600	https://www.ecosounds.org/listen/354535?start=13207	No	No	Spotted Pardalote, Yellow-faced Honeyeater	No	No	**Modera te to strong wind**
20160716_ 071000	https://www.ecosounds.org/listen/354933?start=1445	No	No	White-throated Honeyeater, Torresian Crow	**Light to moderate rain**	No	No

Cluster 2 – Woondum National Park

LIGHT RAIN AND BIRDS (9600 minutes) The dominant sound sources are provided in bold text.

date_ time	Hyperlinks	Insects	Planes	Birds	Rain	Thunder	Wind
20150926_ 084300	https://www.ecosounds.org/listen/333264?start=7027	No	No	**Yellow-faced Honeyeater, Rufous Whistler, Scarlet Honeyeater**	No	No	No
20150821_ 132000	https://www.ecosounds.org/listen/331661?start=23650	No	No	**Scarlet Honeyeater, Yellow-faced Honeyeater, Golden Whistler**	**Light rain**	No	No
20151115_ 141200	https://www.ecosounds.org/listen/352927?start=7155	No	No	**Scarlet Honeyeater**	No	No	Slight wind
20151213_ 080500	https://www.ecosounds.org/listen/353084?start=4743	Insects between 6 and 9 kHz	No	**Grey Shrike-thrush, Eastern Whipbird**	**Light rain**	No	No
20151003_ 074500	https://www.ecosounds.org/listen/333322?start=3545	No	No	**Eastern Whipbird, Yellow-faced Honeyeater, Rufous Whistler, Scarlet Honeyeater**	No	No	No
20160307_ 085400	https://www.ecosounds.org/listen/353559?start=7689	No	No	Pied Currawong	**Light rain**	No	No
20151031_ 161100	https://www.ecosounds.org/listen/352781?start=9553	No	No	**Golden Whistler, Scarlet Honeyeater**	No	No	Moderate wind
20160316_ 083800	https://www.ecosounds.org/listen/353599?start=6726	No	No	**Eastern Whipbird, Brown Cuckoo dove**	**Light rain**	No	Slight wind
20151213_ 083200	https://www.ecosounds.org/listen/353084?start=6363	**Insects between 4 and 10 kHz (cicada**)	No	**Grey Shrike-thrush, others**	No	No	No
20150629_ 143000	https://www.ecosounds.org/listen/269842?start=3504	No	**Yes**	Scarlet Honeyeater, Pied Currawong	**Light to moderate rain**	No	Slight to moderate wind

Cluster 3 – Gympie National Park

BIRDS (Low rate) (16020 minutes) The dominant sound sources are provided in bold text.

date_ time	Hyperlinks	Insects	Planes	Birds	Rain	Thunder	Wind
20160109_ 060300	https://www.ecosounds.org/listen/353994?start=21776	No	No	**Eastern Koel, Fan-tailed Cuckoo**	No	No	No
20150702_ 064000	https://www.ecosounds.org/listen/269817?start=23995	No	No	**Scarlet Honeyeater, White-throated Treecreeper, Eastern Whipbird, Lewin's Honeyeater**	No	No	**Moderate wind**
20160705_ 071900	https://www.ecosounds.org/listen/354860?start=1969	No	No	**White-throated Honeyeater, Eastern Whipbird, Pied Currawong, Spotted Pardalote**	No	No	**Moderate wind**
20160519_ 154900	https://www.ecosounds.org/listen/354364?start=8242	Insects between 7 and 8 kHz	Distant plane	**Lewin's Honeyeater, Yellow-faced Honeyeater**	No	No	Slight wind
20160322_ 071700	https://www.ecosounds.org/listen/353648?start=1868	No	No	**Torresian Crow, Eastern Whipbird**	No	No	Slight wind
20151112_ 102600	https://www.ecosounds.org/listen/352860?start=13208	Insects between 5.5 and 10 kHz	No	**Grey Shrike-thrush, White-throated Treecreeper, Mistletoebird**	No	No	Slight wind
20160504_ 080500	https://www.ecosounds.org/listen/354244?start=4749	No	Distant plane	**Spotted Pardalote, Lewin's Honeyeater, Scarlet Honeyeater**	No	No	Slight wind
20160501_ 092900	https://www.ecosounds.org/listen/354233?start=9784	No	Distant plane	**Rose Robin, Grey Fantail, White-throated Honeyeater**	No	No	Slight wind
20160603_ 090600	https://www.ecosounds.org/listen/354508?start=8410	No	No	**Eastern Whipbird, Grey Fantail**	No	No	**Moderate wind**
20150818_ 135100	https://www.ecosounds.org/listen/331620?start=1160	No	No	**Scarlet Honeyeater, Mistletoebird**	No	No	Slight wind

Cluster 3 – Woondum National Park

BIRDS (Low rate) (9167 minutes) The dominant sound sources are provided in bold text.

date_ time	Hyperlinks	Insects	Planes	Birds	Rain	Thunder	Wind
20151020_ 075100	https://www.ecosounds.org/listen/354783?start=3906	Insects between 5.5 and 9 kHz	No	**Golden Whistler**	No	No	Slight wind
20151222_ 051700	https://www.ecosounds.org/listen/353831?start=19018	No	No	**Olive-backed Oriole, Channel-billed Cuckoo, White-throated Treecreeper, Spangled Drongo**	No	No	Slight wind
20151018_ 091300	https://www.ecosounds.org/listen/354771?start=8824	**Insects between 6 and 9 kHz**	No	**Golden Whistler, Yellow-faced Honeyeater, Scarlet Honeyeater**	No	No	No
20151101_ 152600	https://www.ecosounds.org/listen/352813?start=4141	Insects between 6 and 9 kHz	Moderately loud plane	**Golden Whistler, Scarlet Honeyeater, White-throated Treecreeper**	No	No	**Moderate wind**
20150725_ 150100	https://www.ecosounds.org/listen/277106?start=5361	No	No	**Scarlet Honeyeater**	No	No	Moderate wind
20160228_ 000100	https://www.ecosounds.org/listen/353504?start=56	**Insects between 4.8 and 8 kHz**	No	No	No	No	No
20160705_ 150900	https://www.ecosounds.org/listen/354915?start=5805	No	No	**Scarlet Honeyeater**	No	No	**Moderate wind**
20160506_ 073500	https://www.ecosounds.org/listen/354282?start=2945	No	No	**Australasian Figbird, White-throated Treecreeper, Spotted Pardalole**	No	No	Moderate wind
20160407_ 112700	https://www.ecosounds.org/listen/353796?start=16863	No	No	**Golden Whistler, Grey Fantail**	No	No	Moderate wind
20160405_ 071800	https://www.ecosounds.org/listen/353786?start=1927	No	**Moderately loud plane**	**Brown Cuckoo-dove, White-throated Treecreeper**	No	No	No

Cluster 4 – Gympie National Park

INSECTS AND BIRDS (17521 minutes) The dominant sound sources are provided in bold text.

date_ time	Hyperlinks	Insects	Planes	Birds	Rain	Thunder	Wind
20160117_ 161200	https://www.ecosounds.org/listen/353162?start=9621	**Insects above 8 kHz**	No	Laughing Kookaburra,	No	No	Moderate wind
20151012_ 163000	https://www.ecosounds.org/listen/354704?start=10705	**Insects above 5 and 7 kHz**	Distant plane	**Lewin's honeyeater**	No	No	Moderate wind
20160325_ 191300	https://www.ecosounds.org/listen/353636?start=20482	**Insects between 3 and 6 kHz**	No	No	No	No	No
20150726_ 095000	https://www.ecosounds.org/listen/277079?start=11048	No	No	Lewin's Honeyeater, Pied Butcherbird, Scarlet Honeyeater	No	No	**Moderate wind**
20160103_ 164400	https://www.ecosounds.org/listen/353880?start=11541	**Insects between 5 and 8 kHz**	Distant plane	**White-throated Honeyeater**	No	No	No
20150719_ 085500	https://www.ecosounds.org/listen/277037?start=7745	No	Distant plane	**Eastern Whipbird, Torresian Crow, Noisy Friarbird, Spotted Pardalote**	No	No	No
20160704_ 165500	https://www.ecosounds.org/listen/354856?start=12164	No	No	Laughing Kookaburra	No	No	No
20151118_ 160200	https://www.ecosounds.org/listen/352885?start=9019	**Insects above 5 kHz**	Distant plane	Torresian Crow	No	No	No
20160206_ 162700	https://www.ecosounds.org/listen/353329?start=10516	No	No	**White-throated Honeyeater, Lewin's Honeyeater**	No	No	**Moderate wind**
20151128_ 193300	https://www.ecosounds.org/listen/352954?start=21675	**Insects between 4 and 6.5 and above 8 kHz**	No	Unknown	No	No	No

Cluster 4 – Woondum National Park

INSECTS AND BIRDS (10813 minutes) The dominant sound sources are provided in bold text.

date_ time	Hyperlinks	Insects	Planes	Birds	Rain	Thunder	Wind
20160502_ 095900	https://www.ecosounds.org/listen/354266?start=11590	No	**Moderately loud plane**	White-throated Treecreeper, Grey Fantail	No	No	Slight to moderate wind
20150702_ 104500	https://www.ecosounds.org/listen/269851?start=14346	No	No	**Grey Fantail, Lewin's Honeyeater**	No	No	Moderate wind
20151209_ 012500	https://www.ecosounds.org/listen/353075?start=5095	**Insects between 5 and 7.5 kHz**	Distant plane	Yes	No	No	Slight to moderate wind
20151105_ 014400	https://www.ecosounds.org/listen/352803?start=6237	**Insects between 5 and 7 kHz**	Distant plane	No	No	No	No
20150919_ 112500	https://www.ecosounds.org/listen/331904?start=16743	No	No	Eastern Yellow Robin piping	No	No	Slight wind
20151115_ 082600	https://www.ecosounds.org/listen/352845?start=6003	**Insects above 5.5 kHz**	No	**Mistletoebird, White-throated Treecreeper**	No	No	Moderate wind
20151212_ 055200	https://www.ecosounds.org/listen/353089?start=21118	No	Moderately loud plane	**Pied Currawong, Scarlet Honeyeater**	No	No	Slight wind
20160202_ 204400	https://www.ecosounds.org/listen/353312?start=1588	**Insects between 4 and 7 kHz**	No	No	No	No	No
20151006_ 181200	https://www.ecosounds.org/listen/333363?start=16823	**Insects between 1.9 and 2.1 and 4 and between 5 and 6 kHz**	Moderately loud plane	Russet-tailed Thrush	No	No	No
20160202_ 171900	https://www.ecosounds.org/listen/353314?start=13637	**Insects between 7 and 10 kHz**	No	Yes	No	No	Moderate wind

Cluster 5 – Gympie National Park

FAIRLY QUIET (27053 minutes) The dominant sound sources are provided in bold text.

date_ time	Hyperlinks	Insects	Planes	Birds	Rain	Thunder	Wind
20160722_ 041000	https://www.ecosounds.org/listen/355008?start=14990	Very quiet insects between 4 to 5 kHz	No	No	No	No	Slight wind
20150927_ 223700	https://www.ecosounds.org/listen/333268?start=10061	No	No	No	No	No	Moderate wind
20150928_ 032000	https://www.ecosounds.org/listen/333270?start=11998	No	No	No	No	No	Moderate wind
20151010_ 222300	https://www.ecosounds.org/listen/333348?start=7534	**Insects (quiet) between 4.5 and 5 kHz**	No	No	No	No	No
20150922_ 003000	https://www.ecosounds.org/listen/333219?start=1798	Quiet insects between 4 and 5 kHz	No	No	No	No	Moderate wind
20160420_ 051200	https://www.ecosounds.org/listen/354149?start=18717	Very quiet insects between 4 to 5 kHz	No	No	No	No	Slight to moderate wind
20160623_ 000100	https://www.ecosounds.org/listen/354667?start=50	No	No	No	No	No	No
20160419_ 230500	https://www.ecosounds.org/listen/354125?start=10053	**Insects (quiet) between 4 and 6 kHz**	No	No	No	No	No
20160102_ 023200	https://www.ecosounds.org/listen/353873?start=9116	Very quiet insects between 4.9 and 5.1 kHz	No	No	No	No	No
20150829_ 000300	https://www.ecosounds.org/listen/331773?start=177	No	No	No	No	No	Moderate wind

Cluster 5 – Woondum National Park

FAIRLY QUIET (8942 minutes) The dominant sound sources are provided in bold text.

date_ time	Hyperlinks	Insects	Planes	Birds	Rain	Thunder	Wind
20160525_ 044300	https://www.ecosounds.org/listen/354440?start=16972	No	Moderately loud plane	No	No	No	No
20150926_ 025100	https://www.ecosounds.org/listen/333262?start=10257	No	No	No	No	No	No
20151019_ 205200	https://www.ecosounds.org/listen/354780?start=2078	**Quiet crickets between 4.5 and 5 kHz**	No	No	No	No	No
20160611_ 234200	https://www.ecosounds.org/listen/354540?start=12226	No	Moderately loud plane	No	No	No	No
20160524_ 221600	https://www.ecosounds.org/listen/354451?start=7067	Distant insects between 4.5 and 5 kHz	No	No	No	No	No
20150908_ 214000	https://www.ecosounds.org/listen/331839?start=4943	Quiet insects between 4.5 and 5 kHz	No	No	No	No	No
20151019_ 210700	https://www.ecosounds.org/listen/354780?start=2978	**Quiet insects between 4.5 and 5 kHz**	Distant plane	No	No	No	No
20150814_ 022000	https://www.ecosounds.org/listen/331545?start=8396	No	**Distant plane**	No	No	No	Moderate wind
20160531_ 022200	https://www.ecosounds.org/listen/354465?start=8518	No	No	No	No	No	No
20150728_ 043500	https://www.ecosounds.org/listen/277144?start=16496	No	No	Southern Bookbook	No	No	Moderate wind

Cluster 6 – Gympie National Park

MOSTLY QUIET (2883 minutes) The dominant sound sources are provided in bold text.

date_ time	Hyperlinks	Insects	Planes	Birds	Rain	Thunder	Wind
20150726_ 175200	https://www.ecosounds.org/listen/277172?start=15914	No	No	No	No	No	No
20160115_ 152100	https://www.ecosounds.org/listen/354023?start=6556	No	Distant plane	Distant birds	No	No	No
20150625_ 043300	https://www.ecosounds.org/listen/331567?start=16375	No	No	No	Light rain	No	No
20160513_ 013500	https://www.ecosounds.org/listen/354357?start=5691	No	No	No	No	No	No
20150713_ 220300	https://www.ecosounds.org/listen/276994?start=6333	No	No	No	No	No	No
20160529_ 023100	https://www.ecosounds.org/listen/354453?start=9049	No	No	No	No	No	No
20160611_ 044100	https://www.ecosounds.org/listen/354570?start=16850	No	Distant plane	No	No	No	No
20160531_ 030100	https://www.ecosounds.org/listen/354517?start=10857	No	Distant plane	No	No	No	No
20160614_ 190200	https://www.ecosounds.org/listen/354606?start=19819	No	No	No	Light rain	No	No
20160629_ 050000	https://www.ecosounds.org/listen/354809?start=17996	No	No	No	No	No	No

Cluster 6 – Woondum National Park

MOSTLY QUIET (1640 minutes) The dominant sound sources are provided in bold text.

date_ time	Hyperlinks	Insects	Planes	Birds	Rain	Thunder	Wind
20160119_ 174700	https://www.ecosounds.org/listen/353203?start=15312	Distant insects between 6 and 9 kHz	No	Distant birds	No	No	Moderate wind
20151012_ 160500	https://www.ecosounds.org/listen/354725?start=9199	Distant cicadas above 4.5 kHz	No	Distant birds	No	No	No
20160323_ 142800	https://www.ecosounds.org/listen/353680?start=3373	No	Distant plane	Distant birds	No	No	Moderate wind
20151124_ 135800	https://www.ecosounds.org/listen/352969?start=1578	No	No	Distant birds	No	No	No
20150828_ 025000	https://www.ecosounds.org/listen/331797?start=10196	No	Moderately loud planes	No	Very light	No	Moderate wind
20160628_ 185700	https://www.ecosounds.org/listen/354831?start=19517	No	No	No	No	No	No
20160305_ 023900	https://www.ecosounds.org/listen/353981?start=9537	Distant insects between 4.8 and 6 kHz	No	No	No	No	No
20160513_ 162000	https://www.ecosounds.org/listen/354309?start=10074	No	Distant plane	High frequency birds	No	No	No
20160327_ 133700	https://www.ecosounds.org/listen/353699?start=137	No	No	Lewin's Honeyeater + Animal movements	No	No	No
20160703_ 121900	https://www.ecosounds.org/listen/354907?start=8588	No	Distant plane	High frequency birds	No	No	No

Cluster 7 – Gympie National Park

CICADAS AND BIRDS AND WIND (10702 minutes) The dominant sound sources are provided in bold text.

date_ time	Hyperlinks	Insects	Planes	Birds	Rain	Thunder	Wind
20160211_ 131200	https://www.ecosounds.org/listen/353350?start=23166	**Cicadas above 2 kHz**	No	No	No	No	Slight to moderate wind
20160314_ 085400	https://www.ecosounds.org/listen/353563?start=7686	Insects above 6 kHz	Moderately loud plane	**Torresian Crows, Eastern Whipbird, Laughing Kookaburra, Brown Cuckoo-dove, Mistletoebird**	No	No	Slight to moderate wind
20160325_ 074000	https://www.ecosounds.org/listen/353663?start=3249	**Cicadas above 6 kHz**	No	**Torresian Crow, Eastern Whipbird, Cicadabird, Fan-tailed Cuckoo**	No	No	No
20160102_ 135500	https://www.ecosounds.org/listen/353878?start=1397	**Cicadas above 2.8 kHz**	No	No	No	No	No
20160219_ 124700	https://www.ecosounds.org/listen/353438?start=21667	**Cicadas above 4.5 kHz**	No	**Spotted Pardalote, Lewin's Honeyeater**	No	No	No
20151012_ 080500	https://www.ecosounds.org/listen/354720?start=4752	**Cicadas above 6 kHz**	No	Eastern Whipbird, Spectacled Monarch, White-throated Treecreeper	No	No	No
20151201_ 130000	https://www.ecosounds.org/listen/353016?start=22449	**Cicadas above 2 kHz**	No	**Peaceful Dove, Torresian Crow, White-throated Treecreeper**	No	No	No
20150903_ 223800	https://www.ecosounds.org/listen/331707?start=8430	No	No	No	No	No	**Moderate wind**
20160227_ 170700	https://www.ecosounds.org/listen/353498?start=12920	**Cicadas above 6 kHz**	Distant plane	**Torresian Crow, Lewin's Honeyeater, White-throated Treecreeper, White-throated Honeyeater**	No	No	No
20160218_ 153400	https://www.ecosounds.org/listen/353437?start=7342	**Cicadas above 6 kHz**	No	**Grey Shrike-thrush**	No	No	No

Cluster 7 – Woondum National Park

CICADAS AND BIRDS AND WIND (10208 minutes) The dominant sound sources are provided in bold text.

date_ time	Hyperlinks	Insects	Planes	Birds	Rain	Thunder	Wind
20151230_ 124300	https://www.ecosounds.org/listen/353900?start=21431	**Cicadas above 2.8 kHz**	No	Yes	No	No	No
20151021_ 160100	https://www.ecosounds.org/listen/352714?start=8953	No	No	**Golden Whistler**	No	No	**Moderate wind**
20150924_ 154900	https://www.ecosounds.org/listen/333257?start=8235	No	No	No	No	No	**Moderate wind**
20150919_ 163700	https://www.ecosounds.org/listen/331906?start=11111	No	No	**Scarlet Honeyeater**	No	No	**Moderate wind**
20160223_ 175000	https://www.ecosounds.org/listen/353484?start=15495	No	Distant plane	Cicadabird, White-throated Treecreeper	No	No	**Moderate wind**
20160425_ 082700	https://www.ecosounds.org/listen/354206?start=6069	No	No	No	No	No	**Moderate wind**
20150721_ 112700	https://www.ecosounds.org/listen/277110?start=16869	No	Yes	Eastern Whipbird	**Moderate rain**	No	No
20151114_ 180100	https://www.ecosounds.org/listen/352842?start=16157	No	No	Golden Whistler, Rufous Whistler	No	No	**Moderate wind**
20160315_ 122600	https://www.ecosounds.org/listen/353596?start=20407	**Cicadas between 2 and 6 kHz**	No	No	No	No	No
20151018_ 121300	https://www.ecosounds.org/listen/354771?start=19624	**Cicadas between 6 and 9.5 kHz**	No	**Scarlet Honeyeater, Mistletoebird**	No	No	**Moderate wind**

Cluster 8 – Gympie National Park

CICADAS AND BIRDS AND WIND (18210 minutes) The dominant sound sources are provided in bold text.

date_ time	Hyperlinks	Insects	Planes	Birds	Rain	Thunder	Wind
20160317_ 153600	https://www.ecosounds.org/listen/353577?start=7456	**Cicadas above 5.8 kHz**	No	**Wonga Pigeon, Torresian Crow**	No	No	Moderate wind
20151210_ 072400	https://www.ecosounds.org/listen/353111?start=2289	**Cicadas above 5.8 kHz**	**Loud plane**	**Grey Shrike-thrush, Rufous Whistler**	No	No	No
20160425_ 172200	https://www.ecosounds.org/listen/354210?start=13823	No	No	Silvereye	No	No	**Moderate wind**
20160221_ 102800	https://www.ecosounds.org/listen/353466?start=13329	**Cicadas above 6 kHz**	Moderately loud plane	Lewin's Honeyeater	No	No	**Moderate wind**
20151121_ 175800	https://www.ecosounds.org/listen/352899?start=15976	**Cicadas between 7 and 9 kHz**	No	Mistletoebird	No	No	No
20151128_ 084100	https://www.ecosounds.org/listen/352952?start=6906	Cicadas above 6 kHz	Distant plane	**White-throated Treecreeper, Laughing Kookaburra, White-throated Honeyeater, Rufous Whistler, Grey Shrike-thrush**	No	No	**Moderate wind**
20160222_ 030400	https://www.ecosounds.org/listen/353476?start=11038	**Insects between 3.5 and 6 kHz**	No	No	No	No	Slight wind
20150713_ 090600	https://www.ecosounds.org/listen/277035?start=8412	No	Yes	**White-throated Treecreeper, Mistletoebird, Grey Fantail**	No	No	**Moderate wind**
20151230_ 090800	https://www.ecosounds.org/listen/353889?start=8529	No	Yes	**Unknown, White-throated Honeyeater**	No	No	**Moderate wind**
20160213_ 075300	https://www.ecosounds.org/listen/353383?start=4029	**Cicadas above 6 kHz**	Yes	**Eastern Whipbird**	No	No	Moderate wind

Cluster 8 – Woondum National Park

CICADAS AND BIRDS AND WIND (4673 minutes) The dominant sound sources are provided in bold text.

date_ time	Hyperlinks	Insects	Planes	Birds	Rain	Thunder	Wind
20151022_ 172700	https://www.ecosounds.org/listen/354803?start=14118	**Cicadas above 6 kHz**	Loud plane	**Golden Whistler**	No	No	No
20150924_ 140700	https://www.ecosounds.org/listen/333257?start=2115	No	No	Mistletoebird, Scarlet Honeyeater	No	No	**Slight to moderate wind**
20150923_ 155100	https://www.ecosounds.org/listen/333253?start=8354	No	Yes	**Rufous Whistler, Scarlet Honeyeater, Mistletoebird, Golden Whistler**	No	No	Slight to moderate wind
20150812_ 111300	https://www.ecosounds.org/listen/331540?start=16024	No	Yes	**Scarlet Honeyeater**	No	No	**Moderate wind**
20151101_ 180400	https://www.ecosounds.org/listen/352813?start=13621	**Cicadas between 5 and 9 kHz**	Yes	Pied Currawong	No	No	Slight wind
20160103_ 165800	https://www.ecosounds.org/listen/353917?start=12379	**Insects between 6 and 9 kHz**	No	Brown Cuckoo-dove	No	No	Moderate wind
20151124_ 064200	https://www.ecosounds.org/listen/352965?start=24117	**Cicadas above 5 kHz**	**Loud plane**	**White-throated Treecreeper, Cicadabird, Pied Currawong**	No	No	No
20151109_ 110400	https://www.ecosounds.org/listen/352821?start=15491	**Cicadas between 5 and 8.5 kHz**	**Loud plane**	**White-throated Treecreeper, Yellow-faced Honeyeater**	No	No	Moderate wind
20160706_ 052100	https://www.ecosounds.org/listen/354892?start=19248	No	No	No	No	No	**Moderate to strong wind**
20160302_ 144200	https://www.ecosounds.org/listen/353974?start=4221	**Cicadas above 2.5 kHz**	Distant plane	Golden Whistler	No	No	Moderate wind

Cluster 9 – Gympie National Park

MODERATE WIND (21958 minutes) The dominant sound sources are provided in bold text.

date_ time	Hyperlinks	Insects	Planes	Birds	Rain	Thunder	Wind
20150626_ 191500	https://www.ecosounds.org/listen/331563?start=20596	No	No	No	No	No	**Moderate wind**
20150803_ 200200	https://www.ecosounds.org/listen/331487?start=23423	Insects at 2.8 kHz	No	No	No	No	**Moderate wind**
20160712_ 195500	https://www.ecosounds.org/listen/354941?start=23001	Insects at 5 kHz	No	No	No	No	**Slight to moderate wind**
20151013_ 130600	https://www.ecosounds.org/listen/354706?start=22809	**Insects above 6 kHz**	No	Lewin's Honeyeater	No	No	No
20160605_ 150200	https://www.ecosounds.org/listen/354569?start=15340	No	No	No	No	No	**Moderate wind**
20160606_ 003800	https://www.ecosounds.org/listen/354546?start=2268	No	No	No	No	No	**Slight to moderate wind**
20160128_ 114100	https://www.ecosounds.org/listen/353290?start=17706	Insects above 5 KHz	No	Lewin's Honeyeater	No	No	Slight wind
20160216_ 192600	https://www.ecosounds.org/listen/353454?start=21260	**Insects between 4 and 6 kHz**	No	White-throated Nightjar	No	No	No
20150626_ 040600	https://www.ecosounds.org/listen/331572?start=14756	No	No	No	No	No	**Moderate wind**
20151206_ 081800	https://www.ecosounds.org/listen/353026?start=5529	**Insects above 6 kHz**	No	**Eastern Whipbird, Torresian Crow, Cicadabird, White-throated Honeyeater**	No	No	Moderate wind

Cluster 9 – Woondum National Park

MODERATE WIND (27949 minutes) The dominant sound sources are provided in bold text.

date_ time	Hyperlinks	Insects	Planes	Birds	Rain	Thunder	Wind
20160711_003700	https://www.ecosounds.org/listen/354938?start=2220	No	No	No	No	No	**Moderate wind**
20160107_071200	https://www.ecosounds.org/listen/353920?start=1568	**Insects between 5 and 9 kHz**	No	Distant bird	No	No	No
20160621_141800	https://www.ecosounds.org/listen/354666?start=2743	No	No	Distant bird	No	No	**Moderate to stong wind**
20151204_173300	https://www.ecosounds.org/listen/353034?start=14481	**Insects between 5 and 8 kHz**	**Moderately loud plane**	Distant bird	No	No	No
20160723_091700	https://www.ecosounds.org/listen/355029?start=9050	No	No	Eastern Whipbird	No	No	**Moderate to strong wind**
20150903_013400	https://www.ecosounds.org/listen/331734?start=5638	Insects between 5 and 6 kHz	No	No	No	No	**Moderate to strong wind**
20160705_052100	https://www.ecosounds.org/listen/354914?start=19249	Insects at 5 kHz	No	No	No	No	**Moderate to strong wind**
20160720_092600	https://www.ecosounds.org/listen/355006?start=9588	No	**Moderate to loud plane**	Distant bird	No	No	**Moderate to strong wind**
20160722_115300	https://www.ecosounds.org/listen/355014?start=18406	No	No	Yes	No	No	**Moderate to strong wind**
20160628_080200	https://www.ecosounds.org/listen/354854?start=4567	No	No	Distant bird	No	No	**Moderate to strong wind**

Cluster 10 – Gympie National Park

LIGHT TO MODERATE RAIN (3723 minutes) The dominant sound sources are provided in bold text.

date_ time	Hyperlinks	Insects	Planes	Birds	Rain	Thunder	Wind
20160619_ 014500	https://www.ecosounds.org/listen/354626?start=6295	No	No	No	**Light to moderate rain**	No	No
20151115_ 031300	https://www.ecosounds.org/listen/352895?start=11577	**Insects at 4.3 kHz**	No	No	**Light to moderate rain**	No	No
20160501_ 053700	https://www.ecosounds.org/listen/354255?start=20214	No	No	No	**Moderate rain**	No	No
20151109_ 095600	https://www.ecosounds.org/listen/352876?start=11413	No	No	Mistletoebird, Leaden Flycatcher, White-throated Honeyeater	**Light to moderate rain**	No	No
20151108_ 032300	https://www.ecosounds.org/listen/352789?start=12178	No	No	No	**Light to moderate rain**	**Loud thunder**	No
20160619_ 024400	https://www.ecosounds.org/listen/354626?start=9835	No	No	No	**Light to moderate rain**	No	No
20160616_ 021400	https://www.ecosounds.org/listen/354638?start=8036	No	No	No	**Light to moderate rain**	No	No
20160717_ 080900	https://www.ecosounds.org/listen/354935?start=4991	No	No	**Grey Fantail, Laughing Kookaburra**	**Light to moderate rain**	No	No
20160620_ 001300	https://www.ecosounds.org/listen/354630?start=765	No	No	No	Moderate rain	No	No
20150829_ 220700	https://www.ecosounds.org/listen/331785?start=6566	No	No	No	Moderate rain	No	No

Cluster 10 – Woondum National Park

LIGHT TO MODERATE RAIN (9138 minutes) The dominant sound sources are provided in bold text.

date_ time	Hyperlinks	Insects	Planes	Birds	Rain	Thunder	Wind
20150722_ 012100	https://www.ecosounds.org/listen/277089?start=4856	No	No	No	Light to moderate rain	No	No
20160619_ 055700	https://www.ecosounds.org/listen/354599?start=21417	No	No	No	Light to moderate rain	No	No
20160301_ 125900	https://www.ecosounds.org/listen/353991?start=22389	No	No	Lewin's Honeyeater, Scarlet Honeyeater	Light to moderate rain	No	No
20160615_ 224800	https://www.ecosounds.org/listen/354584?start=9027	No	No	No	Light to moderate rain	No	No
20151109_ 065500	https://www.ecosounds.org/listen/352821?start=551	No	No	No	Light to moderate rain	No	No
20160316_ 101800	https://www.ecosounds.org/listen/353599?start=12726	No	Yes	Golden Whistler	Light to moderate rain	No	No
20160615_ 164900	https://www.ecosounds.org/listen/354611?start=11837	No	No	Lewin's Honeyeater	Light to moderate rain	No	No
20160429_ 050700	https://www.ecosounds.org/listen/354195?start=18413	No	No	No	Light to moderate rain	No	No
20160716_ 151900	https://www.ecosounds.org/listen/354960?start=6439	No	No	No	Light to moderate rain	No	No
20150708_ 190300	https://www.ecosounds.org/listen/277049?start=19879	No	No	No	Light to moderate rain	No	No

Cluster 11 – Gympie National Park

BIRDS (35945 minutes) The dominant sound sources are provided in bold text.

date_ time	Hyperlinks	Insects	Planes	Birds	Rain	Thunder	Wind
20150828_ 132400	https://www.ecosounds.org/listen/331771?start=23887	No	No	**Scarlet Honeyeater, White-throated Honeyeater**	No	No	Slight wind
20151109_ 102400	https://www.ecosounds.org/listen/352876?start=13093	No	Distant plane	**Eastern Whipbird, Mistletoebird**	No	No	**Moderate wind**
20160205_ 065500	https://www.ecosounds.org/listen/353323?start=550	No	No	**Sulphur-crested Cockatoo, Eastern Whipbird, Leaden Flycatcher**	No	No	Slight wind
20160529_ 142600	https://www.ecosounds.org/listen/354490?start=5156	No	Distant plane	**Spotted Pardalote, Lewin's Honeyeater, Golden Whistler**	No	No	Slight wind
20160223_ 065400	https://www.ecosounds.org/listen/353458?start=490	Cicadas above 6 kHz	Distant plane	**Cicadabird, Torresian Crow, White-throated Treecreeper**	No	No	No
20150830_ 065500	https://www.ecosounds.org/listen/331775?start=545	No	No	**Scarlet Honeyeater, Rufous Whistler, White-throated Honeyeater**	No	No	Slight wind
20151021_ 152900	https://www.ecosounds.org/listen/354787?start=7039	**Cicadas above 6 kHz**	Distant plane	**Crested Shrike-tit, Scarlet Honeyeater, Leaden Flycatcher**	No	No	Slight wind
20150804_ 130800	https://www.ecosounds.org/listen/331450?start=22929	No	No	**White-throated Treecreeper, Lewin's Honeyeater, White-throated Honeyeater, Scarlet Honeyeater, Grey Fantail**	No	No	Slight wind
20160718_ 120800	https://www.ecosounds.org/listen/354967?start=19306	No	Distant plane	**Scarlet Honeyeater,**	No	No	Slight wind
20160711_ 091400	https://www.ecosounds.org/listen/354936?start=8886	No	Distant plane	**Scarlet Honeyeater, Torresian Crow, Eastern Whipbird**	No	No	No

Cluster 11 – Woondum National Park

BIRDS (38457 minutes) The dominant sound sources are provided in bold text.

date_ time	Hyperlinks	Insects	Planes	Birds	Rain	Thunder	Wind
20150731_094000	https://www.ecosounds.org/listen/277171?start=10445	No	Distant plane	**Scarlet Honeyeater, Yellow-faced Honeyeater**	No	No	No
20150820_104800	https://www.ecosounds.org/listen/331649?start=14528	No	Distant plane	**Scarlet Honeyeater, Golden Whistler**	No	No	No
20160217_142700	https://www.ecosounds.org/listen/353405?start=3317	**Cicadas between 2.8 and 10 kHz and buzzing**		Yellow-faced Honeyeater	No	No	No
20160126_135300	https://www.ecosounds.org/listen/353233?start=1277	Insects between 2.8 and 5 kHz	No	Mistletoebird	No	No	Moderate wind
20150928_101100	https://www.ecosounds.org/listen/333301?start=12313	No	Distant plane	**Rufous Whistler, Scarlet Honeyeater, Yellow-faced Honeyeater, Crested Shrike-tit**	No	No	No
20150924_045700	https://www.ecosounds.org/listen/333254?start=17815	No	No	**Eastern Yellow Robin**	No	No	No
20150819_071600	https://www.ecosounds.org/listen/331664?start=1810	No	**Moderately loud plane**	**Large-billed Scrubwren, Scarlet Honeyeater, Rufuous Whistler, White-throated Treecreeper**	No	No	No
20150830_174300	https://www.ecosounds.org/listen/331721?start=19615	Insects around 4.8 kHz	Plane	**Eastern Whipbird, Mistletoebird, White-throated Treecreeper, Scarlet Honeyeater**	No	No	No
20160211_054800	https://www.ecosounds.org/listen/353403?start=20878	No	No	**Large-billed Scrubwren, Australasian Figbird**	No	No	No
20150813_064100	https://www.ecosounds.org/listen/331542?start=24056	No	**Moderately loud plane**	**Scarlet Honeyeater, Australian Magpie**	No	No	No

Cluster 12 – Gympie National Park

CICADAS (1659 minutes) The dominant sound sources are provided in bold text.

date_ time	Hyperlinks	Insects	Planes	Birds	Rain	Thunder	Wind
20151223_ 135600	https://www.ecosounds.org/listen/353798?start=1458	**Loud Cicada and insect trail around 3kHz**	Moderately loud plane	No	No	No	No
20151212_ 043700	https://www.ecosounds.org/listen/353093?start=16616	**Loud Cicada call between 5 and 8 kHz**	No	Rufous Whistler, Crested Shrike-thrush	No	No	No
20151211_ 182700	https://www.ecosounds.org/listen/353090?start=17718	**Loud Cicada and insects**	No	No	No	No	No
20151210_ 183400	https://www.ecosounds.org/listen/353086?start=18142	**Loud Cicada broadband call**	Moderately loud plane	No	No	No	No
20151223_ 130600	https://www.ecosounds.org/listen/353822?start=22806	**Loud Cicada above 2.5 kHz**	No	No	No	No	No
20160211_ 184200	https://www.ecosounds.org/listen/353353?start=18615	**Cicada between 1.9 and 8 kHz**	Moderately loud plane	No	No	No	No
20151121_ 182400	https://www.ecosounds.org/listen/352899?start=17536	**Cicadas above 2kHz**	No	Pied Currawong, Spectacled Monarch, White-throated Treecreeper	No	No	No
20151213_ 094000	https://www.ecosounds.org/listen/353096?start=10445	**Insects between 6 and 8 kHz**	No	Lewin's Honeyeater, Yellow-faced Honeyeater, Grey Shrike-thrush, Eastern Whipbird	No	No	Moderate wind
20160130_ 031800	https://www.ecosounds.org/listen/353276?start=11876	**Insects at 4.5 kHz**	Distant plane	No	Very light rain	No	No
20160208_ 110700	https://www.ecosounds.org/listen/353341?start=15673	**Insects above 2.8 kHz**	No	White-throated Treecreeper	No	No	No

Cluster 12 – Woondum National Park

CICADAS (1320 minutes) The dominant sound sources are provided in bold text.

date_ time	Hyperlinks	Insects	Planes	Birds	Rain	Thunder	Wind
20151217_ 190300	https://www.ecosounds.org/listen/353171?start=19880	**Insects below 1kHz and Cicada above 2 kHz**	No	No	No	No	No
20160107_ 135600	https://www.ecosounds.org/listen/353897?start=1459	**Cicadas above 2.8 kHz**	No	No	No	No	Moderate wind
20151208_ 122900	https://www.ecosounds.org/listen/353051?start=20586	**Cicadas above 2.8 kHz**	No	Lewin's Honeyeater	No	No	No
20151227_ 131200	https://www.ecosounds.org/listen/353876?start=23161	**Cicadas above 2.8 kHz and above 7kHz**	No	Eastern Yellow Robin pipping	No	No	No
20160605_ 151600	https://www.ecosounds.org/listen/354518?start=10260	Yes	No + motorbikes	No	No	No	Wind
20151230_ 133900	https://www.ecosounds.org/listen/353923?start=444	**Cicadas around 3kHz and above**	No	No	No	No	No
20160106_ 100600	https://www.ecosounds.org/listen/353929?start=12003	**Cicadas around 3kHz and above**	**Moderately loud plane**	No	No	No	No
20151223_ 145800	https://www.ecosounds.org/listen/353862?start=5177	**Insects and cicadas around 3kHz and above**	No	No	No	No	Moderate wind
20151211_ 174700	https://www.ecosounds.org/listen/353087?start=15316	Insects around 3kHz and above	**Close plane and ticking**	No	No	No	No
20160106_ 141900	https://www.ecosounds.org/listen/353932?start=2832	**Cicadas around 3kHz and above**	No	Eastern Yellow Robin pipping	No	No	No

Cluster 13 – Gympie National Park

QUIET (33692 minutes) The dominant sound sources are provided in bold text.

date_ time	Hyperlinks	Insects	Planes	Birds	Rain	Thunder	Wind
20160417_ 215100	https://www.ecosounds.org/listen/354133?start=4156	**Insects between 5 and 5.8 kHz**	No	No	No	No	No
20160118_ 003200	https://www.ecosounds.org/listen/353187?start=1918	Very quiet insects between 3.8 and 5.5 kHz	No	No	No	No	No
20160609_ 015700	https://www.ecosounds.org/listen/354557?start=7013	Very quiet insects between 4 and 5 kHz	No	No	No	No	No
20160723_ 043500	https://www.ecosounds.org/listen/354987?start=16490	No	No	No	No	No	No
20151013_ 040800	https://www.ecosounds.org/listen/354705?start=14878	No	No	No	No	No	No
20150808_ 044600	https://www.ecosounds.org/listen/331480?start=17158	No	Distant plane	No	No	No	No
20150824_ 215100	https://www.ecosounds.org/listen/331756?start=5611	No	No	No	No	No	Distant wind
20160610_ 015100	https://www.ecosounds.org/listen/354585?start=6650	Very quiet insects around 5 kHz	No	No	No	No	No
20150630_ 183900	https://www.ecosounds.org/listen/269812?start=18439	Very quiet insects around 5 kHz	Distant plane	No	No	No	No
20150712_ 021000	https://www.ecosounds.org/listen/276963?start=7797	No	No	No	No	No	No

Cluster 13 – Woondum National Park

QUIET (50310 minutes) The dominant sound sources are provided in bold text.

date_ time	Hyperlinks	Insects	Planes	Birds	Rain	Thunder	Wind
20150719_ 014400	https://www.ecosounds.org/listen/277028?start=6235	No	Distant plane	No	No	No	No
20150709_ 222100	https://www.ecosounds.org/listen/276974?start=7413	No	No	No	No	No	No
20151018_ 221600	https://www.ecosounds.org/listen/354775?start=8571	**Crickets at 5 kHz**	No	No	No	No	No
20151002_ 232800	https://www.ecosounds.org/listen/333316?start=11433	No	No	No	No	No	Slight wind
20160519_ 195500	https://www.ecosounds.org/listen/354421?start=23000	Distant insects between 4.8 and 6 kHz	No	No	No	No	No
20150728_ 001800	https://www.ecosounds.org/listen/277144?start=1076	No	No	No	No	No	No
20150916_ 032200	https://www.ecosounds.org/listen/331873?start=12116	No	No	Yes	No	No	No
20160516_ 235300	https://www.ecosounds.org/listen/354378?start=12936	No	No	No	No	No	No
20160528_ 195200	https://www.ecosounds.org/listen/354459?start=22784	Very distant insects at 5 kHz	Distant plane	No	No	No	No
20160626_ 183100	https://www.ecosounds.org/listen/354828?start=17981	No	No	No	No	No	No

Cluster 14 – Gympie National Park

BIRDS (3683 minutes) The dominant sound sources are provided in bold text.

date_ time	Hyperlinks	Insects	Planes	Birds	Rain	Thunder	Wind
20151002_ 073200	https://www.ecosounds.org/listen/333288?start=2770	No	No	**Rufous Whistler, White-throated Treecreeper, Brown Cuckoo-dove, Eastern Whipbird, Mistletoebird**	No	No	**Moderate wind**
20151201_ 075400	https://www.ecosounds.org/listen/353016?start=4089	Quiet insects above 7kHz	No	**Brush Cuckoo, Cicadabird**	No	No	**Moderate wind**
20150923_ 062200	https://www.ecosounds.org/listen/333222?start=22916	No	No	**Brown Cuckoo dove, Leaden Flycatcher, Rufous Whistler, Torresian Crow, White-throated Treecreeper, Little Shrike-thrush**	No	No	No
20160217_ 185600	https://www.ecosounds.org/listen/353457?start=19463	**Insects above 2.8 kHz**	Distant plane	**Laughing Kookaburra**	No	No	No
20151009_ 172500	https://www.ecosounds.org/listen/333347?start=13998	No	No	**Unknown, White-throated Honeyeater**	No	No	No
20150909_ 094600	https://www.ecosounds.org/listen/331834?start=10811	No	**Loud plane**	**Rufous Whistler, Scarlet Honeyeater, Eastern Whipbird**	No	No	No
20150822_ 113100	https://www.ecosounds.org/listen/331656?start=17109	No	**Very loud plane**	**Scarlet Honeyeater, Grey Fantail, Rufous Whistler**	No	No	Moderate wind
20150815_ 141000	https://www.ecosounds.org/listen/331517?start=2292	No	No	**Scarlet Honeyeater, Eastern Whipird, Lewin's Honeyeater**	No	No	Moderate wind
20160222_ 050600	https://www.ecosounds.org/listen/353476?start=18358	Quiet insects between 3.5 kHz and 6 kHz	No	**Laughing Kookaburra**	No	No	Moderate wind
20160714_ 101300	https://www.ecosounds.org/listen/354950?start=12428	No	No	**Scarlet Honeyeater, White-throated Treecreeper**	No	No	**Moderate wind**

Cluster 14 – Woondum National Park

BIRDS (804 minutes) The dominant sound sources are provided in bold text.

date_ time	Hyperlinks	Insects	Planes	Birds	Rain	Thunder	Wind
20151102_ 123000	https://www.ecosounds.org/listen/352791?start=20651	No	**Moderately loud plane**	**Scarlet Honeyeater, Mistletoebird**	No	No	No
20150705_ 081700	https://www.ecosounds.org/listen/269835?start=5462	No	**Loud plane**	**Scarlet Honeyeater, Lewin's Honeyeater, Brown Cuckoo-dove**	No	No	No
20151130_ 171700	https://www.ecosounds.org/listen/353019?start=13524	**Very loud cicadas above 2.8 and insects at 2.0 kHz**	No	**Pied Currawong**	No	No	No
20150822_ 112200	https://www.ecosounds.org/listen/331662?start=16566	No	**Very loud plane**	**Scarlet Honeyeater**	Light rain	No	No
20151025_ 093800	https://www.ecosounds.org/listen/352713?start=10323	Distant insects	**Very loud plane**	**Golden Whistler, Scarlet Honeyeater, Yellow-faced Honeyeater**	No	No	No
20151008_ 062300	https://www.ecosounds.org/listen/333368?start=22978	No	**Very loud plane**	**White-throated Treecreeper, Rufous Whistler, White-throated Honeyeater, Eastern Whipbird**	No	No	No
20150824_ 065600	https://www.ecosounds.org/listen/331779?start=612	No	**Moderately loud plane**	**Scarlet Honeyeater, White-throated Treecreeper, Golden Whistler**	No	No	No
20150627_ 072900	https://www.ecosounds.org/listen/331622?start=2590	No	**Loud plane**	**Scarlet Honeyeater, Pied Currawong**	No	No	Yes
20160113_ 125000	https://www.ecosounds.org/listen/354047?start=21846	No	**Moderately loud plane**	Scarlet Honeyeater	No	No	No
20150806_ 135600	https://www.ecosounds.org/listen/331485?start=1462	No	**Moderate to loud plane**	**Yellow-faced Honeyeater**	No	No	No

Cluster 15 – Gympie National Park

BIRDS (20245 minutes) The dominant sound sources are provided in bold text.

date_ time	Hyperlinks	Insects	Planes	Birds	Rain	Thunder	Wind
20160626_ 151300	https://www.ecosounds.org/listen/354807?start=12602	No	Moderately loud plane	**Australasian Figbird, White-throated Honeyeater**	No	No	No
20150801_ 102700	https://www.ecosounds.org/listen/277123?start=13265	No	No	**Scarlet Honeyeater, Lewin's Honeyeater**	No	No	No
20160713_ 143900	https://www.ecosounds.org/listen/354922?start=4044	Insects between 7 and 9 kHz	Quiet plane	**Scarlet Honeyeater**	No	No	No
20150725_ 122000	https://www.ecosounds.org/listen/277107?start=20048	No	Quiet to Moderately loud plane	Scarlet Honeyeater, White-throated Treecreeper	No	No	**Moderate wind**
20151108_ 042300	https://www.ecosounds.org/listen/352789?start=15778	Insects between 5 and 5.5 kHz	No	**Eastern Yellow Robin, Spectacled Monarch**	No	No	Moderate wind
20150828_ 092000	https://www.ecosounds.org/listen/331771?start=9247	No	No	**Scarlet honeyeater, Mistletoebird**	No	No	**Moderate wind**
20151112_ 062900	https://www.ecosounds.org/listen/352882?start=23338	No	No	**White-throated Treecreeper, Mistletoebird, Brown Cuckoo-dove**	No	No	Slight wind
20160518_ 091100	https://www.ecosounds.org/listen/354379?start=8707	No	No	**White-throated Treecreeper, Grey Fantail**	No	No	Slight wind
20160612_ 130300	https://www.ecosounds.org/listen/354602?start=7298	No	No	**White-throated Treecreeper, Torresian Crow**	No	No	No
20150922_ 152500	https://www.ecosounds.org/listen/333221?start=6799	No	No	**Scarlet Honeyeater, Torresian Crow**	No	No	No

Cluster 15 – Woondum National Park

BIRDS (11345 minutes) The dominant sound sources are provided in bold text.

date_ time	Hyperlinks	Insects	Planes	Birds	Rain	Thunder	Wind
20160102_ 061100	https://www.ecosounds.org/listen/353934?start=22255	No	No	**Unknown, White-throated Treecreeper**	No	No	No
20160407_ 172300	https://www.ecosounds.org/listen/353772?start=13871	Insects at 8 kHz	No	**Eastern Whipbird, Lewin's Honeyeater**	No	No	No
20150902_ 085600	https://www.ecosounds.org/listen/331733?start=7809	No	Very distant plane	**Scarlet Honeyeater, Yellow-faced Honeyeater**	No	No	No
20160617_ 105900	https://www.ecosounds.org/listen/354591?start=15185	No	No	**White-throated Treecreeper, Grey Fantail**	No	No	**Moderate wind**
20160605_ 063800	https://www.ecosounds.org/listen/354488?start=23874	No	**Moderately loud plane**	**Scarlet Honeyeater**	No	No	No
20151227_ 210600	https://www.ecosounds.org/listen/353849?start=2897	Insects between 5 and 7.2 kHz	No	**Frog(?)**	Light rain	No	No
20150809_ 113200	https://www.ecosounds.org/listen/331498?start=17168	No	No	**Scarlet Honeyeater, Yellow-faced Honeyeater**	No	No	No
20160325_ 073500	https://www.ecosounds.org/listen/353660?start=2948	No	No	**Rainbow Lorikeet, Pied Currawong**	No	No	No
20160305_ 145200	https://www.ecosounds.org/listen/353984?start=4823	**Insects at 8 kHz and above 5 kHz**	No	Yes	No	No	No
20160331_ 171900	https://www.ecosounds.org/listen/353716?start=13636	**Cicada zizzt**	No	**Australian King Parrot, White-throated Treecreeper**	No	No	No

Cluster 16 – Gympie National Park

CICADAS (6352 minutes) The dominant sound sources are provided in bold text.

date_ time	Hyperlinks	Insects	Planes	Birds	Rain	Thunder	Wind
20160110_ 213600	https://www.ecosounds.org/listen/353998?start=4712	Insects between 4 and 6 and above 8 kHz	No	Southern Boobook - quiet	No	No	No
20160107_ 220600	https://www.ecosounds.org/listen/353989?start=4511	Insects between 4 and 6 and above 7 kHz	No	No	No	No	No
20151106_ 203200	https://www.ecosounds.org/listen/352783?start=870	Insects at 5 kHz	No	No	No	No	Moderate to strong wind
20151224_ 163000	https://www.ecosounds.org/listen/353824?start=10698	Insects above 6 kHz	No	White-throated Treecreeper, Cicadabird	No	No	No
20151124_ 135000	https://www.ecosounds.org/listen/352939?start=1097	Insects above 6 kHz	No	Rufous Whistler	No	No	No
20160207_ 233600	https://www.ecosounds.org/listen/353337?start=21675	Insects between 4 and 6 kHz and above 8 kHz	No	No	No	No	No
20160103_ 083800	https://www.ecosounds.org/listen/353902?start=6728	Distant insects above 7 kHz	No	Cicadabird	No	No	No
20160128_ 154800	https://www.ecosounds.org/listen/353269?start=8176	Insects above 5.5 kHz	Moderately loud plane	Lewin's Honeyeater, White-throated Honeyeater	No	No	No
20160112_ 152600	https://www.ecosounds.org/listen/354033?start=6858	Insects above 6 kHz	No	No	No	No	Light wind
20151102_ 091800	https://www.ecosounds.org/listen/352766?start=9132	Insects above 6 kHz	Moderately loud plane	Rufous Whistler, White-throated Treecreeper	No	No	No

Cluster 16 – Woondum National Park

CICADAS (1740 minutes) The dominant sound sources are provided in bold text.

date_ time	Hyperlinks	Insects	Planes	Birds	Rain	Thunder	Wind
20160110_ 111900	https://www.ecosounds.org/listen/354060?start=16384	Insects above 3 kHz	No	Grey shrike-thrush	No	No	No
20151129_ 170000	https://www.ecosounds.org/listen/353017?start=21032	Insects above 5.8 kHz	Moderate plene	Cicadabird	No	No	No
20160108_ 170600	https://www.ecosounds.org/listen/354029?start=12863	Insects above 5 kHz	Quiet plane	No	No	No	Moderate wind
20151130_ 142300	https://www.ecosounds.org/listen/353019?start=3084	Insects above 2.8 kHz	Moderately loud plane	Distant bird	No	No	No
20160501_ 225900	https://www.ecosounds.org/listen/354283?start=16511	Insects above 5 kHz	No	No	No	No	Moderate wind
20151117_ 130600	https://www.ecosounds.org/listen/352931?start=22808	Insects above 6 kHz	No	Distant bird	No	No	Moderate wind
20160108_ 160000	https://www.ecosounds.org/listen/354029?start=8903	Insects above 5 kHz	No	No	No	No	Moderate wind
20160114_ 173400	https://www.ecosounds.org/listen/354057?start=14529	Insects above 7 kHz	No	No	No	No	Moderate wind
20160106_ 172000	https://www.ecosounds.org/listen/353932?start=13692	Insects above 5 kHz	Quiet plane	White-throated Treecreeper	No	No	No
20151209_ 161200	https://www.ecosounds.org/listen/353079?start=9615	Insects between 7 and 9 kHz	No	Pied Currawong	No	No	Moderate wind

Cluster 17 – Gympie National Park

LIGHT RAIN OR INSECTS (5069 minutes) - Inconsistent The dominant sound sources are provided in bold text.

date_ time	Hyperlinks	Insects	Planes	Birds	Rain	Thunder	Wind
20160327_ 032000	https://www.ecosounds.org/listen/353644?start=11996	**Distant insects between 4 and 6 kHz**	No	No	No	No	No
20160602_ 030900	https://www.ecosounds.org/listen/354530?start=11337	Very distant insects between 4 and 5 kHz	No	No	No	No	Slight wind
20160107_ 084800	https://www.ecosounds.org/listen/353859?start=7331	**Cicada zizzt above 5 kHz**	No	**Rufous Whistler, White-throated Treecreeper, Grey shrike-thrush**	No	No	No
20160106_ 021900	https://www.ecosounds.org/listen/353890?start=8336	**Insects between 4 and 6 kHz**	No	No	**Very light rain**	No	No
20160311_ 041800	https://www.ecosounds.org/listen/353549?start=15476	**Insects between 4 and 6 kHz**	No	No	**Very light rain**	No	No
20160429_ 195900	https://www.ecosounds.org/listen/354223?start=23210	**Insects at 3 kHz and above 5 kHz**	No	No	No	No	No
20151226_ 071600	https://www.ecosounds.org/listen/353811?start=1809	Insects at 3 kHz	No	**Fan-tailed Cuckoo, Mistletoebird**	No	No	No
20160314_ 021100	https://www.ecosounds.org/listen/353564?start=7858	**Insects between 4 and 6 kHz**	No	No	No	No	No
20160311_ 034600	https://www.ecosounds.org/listen/353549?start=13556	Insects between 4 and 5 kHz	No	No	**Very light rain**	No	No
20150630_ 025100	https://www.ecosounds.org/listen/269811?start=10257	No	No	No	**Light rain**	No	No

Cluster 17 – Woondum National Park

LIGHT RAIN OR INSECTS (12957 minutes) - Inconsistent The dominant sound sources are provided in bold text.

date_ time	Hyperlinks	Insects	Planes	Birds	Rain	Thunder	Wind
20150821_ 053900	https://www.ecosounds.org/listen/331655?start=20337	No	No	Eastern Yellow Robin - distant	**Light rain**	No	No
20151208_ 154300	https://www.ecosounds.org/listen/353074?start=7875	Very distant high frequency insects	No	**White-throated Treecreeper**	No	No	No
20150812_ 224700	https://www.ecosounds.org/listen/331535?start=8962	No	No	Spotted Pardalote(?)	**Light rain**	No	No
20160603_ 123100	https://www.ecosounds.org/listen/354501?start=20707	No	No	Distant	**Light rain**	No	No
20160303_ 072000	https://www.ecosounds.org/listen/353976?start=2050	No	No	**Rufous Fantail**	No	No	No
20151030_ 005500	https://www.ecosounds.org/listen/352751?start=3296	Insects between 4 and 6 kHz	No	No	**Light rain**	No	No
20160319_ 214200	https://www.ecosounds.org/listen/353615?start=5063	Insects between 5 and 7	No	No	**Light rain**	No	No
20160604_ 014400	https://www.ecosounds.org/listen/354503?start=6235	Insects around 5 kHz	No	No	**Light rain**	No	No
20151124_ 141600	https://www.ecosounds.org/listen/352969?start=2658	**Cicadas above 5 kHz**	No	Yellow-faced Honeyeater	No	No	No
20151202_ 180000	https://www.ecosounds.org/listen/353048?start=16092	Insects between 5 and 8 kHz	No	Yes	**Light rain (?)**	No	No

Cluster 18 – Gympie National Park

MODERATE RAIN (4073 minutes) The dominant sound sources are provided in bold text.

date_ time	Hyperlinks	Insects	Planes	Birds	Rain	Thunder	Wind
20160204_ 001200	https://www.ecosounds.org/listen/353317?start=717	No	No	No	Moderate rain	No	No
20160603_ 233600	https://www.ecosounds.org/listen/354522?start=11914	No	No	No	Moderate rain	No	No
20160313_ 150900	https://www.ecosounds.org/listen/353585?start=8870	No	No	Torresian Crow	Moderate rain	No	No
20151114_ 162700	https://www.ecosounds.org/listen/352892?start=10518	No	No	White-throated Honeyeater	Moderate rain	Moderately loud thunder	No
20160619_ 153700	https://www.ecosounds.org/listen/354652?start=14980	No	No	No	Moderate rain	No	No
20160716_ 000100	https://www.ecosounds.org/listen/354931?start=55	No	No	No	Moderate rain	No	No
20160206_ 070400	https://www.ecosounds.org/listen/353328?start=1087	No	No	Yes	Moderate rain	No	No
20160202_ 185600	https://www.ecosounds.org/listen/353288?start=19458	Insects at 2.3 kHz	No	No	Moderate rain	No	No
20160602_ 143500	https://www.ecosounds.org/listen/354504?start=3801	No	No	Lewin's Honeyeater	Moderate rain	No	No
20160503_ 010900	https://www.ecosounds.org/listen/354265?start=4138	No	No	No	Moderate rain	No	No

Cluster 18 – Woondum National Park

MODERATE RAIN (5522 minutes) The dominant sound sources are provided in bold text.

date_ time	Hyperlinks	Insects	Planes	Birds	Rain	Thunder	Wind
20151211_ 205800	https://www.ecosounds.org/listen/353063?start=2426	No	No	White-throated Honeyeater	Moderate rain	Moderately loud thunder	No
20151114_ 201900	https://www.ecosounds.org/listen/352856?start=86	No	No	No	Moderate rain	Moderately loud thunder	No
20160616_ 005300	https://www.ecosounds.org/listen/354609?start=3177	No	No	No	Moderate rain	No	No
20160604_ 022000	https://www.ecosounds.org/listen/354503?start=8395	No	No	No	Moderate rain	No	No
20160604_ 065600	https://www.ecosounds.org/listen/354482?start=605	No	No	No	Moderate rain	No	No
20160430_ 232200	https://www.ecosounds.org/listen/354214?start=11039	No	No	No	Moderate rain	No	No
20151109_ 034600	https://www.ecosounds.org/listen/352844?start=13558	No	No	No	Moderate rain	No	No
20150627_ 203600	https://www.ecosounds.org/listen/331590?start=1109	No	No	No	Moderate rain	No	No
20150917_ 202100	https://www.ecosounds.org/listen/331883?start=207	No	No	No	Moderate rain	No	No
20151107_ 183000	https://www.ecosounds.org/listen/352815?start=17892	Insects at 0.9 kHz	No	No	Moderate rain	No	No

Cluster 19 – Gympie National Park

MODERATE WIND (11580 minutes) The dominant sound sources are provided in bold text.

date_ time	Hyperlinks	Insects	Planes	Birds	Rain	Thunder	Wind
20160116_ 122400	https://www.ecosounds.org/listen/354046?start=20290	No	No	No	No	No	**Moderate to strong wind**
20160405_ 100100	https://www.ecosounds.org/listen/353759?start=11711	No	No	Yes	No	No	**Moderate wind**
20160414_ 205500	https://www.ecosounds.org/listen/354090?start=2250	No	No	No	No	No	**Strong wind**
20160209_ 030800	https://www.ecosounds.org/listen/353366?start=11278	Quiet insects between 4 and 7 kHz	No	No	No	No	**Moderate wind**
20160417_ 151400	https://www.ecosounds.org/listen/354119?start=4683	Insects between 7 and 8 kHz	No	No	No	No	**Moderate wind**
20151010_ 124900	https://www.ecosounds.org/listen/333350?start=21790	Quiet cicadas above 6 kHz	No	No	No	No	**Moderate wind**
20160704_ 204200	https://www.ecosounds.org/listen/354868?start=1422	Quiet insects between 4 and 5 kHz	**Loud plane**	No	No	No	**Moderate wind**
20151121_ 074200	https://www.ecosounds.org/listen/352897?start=3366	Quiet cicadas above 5 kHz	Quiet plane	Distant bird	No	No	**Moderate wind**
20160208_ 213200	https://www.ecosounds.org/listen/353351?start=4479	**Insects above 4 kHz**	No	No	No	No	No
20160706_ 134200	https://www.ecosounds.org/listen/354891?start=589	No	**Moderate plane**	No	No	No	**Moderate wind**

Cluster 19 – Woondum National Park

MODERATE WIND (3904 minutes) The dominant sound sources are provided in bold text.

date_ time	Hyperlinks	Insects	Planes	Birds	Rain	Thunder	Wind
20160628_ 050300	https://www.ecosounds.org/listen/354853?start=18177	No	No	No	No	No	**Moderate wind**
20151126_ 201600	https://www.ecosounds.org/listen/353000?start=24258	Quiet insects between 5 and 8 kHz	Moderately loud plane	No	No	No	**Moderate to strong wind**
20151211_ 041400	https://www.ecosounds.org/listen/353060?start=15237	Quiet insects between 5 and 6 kHz	Quiet plane	**Eastern Yellow Robin + Frog calls**	No	No	No
20160706_ 125900	https://www.ecosounds.org/listen/354893?start=22368	No	No	No	No	No	**Moderate wind**
20150714_ 132500	https://www.ecosounds.org/listen/277007?start=23946	No	No	Eastern Yellow Robin	No	No	**Moderate wind**
20150903_ 150800	https://www.ecosounds.org/listen/331752?start=5778	No	No	Scarlet Honeyeater, Eastern Whipbird	No	No	**Moderate to strong wind**
20160628_ 002200	https://www.ecosounds.org/listen/354853?start=1317	No	No	No	No	No	**Moderate wind**
20160321_ 141800	https://www.ecosounds.org/listen/353646?start=2785	Insects between 8 and 9 kHz	**Loud plane**	Distant bird	No	No	**Moderate wind**
20150927_ 160500	https://www.ecosounds.org/listen/333298?start=17560	No	No	No	No	No	**Moderate to strong wind**
20150806_ 124900	https://www.ecosounds.org/listen/331483?start=21790	No	**Moderately loud plane**	Scarlet Honeyeater, Eastern Whipbird	No	No	**Moderate wind**

Cluster 20 – Gympie National Park

MODERATE WIND (3950 minutes) The dominant sound sources are provided in bold text.

date_ time	Hyperlinks	Insects	Planes	Birds	Rain	Thunder	Wind
20160605_ 052600	https://www.ecosounds.org/listen/354515?start=19557	No	No	No	No	No	**Moderate wind**
20160605_ 020200	https://www.ecosounds.org/listen/354515?start=7317	No	No	No	No	No	**Moderate wind**
20160205_ 023600	https://www.ecosounds.org/listen/353322?start=9357	**Insects between 3 and 6 kHz and above 8 kHz**	Quiet plane	No	No	No	No
20160204_ 034100	https://www.ecosounds.org/listen/353317?start=13257	**Insects between 4 and 7 kHz**	Quiet plane	No	No	No	No
20150708_ 090700	https://www.ecosounds.org/listen/276955?start=8469	No	Quiet plane	Distant bird	No	No	Moderate wind
20150629_ 085400	https://www.ecosounds.org/listen/269845?start=7691	No	Quiet plane	No	No	No	**Moderate wind**
20160206_ 230800	https://www.ecosounds.org/listen/353306?start=10227	**Insects between 4 and 6 kHz**	Quiet plane	No	No	No	No
20160614_ 014200	https://www.ecosounds.org/listen/354629?start=6117	No	Quiet plane	No	No	No	**Moderate wind**
20150720_ 071200	https://www.ecosounds.org/listen/277068?start=1571	No	No	Eastern Whipbird	No	No	**Moderate wind**
20160206_ 215500	https://www.ecosounds.org/listen/353306?start=5847	**Insects between 4 and 6 kHz and above 8 kHz**	No	No	No	No	No

Cluster 20 – Woondum National Park

MODERATE WIND (17562 minutes) The dominant sound sources are provided in bold text.

date_ time	Hyperlinks	Insects	Planes	Birds	Rain	Thunder	Wind
20160303_ 023200	https://www.ecosounds.org/listen/353997?start=9118	Insects between 4 and 8 kHz	No	No	No	No	**Slight to moderate wind**
20160721_ 030900	https://www.ecosounds.org/listen/355009?start=11331	No	No	No	No	No	**Moderate wind**
20160722_ 092900	https://www.ecosounds.org/listen/355014?start=9766	No	No	White-throated Treecreeper	No	No	**Moderate wind**
20160718_ 140000	https://www.ecosounds.org/listen/354997?start=1669	No	No	No	No	No	**Moderate wind**
20160201_ 002900	https://www.ecosounds.org/listen/353333?start=1738	Insects between 2 and 7 kHz	No	**Frogs at 2 kHz**	No	No	Moderate wind
20160721_ 052000	https://www.ecosounds.org/listen/355009?start=19191	No	No	No	No	No	**Moderate wind**
20150813_ 040100	https://www.ecosounds.org/listen/331542?start=14456	No	No	No	No	No	**Moderate wind**
20160707_ 015700	https://www.ecosounds.org/listen/354920?start=7010	No	Microphone problem	No	No	No	**Moderate wind**
20160712_ 182400	https://www.ecosounds.org/listen/354969?start=17538	Insects between 5 and 6 kHz	Microphone problem	No	No	No	**Moderate wind**
20160618_ 220300	https://www.ecosounds.org/listen/354597?start=6329	Insects at 5 kHz	No	No	No	No	**Moderate wind**

Cluster 21 – Gympie National Parks

LIGHT RAIN (1171 minutes) The dominant sound sources are provided in bold text.

date_ time	Hyperlinks	Insects	Planes	Birds	Rain	Thunder	Wind
20150821_ 040900	https://www.ecosounds.org/listen/331654?start=14937	No	No	No	**Moderate rain**	No	Moderate wind
20151014_ 225900	https://www.ecosounds.org/listen/354708?start=9684	No	No	No	**Light to moderate rain**	No	Moderate wind
20160305_ 233900	https://www.ecosounds.org/listen/353970?start=12094	Insects between 5 and 6 kHz	No	No	**Light rain**	No	Moderate wind
20160430_ 043200	https://www.ecosounds.org/listen/354225?start=16312	Insects at 5 kHz	No	No	**Light rain**	No	Moderate wind
20160103_ 194100	https://www.ecosounds.org/listen/353880?start=22161	Insects between 4 and 6 and above 8 kHz	No	No	No	No	No
20151115_ 032500	https://www.ecosounds.org/listen/352895?start=12297	**Insects at 4.4 kHz**	No	No	**Light to moderate rain**	No	Moderate wind
20160503_ 133800	https://www.ecosounds.org/listen/354264?start=384	No	No	**Yes**	No	No	Moderate wind
20160311_ 060500	https://www.ecosounds.org/listen/353549?start=21896	No	No	Grey shrike-thrush, White-throated Treecreeper	**Light rain**	No	Moderate wind
20150830_ 041400	https://www.ecosounds.org/listen/331794?start=15238	No	No	No	**Moderate rain**	No	Moderate wind
20160429_ 023000	https://www.ecosounds.org/listen/354245?start=8991	No	No	No	**Light rain**	No	No

Cluster 21 – Woondum National Park

LIGHT RAIN (6961 minutes) The dominant sound sources are provided in bold text.

date_ time	Hyperlinks	Insects	Planes	Birds	Rain	Thunder	Wind
20160313_ 214700	https://www.ecosounds.org/listen/353609?start=13997	Quiet insects between 4 and 6 kHz	No	No	**Light rain**	No	Distant wind
20160126_ 172100	https://www.ecosounds.org/listen/353233?start=13757	Quiet insects between 5.4 and 7 kHz	No	**Golden Whistler**	Light rain	No	No
20160430_ 052700	https://www.ecosounds.org/listen/354224?start=19606	No	No	No	**Light rain**	No	Distant wind
20160428_ 212500	https://www.ecosounds.org/listen/354203?start=4014	Insects between 4.8 and 5.6 kHz	No	No	**Light rain**	No	Distant wind
20160615_ 202500	https://www.ecosounds.org/listen/354584?start=447	Insects between 4 and 5 kHz	No	No	**Light rain**	No	Distant wind
20160116_ 020000	https://www.ecosounds.org/listen/354058?start=7196	Insects between 4 and 5 kHz	No	No	**Light rain**	No	Distant wind
20150812_ 191900	https://www.ecosounds.org/listen/331541?start=20833	Insects at 5 kHz	No	No	**Light rain**	No	Distant wind
20160429_ 075600	https://www.ecosounds.org/listen/354197?start=4189	No	No	Yes	**Light rain**	No	Distant wind
20150821_ 004400	https://www.ecosounds.org/listen/331655?start=2637	No	No	No	**Light rain**	No	Distant wind
20151229_ 162600	https://www.ecosounds.org/listen/353899?start=8406	No	No	Scarlet Honeyeater, Australasian Figbird - distant	**Light rain**	No	Distant wind

Cluster 22 – Gympie National Park

INSECTS AND BIRDS (27000 minutes) The dominant sound sources are provided in bold text.
MID-FREQUENCY INSECTS

date_ time	Hyperlinks	Insects	Planes	Birds	Rain	Thunder	Wind
20160320_ 033900	https://www.ecosounds.org/listen/353588?start=13138	**Insects between 3.8 and 6 kHz**	No	No	No	No	No
20160113_ 172600	https://www.ecosounds.org/listen/354012?start=14055	**Insects above 7 kHz**	No	White-throated Treecreeper	No	No	No
20160221_ 031000	https://www.ecosounds.org/listen/353446?start=11397	**Insects between 3.8 and 6 kHz**	No	No	No	No	No
20160218_ 214400	https://www.ecosounds.org/listen/353435?start=5194	**Insects between 3.8 and 6 kHz**	No	No	No	No	No
20150826_ 052800	https://www.ecosounds.org/listen/331780?start=19676	No	No	**Eastern Yellow Robin**	No	No	Moderate wind
20160217_ 215600	https://www.ecosounds.org/listen/353433?start=5916	**Insects between 3.8 and 6.8 kHz**	No	No	No	No	No
20151118_ 194400	https://www.ecosounds.org/listen/352885?start=22339	**Insects between 4 and 6 kHz**	Distant plane	No	No	No	No
20160403_ 233400	https://www.ecosounds.org/listen/353726?start=8998	**Insects between 4 and 6 kHz**	No	No	No	No	No
20160315_ 015500	https://www.ecosounds.org/listen/353590?start=6897	**Quiet insects between 4 and 6 kHz**	No	No	No	No	No
20160501_ 142000	https://www.ecosounds.org/listen/354236?start=14869	No	No	**Rufous Fantail(?)**	No	No	No

Cluster 22 – Woondum National Park

INSECTS AND BIRDS (47630 minutes) The dominant sound sources are provided in bold text.
MID-FREQUENCY INSECTS

date_ time	Hyperlinks	Insects	Planes	Birds	Rain	Thunder	Wind
20160714_ 144800	https://www.ecosounds.org/listen/354977?start=4575	No	Distant plane	Distant bird	No	No	Moderate wind
20160601_ 095000	https://www.ecosounds.org/listen/354471?start=11048	No	Distant plane	**Spotted Pardalote, White-throated Honeyeater**	No	No	Moderate wind
20160624_ 013000	https://www.ecosounds.org/listen/354690?start=5388	No	No	No	No	No	**Moderate wind**
20150708_ 125300	https://www.ecosounds.org/listen/277053?start=22029	No	Distant plane	**Scarlet Honeyeater**	No	No	Moderate to strong wind
20160723_ 022600	https://www.ecosounds.org/listen/355016?start=8750	No	No	No	No	No	**Moderate wind**
20160118_ 000900	https://www.ecosounds.org/listen/353198?start=537	**Insects between 4 and 6 kHz**	No	No	No	No	Moderate wind
20160213_ 215600	https://www.ecosounds.org/listen/353388?start=5899	**Insects between 4 and 7 kHz**	No	No	No	No	Moderate wind
20160422_ 060900	https://www.ecosounds.org/listen/354164?start=22136	No	No	Distant bird	No	No	Moderate wind
20150725_ 060000	https://www.ecosounds.org/listen/337203?start=21597	No	Moderately loud plane	**Eastern Yellow Robin - distant**	No	No	Moderate to strong wind
20160221_ 010800	https://www.ecosounds.org/listen/353419?start=4074	**Insects between 4 and 7 kHz**	No	No	No	No	Moderate wind

Cluster 23 – Gympie National Park

PLANES, MOTORBIKES, THUNDER (2690 minutes) The dominant sound sources are provided in bold text.

date_ time	Hyperlinks	Insects	Planes	Birds	Rain	Thunder	Wind
20160130_ 171400	https://www.ecosounds.org/listen/353278?start=13339	No	No	No	**Heavy rain**	**Moderately loud thunder**	No
20160408_ 173700	https://www.ecosounds.org/listen/353773?start=14719	Quiet insects at 5.5 kHz	**Moderately loud plane**	**White-throated Treecreeper, White-throated Honeyeater**	No	No	No
20151230_ 005000	https://www.ecosounds.org/listen/353887?start=2997	**Insects at 4 and 5 kHz**	Quiet plane	No	No	No	No
20151118_ 143400	https://www.ecosounds.org/listen/352885?start=3739	Quiet cicadas	**Loud plane**	Channel-billed Cuckoo, White-throated Treecreeper	No	No	No
20160114_ 183100	https://www.ecosounds.org/listen/354016?start=17958	Insects above 5 kHz	**Loud plane**	Torresian Crow	No	No	No
20150714_ 154800	https://www.ecosounds.org/listen/277029?start=8179	No	**Loud plane**	Scarlet Honeyeater, White-throated Honeyeater	No	No	No
20160412_ 120300	https://www.ecosounds.org/listen/354066?start=19027	No	**Loud plane**	Grey Fantail, Lewin's Honeyeater, Spangled Drongo	No	No	No
20151204_ 030000	https://www.ecosounds.org/listen/353009?start=10798	Quiet insects at 5 kHz	**Moderate plane**	**Channel-billed Cuckoo**	No	No	No
20160217_ 221600	https://www.ecosounds.org/listen/353433?start=7116	**Insects between 4 and 6.5 kHz**	**Loud plane**	No	No	No	No
20150704_ 121200	https://www.ecosounds.org/listen/269823?start=19568	Insects at 8 kHz	No + **motorbikes**	Scarlet Honeyeater, Lewin's Honeyeater	No	No	No

Cluster 23 – Woondum National Park

PLANES, MOTORBIKES, THUNDER (1134 minutes) The dominant sound sources are provided in bold text.

date_ time	Hyperlinks	Insects	Planes	Birds	Rain	Thunder	Wind
20160701_ 174300	https://www.ecosounds.org/listen/354845?start=15079	No	No + **Vehicle**	No	No	No	No
20160130_ 174700	https://www.ecosounds.org/listen/353273?start=15311	No	No	No	**Moderate rain**	**Loud thunder**	No
20151210_ 195300	https://www.ecosounds.org/listen/353059?start=22880	Insects above 4.5 kHz	**Loud plane**	No	No	No	No
20160623_ 090300	https://www.ecosounds.org/listen/354673?start=8209	No	**Loud plane**	Lewin's Honeyeater	No	No	No
20160608_ 074700	https://www.ecosounds.org/listen/354553?start=3650	No	**Loud plane**	Eastern Whipbird	No	No	No
20151211_ 205000	https://www.ecosounds.org/listen/353063?start=1946	No	No	No	**Moderate to heavy rain**	**Loud thunder**	No
20151012_ 181300	https://www.ecosounds.org/listen/354725?start=16879	**Insects at 1.9 and 6.3 kHz**	**Moderate plane**	No	No	No	**Moderate wind**
20151121_ 175900	https://www.ecosounds.org/listen/352925?start=16038	Cicadas above 6 kHz	No + **motorbikes**	Scarlet Honeyeater	No	No	No
20160317_ 174100	https://www.ecosounds.org/listen/353601?start=14953	Quiet insects at 6 kHz	**Moderately loud plane + motorbikes**	White-throated Treecreeper, Lewin's Honeyeater	No	No	No
20160409_ 110900	https://www.ecosounds.org/listen/353778?start=15788	No	**Loud plane**	No	No	No	No

Cluster 24 – Gympie National Park

WIND AND/OR CICADAS (6175 minutes) - Inconsistent The dominant sound sources are provided in bold text.

date_ time	Hyperlinks	Insects	Planes	Birds	Rain	Thunder	Wind
20160115_ 183500	https://www.ecosounds.org/listen/354023?start=18196	**Cicadas between 6 and 9 kHz**	Moderately loud plane	Torresian Crow	No	No	Moderate wind
20160313_ 152100	https://www.ecosounds.org/listen/353585?start=9590	**Cicadas in background above 6 kHz**	No	Torresian Crow	No	No	**Moderate wind**
20160310_ 145900	https://www.ecosounds.org/listen/353521?start=5238	**Cicadas above 6 kHz**	**Moderately loud plane**	White-throated Honeyeater	No	No	Moderate wind
20160213_ 040900	https://www.ecosounds.org/listen/353385?start=14937	No	No	No	No	No	**Moderate wind**
20151228_ 122800	https://www.ecosounds.org/listen/353842?start=20528	No	No	Yes	No	No	**Strong wind**
20160128_ 223500	https://www.ecosounds.org/listen/353267?start=8246	**Cicadas in background and insects at 5 and 6 kHz**	No	No	No	No	No
20151108_ 214000	https://www.ecosounds.org/listen/352848?start=13400	**Insects between 4 and 6 kHz**	No	No	No	No	Moderate to strong wind
20160116_ 152900	https://www.ecosounds.org/listen/353158?start=10109	No	No	White-throated Honeyeater	No	No	**Strong wind**
20160115_ 204400	https://www.ecosounds.org/listen/354021?start=1588	Insects above 8 kHz and between 5 and 6 kHz	Quiet plane	No	No	No	**Strong wind**
20160625_ 002000	https://www.ecosounds.org/listen/354677?start=1190	No	No	No	No	No	**Strong wind**

Cluster 24 – Woondum National Park

WIND AND/OR CICADAS (4030 minutes) - Inconsistent The dominant sound sources are provided in bold text.

date_ time	Hyperlinks	Insects	Planes	Birds	Rain	Thunder	Wind
20150919_ 202100	https://www.ecosounds.org/listen/331886?start=198	Quiet insects at 6 kHz	No	No + **Animal movements**	No	No	Slight wind
20150714_ 040100	https://www.ecosounds.org/listen/277009?start=14457	No	No	No	No	No	**Moderate wind**
20160629_ 081500	https://www.ecosounds.org/listen/354832?start=5344	No	No	No	No	No	**Moderate wind**
20160723_ 000300	https://www.ecosounds.org/listen/355016?start=170	Insects at 6 kHz	Yes	No	No	No	**Moderate air movement**
20151105_ 171300	https://www.ecosounds.org/listen/352805?start=13279	**Cicadas between 6 and 9 kHz**	No	Yellow-faced Honeyeater	No	No	No
20151105_ 084900	https://www.ecosounds.org/listen/352827?start=7388	**Cicadas between 6 and 9 kHz**	Moderately loud plane	Mistletoebird	No	No	Strong wind
20160514_ 131100	https://www.ecosounds.org/listen/354317?start=23093	No	**Loud plane**	No	No	No	**Slight wind**
20160702_ 074300	https://www.ecosounds.org/listen/354849?start=3429	No	No	Australasian Figbird	No	No	**Moderate wind**
20160129_ 002200	https://www.ecosounds.org/listen/353244?start=1317	Buzzing insect and crickets at 7 kHz	No	No	No	No	**Strong wind**
20150719_ 143300	https://www.ecosounds.org/listen/277105?start=6361	No	Moderately loud plane	Yes	No	No	**Moderate wind**

Cluster 25 – Gympie National Park

WIND (8175 minutes) The dominant sound sources are provided in bold text.

date_ time	Hyperlinks	Insects	Planes	Birds	Rain	Thunder	Wind
20150812_ 053400	https://www.ecosounds.org/listen/331508?start=20035	No	No	Eastern Yellow Robin	No	No	**Air movement**
20150628_ 013900	https://www.ecosounds.org/listen/331571?start=5934	No	No	No	**Very Light rain**	No	**Air movement**
20160430_ 025300	https://www.ecosounds.org/listen/354225?start=10372	Insects at 4 and 5 kHz	No	No	Very Light rain	No	Moderate wind
20150917_ 040200	https://www.ecosounds.org/listen/331919?start=14517	No	No	No	No	No	**Moderate wind**
20160405_ 125400	https://www.ecosounds.org/listen/353759?start=22091	Insects at 8 kHz	No	No	No	No	**Air movement**
20160123_ 043500	https://www.ecosounds.org/listen/353207?start=16497	**Insects between 4 and 6 kHz**	No	Laughing Kookaburra – distant	No	No	**Moderate wind**
20150910_ 191600	https://www.ecosounds.org/listen/331840?start=20659	Insects between 4 and 5 kHz	No	No	No	No	**Moderate to strong wind**
20150806_ 011300	https://www.ecosounds.org/listen/331453?start=4377	No	No	No	No	No	**Moderate wind**
20150709_ 041000	https://www.ecosounds.org/listen/277057?start=14998	No	**Loud plane**	No	Very Light rain	No	**Moderate wind**
20151005_ 111700	https://www.ecosounds.org/listen/333330?start=16268	**Cicadas between 5 and 9 kHz**	No	Yes	No	No	Moderate wind

Cluster 25 – Woondum National Park

WIND (16519 minutes) The dominant sound sources are provided in bold text.

date_ time	Hyperlinks	Insects	Planes	Birds	Rain	Thunder	Wind
20160629_ 095000	https://www.ecosounds.org/listen/354832?start=11044	No	No	Scarlet Honeyeater	No	No	**Strong wind**
20160509_ 230300	https://www.ecosounds.org/listen/354294?start=9934	Insects between 5 and 7 kHz	No	No	No	No	**Air movement**
20160413_ 212200	https://www.ecosounds.org/listen/354099?start=3871	Insects between 5 and 7 kHz	No	No	No	No	**Air movement**
20160328_ 115400	https://www.ecosounds.org/listen/353701?start=18489	No	No	Scarlet Honeyeater + Animal movement	No	No	**Air movement**
20160515_ 083900	https://www.ecosounds.org/listen/354321?start=6770	No	No	Eastern Whipbird	No	No	Air movement
20150714_ 063400	https://www.ecosounds.org/listen/277009?start=23637	No	No	Eastern Whipbird, Scarlet Honeyeater	No	No	Air movement
20150925_ 045300	https://www.ecosounds.org/listen/333258?start=17576	No	No	**Eastern Yellow Robin**	No	No	Moderate wind
20160617_ 213400	https://www.ecosounds.org/listen/354604?start=4582	Insects between 4 and 5 kHz	No	No + Animal movement	No	No	**Moderate wind**
20160410_ 075300	https://www.ecosounds.org/listen/353808?start=4022	No	No	**Yes**	No	No	**Air movement**
20160710_ 160300	https://www.ecosounds.org/listen/354963?start=18793	No	No	Lewin's Honeyeater	No	No	**Air movement**

Cluster 26 – Gympie National Park

INSECTS AND WIND (1778 minutes) The dominant sound sources are provided in bold text.
MID-FREQUENCY INSECTS

date_ time	Hyperlinks	Insects	Planes	Birds	Rain	Thunder	Wind
20151027_ 040100	https://www.ecosounds.org/listen/352709?start=14458	Insects at 4.8 kHz	No	No	No	No	**Moderate wind**
20151207_ 011200	https://www.ecosounds.org/listen/353072?start=4320	**Insects at 4.9 kHz**	No	No	No	No	No
20151206_ 002000	https://www.ecosounds.org/listen/353014?start=1198	**Insects at 5 kHz**	No	No	No	No	No
20151027_ 041200	https://www.ecosounds.org/listen/352709?start=15118	Insects between 4.5 and 5 kHz	No	No	No	No	**Moderate wind**
20160126_ 025000	https://www.ecosounds.org/listen/353257?start=10198	**Insects between 4 and 6 kHz**	No	No	No	No	Moderate wind
20150703_ 171000	https://www.ecosounds.org/listen/269849?start=13094	No	No	**Scarlet Honeyeater**	No	No	Moderate wind
20151227_ 012800	https://www.ecosounds.org/listen/353812?start=5275	**Insects above 4.8 kHz**	No	No	No	No	Moderate wind
20151112_ 235800	https://www.ecosounds.org/listen/352874?start=13229	**Insects between 3 and 6 kHz**	No	No	No	No	Moderate wind
20160114_ 031500	https://www.ecosounds.org/listen/354037?start=11697	**Insects between 4 and 6 kHz**	No	No	No	No	Slight wind
20160420_ 080900	https://www.ecosounds.org/listen/354150?start=4986	No	No	Lewin's Honeyeater, Scarlet Honeyeater	No	No	Moderate wind

Cluster 26 – Woondum National Park

INSECTS AND WIND (5179 minutes) The dominant sound sources are provided in bold text.
MID-FREQUENCY INSECTS

date_ time	Hyperlinks	Insects	Planes	Birds	Rain	Thunder	Wind
20160709_ 140500	https://www.ecosounds.org/listen/354904?start=1964	No	No	No	No	No	**Slight wind**
20160323_ 124400	https://www.ecosounds.org/listen/353682?start=21484	Insects between 7.8 and 8.5 kHz	No	**Scarlet Honeyeater**	No	No	Moderate wind
20160414_ 024600	https://www.ecosounds.org/listen/354127?start=9958	**Insects between 5 and 6 kHz and high frequency Cicada**	No	No	No	No	Moderate wind
20160112_ 033200	https://www.ecosounds.org/listen/354042?start=12716	Insects between 4 and 5.2 kHz	No	**Yes**	No	No	Moderate wind
20160330_ 130100	https://www.ecosounds.org/listen/353712?start=22508	No	No	**Distant bird**	No	No	Moderate wind
20160102_ 001200	https://www.ecosounds.org/listen/353934?start=715	**Insects between 4.8 and 6 kHz**	No	No	No	No	Moderate wind
20160430_ 114100	https://www.ecosounds.org/listen/354226?start=17687	Cicadas	No	No	No	No	**Slight wind**
20160401_ 014300	https://www.ecosounds.org/listen/353717?start=6175	**Insects between 5 and 6.2 kHz + Cicadas**	No	No	No	No	Moderate to stong wind
20160426_ 011300	https://www.ecosounds.org/listen/354182?start=4378	**Insects between 5 and 6 kHz**	No	No	No	No	Slight wind
20151207_ 024300	https://www.ecosounds.org/listen/353045?start=9778	Insects at 5 kHz	No	No	No	No	Moderate wind

Cluster 27 – Gympie National Park

INSECTS (11494 minutes) The dominant sound sources are provided in bold text.

date_ time	Hyperlinks	Insects	Planes	Birds	Rain	Thunder	Wind
20160130_ 180100	https://www.ecosounds.org/listen/353278?start=16159	No	No	No	**Heavy rain**	**Moderately loud thunder**	Strong wind
20151118_ 193300	https://www.ecosounds.org/listen/352885?start=21679	**Insects between 4 and 6 kHz**	Loud plane	No	No	No	No
20151219_ 133900	https://www.ecosounds.org/listen/353147?start=438	**Insects above 2.5 kHz**	Distant plane	White-throated Treecreeper, Rufous Whistler	No	No	No
20151116_ 013700	https://www.ecosounds.org/listen/352898?start=5818	**Insects between 4 and 5 and at 8.5 kHz**	No	No	No	No	No
20151106_ 192700	https://www.ecosounds.org/listen/352785?start=21320	**Insects between 4 and 6.5 kHz**	Loud plane	No	No	No	No
20151014_ 190200	https://www.ecosounds.org/listen/354709?start=19816	**Insects between 3.9 and 6 kHz and at 9.8 kHz**	Distant plane	No	No	No	No
20151027_ 181900	https://www.ecosounds.org/listen/352711?start=17242	**Insects above 1.8 kHz**	No	Eastern Koel	No	No	No
20160213_ 200600	https://www.ecosounds.org/listen/353384?start=23661	**Insects between 4 and 6 kHz**	No	No	No	No	Moderate wind
20151014_ 185500	https://www.ecosounds.org/listen/354709?start=19396	**Insects between 4 and 6 and at 9.8 kHz**	No	No	No	No	No
20160307_ 181900	https://www.ecosounds.org/listen/353507?start=17243	**Insects between 1.7 and 7 kHz**	No	Yes	No	No	No

Cluster 27 – Woondum National Park

INSECTS (4954 minutes) The dominant sound sources are provided in bold text.

date_ time	Hyperlinks	Insects	Planes	Birds	Rain	Thunder	Wind
20160111_ 141500	https://www.ecosounds.org/listen/354064?start=2597	**Insects above 5.5 kHz**	No	No	No	No	No
20151221_ 183600	https://www.ecosounds.org/listen/353829?start=18265	**Insects above 1.8 kHz**	No	Cicadabird	No	No	No
20160111_ 175100	https://www.ecosounds.org/listen/354064?start=15557	**Insects above 6 kHz**	Moderately loud plane	Yes	No	No	No
20151211_ 173800	https://www.ecosounds.org/listen/353087?start=14776	**Insects above 6 kHz**	Moderately loud plane	Yes	No	No	No
20151211_ 180500	https://www.ecosounds.org/listen/353087?start=16396	**Insects above 1.8 kHz**	Quiet plane	Cicadabird	No	No	No
20151224_ 144700	https://www.ecosounds.org/listen/353841?start=4519	**Insects above 6 kHz**	Distant plane	No	No	No	No
20151227_ 151400	https://www.ecosounds.org/listen/353875?start=6129	**Insects at 3 kHz and above 7 kHz**	No	Grey Shrike-thrush, Rufuous or Golden Whistler	No	No	No
20160113_ 180700	https://www.ecosounds.org/listen/354049?start=16515	**Insects between 6 and 9 kHz**	Moderate to loud plane	No	No	No	No
20151208_ 185800	https://www.ecosounds.org/listen/353074?start=19575	**Insects above 0.8 kHz**	No	Eastern Yellow Robin	No	No	No
20160303_ 184700	https://www.ecosounds.org/listen/354002?start=18921	**Insects above 4.8 kHz and at 2 and 2.6 kHz**	No	No	No	No	No

Cluster 28 – Gympie National Park

BIRDS AND/OR INSECTS OR PLANES (11158 minutes) - Inconsistent The dominant sound sources are provided in bold text.

date_ time	Hyperlinks	Insects	Planes	Birds	Rain	Thunder	Wind
20151123_ 095700	https://www.ecosounds.org/listen/352956?start=11468	Insects above 7 kHz	No	Yes	No	No	**Moderate wind**
20160519_ 134400	https://www.ecosounds.org/listen/354364?start=742	**Insects between 8 and 9 kHz**	No	**Lewin's Honeyeater**	No	No	No
20160507_ 134800	https://www.ecosounds.org/listen/354256?start=977	**Insects between 8 and 9 kHz**	No	**Lewin's Honeyeater**	No	No	No
20150715_ 131000	https://www.ecosounds.org/listen/276993?start=23046	No	Loud plane	**Pied Currawong, Lewin's Honeyeater**	No	No	No
20151103_ 104500	https://www.ecosounds.org/listen/352793?start=14350	Insects above 6.5 kHz	No	**White-throated Honeyeater, Lewin's Honeyeater**	No	No	No
20160525_ 165900	https://www.ecosounds.org/listen/354414?start=12406	No	No	**Scarlet Honeyeater or Rufous Whistler**	No	No	No
20160424_ 040300	https://www.ecosounds.org/listen/354166?start=14577	No	No	No	No	No	**Moderate wind**
20160504_ 134700	https://www.ecosounds.org/listen/354243?start=920	No	No	**Spotted Pardalote**	No	No	No
20160319_ 142200	https://www.ecosounds.org/listen/353587?start=3018	**Insects above 5 kHz**	No	White-throated Treecreeper	No	No	No
20160418_ 122100	https://www.ecosounds.org/listen/354123?start=20111	No	**Loud plane**	Mistletoebird	No	No	Moderate wind

Cluster 28 – Woondum National Park

BIRDS AND/OR INSECTS OR PLANES (3030 minutes) - Inconsistent The dominant sound sources are provided in bold text.

date_ time	Hyperlinks	Insects	Planes	Birds	Rain	Thunder	Wind
20160527_ 185400	https://www.ecosounds.org/listen/354479?start=19303	**Insects between 5 and 6.2 kHz**	**Loud plane-very loud**	No	No	No	No
20160115_ 133000	https://www.ecosounds.org/listen/354080?start=24243	**Insects above 3 kHz**	Moderately loud plane	No	No	No	No
20150807_ 143600	https://www.ecosounds.org/listen/331495?start=3862	No	**Moderately loud plane**	**White-throated Treecreeper**	No	No	No
20160329_ 120300	https://www.ecosounds.org/listen/353708?start=19027	**Cicada zizzt**	No	Grey Fantail	No	No	Moderate wind
20151014_ 144900	https://www.ecosounds.org/listen/354737?start=4639	Insects above 7 kHz and buzz	No	**White-throated Treecreeper**	No	No	Moderate wind
20151106_ 200200	https://www.ecosounds.org/listen/352812?start=23415	Insects between 4.8 and 7 kHz	**Loud plane**	Southern Boobook	No	No	No
20150924_ 094800	https://www.ecosounds.org/listen/333256?start=10925	No	No	**Scarlet Honeyeater**	No	No	Moderate wind
20151108_ 010300	https://www.ecosounds.org/listen/352816?start=3774	No	Loud plane	No	No	No	**Gusty wind**
20150626_ 210200	https://www.ecosounds.org/listen/331596?start=2671	Insects between 4 and 5 kHz	**Loud plane**	No	No	No	Moderate wind
20160522_ 112900	https://www.ecosounds.org/listen/354429?start=16981	No	**Loud plane- very loud**	Yes	No	No	Moderate wind

Cluster 29 – Gympie National Park

INSECTS (33867 minutes) The dominant sound sources are provided in bold text.
MID-FREQUENCY

date_ time	Hyperlinks	Insects	Planes	Birds	Rain	Thunder	Wind
20151116_ 201900	https://www.ecosounds.org/listen/352879?start=94	**Insects between 4 and 6 kHz**	No	No	No	No	No
20151224_ 224900	https://www.ecosounds.org/listen/353802?start=9089	**Insects between 4 and 6 kHz**	No	Southern Boobook	No	No	No
20151204_ 210800	https://www.ecosounds.org/listen/353005?start=3037	**Insects between 4 and 6 kHz**	No	No	No	No	No
20160224_ 230400	https://www.ecosounds.org/listen/353461?start=9991	**Insects between 4 and 6 kHz**	No	No	No	No	No
20160108_ 233500	https://www.ecosounds.org/listen/353992?start=11850	**Insects between 4 and 6 kHz**	No	No	No	No	No
20151210_ 224000	https://www.ecosounds.org/listen/353085?start=8555	**Insects between 4 and 7 kHz**	No	No	No	No	No
20160119_ 213500	https://www.ecosounds.org/listen/353169?start=4653	**Insects between 4 and 6 kHz**	No	No	No	No	No
20151208_ 021500	https://www.ecosounds.org/listen/353077?start=8096	**Insects between 3 and 4 and 5 and 6 kHz**	No	No	No	No	Moderate wind
20160422_ 191800	https://www.ecosounds.org/listen/354168?start=20780	**Insects between 4.6 and 6.2 kHz**	No	No	No	No	No
20160329_ 040900	https://www.ecosounds.org/listen/353676?start=14937	**Quiet insects between 4 and 6 kHz**	No	No	No	No	No

Cluster 29 – Woondum National Park

INSECTS (27796 minutes) The dominant sound sources are provided in bold text.
MID-FREQUENCY

date_ time	Hyperlinks	Insects	Planes	Birds	Rain	Thunder	Wind
20150916_ 222800	https://www.ecosounds.org/listen/331872?start=7830	**Insects between 5 and 6 kHz**	No	No	No	No	Distant wind
20160509_ 230800	https://www.ecosounds.org/listen/354294?start=10234	**Insects between 5 and 6 kHz**	Distant plane	No	No	No	Distant wind
20160709_ 184000	https://www.ecosounds.org/listen/354904?start=18464	Insects between 4 and 5 kHz	No	No	No	No	**Moderate wind**
20151108_ 213600	https://www.ecosounds.org/listen/352818?start=4654	**Insects between 5 and 6 kHz**	No	No	No	No	Distant wind
20151210_ 211900	https://www.ecosounds.org/listen/353057?start=3691	**Insects between 5 and 7 kHz**	No	No	No	No	Distant wind
20151122_ 235600	https://www.ecosounds.org/listen/352977?start=14087	**Insects between 5 and 7 kHz**	No	No	No	No	Distant wind
20160622_ 035100	https://www.ecosounds.org/listen/354689?start=13851	No	No	No	No	No	**Moderate wind**
20151223_ 001800	https://www.ecosounds.org/listen/353834?start=1075	Insects between 5 and 6 kHz	No	No	No	No	Distant wind
20151118_ 193700	https://www.ecosounds.org/listen/352937?start=21915	**Insects between 5 and 6.2 kHz**	No	No	No	No	No
20160327_ 183100	https://www.ecosounds.org/listen/353699?start=17777	**Insects between 5 and 7 kHz and at 2 kHz**	No	No	No	No	No

Cluster 30 – Gympie National Park

BIRDS and QUIET (11222 minutes) The dominant sound sources are provided in bold text.

date_ time	Hyperlinks	Insects	Planes	Birds	Rain	Thunder	Wind
20160420_ 160400	https://www.ecosounds.org/listen/354130?start=9136	Insects between 7 and 8 kHz	Distant plane	**Unknown, Rufous Fantail**	No	No	Slight wind
20160330_ 033400	https://www.ecosounds.org/listen/353703?start=12838	**Insects between 4 and 6 kHz**	No	No	No	No	Slight wind
20160518_ 015700	https://www.ecosounds.org/listen/354359?start=7016	No	No	No	No	No	No
20150813_ 163400	https://www.ecosounds.org/listen/331514?start=10940	No	No	Mistletoebird, Pied Currawong	No	No	**Moderate wind**
20160120_ 094000	https://www.ecosounds.org/listen/353177?start=10448	No	Distant plane	**White-throated Honeyeater, Lewin's Honeyeater**	No	No	Slight wind
20160412_ 074900	https://www.ecosounds.org/listen/354066?start=3787	No	No	**White-throated Honeyeater, Fan-tailed Cuckoo, Spectacled Monarch**	No	No	Slight wind
20160412_ 155300	https://www.ecosounds.org/listen/354094?start=8478	No	No	**Lewin's Honeyeater, White-throated Honeyeater**	No	No	Moderate wind
20160413_ 135400	https://www.ecosounds.org/listen/354073?start=1336	No	No	No	No	No	Slight wind
20160113_ 075400	https://www.ecosounds.org/listen/354011?start=4087	No	No	**White-throated Treecreeper, Brush cuckoo, White-throated Honeyeater**	No	No	Slight wind
20160702_ 191900	https://www.ecosounds.org/listen/354819?start=20842	No	No	No	No	No	Slight wind

Cluster 30 – Woondum National Park

BIRDS AND QUIET (7196 minutes) The dominant sound sources are provided in bold text.

date_ time	Hyperlinks	Insects	Planes	Birds	Rain	Thunder	Wind
20160329_ 163600	https://www.ecosounds.org/listen/353735?start=11059	Insect zizzt	Distant plane	Unknown, Rufous Fantail	No	No	Slight wind
20160409_ 045200	https://www.ecosounds.org/listen/353777?start=17517	**Insect zizzt**	No	**Southern Boobook**	No	No	Slight wind
20160323_ 125500	https://www.ecosounds.org/listen/353682?start=22144	No	Moderately loud plane	Rufous Fantail	No	No	Slight wind
20160524_ 101200	https://www.ecosounds.org/listen/354438?start=12349	No	Distant plane	No	No	No	Slight wind
20160506_ 154000	https://www.ecosounds.org/listen/354306?start=7695	**Insect zizzt**	No	No	No	No	Slight wind
20160530_ 070700	https://www.ecosounds.org/listen/354487?start=1270	No	Moderately loud plane	White-throated Treecreeper, Spotted Pardalote	No	No	Slight wind
20160418_ 102300	https://www.ecosounds.org/listen/354153?start=13029	No	No	**Mistletoebird**	No	No	Slight wind
20160420_ 151900	https://www.ecosounds.org/listen/354162?start=6434	No	No	Grey Fantail	No	No	Moderate wind
20160524_ 081500	https://www.ecosounds.org/listen/354438?start=5329	No	**Loud plane**	No	No	No	Moderate wind
20160513_ 090200	https://www.ecosounds.org/listen/354308?start=8153	No	Distant plane	Mistletoebird	No	No	Slight wind

Cluster 31 – Gympie National Park

QUIET (13150 minutes) The dominant sound sources are provided in bold text.

date_ time	Hyperlinks	Insects	Planes	Birds	Rain	Thunder	Wind
20150710_ 211600	https://www.ecosounds.org/listen/276956?start=3509	No	No	Animal movement	No	No	**Moderate wind**
20150905_ 034900	https://www.ecosounds.org/listen/331712?start=13737	No	No	No	No	No	Moderate wind
20160712_ 013400	https://www.ecosounds.org/listen/354940?start=5637	No	No	No	No	No	No
20160211_ 042900	https://www.ecosounds.org/listen/353373?start=16136	Insects between 4 and 5.5 kHz	No	Yes	No	No	No
20160301_ 043900	https://www.ecosounds.org/listen/353938?start=16738	Insects between 4 and 6 kHz + Cicada zizzt	No	No	No	No	No
20160421_ 100700	https://www.ecosounds.org/listen/354132?start=12067	No	No	Silvereye	No	No	No
20160613_ 212600	https://www.ecosounds.org/listen/354620?start=4110	No	Distant plane	No	No	No	No
20151009_ 225100	https://www.ecosounds.org/listen/333345?start=9210	Insects between 3.7 and 5 kHz	No	No	No	No	No
20160609_ 171300	https://www.ecosounds.org/listen/354559?start=13252	Insects between 5 and 6 kHz	No	Distant Pied Currawong	No	No	No
20150704_ 034200	https://www.ecosounds.org/listen/269824?start=13317	No	No	No	No	No	Moderate wind

Cluster 31 – Woondum National Park

QUIET (25367 minutes) The dominant sound sources are provided in bold text.

date_ time	Hyperlinks	Insects	Planes	Birds	Rain	Thunder	Wind
20160513_ 211200	https://www.ecosounds.org/listen/354311?start=3235	Insects at 5 kHz	No	No	No	No	No
20160630_ 123700	https://www.ecosounds.org/listen/354840?start=21062	Insects at 4.9 kHz	No	No	No	No	Slight wind
20160128_ 044000	https://www.ecosounds.org/listen/353238?start=16797	Insects between 5 and 6 kHz	Distant vehicle	No	No	No	No
20151129_ 040900	https://www.ecosounds.org/listen/352985?start=14935	Insects at 5.4 kHz + Cicada zizzt	No	Eastern Yellow Robin	No	No	No
20160304_ 041000	https://www.ecosounds.org/listen/353979?start=14997	Insect zizzt	No	No	No	No	No
20151121_ 114600	https://www.ecosounds.org/listen/352924?start=18007	Insects above 5 kHz	No	No	No	No	No
20160331_ 122900	https://www.ecosounds.org/listen/353714?start=20587	Insect zizzt	No	Scarlet Honeyeater, Lewin's Honeyeater	No	No	No
20160414_ 145300	https://www.ecosounds.org/listen/354105?start=4878	No	Distant plane	No	No	No	No
20151018_ 020900	https://www.ecosounds.org/listen/354747?start=7734	Insects between 4 and 5 kHz	No	Yes - nocturnal	No	No	No
20150730_ 103800	https://www.ecosounds.org/listen/277148?start=13926	No	No	Scarlet Honeyeater, Lewin's Honeyeater	No	No	No

Cluster 32 – Gympie National Park

CICADAS (9164 minutes) The dominant sound sources are provided in bold text.
HIGH FREQUENCY

date_ time	Hyperlinks	Insects	Planes	Birds	Rain	Thunder	Wind
20160326_ 140200	https://www.ecosounds.org/listen/353641?start=1819	**Cicadas above 7 kHz**	**Loud plane**	Lewin's Honeyeater	No	No	No
20160302_ 122300	https://www.ecosounds.org/listen/353971?start=20227	**Cicadas above 7 kHz**	No	Yes	No	No	Moderate to strong wind
20160226_ 161100	https://www.ecosounds.org/listen/353469?start=9564	**Cicadas above 7 kHz**	**Moderately loud plane**	White-throated Honeyeater	No	No	Slight wind
20151127_ 104800	https://www.ecosounds.org/listen/352948?start=14529	**Cicadas above 7 kHz**	**Loud plane**	Lewin's Honeyeater, Torresian Crow, White-throated Treecreeper	No	No	Slight wind
20151119_ 132800	https://www.ecosounds.org/listen/352888?start=24130	**Cicadas above 4 kHz**	Distant plane	Cicadabird	No	No	No
20151118_ 160700	https://www.ecosounds.org/listen/352885?start=9319	**Cicadas above 7 kHz**	Distant plane	Eastern Yellow Robin	No	No	Slight wind
20151128_ 133000	https://www.ecosounds.org/listen/352952?start=24246	**Cicadas above 4 kHz**	No	Cicadabird, White-throated Treecreeper	No	No	Slight wind
20160305_ 143900	https://www.ecosounds.org/listen/353957?start=4041	**Cicadas above 3 kHz**	No	No	No	No	No
20151125_ 123300	https://www.ecosounds.org/listen/352943?start=20829	**Cicadas above 2 kHz**	No	White-throated Honeyeater	No	No	No
20151218_ 104300	https://www.ecosounds.org/listen/353144?start=14227	**Cicadas above 6 kHz**	**Distant plane**	Torresian Crow	No	No	Moderate wind

Cluster 32 – Woondum National Park

CICADAS (4637 minutes) The dominant sound sources are provided in bold text.
HIGH FREQUENCY

date_ time	Hyperlinks	Insects	Planes	Birds	Rain	Thunder	Wind
20151214_ 152600	https://www.ecosounds.org/listen/353131?start=6864	**Cicadas above 6 kHz**	No	Little Shrike-thrush	No	No	Moderate wind
20160215_ 120000	https://www.ecosounds.org/listen/353397?start=18853	**Cicadas above 5 kHz**	No	Scarlet Honeyeater, Lewin's Honeyeater	No	No	Moderate wind
20160102_ 171000	https://www.ecosounds.org/listen/353916?start=13097	**Cicadas between 5 and 9 kHz**	No	Lewin's Honeyeater, White-throated Treecreeper	No	No	Moderate to strong wind
20160217_ 165300	https://www.ecosounds.org/listen/353405?start=12077	**Cicadas and insects between 2.9 and 9 kHz**	Distant plane	No	No	No	No
20151122_ 120000	https://www.ecosounds.org/listen/352950?start=18844	**Cicadas above 6 kHz**	No	King Parrot, Eastern Yellow Robin, White-throated Treecreeper	No	No	Slight wind
20151205_ 145400	https://www.ecosounds.org/listen/353064?start=4935	**Cicadas above 6 kHz**	No	Cicadabird, Mistletoebird	No	No	Moderate wind
20160114_ 222000	https://www.ecosounds.org/listen/354053?start=7335	**Insects between 4 and 6 kHz and above 8.5 kHz**	No	No	No	No	No
20151221_ 105400	https://www.ecosounds.org/listen/353850?start=14891	**Cicadas above 2.5 kHz**	Distant plane	Olive-backed Oriole	No	No	Strong wind
20160318_ 145600	https://www.ecosounds.org/listen/353634?start=5056	**Cicadas above 2 kHz**	Distant plane	No	No	No	Moderate wind
20160109_ 131400	https://www.ecosounds.org/listen/354031?start=23285	**Insects above 7 kHz**	No	Yes	No	No	Slight wind

Cluster 33 – Gympie National Park

BIRDS (14893 minutes) The dominant sound sources are provided in bold text.

date_ time	Hyperlinks	Insects	Planes	Birds	Rain	Thunder	Wind
20160402_ 061000	https://www.ecosounds.org/listen/353693?start=22196	No	No	**Brown Cuckoo-dove, White-throated Treecreeper, Eastern Whipbird**	No	No	No
20160403_ 104000	https://www.ecosounds.org/listen/353694?start=14047	Insect buzzing	Distant plane	**White-throated Honeyeater**	No	No	No
20150731_ 124800	https://www.ecosounds.org/listen/277168?start=21727	No	No	**Eastern Whipbird, Scarlet Honeyeater, Spotted Pardalote, Lewin's Honeyeater**	No	No	No
20160128_ 075300	https://www.ecosounds.org/listen/353290?start=4026	No	Distant plane	**White-throated Treecreeper, Eastern Yellow Robin, White-throated Honeyeater**	No	No	Slight wind
20160509_ 112200	https://www.ecosounds.org/listen/354349?start=16570	No	**Loud plane**	**White-throated Treecreeper**	No	No	No
20160320_ 205800	https://www.ecosounds.org/listen/353635?start=9125	**Insects between 4 and 7 kHz**	No	No	No	No	No
20150804_ 152300	https://www.ecosounds.org/listen/331456?start=6681	Insects between 7 and 8 kHz	Distant plane	**Rufous Whistler, Scarlet Honeyeater,**	No	No	Slight wind
20160123_ 101900	https://www.ecosounds.org/listen/353186?start=12788	Insects (distant) above 6 kHz	No	**White-throated Treecreeper, Mistletoebird**	No	No	No
20160418_ 101800	https://www.ecosounds.org/listen/354123?start=12731	No	No	**Grey Fantail, White-throated Gerygone**	No	No	Slight wind
20160628_ 165600	https://www.ecosounds.org/listen/354808?start=12259	No	No	**White-throated Honeyeater**	No	No	Slight wind

Cluster 33 – Woondum National Park

BIRDS (3930 minutes) The dominant sound sources are provided in bold text.

date_ time	Hyperlinks	Insects	Planes	Birds	Rain	Thunder	Wind
20150814_ 135900	https://www.ecosounds.org/listen/331548?start=1639	No	**Loud plane**	**Yellow-faced Honeyeater, Scarlet Honeyeater**	No	No	**Moderate wind**
20160320_ 112100	https://www.ecosounds.org/listen/353618?start=16506	No	No	Shining Bronze-cuckoo	No	No	**Moderate wind**
20150923_ 123700	https://www.ecosounds.org/listen/333252?start=21065	No	No	**Rufous Whistler, Golden Whistler**	No	No	Slight wind
20150831_ 140800	https://www.ecosounds.org/listen/331729?start=2187	No	Moderately loud plane	**Scarlet Honeyeater**	No	No	Moderate wind
20160119_ 201500	https://www.ecosounds.org/listen/353203?start=24192	**Insects between 4 and 7 kHz and above 9 kHz**	Moderately loud plane	**Yes**	No	No	No
20160322_ 193900	https://www.ecosounds.org/listen/353652?start=22036	**Insects between 4 and 7 kHz**	Moderately loud plane	No	No	No	Slight wind
20150909_ 113400	https://www.ecosounds.org/listen/331866?start=17286	No	No	**Scarlet Honeyeater, Australasian Figbird**	No	No	Slight wind
20150716_ 170300	https://www.ecosounds.org/listen/277024?start=12677	No	**Loud plane**	**Grey Fantail**	No	No	Slight wind
20160321_ 011800	https://www.ecosounds.org/listen/353645?start=4678	Insects between 6 and 7 kHz	Distant plane	No	No	No	**Moderate wind**
20150808_ 115400	https://www.ecosounds.org/listen/331497?start=18489	No	Moderately loud plane	**Scarlet Honeyeater, Yellow-faced Honeyeater**	No	No	No

Cluster 34 – Gympie National Park

CICADAS (3814 minutes) The dominant sound sources are provided in bold text.

date_ time	Hyperlinks	Insects	Planes	Birds	Rain	Thunder	Wind
20160106_ 092300	https://www.ecosounds.org/listen/353891?start=9427	**Very loud insects above 2 kHz**	No	Yes	No	No	No
20151102_ 171300	https://www.ecosounds.org/listen/352790?start=13285	**Insects above 6 kHz**	Distant plane	Yes	No	No	No
20160116_ 045600	https://www.ecosounds.org/listen/354025?start=17758	**Insects between 2.5 and 11 kHz**	Distant plane	Lewin's Honeyeater	No	No	No
20160326_ 134800	https://www.ecosounds.org/listen/353641?start=979	**Insects between 2.8 and 8 kHz**	No	White-throated Gerygone	No	No	No
20151201_ 041700	https://www.ecosounds.org/listen/352990?start=15418	**Insects between 2.5 and 5.5 kHz**	No	Spectacled Monarch, White-throated Honeyeater, Eastern Yellow Robin, White-throated Treecreeper	No	No	**Moderate wind**
20160218_ 111600	https://www.ecosounds.org/listen/353460?start=16210	**Insects between 2.5 and 11 kHz**	No	No	No	No	No
20151123_ 170100	https://www.ecosounds.org/listen/352960?start=12559	**Insects above 7 kHz**	No	White-throated Honeyeater, Mistletoebird	No	No	Moderate wind
20160102_ 125400	https://www.ecosounds.org/listen/353901?start=22086	**Insects above 2.8 kHz**	No	No	No	No	No
20151224_ 081100	https://www.ecosounds.org/listen/353826?start=5108	**Insects from 2 to 7 and 8 to 9 kHz**	No	No	No	No	No
20151221_ 042500	https://www.ecosounds.org/listen/353790?start=15898	**Insects from 2.5 to 9 kHz**	No	Brush Cuckoo	No	No	No

Cluster 34 – Woondum National Park

CICADAS (5449 minutes) The dominant sound sources are provided in bold text.

date_ time	Hyperlinks	Insects	Planes	Birds	Rain	Thunder	Wind
20151220_ 100600	https://www.ecosounds.org/listen/353180?start=12001	**Insects from 2 to 11 kHz**	No	No	No	No	No
20151204_ 123800	https://www.ecosounds.org/listen/353036?start=21129	**Insects above 3 kHz**	Very distant plane	**White-throated Treecreeper, Grey Shrike-thrush**	No	No	No
20160208_ 120900	https://www.ecosounds.org/listen/353367?start=19390	**Loud insects above 2.5 kHz**	No	No	No	No	No
20160120_ 123500	https://www.ecosounds.org/listen/353231?start=20944	**Insects between 2.5 and 10 kHz and Buzzing insect**	No	No	No	No	No
20160216_ 172400	https://www.ecosounds.org/listen/353427?start=13933	**Insects above 2 kHz**	Moderately loud plane	No	No	No	No
20160219_ 103000	https://www.ecosounds.org/listen/353410?start=13448	**Very loud insects above 2 kHz**	No	No	No	No	No
20160310_ 123300	https://www.ecosounds.org/listen/353547?start=20825	**Insects between 2.8 and 8 kHz**	No	No	No	No	No
20160130_ 091700	https://www.ecosounds.org/listen/353274?start=9064	**Insects above 2.8 kHz**	**Loud plane**	No	No	No	No
20160311_ 145100	https://www.ecosounds.org/listen/353583?start=4752	**Insects above 2.5 kHz**	**Loud plane**	No	No	No	No
20160318_ 145200	https://www.ecosounds.org/listen/353634?start=4816	**Insects between 2.5 and 6 and above 8 kHz**	No	Yes	No	No	Moderate wind

Cluster 35 – Gympie National Park

QUIET (17113 minutes) The dominant sound sources are provided in bold text.

date_ time	Hyperlinks	Insects	Planes	Birds	Rain	Thunder	Wind
20150916_ 045100	https://www.ecosounds.org/listen/331921?start=17458	No	Moderate to loud plane	Eastern Yellow Robin	No	No	No
20150911_ 025200	https://www.ecosounds.org/listen/331823?start=10317	No	No	No	No	No	No
20150929_ 035700	https://www.ecosounds.org/listen/333274?start=14217	No	No	No	No	No	No
20160603_ 015300	https://www.ecosounds.org/listen/354507?start=6778	No	No	No	No	No	No
20150829_ 052500	https://www.ecosounds.org/listen/331773?start=19497	No	No	Eastern Yellow Robin	No	No	No
20160614_ 024500	https://www.ecosounds.org/listen/354629?start=9897	Insects between 4 and 5 kHz	No	No	No	No	No
20160520_ 210200	https://www.ecosounds.org/listen/354366?start=2675	Insects at 5 kHz	No	No	No	No	No
20150720_ 031200	https://www.ecosounds.org/listen/277063?start=11518	No	No	No	No	No	Moderate wind
20160411_ 160300	https://www.ecosounds.org/listen/354063?start=9085	Insects at 1.8 and 8 kHz	Very distant plane	Distant birds	No	No	No
20160409_ 102000	https://www.ecosounds.org/listen/353776?start=12847	Insects (very quiet) above 6 kHz	No	Grey Fantail	No	No	No

Cluster 35 – Woondum National Park

QUIET (50457 minutes) The dominant sound sources are provided in bold text.

date_ time	Hyperlinks	Insects	Planes	Birds	Rain	Thunder	Wind
20160508_ 224900	https://www.ecosounds.org/listen/354290?start=5298	Insects (quiet) between 4 and 6 kHz	Very distant plane	No	No	No	No
20160324_ 020400	https://www.ecosounds.org/listen/353657?start=7438	Insects between 5 and 6.2 kHz	No	No	No	No	No
20160710_ 123400	https://www.ecosounds.org/listen/354963?start=6253	No	No	No	No	No	Moderate wind
20160515_ 184700	https://www.ecosounds.org/listen/354373?start=20084	Insects between 4.8 and 6 kHz	No	No	No	No	No
20160412_ 034600	https://www.ecosounds.org/listen/354096?start=13557	Insects at 5.1 kHz	Distant plane	No	No	No	No
20160516_ 232000	https://www.ecosounds.org/listen/354378?start=10956	No	No	No	No	No	No
20160517_ 020500	https://www.ecosounds.org/listen/354380?start=7498	No	No	No	No	No	No
20160225_ 043300	https://www.ecosounds.org/listen/353515?start=16375	No	Distant plane	No	No	No	No
20160519_ 085100	https://www.ecosounds.org/listen/354392?start=7508	No	Distant plane	Yes	No	No	No
20160227_ 020000	https://www.ecosounds.org/listen/353523?start=7197	Insects (quiet) between 4 and 7 kHz	No	No	No	No	No

Cluster 36 – Gympie National Park

QUIET AND/OR PLANES (8258 minutes) - Inconsistent The dominant sound sources are provided in bold text.

date_ time	Hyperlinks	Insects	Planes	Birds	Rain	Thunder	Wind
20151121_ 034400	https://www.ecosounds.org/listen/352920?start=13437	No	No	No	No	No	**Slight wind**
20160617_ 061000	https://www.ecosounds.org/listen/354643?start=22198	No	No	Distant Laughing Kookaburra	No	No	Slight wind
20160326_ 043100	https://www.ecosounds.org/listen/353637?start=16258	**Insects (quiet) at 5 kHz**	No	No	No	No	Slight wind
20150727_ 012500	https://www.ecosounds.org/listen/277167?start=5097	Insects at 5 kHz	No	No	No	No	**Slight wind**
20160418_ 095300	https://www.ecosounds.org/listen/354123?start=11231	No	Distant plane	Distant birds	No	No	Slight wind
20150726_ 211100	https://www.ecosounds.org/listen/277120?start=3504	Insects (quiet) at 5 kHz	No	No	No	No	Slight wind
20160504_ 235300	https://www.ecosounds.org/listen/354246?start=12932	Insects (quiet) between 4.5 and 5 kHz	Distant plane	No	No	No	No
20160427_ 225500	https://www.ecosounds.org/listen/354232?start=9448	**Insects (quiet) from 3 to 5 kHz**	Distant plane	No	No	No	No
20160626_ 050500	https://www.ecosounds.org/listen/354654?start=18291	No	No	No	No	No	No
20150629_ 185900	https://www.ecosounds.org/listen/269847?start=19643	No	**Loud plane**	No	No	No	No

Cluster 36 – Woondum National Park

QUIET AND/OR PLANES (4022 minutes) - Inconsistent The dominant sound sources are provided in bold text.

date_ time	Hyperlinks	Insects	Planes	Birds	Rain	Thunder	Wind
20150715_ 172300	https://www.ecosounds.org/listen/277026?start=13874	No	**Loud plane**	Eastern Whipbird	No	No	No
20160609_ 021000	https://www.ecosounds.org/listen/354529?start=7790	No	**Loud plane**	No	No	No	Slight wind
20150913_ 143900	https://www.ecosounds.org/listen/331863?start=3131	No	Distant plane	**Eastern Yellow Robin (piping)**	No	No	Slight wind
20160518_ 230200	https://www.ecosounds.org/listen/354387?start=9865	No	**Loud plane**	No	No	No	Slight wind
20160513_ 053500	https://www.ecosounds.org/listen/354307?start=20094	No	Loud plane	Distant birds	No	No	Slight wind
20150918_ 183000	https://www.ecosounds.org/listen/331888?start=17894	Insects between 4 and 5.3 kHz	**Loud plane**	No	No	No	Slight wind
20151020_ 190700	https://www.ecosounds.org/listen/354802?start=20115	Insects between 4.5 and 6 kHz	**Loud plane**	No	No	No	No
20150916_ 215400	https://www.ecosounds.org/listen/331872?start=5790	Insects between 4.5 and 6 kHz	**Moderately loud plane**	No	No	No	No
20160417_ 120900	https://www.ecosounds.org/listen/354116?start=19388	No	No	**White-throated Treecreeper**	Very light rain	No	Slight wind
20150706_ 142100	https://www.ecosounds.org/listen/276971?start=22887	No	**Loud plane**	No	No	No	Moderate wind

Cluster 37 – Gympie National Park

BIRDS - MORNING CHORUS (17011 minutes) The dominant sound sources are provided in bold text.

date_ time	Hyperlinks	Insects	Planes	Birds	Rain	Thunder	Wind
20150913_071000	https://www.ecosounds.org/listen/331848?start=1449	No	No	**Leaden, Eastern Whipbird, Scarlet Honeyeater, White-throated Treecreeper**	No	No	No
20160721_152000	https://www.ecosounds.org/listen/354984?start=6469	Insects between 7 and 8 kHz	Moderately loud plane	**Scarlet Honeyeater, Lewin's Honeyeater,**	No	No	No
20151110_044400	https://www.ecosounds.org/listen/352852?start=17038	No	No	**Russet-tailed Thrush, Leaden Flycatcher, Mistletoebird, White-throated Honeyeater, Lewin's Honeyeater, Eastern Whipbird**	No	No	Moderate wind
20160223_063300	https://www.ecosounds.org/listen/353455?start=23578	Cicadas (quiet) above 6 kHz	No	**Fan-tailed Cuckoo, Scarlet Honeyeater, Wonga Pigeon, Leaden Flycatcher, White-throated Honeyeater, Eastern Whipbird**	No	No	Slight wind
20160526_080300	https://www.ecosounds.org/listen/354419?start=4614	No	No	**Torresian Crow, Eastern Whipbird**	No	No	No
20151001_085800	https://www.ecosounds.org/listen/333284?start=7930	Insects above 6 kHz	No	**Mistletoebird, Eastern Whipbird,**	No	No	**Moderate wind**
20150924_053100	https://www.ecosounds.org/listen/333227?start=19857	No	Distant plane	**Eastern Whipbird, Mistletoebird, Scarlet Honeyeater, Rufous Whistler, Leaden Flycatcher, White-throated Treecreeper**	No	No	Moderate wind
20160410_094000	https://www.ecosounds.org/listen/353758?start=10449	No	No	**Mistletoebird, Lewin's Honeyeater, White-throated Gerygone**	No	No	Moderate wind
20160323_090500	https://www.ecosounds.org/listen/353627?start=8346	No	No	**Torresian Crow, Lewin's Honeyeater, unknown**	No	No	Moderate wind
20160720_075900	https://www.ecosounds.org/listen/354980?start=4369	No	Distant plane	**Rose Robin, Lewin's Honeyeater, Yellow-faced Honeyeater, Scarlet Honeyeater**	No	No	Moderate wind

Cluster 37 – Woondum National Park

BIRDS - MORNING CHORUS (14301 minutes) The dominant sound sources are provided in bold text.

date_ time	Hyperlinks	Insects	Planes	Birds	Rain	Thunder	Wind
20151106_ 045900	https://www.ecosounds.org/listen/352808?start=17938	No	No	**White-throated Treecreeper, Yellow-faced Honeyeater, Rufous Whistler, Variegated Fairywren, Golden Whistler**	No	No	Slight wind
20150916_ 052900	https://www.ecosounds.org/listen/331873?start=19736	No		**Scarlet Honeyeater, Pied Currawong, White-throated Treecreeper**	No	No	Slight wind
20151001_ 085000	https://www.ecosounds.org/listen/333313?start=7444	No	No	**Rufous Whistler, Scarlet Honeyeater, Fan-tailed Cuckoo, Mistletoebird, Yellow-faced Honeyeater**	No	No	Slight wind
20160220_ 064700	https://www.ecosounds.org/listen/353415?start=64	Insects at 3 kHz	No	**Mistletoebird, Scarlet Honeyeater, Silvereye,**	No	No	Slight wind
20150907_ 111000	https://www.ecosounds.org/listen/331835?start=15851	No	Moderatel y loud plane	**Scarlet Honeyeater, White-throated Treecreeper, Yellow-faced Honeyeater, Eastern Yellow Robin**	No	No	Slight wind
20150828_ 100200	https://www.ecosounds.org/listen/331800?start=11767	No	Moderatel y loud plane	**Golden Whistler, Scarlet Honeyeater, Lewin's Honeyeater**	No	No	Slight wind
20151010_ 074100	https://www.ecosounds.org/listen/333378?start=3309	Cicadas between 5 and 9 kHz	No	**Scarlet Honeyeater, Golden Whistler, Yellow-faced Honeyeater**	No	No	Slight wind
20150825_ 082800	https://www.ecosounds.org/listen/331787?start=6125	No	No	**Golden Whistler, Scarlet Honeyeater**	No	No	Slight wind
20150831_ 073400	https://www.ecosounds.org/listen/331746?start=2894	No	No	**Scarlet Honeyeater, White-throated Treecreeper,**	No	No	Slight wind
20150809_ 060600	https://www.ecosounds.org/listen/331500?start=21957	No	No	**Variegated Fairywren, Yellow-faced Honeyeater, Eastern Whipbird**	No	No	Slight wind

Cluster 38 – Gympie National Park

QUIET (3835 minutes) The dominant sound sources are provided in bold text.

date_ time	Hyperlinks	Insects	Planes	Birds	Rain	Thunder	Wind
20150721_ 005900	https://www.ecosounds.org/listen/277116?start=3537	No	No	No	**Very light rain**	No	Moderate wind
20160111_ 033100	https://www.ecosounds.org/listen/354030?start=12657	**Quiet insects between 3 and 6 kHz**	No	No	No	No	No
20150625_ 034600	https://www.ecosounds.org/listen/331567?start=13555	No	No	No	**Very light rain**	No	Slight wind
20150713_ 193800	https://www.ecosounds.org/listen/277040?start=21983	No	Very distant plane	No	No	No	No
20160405_ 041500	https://www.ecosounds.org/listen/353736?start=15298	**Quiet insects between 4 and 5 kHz**	No	No	Very light rain	No	No
20160103_ 032900	https://www.ecosounds.org/listen/353879?start=12536	Insects between 4 and 6 kHz	Very distant plane	Yes – distant + Animal movement	No	No	No
20150821_ 034900	https://www.ecosounds.org/listen/331654?start=13737	No	No	No	**Very light rain**	No	Moderate wind
20151105_ 035100	https://www.ecosounds.org/listen/352800?start=13857	Insects (quiet) between 5 and 6 kHz	No	Yes	No	No	Moderate wind
20150723_ 034900	https://www.ecosounds.org/listen/277115?start=13737	No	No	No	**Very light rain**	No	Moderate wind
20150808_ 014700	https://www.ecosounds.org/listen/331480?start=6418	No	No	Animal movement	No	No	Slight wind

Cluster 38 – Woondum National Park

QUIET (11016 minutes) The dominant sound sources are provided in bold text.

date_ time	Hyperlinks	Insects	Planes	Birds	Rain	Thunder	Wind
20160513_ 175800	https://www.ecosounds.org/listen/354309?start=15954	Insects (quiet) between 5 and 6 kHz	No	No	No	No	No
20160116_ 124200	https://www.ecosounds.org/listen/353190?start=9068	No	No	**Yes**	No	No	No
20160508_ 052800	https://www.ecosounds.org/listen/354310?start=19677	**Cicada zizzt (quiet) between 6 and 9 kHz**	No	No	Very light rain	No	Slight wind
20160113_ 034300	https://www.ecosounds.org/listen/354044?start=13378	**Insects between 3.8 and 5.2 kHz**	Moderately loud plane	Animal movements	No	No	No
20160313_ 010400	https://www.ecosounds.org/listen/353561?start=3836	No	No	No	No	No	Slight wind
20150625_ 004500	https://www.ecosounds.org/listen/331585?start=2698	No	No	No	**Very light rain**	No	Slight wind
20160214_ 041200	https://www.ecosounds.org/listen/353416?start=15116	No	No	**Yes**	No	No	Slight wind
20160519_ 055500	https://www.ecosounds.org/listen/354413?start=21296	No	Distant plane	**Distant birds**	Very light rain	No	No
20160506_ 053800	https://www.ecosounds.org/listen/354281?start=20275	No	Distant plane	No	**Very light rain**	No	Slight wind
20160603_ 104600	https://www.ecosounds.org/listen/354501?start=14407	No	No	No	No	No	Slight wind

Cluster 39 - Gympie National Park

BIRDS AND PLANES (11897 minutes) The dominant sound sources are provided in bold text.

date_ time	Hyperlinks	Insects	Planes	Birds	Rain	Thunder	Wind
20150807_ 061400	https://www.ecosounds.org/listen/331481?start=22437	No	No	**Torresian Crow, White-throated Treecreeper, Eastern Whipbird, Scarlet Honeyeater, White-throated Honeyeater**	No	No	No
20151120_ 125400	https://www.ecosounds.org/listen/352891?start=22090	Insects (quiet) above 7 kHz	Very distant plane	**Rufous Whistler, White-throated Honeyeater**	No	No	Moderate wind
20160720_ 160300	https://www.ecosounds.org/listen/354981?start=9045	Insects between 7 and 8 kHz	Loud plane	**Lewin's Honeyeater, Scarlet Honeyeater, Grey Fantail, Spotted Pardalote**	No	No	No
20150826_ 144600	https://www.ecosounds.org/listen/331781?start=4452	No	Loud plane	**Scarlet Honeyeater**	No	No	**Moderat e wind**
20160415_ 091400	https://www.ecosounds.org/listen/354078?start=8890	No	**Loud plane**	**Spangled Drongo, Spectacled Monarch**	No	No	Moderate wind
20150817_ 142700	https://www.ecosounds.org/listen/331613?start=3322	Quiet insects between 7 and 8 kHz	**Loud plane**	**Scarlet Honeyeater**	No	No	Slight wind
20160329_ 094300	https://www.ecosounds.org/listen/353677?start=10624	No	**Loud plane**	**Fan-tailed Cuckoo, Rufous Whistler, White-throated Gerygone**	No	No	Slight wind
20160512_ 060200	https://www.ecosounds.org/listen/354358?start=21717	No	Quiet plane	**Eastern Yellow Robin, White-throated Honeyeater, Lewin's Honeyeater**	No	No	Slight wind
20160106_ 183500	https://www.ecosounds.org/listen/353892?start=18198	**Cicadas above 3.2 kHz**	Quiet plane	Lewin's Honeyeater, Rainbow Lorikeet, Laughing Kookaburra	No	No	No
20160703_ 164600	https://www.ecosounds.org/listen/354878?start=17610	No	Moderately loud plane	**Scarlet Honeyeater, Torresian Crow, Yellow-faced Honeyeater, White-throated Treecreeper**	No	No	No

Cluster 39 - Woondum National Park

BIRDS AND PLANES (1938 minutes) The dominant sound sources are provided in bold text.

date_ time	Hyperlinks	Insects	Planes	Birds	Rain	Thunder	Wind
20151020_ 124000	https://www.ecosounds.org/listen/354783?start=21246	Insects above 6 kHz	**Loud plane**	**Rufous Whistler, Yellow-faced Honeyeater**	No	No	No
20150822_ 120100	https://www.ecosounds.org/listen/331662?start=18906	No	**Loud plane**	**Scarlet Honeyeater**	**Light rain**	No	Slight wind
20151119_ 053300	https://www.ecosounds.org/listen/352942?start=19975	No	**??**	**Golden Whistler, Cicadabird**	No	No	No
20151007_ 102600	https://www.ecosounds.org/listen/333366?start=13210	Insects between 6 and 9 kHz	**Loud plane**	**Rufous Whistler, Yellow-faced Honeyeater, Scarlet Honeyeater**	No	No	Slight wind
20150801_ 072800	https://www.ecosounds.org/listen/277157?start=2527	No	**Loud plane**	**Eastern Whipbird, Scarlet Honeyeater, Yellow-faced Honeyeater, Shining Bronze Cuckoo**	No	No	Slight wind
20160628_ 192700	https://www.ecosounds.org/listen/354831?start=21317	No	Moderately loud plane	No	No	No	**Microphone problem**
20160702_ 084300	https://www.ecosounds.org/listen/354849?start=7029	No	No	Yes	No	No	**Microphone problem**
20150902_ 130300	https://www.ecosounds.org/listen/331733?start=22629	No	No	**White-throated Treecreeper, Scarlet Honeyeater**	No	No	Slight wind
20151229_ 082200	https://www.ecosounds.org/listen/353823?start=5768	No	No	**Grey Shrike-thrush, Brown Cuckoo-dove**	No	No	**Strong wind**
20150809_ 121500	https://www.ecosounds.org/listen/331498?start=19748	No	**Loud plane**	**Scarlet Honeyeater**	No	No	Slight wind

Cluster 40 - Gympie National Park

WIND AND/OR BIRDS OR INSECTS (8594 minutes) - Inconsistent The dominant sound sources are provided in bold text.

date_ time	Hyperlinks	Insects	Planes	Birds	Rain	Thunder	Wind
20150820_ 133500	https://www.ecosounds.org/listen/331652?start=199	No	No	Scarlet Honeyeater	No	No	**Strong wind**
20150904_ 070700	https://www.ecosounds.org/listen/331710?start=1268	No	No	**White-throated Treecreeper, Mistletoebird, Golden Whistler, Spectacled Monarch**	No	No	**Strong wind**
20150629_ 080700	https://www.ecosounds.org/listen/269845?start=4871	No	No	**Olive-backed Oriole, Grey Fantail, Lewin's Honeyeater**	No	No	**Moderate wind**
20150813_ 091000	https://www.ecosounds.org/listen/331538?start=8648	No	Moderately loud plane	Scarlet Honeyeater	No	No	**Strong wind**
20160622_ 070200	https://www.ecosounds.org/listen/354642?start=950	No	**Loud plane**	Scarlet Honeyeater, Lewin's Honeyeater	No	No	**Strong wind**
20160612_ 122100	https://www.ecosounds.org/listen/354602?start=4778	No	Moderately loud plane	White-throated Honeyeater, White-throated Treecreeper	No	No	**Strong wind**
20160214_ 064800	https://www.ecosounds.org/listen/353361?start=129	**Cicadas between 6 and 9 kHz**	No	**Wonga Pigeon, White-throated Honeyeater, Australasian Figbird, Rainbow Lorikeet**	No	No	Moderate wind
20160317_ 055500	https://www.ecosounds.org/listen/353573?start=21297	Insects between 6 and 9 kHz	No	Eastern Whipbird, White-throated Honeyeater,	No	No	**Moderate wind**
20150628_ 103200	https://www.ecosounds.org/listen/331576?start=13561	No	No Motorbikes	White-throated Honeyeater, Scarlet Honeyeater, Torresian Crow	No	No	**Strong wind**
20160606_ 133500	https://www.ecosounds.org/listen/354572?start=166	No	No	Yes	No	No	**Strong wind**

Cluster 40 - Woondum National Park

WIND AND/OR BIRDS OR INSECTS BIRDS (11039 minutes) - Inconsistent The dominant sound sources are provided in bold text.

date_ time	Hyperlinks	Insects	Planes	Birds	Rain	Thunder	Wind
20151114_ 070500	https://www.ecosounds.org/listen/352866?start=1148	Insects between 5 and 9 kHz	**Loud plane**	Red-browed Finch, Mistletoebird	No	No	No
20160527_ 005500	https://www.ecosounds.org/listen/354452?start=3288	No	Moderately loud plane	No	No	No	**Very strong wind**
20150928_ 065800	https://www.ecosounds.org/listen/333301?start=733	No	**Loud plane**	Rufous Whistler, Mistletoebird,	No	No	Moderate wind
20160308_ 151300	https://www.ecosounds.org/listen/353565?start=6070	No	No	No	No	No	**Very strong wind**
20151022_ 120500	https://www.ecosounds.org/listen/352718?start=19147	**Cicadas between 6 and 9 kHz**	No	Golden Whistler	No	No	**Strong wind**
20151103_ 100500	https://www.ecosounds.org/listen/352797?start=11945	**Cicadas between 6 and 9 kHz**	No	Scarlet Honeyeater, Olive-backed Oriole	No	No	**Moderate wind**
20150725_ 101300	https://www.ecosounds.org/listen/277102?start=12430	No	No	Eastern Whipbird, Scarlet Honeyeater, Silvereye	No	No	**Moderate wind**
20160117_ 100300	https://www.ecosounds.org/listen/353221?start=11830	No	No	Eastern Yellow Robin	No	No	**Strong wind**
20151027_ 085400	https://www.ecosounds.org/listen/352739?start=7687	**Cicadas between 6 and 9 kHz**	No	Scarlet Honeyeater, Golden Whistler	No	No	Strong wind
20160131_ 171300	https://www.ecosounds.org/listen/353310?start=14046	No	No	No	No	No	**Strong wind**

Cluster 41 - Gympie National Park

VERY QUIET (4437 minutes) The dominant sound sources are provided in bold text.

date_ time	Hyperlinks	Insects	Planes	Birds	Rain	Thunder	Wind
20150715_ 014400	https://www.ecosounds.org/listen/277036?start=6237	No	No	No	No	No	No
20150715_ 235400	https://www.ecosounds.org/listen/276990?start=12984	No	No	No	No	No	No
20150717_ 012200	https://www.ecosounds.org/listen/277039?start=4915	No	No	No	No	No	No
20150708_ 175200	https://www.ecosounds.org/listen/276953?start=15621	No	No	No	No	No	No
20150915_ 003100	https://www.ecosounds.org/listen/331898?start=1857	Insects (quiet) at 4.5 kHz	Very distant plane	No	No	No	No
20150712_ 225900	https://www.ecosounds.org/listen/276986?start=19251	No	No	No	No	No	No
20150716_ 023600	https://www.ecosounds.org/listen/276997?start=9355	No	Very distant plane	No	No	No	No
20150717_ 234000	https://www.ecosounds.org/listen/276996?start=12144	No	Very distant plane	No	No	No	No
20150801_ 010600	https://www.ecosounds.org/listen/277124?start=3956	No	No	No	No	No	No
20150715_ 205500	https://www.ecosounds.org/listen/276990?start=2244	No	Distant plane	No	No	No	No

Cluster 41 - Woondum National Park

VERY QUIET (1063 minutes) The dominant sound sources are provided in bold text.

date_ time	Hyperlinks	Insects	Planes	Birds	Rain	Thunder	Wind
20160528_ 213900	https://www.ecosounds.org/listen/354457?start=4842	No	Very distant plane	No	No	No	No
20150805_ 194000	https://www.ecosounds.org/listen/331505?start=22100	No	Distant plane	No	No	No	No
20160530_ 022900	https://www.ecosounds.org/listen/354463?start=8938	No	Distant plane	No	No	No	No
20150712_ 181300	https://www.ecosounds.org/listen/277003?start=18534	No	No	No	No	No	Slight wind
20160525_ 210300	https://www.ecosounds.org/listen/354441?start=2689	No	Very distant plane	No	No	No	No
20160528_ 051000	https://www.ecosounds.org/listen/354477?start=18589	No	Moderately loud plane	No	No	No	No
20150904_ 024500	https://www.ecosounds.org/listen/331754?start=9897	No	No	No	No	No	No
20160529_ 031200	https://www.ecosounds.org/listen/354481?start=11511	No	Very distant plane	No	No	No	No
20160530_ 234000	https://www.ecosounds.org/listen/354473?start=12156	No	No	No	No	No	Slight wind
20160530_ 002400	https://www.ecosounds.org/listen/354463?start=1438	No	No	No	No	No	No

Cluster 42 - Gympie National Park

STRONG WIND (4591 minutes) The dominant sound sources are provided in bold text.

date_ time	Hyperlinks	Insects	Planes	Birds	Rain	Thunder	Wind
20160209_ 101900	https://www.ecosounds.org/listen/353342?start=12789	Cicadas above 6 kHz	No	No	No	No	**Strong wind**
20160702_ 084100	https://www.ecosounds.org/listen/354821?start=6910	No	No + **motorbikes**	Yes + **Animal movement**	No	No	Slight wind
20150812_ 123200	https://www.ecosounds.org/listen/331509?start=20764	No	No	Scarlet Honeyeater	No	No	**Strong wind**
20160120_ 144000	https://www.ecosounds.org/listen/353178?start=4098	**Cicadas above 7 kHz**	No	No	No	No	**Strong wind in 2nd 30 seconds**
20160614_ 215000	https://www.ecosounds.org/listen/354608?start=5550	No	No	No	**Moderate rain**	No	No
20160206_ 015200	https://www.ecosounds.org/listen/353305?start=6717	Insects between 5 and 6 kHz	No	No	No	No	**Very strong wind**
20150812_ 125700	https://www.ecosounds.org/listen/331509?start=22264	No	**Moderately loud plane**	Scarlet Honeyeater	No	No	**Strong wind**
20160116_ 052900	https://www.ecosounds.org/listen/354025?start=19738	No	No	Lewin's Honeyeater	No	No	**Strong wind**
20151022_ 135200	https://www.ecosounds.org/listen/354767?start=1218	Insects above 7 kHz	No	No	No	No	**Very strong wind**
20160212_ 153000	https://www.ecosounds.org/listen/353358?start=7102	No	No	Spotted Pardalote	No	No	**Very strong wind**

Cluster 42 - Woondum National Park

STRONG WIND (2602 minutes) The dominant sound sources are provided in bold text.

date_ time	Hyperlinks	Insects	Planes	Birds	Rain	Thunder	Wind
20150712_ 001900	https://www.ecosounds.org/listen/276984?start=1138	No	No	No	No	No	**Very strong wind**
20160702_ 071400	https://www.ecosounds.org/listen/354849?start=1689	No	No	Scarlet Honeyeater	No	No	**Moderate wind**
20160128_ 184700	https://www.ecosounds.org/listen/353241?start=18919	**Cicadas and insects above 2 kHz**	No	No	No	No	No
20151228_ 120900	https://www.ecosounds.org/listen/353853?start=19386	**Cicadas above 2.8 kHz**	No	No	No	No	**Strong wind**
20150805_ 124000	https://www.ecosounds.org/listen/331506?start=21249	No	No	Eastern Whipbird	No	No	**Very strong wind**
20160202_ 184300	https://www.ecosounds.org/listen/353314?start=18677	**Cicadas above 0 kHz**	No	No	No	No	No
20160624_ 140600	https://www.ecosounds.org/listen/354679?start=2021	No	**Loud plane**	No	No	No	Strong wind
20151022_ 121400	https://www.ecosounds.org/listen/352718?start=19687	Insects above 7 kHz	No	Yes	No	No	**Very strong wind**
20160108_ 162400	https://www.ecosounds.org/listen/354029?start=10343	**Insects above 6 kHz**	No	No	No	No	Moderate wind
20160130_ 081000	https://www.ecosounds.org/listen/353274?start=5044	No	No	**Laughing Kookaburra, Rufous Fantail**	No	No	**Strong wind**

Cluster 43 - Gympie National Park

BIRDS - MORNING CHORUS (3324 minutes) The dominant sound sources are provided in bold text.

date_ time	Hyperlinks	Insects	Planes	Birds	Rain	Thunder	Wind
20160505_ 073800	https://www.ecosounds.org/listen/354251?start=3128	No	No	**Torresian Crow, Eastern Whipbird, Fan-tailed Cuckoo, Spotted Pardalote**	No	No	No
20151112_ 042400	https://www.ecosounds.org/listen/352882?start=15838	No	No	**Eastern Yellow Robin, White-throated Honeyeater,**	No	No	Moderate wind
20150921_ 052000	https://www.ecosounds.org/listen/333215?start=19198	No	Distant plane	**Eastern Yellow Robin, White-throated Honeyeater,**	**Light rain**	No	No
20160106_ 060900	https://www.ecosounds.org/listen/353890?start=22136	No	No	**White-throated Honeyeater, Spangled Drongo, White-throated Trecreeper, Rainbow Lorikeet**	No	No	Moderate wind
20151207_ 085800	https://www.ecosounds.org/listen/353095?start=7931	No	No	**Rufous Whistler**	No	No	Moderate wind
20151010_ 045200	https://www.ecosounds.org/listen/333349?start=17517	No	No	**Eastern Yellow Robin, Leaden Flycatcher, White-throated Honeyeater, Laughing Kookaburra**	No	No	Moderate wind
20160514_ 081800	https://www.ecosounds.org/listen/354365?start=5509	No	Moderately loud plane	**Eastern Whipbird,**	No	No	Moderate to light wind
20160603_ 061900	https://www.ecosounds.org/listen/354507?start=22738	No	No	**Lewin's Honeyeater, White-throated Honeyeater, Eastern Whipbird, Pied Currawong**	No	No	Slight wind
20160507_ 061000	https://www.ecosounds.org/listen/354280?start=22196	No	No	**White-throated Honeyeater, Lewin's Honeyeater, Grey Fantail,**	No	No	Slight wind
20160417_ 064100	https://www.ecosounds.org/listen/354091?start=24057	No	No	**Torresian Crow, Rose Robin, Spotted Pardalote**	No	No	Slight wind

Cluster 43 - Woondum National Park

BIRDS - MORNING CHORUS (4168 minutes) The dominant sound sources are provided in bold text.

date_ time	Hyperlinks	Insects	Planes	Birds	Rain	Thunder	Wind
20150909_ 090400	https://www.ecosounds.org/listen/331866?start=8286	No	No	**Rufous Whistler, White-throated Treecreeper, Scarlet Honeyeater,**	No	No	No
20150926_ 095900	https://www.ecosounds.org/listen/333264?start=11587	No	**Moderately loud plane**	**Brown Cuckoo-dove, Rufous Whistler, Lewin's Honeyeater, Scarlet Honeyeater**	No	No	No
20160227_ 084600	https://www.ecosounds.org/listen/353524?start=7207	No	No	**Lorikeets ?, Australian Figbird**	No	No	No
20160221_ 111000	https://www.ecosounds.org/listen/353445?start=15841	No	No	**Scarlet Honeyeater**	No	No	No
20150830_ 075900	https://www.ecosounds.org/listen/331819?start=4382	No	No	**Golden Whistler, Scarlet Honeyeater, Torresian Crow,**	No	No	Slight wind-moderate
20150720_ 061800	https://www.ecosounds.org/listen/277097?start=22678	No	No	**White-throated Treecreeper, Scarlet Honeyeater, Eastern Whipbird,**	**Very light rain**	No	No
20160210_ 084300	https://www.ecosounds.org/listen/353376?start=7028	No	No	**Fairywren, Lewin's Honeyeater, Rainbow Lorikeet**	No	No	No
20160227_ 104100	https://www.ecosounds.org/listen/353524?start=14107	No	Distant plane	**Rainbow Lorikeet**	No	No	No
20151001_ 095600	https://www.ecosounds.org/listen/333313?start=11404	No	No	**Rufous Whistler, Scarlet Honeyeater,**	No	No	No
20150818_ 055000	https://www.ecosounds.org/listen/331638?start=20997	No	No	**Scarlet Honeyeater**	No	No	No

Cluster 44 - Gympie National Park

LOUD CICADAS (3858 minutes) The dominant sound sources are provided in bold text.

date_ time	Hyperlinks	Insects	Planes	Birds	Rain	Thunder	Wind
20160112_ 171700	https://www.ecosounds.org/listen/354033?start=13518	**Cicadas between 6 and 9 kHz**	No	Lewin's Honeyeater	No	No	Moderate wind
20160124_ 045400	https://www.ecosounds.org/listen/353188?start=17638	**Cicadas between 1.8 and 9 kHz**	No	No	No	No	No
20151227_ 185100	https://www.ecosounds.org/listen/353838?start=19157	**Cicadas (loud) between 1.8 and 9 kHz**	No	No	Light rain	No	No
20151215_ 135700	https://www.ecosounds.org/listen/353134?start=1521	**Cicadas above 1.8 kHz**	No	White-throated Treecreeper	No	No	No
20160707_ 065300	https://www.ecosounds.org/listen/354866?start=404	No	**Moderately loud plane**	White-throated Honeyeater, Eastern Whipbird	No	No	Slight wind
20151209_ 104400	https://www.ecosounds.org/listen/353083?start=14288	**Cicadas above 7 kHz**	No	Crested Shrike-tit	No	No	No
20151205_ 160600	https://www.ecosounds.org/listen/353012?start=9258	**Cicadas above 6 kHz**	No	Mistletoebird	No	No	No
20151231_ 043900	https://www.ecosounds.org/listen/353866?start=16738	**Cicadas above 2 kHz**	No	White-throated Honeyeater, Laughing Kookaburra	No	No	No
20151123_ 165700	https://www.ecosounds.org/listen/352960?start=12319	**Cicadas above 6.5 kHz**	No	Cicadabird, Eastern Yellow Robin	No	No	No
20151121_ 183100	https://www.ecosounds.org/listen/352899?start=17956	**Cicadas above 1.9 kHz**	Yes	Unknown	No	No	No

Cluster 44 - Woondum National Park

LOUD CICADAS (7223 minutes) The dominant sound sources are provided in bold text.

date_ time	Hyperlinks	Insects	Planes	Birds	Rain	Thunder	Wind
20160219_ 140800	https://www.ecosounds.org/listen/353411?start=2177	**Cicadas above 2.8 kHz**	No	No	No	No	Strong wind
20160201_ 190600	https://www.ecosounds.org/listen/353334?start=20063	**Loud cicadas above 2.8 kHz**	No	No	No	No	Strong wind
20160322_ 053200	https://www.ecosounds.org/listen/353678?start=19918	**Cicadas between 2.8 and 9 kHz**	No	Lewin's Honeyeater	No	No	Strong wind
20160215_ 183100	https://www.ecosounds.org/listen/353420?start=17967	**Loud cicadas above 1.8 kHz**	No	Pied Currawong	No	No	No
20151129_ 183300	https://www.ecosounds.org/listen/353038?start=2263	**Cicadas above 1.8 kHz and at 0.9 kHz**	No	No	No	No	No
20160123_ 133200	https://www.ecosounds.org/listen/353218?start=11	**Cicada (zizzt) above 5 kHz**	**Loud plane**	No	No	No	Moderate wind
20160205_ 185400	https://www.ecosounds.org/listen/353330?start=19341	**Cicadas above 2 kHz**	**Loud plane**	No	No	No	No
20160213_ 184400	https://www.ecosounds.org/listen/353389?start=18732	**Cicadas above 1.9 kHz**	No	No	No	No	No
20160213_ 095800	https://www.ecosounds.org/listen/353387?start=11524	**Cicadas above 2 kHz**	No	No	No	No	Moderate wind
20160127_ 102700	https://www.ecosounds.org/listen/353235?start=13266	**Cicadas above 2.8 kHz**	No	Eastern Yellow Robin	No	No	Moderate wind

Cluster 45 - Gympie National Park

WIND AND PLANES (2767 minutes) The dominant sound sources are provided in bold text.

date_ time	Hyperlinks	Insects	Planes	Birds	Rain	Thunder	Wind
20160222_ 152800	https://www.ecosounds.org/listen/353477?start=6981	**Insects between 7.5 and 9.5 kHz**	No	Lewin's Honeyeater, Unknown	No	No	**Slight wind**
20160321_ 152800	https://www.ecosounds.org/listen/353643?start=6982	No	No	Lewin's Honeyeater, White-throated Honeyeater	No	No	**Moderate wind**
20160310_ 170700	https://www.ecosounds.org/listen/353521?start=12918	Insects between 7 and 9 kHz	**Moderatel y loud plane**	Spotted Pardalote	No	No	Moderate wind
20160423_ 135700	https://www.ecosounds.org/listen/354142?start=1520	No	No	Grey Fantail, White-throated Gerygone	No	No	**Moderate wind**
20160303_ 135300	https://www.ecosounds.org/listen/353947?start=1280	**Cicadas above 6 kHz**	No	Fan-tailed Cuckoo, Lewin's Honeyeater	No	No	Moderate wind
20160317_ 171400	https://www.ecosounds.org/listen/353577?start=13336	No	No	White-throated Treecreeper, Pied Butcherbird	No	No	**Slight wind**
20160330_ 102200	https://www.ecosounds.org/listen/353681?start=12971	Cicadas between 6 and 9 kHz	No	**Lewin's Honeyeater, Fan-tailed Cuckoo, Fan-tailed Cuckoo**	No	No	Moderate wind
20150903_ 125500	https://www.ecosounds.org/listen/331722?start=22149	No	No	Eastern Yellow Robin	No	No	**Strong wind**
20160205_ 103400	https://www.ecosounds.org/listen/353323?start=13690	Cicadas between 7 and 9 kHz	No	White-throated Honeyeater,	No	No	**Moderate wind**
20150924_ 061800	https://www.ecosounds.org/listen/333227?start=22677	No	**Loud plane**	Eastern Whipbird, Scarlet Honeyeater, White-throated Treecreeper, White-throated Honeyeater	No	No	Moderate wind

Cluster 45 - Woondum National Park

WIND AND PLANES (613 minutes) The dominant sound sources are provided in bold text.

date_ time	Hyperlinks	Insects	Planes	Birds	Rain	Thunder	Wind
20160112_ 100200	https://www.ecosounds.org/listen/354071?start=11767	Insects above 6 kHz	Distant plane	White-throated Treecreeper	Very light	No	**Moderate wind**
20151222_ 170000	https://www.ecosounds.org/listen/353857?start=12497	Insects between 6 and 9 kHz	**Loud plane**	Spangled Drongo, Cicadabird	No	No	No
20150908_ 114200	https://www.ecosounds.org/listen/331859?start=17767	No	Moderately loud plane	White-throated Treecreeper, Rufous Whistler + Animal movement	No	No	**Moderate wind**
20151205_ 070400	https://www.ecosounds.org/listen/353040?start=1086	Insects between 5 and 8 kHz	**Loud plane**	Grey Shrike-thrush, White-throated Treecreeper	No	No	No
20160118_ 122200	https://www.ecosounds.org/listen/353200?start=20167	Insects between 7.8 and 9 kHz	No	White-throated Treecreeper	No	No	**Moderate wind**
20151120_ 135000	https://www.ecosounds.org/listen/352922?start=1097	Distant cicadas above 7 kHz	No	**Golden Whistler**	No	No	**Moderate wind**
20160124_ 101700	https://www.ecosounds.org/listen/353248?start=12663	**Insects above 5 kHz**	**Moderately loud plane**	No	No	No	No
20150925_ 141200	https://www.ecosounds.org/listen/333261?start=2418	No	Distant plane	Yes	No	No	**Moderate to strong wind**
20151101_ 161900	https://www.ecosounds.org/listen/352813?start=7321	Insects between 5 and 9 kHz	No	Scarlet Honeyeater, Mistletoebird, Eastern Yellow Robin	No	No	**Moderate to strong wind**
20150916_ 114600	https://www.ecosounds.org/listen/331893?start=18008	No	No	Scarlet Honeyeater, Mistletoebird	No	No	**Moderate wind**

Cluster 46 - Gympie National Park

WIND (2333 minutes) The dominant sound sources are provided in bold text.

date_ time	Hyperlinks	Insects	Planes	Birds	Rain	Thunder	Wind
20160206_ 104900	https://www.ecosounds.org/listen/353328?start=14587	No	No	Yes	No	No	**Moderate wind**
20151018_ 071000	https://www.ecosounds.org/listen/354722?start=1447	**Cicadas above 6 kHz**	No	Fan-tailed Cuckoo, Scarlet Honeyeater, Lewin's Honeyeater	No	No	Moderate wind
20160609_ 212600	https://www.ecosounds.org/listen/354574?start=4069	No	No	No	No	No	Moderate wind
20160716_ 051200	https://www.ecosounds.org/listen/354931?start=18715	No	No	No	**Light rain**	No	Moderate wind
20151123_ 094500	https://www.ecosounds.org/listen/352956?start=10748	**Cicadas above 4 kHz**	No	Torresian Crow	No	No	No
20150804_ 184600	https://www.ecosounds.org/listen/331456?start=18861	No	No	No	**Light rain**	No	No
20160425_ 021200	https://www.ecosounds.org/listen/354207?start=7917	No	Distant plane	No	No	No	**Moderate wind**
20150825_ 010800	https://www.ecosounds.org/listen/331758?start=4077	No	No	No	No	No	**Moderate wind**
20151013_ 081000	https://www.ecosounds.org/listen/354706?start=5049	**Cicadas above 5.5 kHz**	Distant plane	Lewin's Honeyeater, Grey Shrike-thrush, White-throated Treecreeper	No	No	Moderate wind
20151123_ 194900	https://www.ecosounds.org/listen/352960?start=22639	Insects between 4.8 and 6.2 kHz	Moderatel y loud plane	No	No	No	**Moderate wind**

Cluster 46 - Woondum National Park

WIND (5594 minutes) The dominant sound sources are provided in bold text.

date_ time	Hyperlinks	Insects	Planes	Birds	Rain	Thunder	Wind
20160116_ 004900	https://www.ecosounds.org/listen/354058?start=2936	Insects between 4 and 6.2 kHz	No	No	**Light rain**	No	No
20160720_ 093100	https://www.ecosounds.org/listen/355006?start=9888	No	**Moderately loud plane**	Yes	No	No	**Moderate wind**
20160627_ 091100	https://www.ecosounds.org/listen/354851?start=8711	No	No	No	**Light rain**	No	Moderate wind
20160117_ 111700	https://www.ecosounds.org/listen/353221?start=16270	No	Distant plane	No	No	No	**Moderate wind**
20160715_ 203700	https://www.ecosounds.org/listen/354968?start=1164	No	No	No	Light rain	No	**Moderate wind**
20160620_ 093800	https://www.ecosounds.org/listen/354659?start=10316	No	No	Yes	No	No	**Moderate wind**
20160205_ 033800	https://www.ecosounds.org/listen/353349?start=13077	**Insects between 5 and 6 kHz**	No	No	No	No	**Moderate wind**
20160425_ 152100	https://www.ecosounds.org/listen/354181?start=6562	No	**Moderately loud plane**	Yes	No	No	**Moderate wind**
20160530_ 163000	https://www.ecosounds.org/listen/354486?start=10703	No	No	No	No	No	**Moderate wind**
20151008_ 225500	https://www.ecosounds.org/listen/333370?start=9451	Insects between 5 and 6 kHz	No	No	**Light rain**	No	Moderate wind

Cluster 47 - Gympie National Park

VERY STRONG WIND (10032 minutes) The dominant sound sources are provided in bold text.

date_ time	Hyperlinks	Insects	Planes	Birds	Rain	Thunder	Wind
20150917_ 074700	https://www.ecosounds.org/listen/331920?start=3668	No	No	Brown Cuckoo-dove, Rufous Whistler, Leaden Flycatcher, Mistletoebird	No	No	**Strong wind**
20160605_ 110500	https://www.ecosounds.org/listen/354569?start=1120	No	No	No	No	No	**Very strong wind**
20151204_ 153200	https://www.ecosounds.org/listen/353030?start=7224	No	No	White-throated Treecreeper	No	No	**Very strong wind**
20150812_ 180500	https://www.ecosounds.org/listen/331539?start=16393	Insects between 4 and 5 kHz	No	No	No	No	**Very strong wind**
20160130_ 002800	https://www.ecosounds.org/listen/353276?start=1676	No	No	No	**Heavy rain**	**Moderately loud thunder**	**Moderate wind**
20151022_ 123600	https://www.ecosounds.org/listen/354764?start=21007	No	**Moderately loud plane**	No	No	No	**Strong wind**
20150929_ 150000	https://www.ecosounds.org/listen/333277?start=5299	No	No	Torresian Crow, Scarlet Honeyeater	No	No	**Strong wind**
20160129_ 230500	https://www.ecosounds.org/listen/353270?start=10052	No	No	No	No	No	**Strong wind**
20150812_ 170000	https://www.ecosounds.org/listen/331539?start=12493	No	No	Scarlet Honeyeater	No	No	**Strong wind**
20160210_ 132200	https://www.ecosounds.org/listen/353346?start=23761	Insect zizzt	No	White-throated Honeyeater	No	No	**Very strong wind**

Cluster 47 - Woondum National Park

VERY STRONG WIND (4600 minutes) The dominant sound sources are provided in bold text.

date_ time	Hyperlinks	Insects	Planes	Birds	Rain	Thunder	Wind
20160723_ 132800	https://www.ecosounds.org/listen/355029?start=24110	No	No	No	No	No	**Strong wind**
20160624_ 150900	https://www.ecosounds.org/listen/354679?start=5801	No	Distant plane	No	No	No	**Very strong wind**
20150903_ 111300	https://www.ecosounds.org/listen/331751?start=16028	No	**Loud plane**	No	No	No	**Very strong wind**
20160127_ 172000	https://www.ecosounds.org/listen/353234?start=13697	No	Moderate plane	No	No	No	**Strong wind**
20150902_ 211300	https://www.ecosounds.org/listen/331740?start=3331	Insects between 5 and 6 kHz	No	No	No	No	**Strong wind**
20150812_ 050700	https://www.ecosounds.org/listen/331536?start=18415	No	No	No	No	No	**Strong wind**
20151025_ 105600	https://www.ecosounds.org/listen/352757?start=57	No	No	Mistletoebird + **Animal movement**	No	No	**Strong wind**
20150928_ 150200	https://www.ecosounds.org/listen/333302?start=5426	No	No	Rufous Whistler, Scarlet Honeyeater	No	No	**Strong wind**
20150706_ 182000	https://www.ecosounds.org/listen/276970?start=12880	Insects between 4 and 5 kHz	No	No	No	No	**Strong wind**
20160627_ 123100	https://www.ecosounds.org/listen/354851?start=20711	No	No	No	No	No	**Very strong wind**

Cluster 48 - Gympie National Park

CICADAS (3901 minutes) The dominant sound sources are provided in bold text.

date_ time	Hyperlinks	Insects	Planes	Birds	Rain	Thunder	Wind
20160111_ 124200	https://www.ecosounds.org/listen/354004?start=21369	**Cicadas above 2 kHz**	No	No	No	No	No
20160308_ 121800	https://www.ecosounds.org/listen/353509?start=19929	**Cicadas above 3 kHz**	No	Fan-tailed Cuckoo, Mistletoebird	No	No	No
20160113_ 124700	https://www.ecosounds.org/listen/354011?start=21667	**Cicadas above 3 kHz**	**Loud plane**	Lewin's Honeyeater, White-throated Treecreeper	No	No	No
20160102_ 141600	https://www.ecosounds.org/listen/353878?start=2657	**Cicadas above 3 kHz**	No	Eastern Yellow Robin	No	No	No
20151215_ 102300	https://www.ecosounds.org/listen/353108?start=13029	**Cicadas above 2.4 kHz**	No	Lewin's Honeyeater	No	No	No
20160125_ 130000	https://www.ecosounds.org/listen/353256?start=22449	**Cicadas above 3 kHz**	No	Mistletoebird	No	No	No
20160220_ 110400	https://www.ecosounds.org/listen/353467?start=15486	**Cicadas above 2.8 kHz**	No	White-throated Treecreeper, Cicadabird	No	No	No
20160220_ 184200	https://www.ecosounds.org/listen/353444?start=18617	**Cicadas between 1.9 and 9 kHz**	Distant plane	Torresian Crow, Australian Magpie	No	No	No
20160326_ 134600	https://www.ecosounds.org/listen/353641?start=859	**Cicadas above 3 kHz**	No	Lewin's Honeyeater, White-throated Gerygone	No	No	No
20151223_ 043700	https://www.ecosounds.org/listen/353797?start=16618	**Cicadas between 2 and 9 kHz**	No	White-throated Honeyeater, Grey Shrike-thrush	No	No	No

Cluster 48 - Woondum National Park

CICADAS (11044 minutes) The dominant sound sources are provided in bold text.

date_ time	Hyperlinks	Insects	Planes	Birds	Rain	Thunder	Wind
20160223_ 103000	https://www.ecosounds.org/listen/353508?start=13446	**Cicadas above 2 kHz and zizzts**	No	No + Animal movement	No	No	No
20151211_ 133600	https://www.ecosounds.org/listen/353087?start=256	**Cicadas above 2.5 kHz**	No	No	No	No	No
20160303_ 151300	https://www.ecosounds.org/listen/354002?start=6081	**Cicadas above 2.5 kHz**	No	Lewin's Honeyeater, Grey Shrike-thrush	No	No	No
20160201_ 120700	https://www.ecosounds.org/listen/353311?start=19271	**Cicadas above 3 kHz**	No	Yes	No	No	Strong wind
20160227_ 052400	https://www.ecosounds.org/listen/353523?start=19437	**Cicadas above 2 kHz**	Moderately loud plane	Yes	No	No	No
20160221_ 090200	https://www.ecosounds.org/listen/353445?start=8161	**Cicadas above 3 kHz**	Moderately loud plane	Golden Whistler	No	No	Strong wind
20151206_ 132400	https://www.ecosounds.org/listen/353043?start=4790	**Cicada zizzt above 2.8 kHz**	No	White-throated Treecreeper	No	No	No
20160228_ 140100	https://www.ecosounds.org/listen/353961?start=2334	**Cicadas zizzt above 2.8 kHz**	Distant plane	Scarlet Honeyeater, Lewin's Honeyeater	No	No	No
20160315_ 103300	https://www.ecosounds.org/listen/353596?start=13627	**Cicadas between 2.5 and 6 kHz and above 8 kHz**	No	Yes	No	No	No
20151211_ 145700	https://www.ecosounds.org/listen/353087?start=5116	**Cicadas above 2.8 kHz**	No	No	No	No	Moderate wind

Cluster 49 - Gympie National Park

PLANES (INCLUDING THUNDER) (2173 minutes) The dominant sound sources are provided in bold text.

date_ time	Hyperlinks	Insects	Planes	Birds	Rain	Thunder	Wind
20160601_ 102000	https://www.ecosounds.org/listen/354526?start=12847	No	**Very loud plane**	Lewin's Honeyeater	No	No	No
20151211_ 221800	https://www.ecosounds.org/listen/353104?start=7228	**Insects between 4.6 and 6.2 kHz**	No	No	Light	**Loud thunder**	No
20151128_ 232200	https://www.ecosounds.org/listen/352951?start=11065	Insects between 5 and 6.4 kHz	**Loud plane**	No	No	No	No
20150809_ 151400	https://www.ecosounds.org/listen/331502?start=9419	Insects between 6.5 and 7.5 kHz	**Very loud plane**	**Scarlet Honeyeater, Lewin's Honeyeater,**	No	No	Slight wind
20160201_ 113200	https://www.ecosounds.org/listen/353281?start=17171	Insects between 2 and 8 kHz	Plane (?)	No	No	No	Slight wind
20150706_ 143000	https://www.ecosounds.org/listen/277044?start=19179	Insects between 7 and 9 kHz	**Loud plane**	Lewin's Honeyeater, White-throated Honeyeater	No	No	No
20150716_ 125400	https://www.ecosounds.org/listen/276995?start=22084	Insects between 6 and 8 kHz	**Loud plane**	Scarlet Honeyeater, Lewin's Honeyeater, Mistletoebird, Grey Fantail	No	No	No
20151208_ 094200	https://www.ecosounds.org/listen/353078?start=10564	Insects between 6 and 9 kHz	**Loud plane**	White-throated Treecreeper, Mistletoebird, Rufous Whistler	No	No	No
20160608_ 140800	https://www.ecosounds.org/listen/354579?start=2145	Insects between 7 and 8 kHz	**Loud plane**	Yes	No	No	No
20150925_ 160900	https://www.ecosounds.org/listen/333233?start=9443	No	**Loud plane**	Scarlet Honeyeater, White-throated Treecreeper,	No	No	No

Cluster 49 - Woondum National Park

PLANES (INCLUDING THUNDER) (1025 minutes) The dominant sound sources are provided in bold text.

date_ time	Hyperlinks	Insects	Planes	Birds	Rain	Thunder	Wind
20160530_ 175900	https://www.ecosounds.org/listen/354486?start=16043	Insects between 4 and 6.2 kHz	**Very loud plane**	No	No	No	No
20160528_ 112200	https://www.ecosounds.org/listen/354460?start=16548	No	**Very loud plane**	No	No	No	No
20150812_ 172700	https://www.ecosounds.org/listen/331541?start=14113	No	No	Distant birds	No	**Loud thunder**	Slight wind
20150925_ 171800	https://www.ecosounds.org/listen/333261?start=13578	No	**Loud plane**	White-throated Treecreeper, Yellow-faced Honeyeater, Scarlet Honeyeater, Spectacled Monarch	No	No	Slight wind
20160330_ 121800	https://www.ecosounds.org/listen/353712?start=19928	No	**Loud plane**	Lewin's Honeyeater, unknown	No	No	Slight wind
20151202_ 082400	https://www.ecosounds.org/listen/353024?start=5884	Insects between 6 and 8 kHz	**Loud plane**	Golden Whistler, Grey Shrike-thrush,	No	No	Slight wind
20160213_ 073000	https://www.ecosounds.org/listen/353387?start=2644	No	**Loud plane**	Golden Whistler, Lewin's Honeyeater, Scarlet Honeyeater	No	No	No
20150716_ 121700	https://www.ecosounds.org/listen/277014?start=19866	No	**Very loud plane**	Lewin's Honeyeater, Scarlet Honeyeater	No	No	No
20160712_ 150200	https://www.ecosounds.org/listen/354969?start=5418	No	**Loud plane**	Scarlet Honeyeater	No	No	Slight wind
20160412_ 115500	https://www.ecosounds.org/listen/354097?start=18548	No	No	White-throated Treecreeper, Golden Whistler	No	**Very Loud thunder**	Slight wind

Cluster 50 - Gympie National Park

QUIET AND INSECTS AND BIRDS (1766 minutes) - Inconsistent The dominant sound sources are provided in bold text.

date_ time	Hyperlinks	Insects	Planes	Birds	Rain	Thunder	Wind
20150823_ 220700	https://www.ecosounds.org/listen/331764?start=17619	No	No	No	No	No	Moderate wind
20150922_ 231700	https://www.ecosounds.org/listen/333218?start=10770	**Insects(quiet) at 5 kHz**	Very distant plane	No	No	No	No
20160719_ 191100	https://www.ecosounds.org/listen/354999?start=20328	Insects (quiet) between 4.5 and 5.2 kHz	Distant plane	No	No	No	No
20151205_ 230100	https://www.ecosounds.org/listen/353011?start=9809	**Insects between 3.8 and 5.2 kHz**	No	No	No	No	No
20151024_ 222300	https://www.ecosounds.org/listen/354774?start=7534	**Insect between 3.8 and 5.2 kHz**	Very distant plane	No	No	No	No
20160712_ 234300	https://www.ecosounds.org/listen/354918?start=12333	Insects (quiet) between 4.5 and 5 kHz	Very distant plane	No	No	No	Slight wind
20160612_ 053700	https://www.ecosounds.org/listen/354595?start=20209	Insects at 4 kHz	No	No	No	No	No
20151117_ 213200	https://www.ecosounds.org/listen/352881?start=4472	**Insect between 3.8 and 5.2 kHz**	No	No	No	No	No
20150817_ 181700	https://www.ecosounds.org/listen/331613?start=17122	**Insects at 4.5 kHz**	Very distant plane	No	No	No	No
20150707_ 165300	https://www.ecosounds.org/listen/277051?start=12083	No	No	**White-throated Honeyeater, Eastern Whipbird, Grey Fantail**	No	No	No

Cluster 50 - Woondum National Park

QUIET AND INSECTS OR BIRDS (3180 minutes) - Inconsistent The dominant sound sources are provided in bold text.

date_ time	Hyperlinks	Insects	Planes	Birds	Rain	Thunder	Wind
20160125_ 033900	https://www.ecosounds.org/listen/353251?start=13138	**Insects between 4 and 5 kHz**	No	No	No	No	Slight wind
20160527_ 123300	https://www.ecosounds.org/listen/354455?start=20805	No	No	White-throated Honeyeater,	No	No	Moderate wind
20151118_ 225700	https://www.ecosounds.org/listen/352912?start=9564	Insects (quiet) between 4 and 5.2 kHz	Very distant plane	No	No	No	No
20160110_ 024300	https://www.ecosounds.org/listen/354034?start=9775	**Insects between 3.9 and 5.5 kHz**	No	No	No	No	No
20150723_ 224400	https://www.ecosounds.org/listen/277091?start=8795	**Insect between 4 and 5.2 kHz**	No	No	No	No	Moderate wind
20160322_ 160300	https://www.ecosounds.org/listen/353652?start=9076	No	No	**Lewin's Honeyeater, White-throated Treecreeper**	No	No	No
20160525_ 110700	https://www.ecosounds.org/listen/354468?start=15658	No	Distant plane	**Red-backed Fairy-wren**	No	No	No
20151219_ 060100	https://www.ecosounds.org/listen/353175?start=21658	No	Distant plane	**Brown Cuckoo-dove, White-throated Treecreeper, Cicadabird, Olive-backed Oriole**	No	No	No
20160117_ 074900	https://www.ecosounds.org/listen/353221?start=3790	No	No	**White-throated Honeyeater**	No	No	No
20160103_ 062900	https://www.ecosounds.org/listen/353939?start=23336	No	No	Cicadabird	No	No	No

Cluster 51 - Gympie National Park

STRONG WIND (4708 minutes) The dominant sound sources are provided in bold text.

date_ time	Hyperlinks	Insects	Planes	Birds	Rain	Thunder	Wind
20160429_ 151200	https://www.ecosounds.org/listen/354223?start=5990	Insect from 7.5 to 9.5 kHz	No	Scarlet Honeyeater	No	No	**Strong wind**
20150811_ 184600	https://www.ecosounds.org/listen/331534?start=18855	No	No	No	No	No	**Very strong wind**
20150812_ 113100	https://www.ecosounds.org/listen/331509?start=17104	No	**Moderately loud plane**	Scarlet Honeyeater	No	No	**Very strong wind**
20150726_ 130000	https://www.ecosounds.org/listen/277079?start=22448	No	No	Scarlet Honeyeater, Eastern Whipbird	No	No	**Strong wind**
20160302_ 163700	https://www.ecosounds.org/listen/353945?start=11117	Cicadas above 6 kHz	No	White-throated Honeyeater, White-throated Treecreeper	No	No	**Very strong wind**
20160604_ 041900	https://www.ecosounds.org/listen/354536?start=15537	No	No	No	**Light rain**	No	**Very strong wind**
20160206_ 171600	https://www.ecosounds.org/listen/353329?start=13456	No	No	**Rainbow Lorikeet, Spangled Drongo, White-throated Honeyeater, White-throated Treecreeper**	No	No	**Moderate wind**
20160116_ 063000	https://www.ecosounds.org/listen/354025?start=23398	No	No	White-throated Treecreeper	No	No	**Very strong wind**
20160619_ 090900	https://www.ecosounds.org/listen/354640?start=8586	No	No	No	Very light rain	No	**Very strong wind**
20160606_ 040200	https://www.ecosounds.org/listen/354546?start=14508	No	No	No	No	No	**Strong wind**

Cluster 51 - Woondum National Park

STRONG WIND (4253 minutes) The dominant sound sources are provided in bold text.

date_ time	Hyperlinks	Insects	Planes	Birds	Rain	Thunder	Wind
20151229_ 100200	https://www.ecosounds.org/listen/353823?start=11768	No	No	Mistletoebird	No	No	**Very strong wind**
20151228_ 104600	https://www.ecosounds.org/listen/353853?start=14406	No	No	Yes	No	No	**Very strong wind**
20160220_ 120700	https://www.ecosounds.org/listen/353415?start=19264	**Cicadas above 2.8 kHz**	Loud plane	Yes	No	No	No
20150626_ 113800	https://www.ecosounds.org/listen/331583?start=17528	No	No	Yes	No	No	**Very strong wind**
20160406_ 100200	https://www.ecosounds.org/listen/353770?start=11765	No	No	Grey Fantail, Mistletoebird	No	No	**Strong to very strong wind**
20160226_ 100000	https://www.ecosounds.org/listen/353519?start=11647	**Cicadas between 2.2 and 8 kHz**	**Loud plane**	Red-backed Fairy-wren, Lewin's Honeyeater	No	No	No
20150920_ 183100	https://www.ecosounds.org/listen/333241?start=17018	Insects at 5.2 kHz	Distant plane	No	No	No	**Moderate wind**
20150813_ 120300	https://www.ecosounds.org/listen/331543?start=19028	No	No	Golden Whistler	No	No	**Strong wind**
20160714_ 121700	https://www.ecosounds.org/listen/354976?start=19866	No	No	Eastern Whipbird	No	No	**Moderate wind**
20151025_ 133200	https://www.ecosounds.org/listen/352757?start=9417	**Cicadas between 6 and 9 kHz**	No	Golden Whistler, Mistletoebird	No	No	**Strong wind**

Cluster 52 - Gympie National Park

WIND (16324 minutes) The dominant sound sources are provided in bold text.

date_ time	Hyperlinks	Insects	Planes	Birds	Rain	Thunder	Wind
20160425_ 144900	https://www.ecosounds.org/listen/354210?start=4643	No	Distant plane	White-throated Treecreeper	No	No	**Slight wind in trees**
20160121_ 180300	https://www.ecosounds.org/listen/353206?start=16280	No	No	White-throated Treecreeper	No	No	**Slight wind in trees**
20160426_ 120300	https://www.ecosounds.org/listen/354213?start=19026	No	**Very loud plane**	No	No	No	**Slight wind**
20160406_ 111200	https://www.ecosounds.org/listen/353765?start=15970	No	Plane	Yes	No	No	**Slight wind**
20151026_ 141300	https://www.ecosounds.org/listen/352734?start=2484	**Cicadas above 6 kHz**	No	**Rufous Whistler, White-throated Honeyeater, White-throated Treecreeper**	No	No	No
20151116_ 081800	https://www.ecosounds.org/listen/352878?start=5530	Cicadas above 3 kHz	No	Eastern Whipbird, Rainbow Lorikeet	No	No	**Slight wind**
20160404_ 161000	https://www.ecosounds.org/listen/353732?start=9502	No	No	No	No	No	**Moderate wind**
20151129_ 083300	https://www.ecosounds.org/listen/352955?start=6427	Insects between 7 and 8 kHz	No	Fan-tailed Cuckoo	No	No	**Moderate wind**
20160228_ 013200	https://www.ecosounds.org/listen/353475?start=5518	Insects between 5 and 6.5 kHz	No	No	No	No	**Moderate wind**
20160126_ 171000	https://www.ecosounds.org/listen/353260?start=13103	Insects between 5 and 6 kHz	No	White-throated Treecreeper, Cicadabird	No	No	**Moderate wind**

Cluster 52 - Woondum National Park

WIND (2413 minutes) The dominant sound sources are provided in bold text.

date_ time	Hyperlinks	Insects	Planes	Birds	Rain	Thunder	Wind
20160712_ 140500	https://www.ecosounds.org/listen/354969?start=1998	No	No	No	No	No	**Moderate wind**
20151014_ 174200	https://www.ecosounds.org/listen/354737?start=15019	No	**Loud plane**	Golden Whistler, Yellow-faced Honeyeater	No	No	No
20150902_ 192400	https://www.ecosounds.org/listen/331750?start=21140	Insects at 5.2 kHz	No	No	No	No	**Moderate wind**
20151220_ 141700	https://www.ecosounds.org/listen/353153?start=2708	No	No	Grey Shrike-thrush	No	No	**Moderate wind**
20160401_ 190900	https://www.ecosounds.org/listen/353744?start=20232	**Insects between 4.5 and 6.5 kHz + Cicada zizzt**	No	No	No	No	No
20151120_ 150700	https://www.ecosounds.org/listen/352922?start=5717	No	No	Yes	No	No	**Moderate wind**
20150928_ 170200	https://www.ecosounds.org/listen/333302?start=12626	No	No	Eastern Yellow Robin, Scarlet Honeyeater	No	No	Strong wind
20151219_ 145700	https://www.ecosounds.org/listen/353179?start=5115	No	Distant plane	Yellow-faced Honeyeater	No	No	**Moderate wind**
20150829_ 190600	https://www.ecosounds.org/listen/331803?start=20056	No	No	No	**Moderate rain**	No	No
20151114_ 110300	https://www.ecosounds.org/listen/352866?start=15428	**Cicadas above 7 kHz**	No	Variegated Fairy-wren	No	No	**Slight wind**

Cluster 53 - Gympie National Park

MOSTLY QUIET (6378 minutes) Almost exclusively at Gympie NP The dominant sound sources are provided in bold text.

date_ time	Hyperlinks	Insects	Planes	Birds	Rain	Thunder	Wind
20160513_ 040500	https://www.ecosounds.org/listen/354357?start=14691	No	No	No	No	No	No
20151027_ 211100	https://www.ecosounds.org/listen/352710?start=3214	Insects at 5 kHz	No	No	Light to Moderate rain	Quiet thunder	No
20160512_ 211400	https://www.ecosounds.org/listen/354334?start=3377	Insects at 5 kHz	No	No	No	No	No
20160512_ 205000	https://www.ecosounds.org/listen/354334?start=1937	Insects at 4.9 kHz	No	No	No	No	No
20160516_ 041000	https://www.ecosounds.org/listen/354352?start=15000	No	No	No	No	No	No
20160515_ 014400	https://www.ecosounds.org/listen/354345?start=6230	No	No	No	No	No	No
20160512_ 222800	https://www.ecosounds.org/listen/354334?start=7817	Insects at 4.7 kHz	No	No	No	No	No
20160513_ 003200	https://www.ecosounds.org/listen/354357?start=1911	No	Very distant plane	No	No	No	No
20160611_ 015300	https://www.ecosounds.org/listen/354570?start=6770	No	Very distant plane	No	No	No	No
20160514_ 032200	https://www.ecosounds.org/listen/354341?start=12110	Insects at 4.6 kHz	Distant plane	No	No	No	No

Cluster 53 - Woondum National Park

MOSTLY QUIET (204 minutes) Almost exclusively at Gympie NP The dominant sound sources are provided in bold text.

date_ time	Hyperlinks	Insects	Planes	Birds	Rain	Thunder	Wind
20160421_ 112700	https://www.ecosounds.org/listen/354191?start=16863	No	Distant plane	Distant birds	No	No	No
20150801_ 133900	https://www.ecosounds.org/listen/277158?start=438	No	No	No	No	No	No
20160608_ 190700	https://www.ecosounds.org/listen/354528?start=20088	Insects between 4 and 4.5 kHz	Distant plane	No	No	No	No
20150831_ 212600	https://www.ecosounds.org/listen/331727?start=4120	No	No	Yes	No	No	Slight wind
20150909_ 195200	https://www.ecosounds.org/listen/331869?start=22816	Insects between 4 and 4.5 kHz	No	Yes	No	No	No
20160521_ 125200	https://www.ecosounds.org/listen/354425?start=21965	No	No	No	No	No	No
20160609_ 002500	https://www.ecosounds.org/listen/354529?start=1490	No	Loud plane	No	No	No	No
20160517_ 164400	https://www.ecosounds.org/listen/354382?start=11535	No	Moderately loud plane	White-throated Treecreeper	No	No	No
20160419_ 151900	https://www.ecosounds.org/listen/354161?start=6439	No	No	No	No	No	No
20160707_ 154100	https://www.ecosounds.org/listen/354901?start=7724	No	Distant plane	No	No	No	Moderate wind

Cluster 54 - Gympie National Park

MODERATE RAIN AND BIRDS (1445 minutes) The dominant sound sources are provided in bold text.

date_ time	Hyperlinks	Insects	Planes	Birds	Rain	Thunder	Wind
20160614_ 103300	https://www.ecosounds.org/listen/354607?start=13628	No	No	Eastern Whipbird	**Moderate rain**	No	No
20160516_ 092400	https://www.ecosounds.org/listen/354375?start=9493	No	Moderately loud plane	**Golden Whistler, White-throated Treecreeper**	No	No	No
20150718_ 123400	https://www.ecosounds.org/listen/277001?start=20888	No	No	**Grey Fantail**	No	No	No
20160430_ 130700	https://www.ecosounds.org/listen/354229?start=22852	No	No	**White-throated Honeyeater**	**Light rain**	No	No
20160705_ 121600	https://www.ecosounds.org/listen/354860?start=19789	No	No	**White-throated Honeyeater, Torresian Crow**	**Light rain**	No	No
20160617_ 172300	https://www.ecosounds.org/listen/354646?start=13881	No	No	**Grey Fantail**	No	No	No
20150624_ 133700	https://www.ecosounds.org/listen/331597?start=312	No	No	**Torresian Crow, Yellow-tailed Black Cockatoo, Grey Fantail, Scarlet Honeyeater**	**Light rain**	No	No
20160616_ 030000	https://www.ecosounds.org/listen/354638?start=10796	No	No	No	**Light rain**	No	No
20160616_ 060300	https://www.ecosounds.org/listen/354638?start=21776	No	No	No	**Light rain**	No	No
20150709_ 013600	https://www.ecosounds.org/listen/277057?start=5758	No	No	No	**Moderate rain**	No	No

Cluster 54 - Woondum National Park

MODERATE RAIN AND BIRDS (11589 minutes) The dominant sound sources are provided in bold text.

date_ time	Hyperlinks	Insects	Planes	Birds	Rain	Thunder	Wind
20160311_ 040100	https://www.ecosounds.org/listen/353554?start=14455	Insects - some	No	No	**Moderate rain**	No	No
20150723_ 045200	https://www.ecosounds.org/listen/277109?start=17517	No	No	No	**Moderate rain**	No	No
20160207_ 002700	https://www.ecosounds.org/listen/353360?start=1616	Insects between 4.8 and 7 kHz	No	No	**Moderate rain**	No	Slight wind
20151008_ 185400	https://www.ecosounds.org/listen/333371?start=19340	**Insects between 5 and 6 kHz**	No	No	**Moderate rain**	No	No
20150724_ 052000	https://www.ecosounds.org/listen/277093?start=19197	Insects - distant	No	No	**Moderate rain**	No	No
20160302_ 221900	https://www.ecosounds.org/listen/353985?start=7293	**Insects between 4.8 and 7 kHz**	No	No	**Moderate rain**	No	No
20160314_ 013800	https://www.ecosounds.org/listen/353591?start=5880	Insects between 4.8 and 7 kHz	No	No	**Moderate rain**	No	No
20150822_ 042900	https://www.ecosounds.org/listen/331663?start=16137	Insects - distant	No	No	**Moderate rain**	No	No
20160320_ 004900	https://www.ecosounds.org/listen/353640?start=2936	Insects between 4.8 and 7 kHz	No	No	**Moderate rain**	No	No
20160414_ 113400	https://www.ecosounds.org/listen/354129?start=17288	No	No	No	**Moderate rain**	No	No

Cluster 55 - Gympie National Park

QUIET (8208 minutes) The dominant sound sources are provided in bold text.

date_ time	Hyperlinks	Insects	Planes	Birds	Rain	Thunder	Wind
20150929_ 224300	https://www.ecosounds.org/listen/333275?start=8729	Insects at 4.3 kHz	Very distant plane	No	No	No	No
20160512_ 220000	https://www.ecosounds.org/listen/354334?start=6137	Quiet insects at 4.5 kHz	No	No	No	No	No
20160513_ 214100	https://www.ecosounds.org/listen/354339?start=4973	Quiet Insects at 4.7 to 5 kHz	No	No	No	No	No
20160531_ 055200	https://www.ecosounds.org/listen/354517?start=21117	No	Distant plane	No	No	No	No
20160521_ 035100	https://www.ecosounds.org/listen/354369?start=13858	No	No	No	No	No	No
20160517_ 011200	https://www.ecosounds.org/listen/354374?start=4318	No	No	No	No	No	No
20160505_ 001200	https://www.ecosounds.org/listen/354273?start=718	Quiet insects between 4.5 and 5 kHz	No	No	No	No	No
20160413_ 043600	https://www.ecosounds.org/listen/354072?start=16556	Insects between 4 and 5 kHz	Very distant plane	No	No	No	No
20160519_ 011000	https://www.ecosounds.org/listen/354361?start=4197	Insects at 4.9 kHz	No	No	No	No	No
20150801_ 051100	https://www.ecosounds.org/listen/277124?start=18656	No	Very distant plane	No	No	No	No

Cluster 55 - Woondum National Park

QUIET (1211 minutes) The dominant sound sources are provided in bold text.

date_ time	Hyperlinks	Insects	Planes	Birds	Rain	Thunder	Wind
20150805_ 233200	https://www.ecosounds.org/listen/331476?start=11672	No	Distant plane	No	No	No	No
20160512_ 194100	https://www.ecosounds.org/listen/354327?start=22142	Insects between 4 and 5 kHz	Distant plane	No	No	No	No
20160530_ 034600	https://www.ecosounds.org/listen/354463?start=13558	No	Very distant plane	No	No	No	No
20160606_ 235800	https://www.ecosounds.org/listen/354531?start=13191	No	Very distant plane	No	No	No	No
20150928_ 024000	https://www.ecosounds.org/listen/333299?start=9600	No	Moderately loud plane	No	No	No	No
20150903_ 194300	https://www.ecosounds.org/listen/331752?start=22278	Insects between 4.7 and 5.0 kHz	Moderately loud plane	No	No	No	No
20160610_ 234400	https://www.ecosounds.org/listen/354538?start=12340	No	Very distant plane	No	No	No	No
20160530_ 035100	https://www.ecosounds.org/listen/354463?start=13858	No	No	No	No	No	No
20150717_ 200200	https://www.ecosounds.org/listen/277019?start=23420	No	Very distant plane	No	No	No	No
20160529_ 184100	https://www.ecosounds.org/listen/354511?start=15857	Insects between 4 and 5 kHz	Moderately loud plane	No + Animal movement	No	No	No

Cluster 56 - Gympie National Park

WIND (6419 minutes) The dominant sound sources are provided in bold text.

date_ time	Hyperlinks	Insects	Planes	Birds	Rain	Thunder	Wind
20160407_ 155500	https://www.ecosounds.org/listen/353743?start=8597	No	Very distant plane	Yes	No	No	No
20160324_ 163600	https://www.ecosounds.org/listen/353632?start=11062	Quiet cicadas above 7 kHz	No	Yes	No	No	No
20150728_ 135700	https://www.ecosounds.org/listen/277129?start=1513	No	**Moderately loud plane**	Yes	No	No	**Moderate wind**
20160624_ 203600	https://www.ecosounds.org/listen/354660?start=1067	No	No	No	No	No	**Moderate wind**
20160228_ 050400	https://www.ecosounds.org/listen/353475?start=18238	Insects between 5 and 6 kHz	No	No	No	No	**Moderate wind**
20150924_ 124700	https://www.ecosounds.org/listen/333228?start=21669	No	Distant plane	**Lewin's Honeyeater, Scarlet Honeyeater**	No	No	Slight wind
20151012_ 170700	https://www.ecosounds.org/listen/354704?start=12925	No	No	Shining Bronze Cuckoo, Mistletoebird, Torresian Crow	No	No	**Strong wind**
20151008_ 023300	https://www.ecosounds.org/listen/333340?start=9176	No	No	No	No	No	**Moderate wind**
20160322_ 003000	https://www.ecosounds.org/listen/353622?start=1798	Insects between 5 and 5.5 kHz	No	No	No	No	**Strong wind**
20160121_ 121200	https://www.ecosounds.org/listen/353181?start=19569	Cicadas above 8 kHz	No	Lewin's Honeyeater	No	No	Slight wind

Cluster 56 - Woondum National Park

WIND (2228 minutes) The dominant sound sources are provided in bold text.

date_ time	Hyperlinks	Insects	Planes	Birds	Rain	Thunder	Wind
20151203_ 160300	https://www.ecosounds.org/listen/353054?start=9074	No	Moderate to loud plane	Yes	No	No	**Strong wind**
20160122_ 174000	https://www.ecosounds.org/listen/353216?start=14894	Cicadas above 5 kHz	Distant plane	No	No	No	**Moderate to strong wind**
20151114_ 115800	https://www.ecosounds.org/listen/352866?start=18728	**Cicadas above 6.5 kHz**	**Loud plane**	Mistletoebird	No	No	No
20160409_ 140500	https://www.ecosounds.org/listen/353779?start=1998	No	No	Grey Fantail	Light rain	No	**Slight wind**
20151004_ 165800	https://www.ecosounds.org/listen/333355?start=7728	No	No	Scarlet Honeyeater, White-throated Treecreeper	Light rain	No	Moderate wind
20160416_ 135300	https://www.ecosounds.org/listen/354141?start=1272	No	No	Spotted Pardalote	No	No	**Moderate wind**
20160122_ 133200	https://www.ecosounds.org/listen/353216?start=14	**Cicada zizzt above 7 kHz**	No	Creaking Tree	No	No	**Strong wind**
20160324_ 104500	https://www.ecosounds.org/listen/353685?start=14349	No	**Loud plane**	Scarlet Honeyeater, Lewin's Honeyeater	No	No	No
20160528_ 131100	https://www.ecosounds.org/listen/354460?start=23088	No	No	No	No	No	**Strong wind**
20160628_ 032800	https://www.ecosounds.org/listen/354853?start=12477	No	No	No	No	No	**Moderate wind**

Cluster 57 - Gympie National Park

BIRDS OR WIND (745 minutes) - Inconsistent The dominant sound sources are provided in bold text.

date_ time	Hyperlinks	Insects	Planes	Birds	Rain	Thunder	Wind
20160310_ 100600	https://www.ecosounds.org/listen/353520?start=12008	Insects above 4 kHz	No	**Torresian Crow, Wonga Pigeon, Mistletoebird, Spotted Pardalote, Lewin's Honeyeater, Eastern Whipbird, Red-backed Fairy-wren**	No	No	No
20160215_ 170900	https://www.ecosounds.org/listen/353449?start=13037	Insects above 6.5 kHz	No	**Torresian Crow, Rainbow Lorikeet, White-throated Honeyeater, Lewin's Honeyeater**	No	No	No
20160207_ 163600	https://www.ecosounds.org/listen/353338?start=20822	Insects between 6 and 9 kHz	No	**Rainbow Lorikeet**	No	No	Slight wind
20160717_ 112200	https://www.ecosounds.org/listen/354935?start=16571	No	**Moderately loud plane**	**Eastern Whipbird**	No	No	Moderate wind
20160321_ 064200	https://www.ecosounds.org/listen/353619?start=24118	No	No	**Torresian Crow, White-throated Treecreeper, Eastern Whipbird, Rufous Fantail, Spotted Pardalote**	No	No	No
20160417_ 060600	https://www.ecosounds.org/listen/354091?start=21957	No	No	**Eastern Whipbird, Lewin's Honeyeater, White-naped Honeyeater + Animal movements**	No	No	No
20160320_ 064700	https://www.ecosounds.org/listen/353589?start=69	Insects above 7 kHz	No	**Torresian Crow, Magpie, White-throated Honeyeater**	No	No	No
20160313_ 122900	https://www.ecosounds.org/listen/353533?start=20588	No	No	**Torresian Crow, White-winged Chough**	Light	No	Slight wind
20160314_ 071100	https://www.ecosounds.org/listen/353563?start=1506	Insects above 6.5 kHz	No	**Torresian Crow, Rufous Fantail, Eastern Whipbird, Grey Butcherbird, Spotted Pardalote**	No	No	Slight wind
20160313_ 114800	https://www.ecosounds.org/listen/353533?start=18128	Insects above 6 kHz	Distant plane	**Torresian Crow, Eastern Whipbird, Rufous Whistler**	No	No	Slight wind

Cluster 57 - Woondum National Park

BIRDS OR WIND (1585 minutes) - Inconsistent The dominant sound sources are provided in bold text.

date_ time	Hyperlinks	Insects	Planes	Birds	Rain	Thunder	Wind
20160405_ 093400	https://www.ecosounds.org/listen/353786?start=10087	No	No	Yes	Light rain	No	**Slight wind**
20151213_ 113700	https://www.ecosounds.org/listen/353127?start=1091	No	No	Grey Shrike-thrush	No	No	**Moderate wind**
20160227_ 160000	https://www.ecosounds.org/listen/353503?start=8897	Insects between 8 and 9 kHz	No	**Rainbow Lorikeet**	No	No	No
20150928_ 173400	https://www.ecosounds.org/listen/333302?start=14546	No	**Moderately loud plane**	**Scarlet Honeyeater, Red-browed Finch**	No	No	No
20151002_ 131800	https://www.ecosounds.org/listen/333317?start=23529	No	No	Yes	No	No	**Strong wind**
20151025_ 150800	https://www.ecosounds.org/listen/352757?start=15177	Cicadas between 5.5 and 9 kHz	No	**Golden Whistler, Mistletoebird**	No	No	Strong wind
20160614_ 130000	https://www.ecosounds.org/listen/354581?start=22448	No	No	No	Light	No	**Moderate wind**
20151025_ 151700	https://www.ecosounds.org/listen/352757?start=15717	No	No	**Golden Whistler, Mistletoebird**	No	No	Moderate to strong wind
20151001_ 183000	https://www.ecosounds.org/listen/333314?start=17894	**Insects at 0.9 and 2 kHz and between 4.8 and 5.2 kHz**	Moderately loud plane	No	**Light rain**	No	**Moderate wind**
20160227_ 065900	https://www.ecosounds.org/listen/353524?start=787	No	No	**Rainbow Lorikeet**	No	No	Slight wind

Cluster 58 - Gympie National Park

LOUD BIRDS (3121 minutes) The dominant sound sources are provided in bold text.

date_ time	Hyperlinks	Insects	Planes	Birds	Rain	Thunder	Wind
20160320_ 072800	https://www.ecosounds.org/listen/353589?start=2529	No	No	**Torresian Crow, White-throated Treecreeper, Fan-tailed Cuckoo**	No	No	No
20160521_ 164600	https://www.ecosounds.org/listen/354371?start=11659	No	No	**Spangled Drongo, Unknown**	No	No	No
20160311_ 084100	https://www.ecosounds.org/listen/353550?start=6906	Insects above 7 kHz	**Moderately loud plane**	**Torresian Crow**	No	No	No
20150913_ 173700	https://www.ecosounds.org/listen/331914?start=20158	No	No	**Laughing Kookaburra, Lewin's Honeyeater, White-throated Honeyeater**	No	No	No
20151226_ 090400	https://www.ecosounds.org/listen/353811?start=8289	Insects between 7 and 9 kHz	Moderately loud plane	**Cicadabird, Rufous Whistler, Spangled Drongo, White-throated Honeyeater**	No	No	No
20160320_ 085500	https://www.ecosounds.org/listen/353589?start=7749	No	No	**Torresian Crow, Fan-tailed Cuckoo**	No	No	Moderate wind
20150922_ 095200	https://www.ecosounds.org/listen/333220?start=11168	No	No	**Torresian Crow, Laughing Kookaburra**	No	No	Moderate wind
20160311_ 115500	https://www.ecosounds.org/listen/353550?start=18546	Insects between 6 and 7 kHz	No	**Torresian Crow**	No	No	Moderate wind
20160721_ 054200	https://www.ecosounds.org/listen/355004?start=20507	No	No	**Laughing Kookaburra (chorus)**	No	No	No
20150830_ 084500	https://www.ecosounds.org/listen/331775?start=7145	No	No	**Rufous Whistler, Scarlet Honeyeater, Fan-tailed Cuckoo**	No	No	Moderate wind

Cluster 58 - Woondum National Park

LOUD BIRDS (423 minutes) The dominant sound sources are provided in bold text.

date_ time	Hyperlinks	Insects	Planes	Birds	Rain	Thunder	Wind
20151203_ 055200	https://www.ecosounds.org/listen/353027?start=21115	Insects (quiet) between 6 and 8 kHz	No	**Laughing Kookaburra, Pied Currawong**	No	No	Moderate wind
20150918_ 133500	https://www.ecosounds.org/listen/331888?start=194	No	Loud plane	**Golden Whistler, Rufous Whistler**	No	No	Slight wind-moderate
20160628_ 171300	https://www.ecosounds.org/listen/354831?start=13277	No	No	**Yellow-tailed Black Cockatoo, Pied Currawong, White-throated Treecreeper, Grey Fantail**	No	No	No
20151023_ 072800	https://www.ecosounds.org/listen/354790?start=2526	Insects between 6 and 8 kHz	Loud plane	**Scarlet Honeyeater, Mistletoebird**	No	No	Slight to moderate wind
20151001_ 073000	https://www.ecosounds.org/listen/333313?start=2644	No	Loud plane	**Rufous Whistler, Scarlet Honeyeater, Yellow-faced Honeyeater**	No	No	No
20160130_ 074800	https://www.ecosounds.org/listen/353274?start=3724	No	No	**Laughing Kookaburra, Grey Fantail**	No	No	Moderate wind
20160718_ 075000	https://www.ecosounds.org/listen/354994?start=3831	No	No	**Laughing Kookaburra**	No	No	Strong wind
20150926_ 084800	https://www.ecosounds.org/listen/333264?start=7327	No	**Very loud plane**	**Yellow-faced Honeyeater, Scarlet Honeyeater, Rufous Whistler, Golden Whistler**	No	No	No
20150911_ 065000	https://www.ecosounds.org/listen/331853?start=248	No	Loud plane	**Torresian Crow, Eastern Yellow Robin**	No	No	No
20151011_ 124800	https://www.ecosounds.org/listen/333381?start=21727	No	**Loud plane**	**Golden Whistler**	No	No	No

Cluster 59 - Gympie National Park

MODERATE RAIN (782 minutes) The dominant sound sources are provided in bold text.

date_ time	Hyperlinks	Insects	Planes	Birds	Rain	Thunder	Wind
20151114_ 171200	https://www.ecosounds.org/listen/352892?start=13218	No	No	No	**Moderate rain**	Moderately loud thunder	No
20160105_ 033500	https://www.ecosounds.org/listen/353884?start=12897	No	No	No	**Moderate rain**	No	No
20150829_ 213400	https://www.ecosounds.org/listen/331785?start=4586	No	No	No	**Moderate rain**	No	No
20160302_ 032700	https://www.ecosounds.org/listen/353966?start=12417	No	No	No	**Moderate rain**	No	No
20160501_ 040800	https://www.ecosounds.org/listen/354255?start=14874	No	No	No	**Moderate rain**	No	No
20160106_ 013000	https://www.ecosounds.org/listen/353890?start=5396	Insects at 4.8 kHz	No	No	**Moderate rain**	No	No
20150829_ 224100	https://www.ecosounds.org/listen/331785?start=8606	No	No	No	**Moderate rain**	No	No
20151115_ 030600	https://www.ecosounds.org/listen/352895?start=11157	Insects at 4.3 kHz	No	No	**Moderate rain**	No	No
20150723_ 013700	https://www.ecosounds.org/listen/277115?start=5817	No	No	No	**Moderate rain**	No	No
20150821_ 214800	https://www.ecosounds.org/listen/331623?start=5435	No	No	No	**Moderate rain**	No	No

Cluster 59 - Woondum National Park

MODERATE RAIN (10707 minutes) The dominant sound sources are provided in bold text.

date_ time	Hyperlinks	Insects	Planes	Birds	Rain	Thunder	Wind
20160202_ 180300	https://www.ecosounds.org/listen/353314?start=16277	No	No	No	**Heavy rain**	No	Yes
20160205_ 220000	https://www.ecosounds.org/listen/353344?start=6152	Insects at 6.8 kHz and 4.8 kHz	No	Eastern Yellow Robin	**Heavy rain**	No	No
20160426_ 204100	https://www.ecosounds.org/listen/354184?start=1410	No	No	No	**Heavy rain**	No	No
20151109_ 035500	https://www.ecosounds.org/listen/352844?start=14098	No	No	No	**Heavy rain**	No	No
20151028_ 192000	https://www.ecosounds.org/listen/352745?start=20900	No	No	No	**Moderate rain**	No	No
20160316_ 161700	https://www.ecosounds.org/listen/353600?start=9915	No	No	No	**Heavy rain**	No	No
20160716_ 071800	https://www.ecosounds.org/listen/354985?start=1927	No	No	No	**Heavy rain**	No	No
20151029_ 064600	https://www.ecosounds.org/listen/352749?start=8	No	No	Eastern Whipbird, Scarlet Honeyeater	**Moderate rain**	No	No
20160501_ 001700	https://www.ecosounds.org/listen/354201?start=1018	No	No	No	**Heavy rain**	No	No
20160604_ 081600	https://www.ecosounds.org/listen/354482?start=5405	No	No	Laughing Kookaburra	**Heavy rain**	No	No

Cluster 60 - Gympie National Park

RAIN OR BIRDS (276 minutes) The dominant sound sources are provided in bold text.

date_ time	Hyperlinks	Insects	Planes	Birds	Rain	Thunder	Wind
20160717_ 094700	https://www.ecosounds.org/listen/354935?start=10871	No	No	**Torresian Crow, Eastern Whipbird, Spotted Pardalote**	**Moderate rain**	No	No
20160301_ 053600	https://www.ecosounds.org/listen/353938?start=20158	Insects above 6 kHz	No	**Rainbow Lorikeet, Australian Magpie, Grey Shrike-thrush**	No	No	No
20160227_ 053300	https://www.ecosounds.org/listen/353471?start=19978	No	No	**Rainbow Lorikeet, Australian Magpie, Grey Shrike-thrush, Lewin's Honeyeater**	No	No	No
20160626_ 064900	https://www.ecosounds.org/listen/354671?start=168	No	Distant plane	**Eastern Whipbird, Australasian Figbird + Animal movement**	No	No	No
20160703_ 194800	https://www.ecosounds.org/listen/354872?start=4164	No	No	**Animal movement**	No	No	No
20160213_ 180600	https://www.ecosounds.org/listen/353384?start=16461	No	No	**Rainbow Lorikeet, Eastern Whipbird, Lewin's Honeyeater**	No	No	No
20160305_ 063600	https://www.ecosounds.org/listen/353955?start=23757	No	No	**Fan-tailed Cuckoo, Noisy miner, Spotted Pardalote, Brown Cuckoo-dove**	No	No	No
20160529_ 192100	https://www.ecosounds.org/listen/354490?start=22856	Insects between 4.5 and 5 kHz	No	**Animal movement**	No	No	No
20160520_ 042200	https://www.ecosounds.org/listen/354389?start=15717	No	No	**Animal movement**	No	No	No
20160126_ 160500	https://www.ecosounds.org/listen/353260?start=9203	No	No	**Australian King Parrot, White-throated Honeyeater, White-throated Treecreeper**	**Light rain**	No	No

Cluster 60 - Woondum National Park

RAIN OR BIRDS (323 minutes) The dominant sound sources are provided in bold text.

date_ time	Hyperlinks	Insects	Planes	Birds	Rain	Thunder	Wind
20151225_ 050300	https://www.ecosounds.org/listen/353867?start=18177	No	No	**Loud bird**	No	No	No
20151212_ 174100	https://www.ecosounds.org/listen/353091?start=14954	No	No	No	**Light-moderate rain**	No	Slight wind
20160716_ 214600	https://www.ecosounds.org/listen/354975?start=5311	No	No	No	**Moderate rain**	No	Slight wind
20160202_ 201900	https://www.ecosounds.org/listen/353312?start=88	Insects between 4.5 and 7 kHz	No	No	**Moderate rain**	No	Slight wind
20160302_ 040400	https://www.ecosounds.org/listen/353969?start=14640	No	No	No	**Moderate rain**	No	Slight wind
20160116_ 012200	https://www.ecosounds.org/listen/354058?start=4916	Insects between 4 and 6.5 kHz	No	No	**Light-moderate rain**	No	Slight wind
20151024_ 041400	https://www.ecosounds.org/listen/354791?start=15237	No	No	No	**Light-moderate rain**	No	Slight wind
20160604_ 021300	https://www.ecosounds.org/listen/354503?start=7975	No	No	No	**Moderate rain**	No	No
20160206_ 142400	https://www.ecosounds.org/listen/353356?start=3136	No	No	**Rainbow Lorikeet**	No	No	No
20160615_ 230500	https://www.ecosounds.org/listen/354584?start=10047	No	No	No	**Moderate rain**	No	No

References

1. Truskinger A, Cottman-Fields M, Eichinski P, Towsey M, Roe P. Practical Analysis of Big Acoustic Sensor Data for Environmental Monitoring. 2014 IEEE Fourth International Conference on Big Data and Cloud Computing; Sydney, Australia2014.
2. Truskinger A, Cottman-Fields M, Roe P. Acoustic Workbench (Version 1.2.2) [Computer software]. Brisbane: QUT Ecoacoustics Research Group 2017 [Available from: https://github.com/QutBioacoustics/.
