# Supplementary material for: Revealing the ecological content of long-duration audio-recordings of the environment through clustering and visualisation
Source: PLoS One. 2018 Mar 1;13(3):e0193345. doi: 10.1371/journal.pone.0193345 (PMC5832236; doi:10.1371/journal.pone.0193345)
Supplement: S5 File — Complete sets of the temporal distribution plots, radar plots, polar histograms for each of the 60 clusters. (PDF) [file pone.0193345.s005.pdf]

S3 Cluster Statistics

The following pages contain five sections:

Section A: Temporal distribution plots –showing the average number of minutes per two hour period per month for all sixty clusters

Section B: Polar Histograms for Gympie and Woondum National Park sites.

Section C: Radar Plots – radar plots showing the medoid of each cluster for each of the sixty clusters.

Section D: Boxplots – The boxplot statistics of each of the twelve summary acoustic indices for a select number of clusters in each acoustic class.

Section A: Two hour plots

The clusters are grouped in the acoustic classes: Birds, Insects, Cicadas, Planes, Wind, Rain and Quiet. Refer to article for information on each of these classes. These plots are scaled to the maximum of the average number of minutes in that cluster across the two sites. Some averages are large in relation to the 120 minute period, for example cluster 13 in the Quiet acoustic class has an average of 70 minutes. Some clusters are very small and have very low averages, for example clusters 6, 23, 24, 49, 50, 56 and 58. Both of the plane clusters are in this category. Clusters such as clusters 7 and 8 are mixtures of classes, in this case Cicadas, Birds and Wind. Clusters 7 and 8 were classified into the Cicada class because this is the most dominant sound in the minutes sampled, see S2 Sample minutes. The number of minutes in each cluster in each two hour period is averaged over the number of days recorded in each month. The recording started on the 22nd June 2015 and ended on the 23rd July 2016, as a consequence these months were averaged over nine and twenty-three days respectively.

BIRD CLUSTERS (#3, 11, 14, 15, 28, 33, 37, 39)

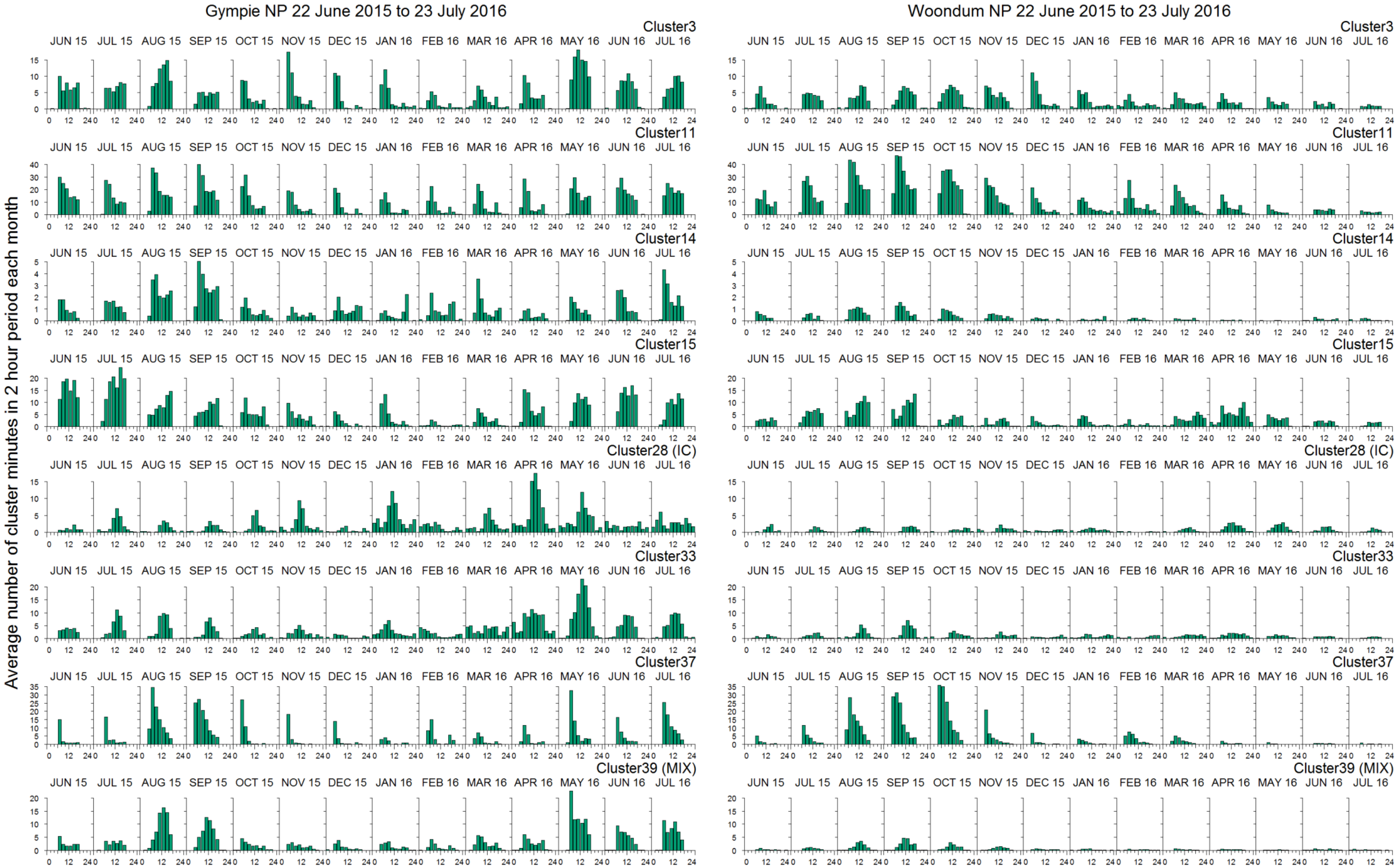

(MIX) - Cluster with more than one dominant acoustic class; (IC) - Inconsistent cluster

BIRDS CLUSTERS CONTINUED (#43, 57, 58)

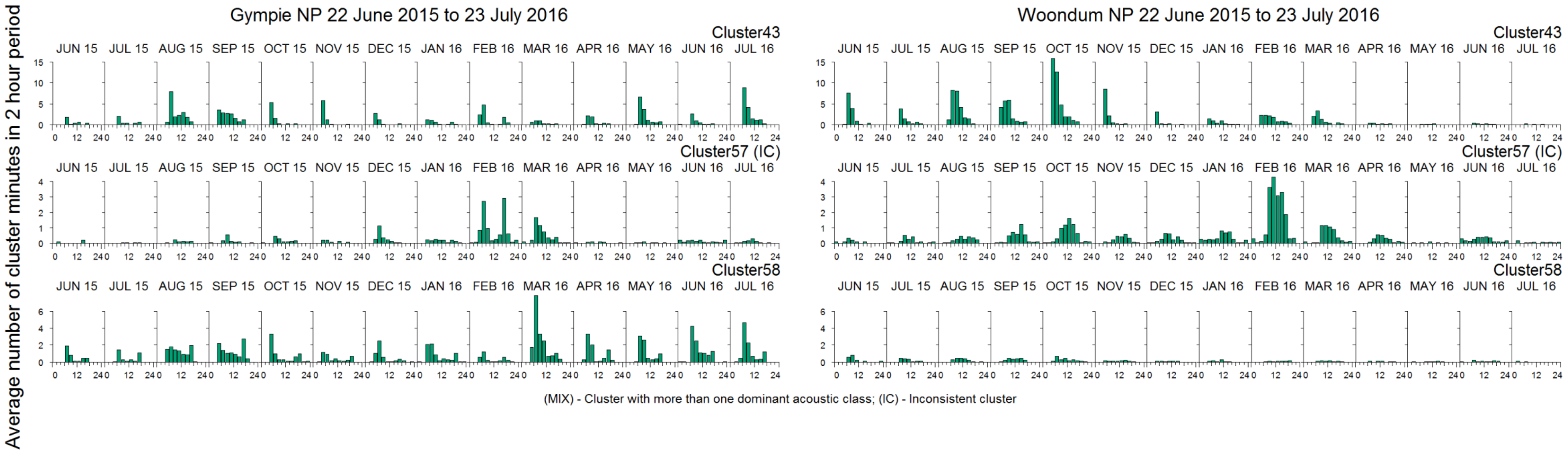

(MIX) - Cluster with more than one dominant acoustic class; (IC) - Inconsistent cluster

INSECT CLUSTERS (#1, 4, 22, 26, 27, 29)

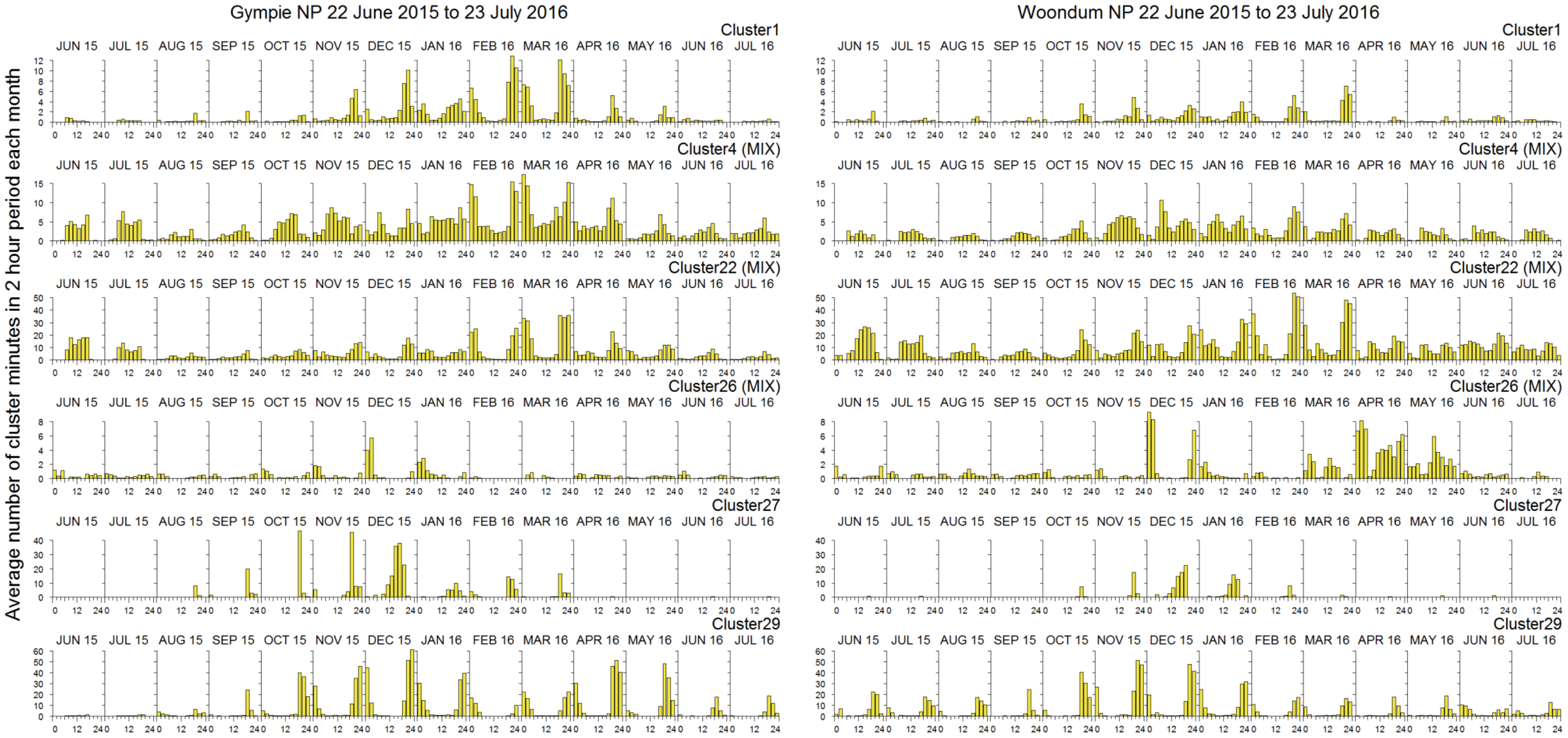

(MIX) - Cluster with more than one dominant acoustic class; (IC) - Inconsistent cluster

CICADA CLUSTERS (#7, 8, 12, 16, 32, 34, 44, 48)

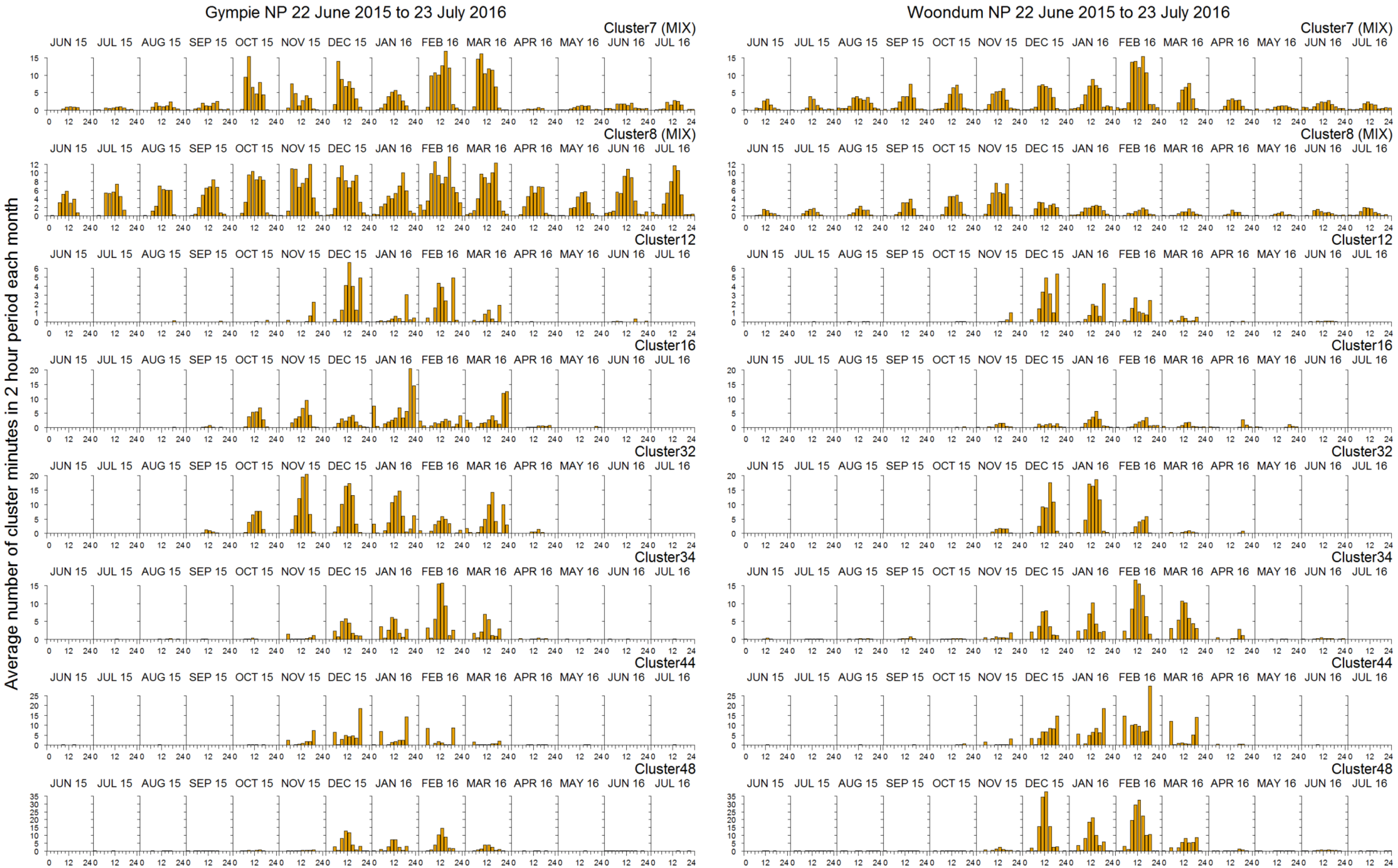

(MIX) - Cluster with more than one dominant acoustic class; (IC) - Inconsistent cluster

RAIN CLUSTERS (#2, 10, 17, 18, 21, 54, 59, 60)

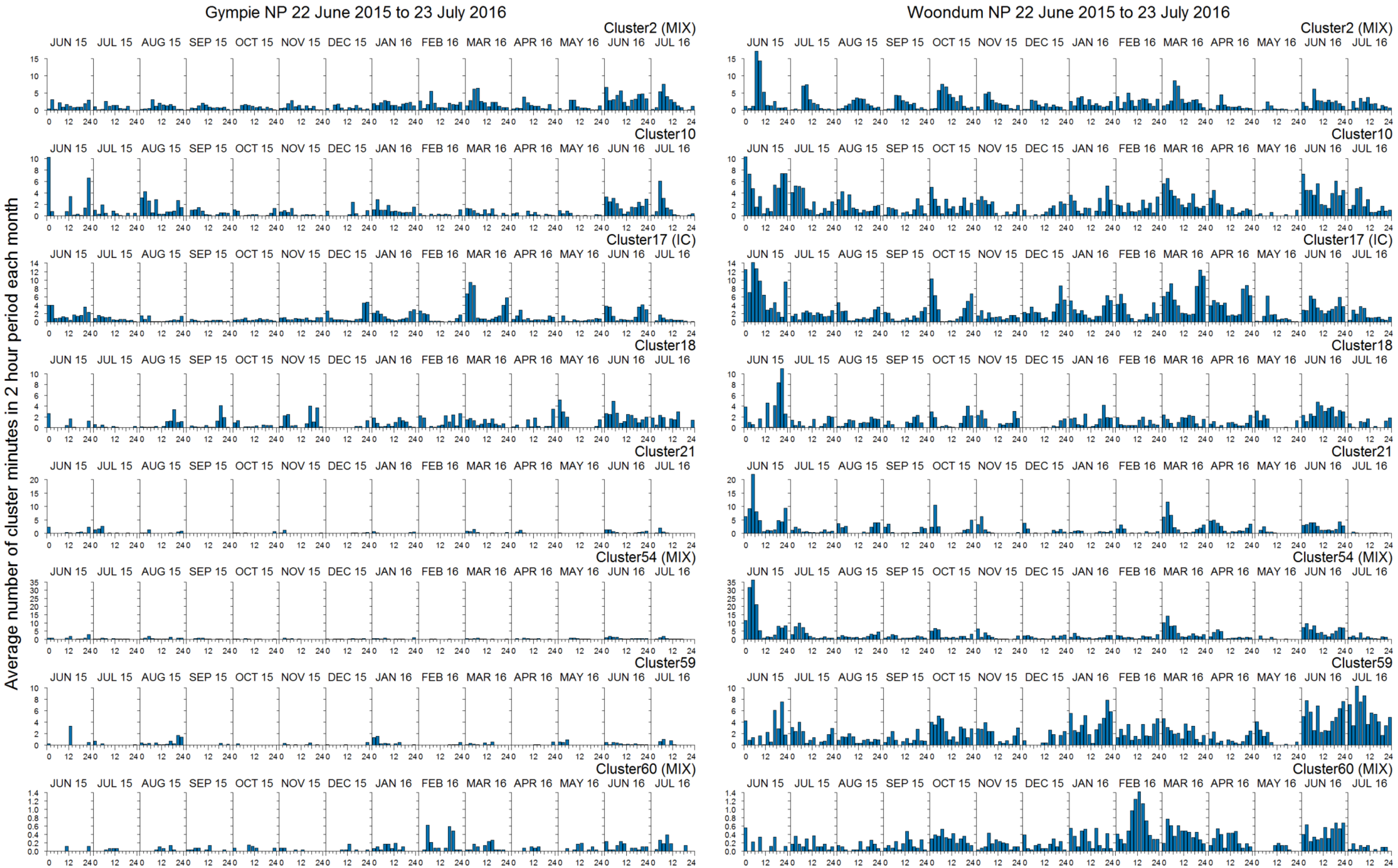

(MIX) - Cluster with more than one dominant acoustic class; (IC) - Inconsistent cluster

WIND CLUSTERS (#9, 19, 20, 24, 25, 30, 40, 42)

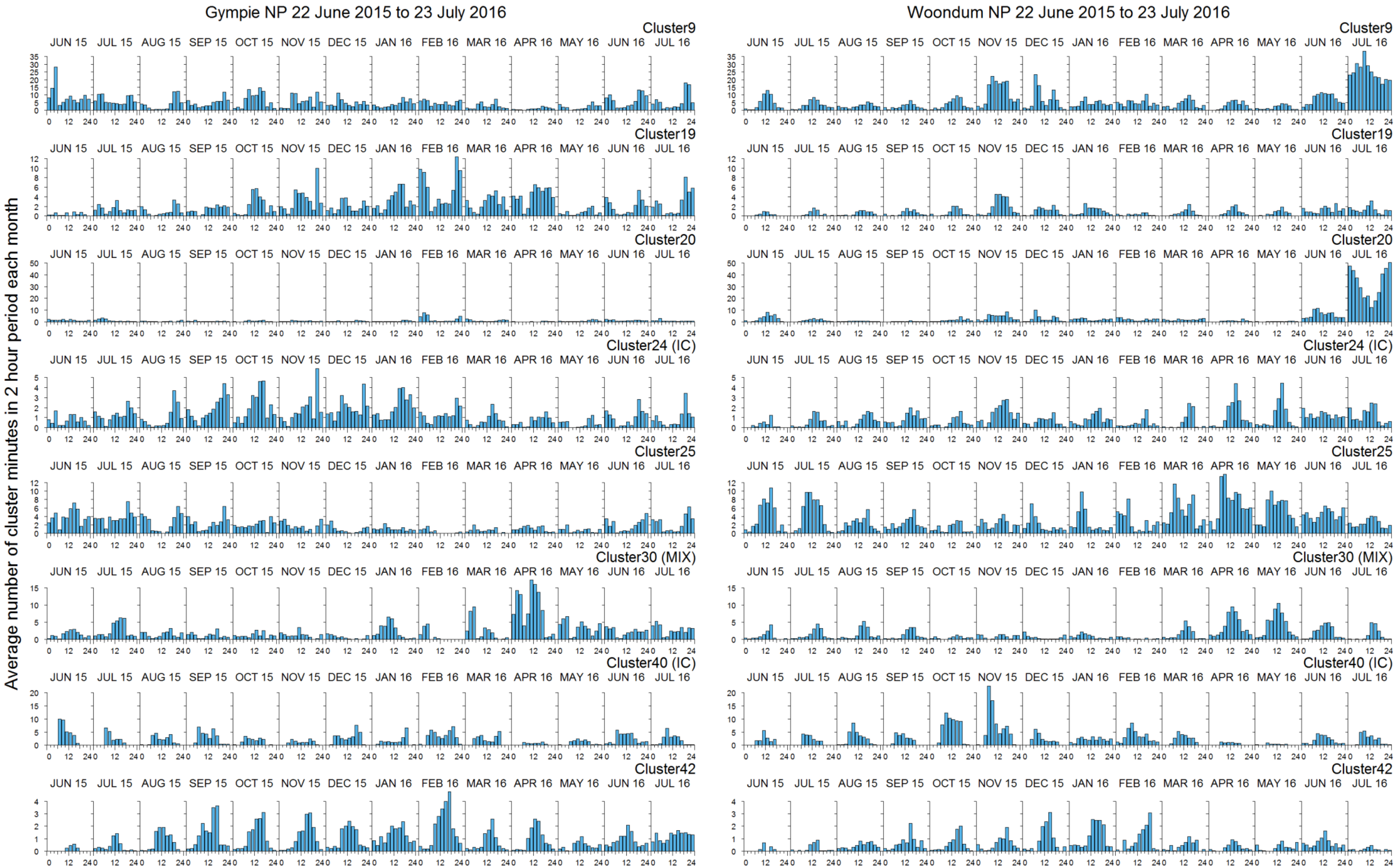

(MIX) - Cluster with more than one dominant acoustic class; (IC) - Inconsistent cluster

WIND CLUSTERS CONTINUED (#45, 46, 47, 51, 52, 56)

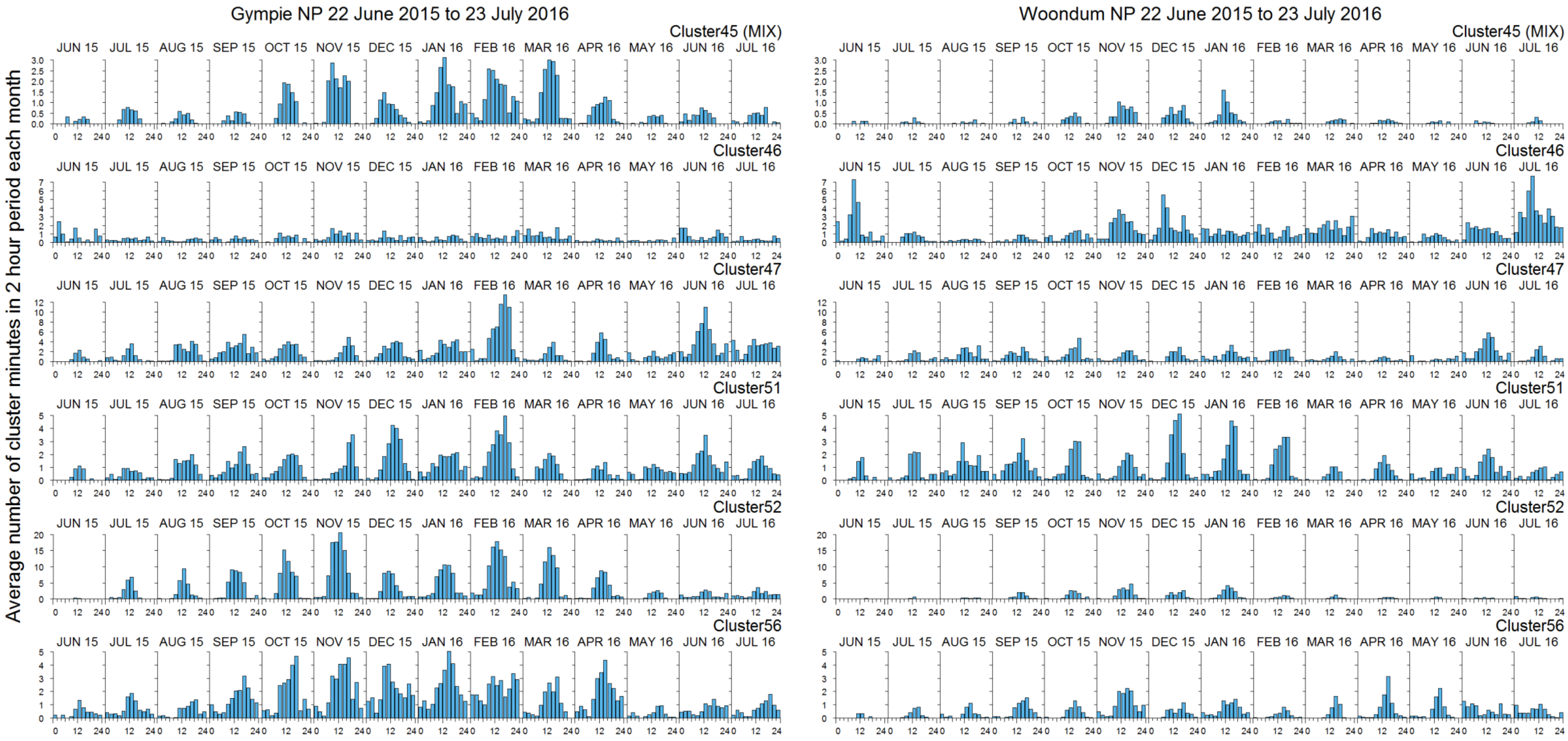

(MIX) - Cluster with more than one dominant acoustic class; (IC) - Inconsistent cluster

QUIET CLUSTERS (#5, 6, 13, 31, 35, 38, 41)

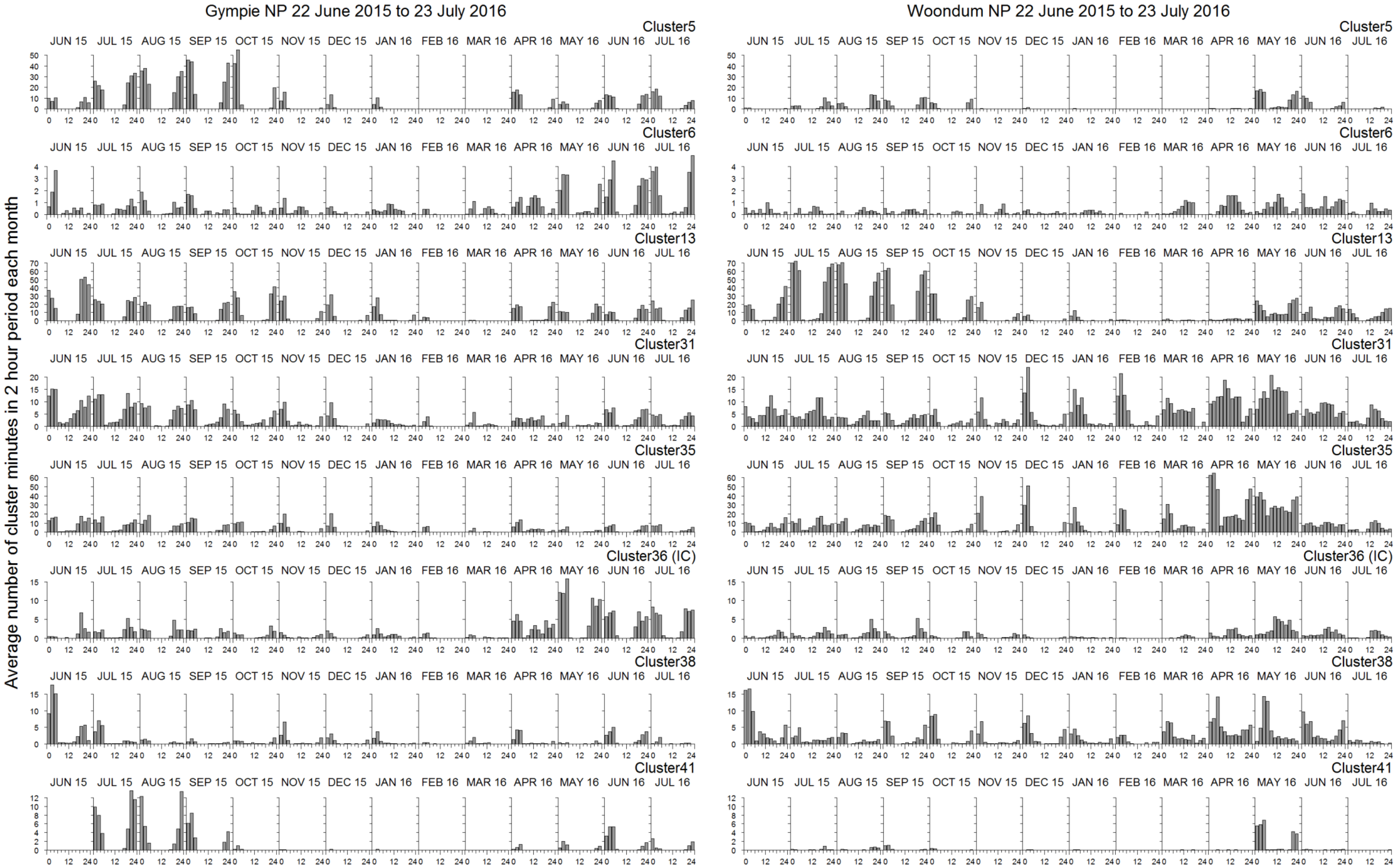

(MIX) - Cluster with more than one dominant acoustic class; (IC) - Inconsistent cluster

QUIET CLUSTERS CONTINUED (#50, 53, 55)

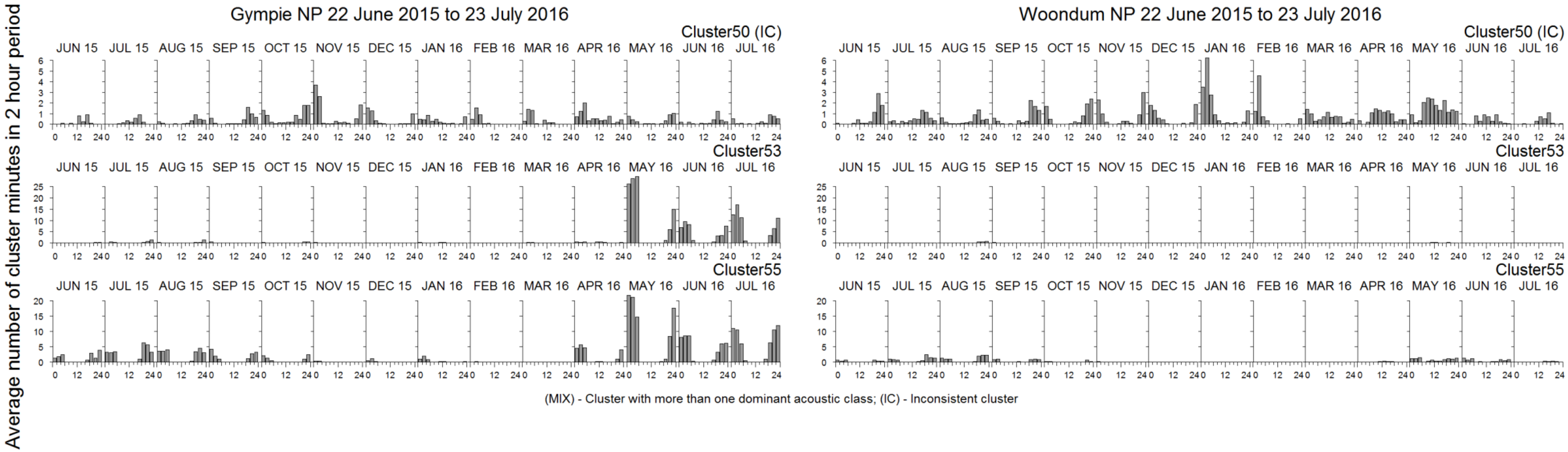

PLANE CLUSTERS (#23, 49)

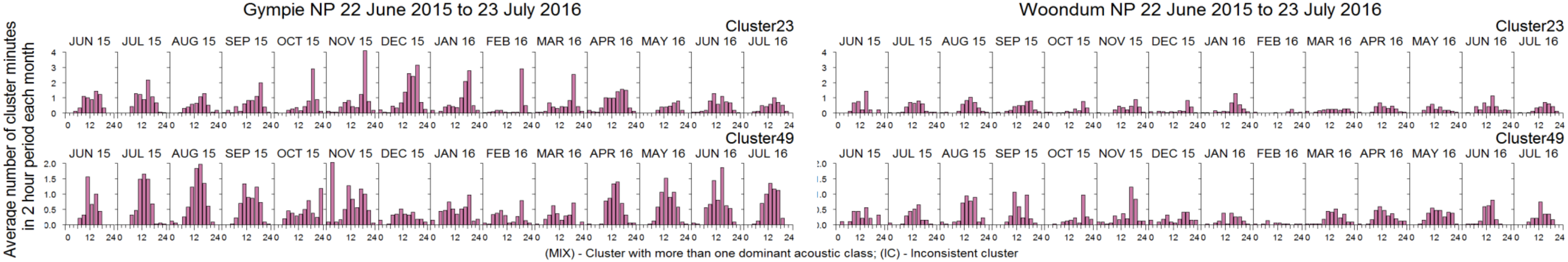

Section B: Polar Histogram for both sites

Two polar histogram plot displaying the proportion of the acoustic classes in each of the 398 days from the 22 June 2015 to the 23 July 2016 one from Gympie National Park and the other from Woondum National Park.

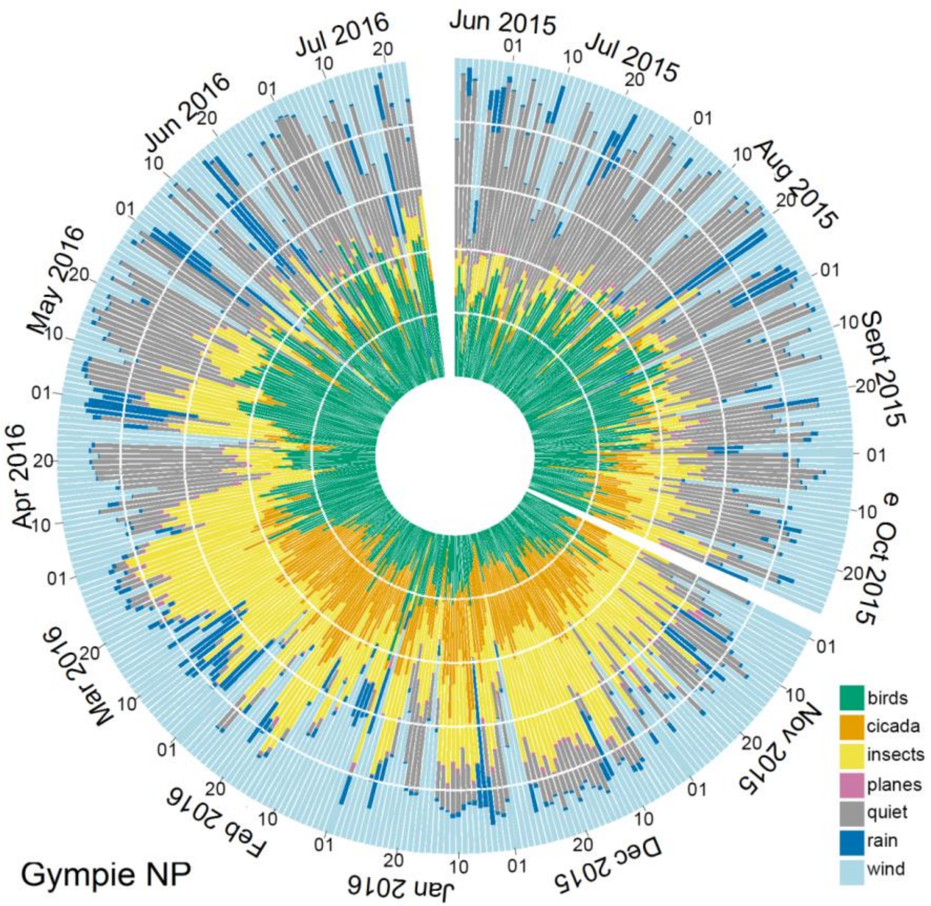

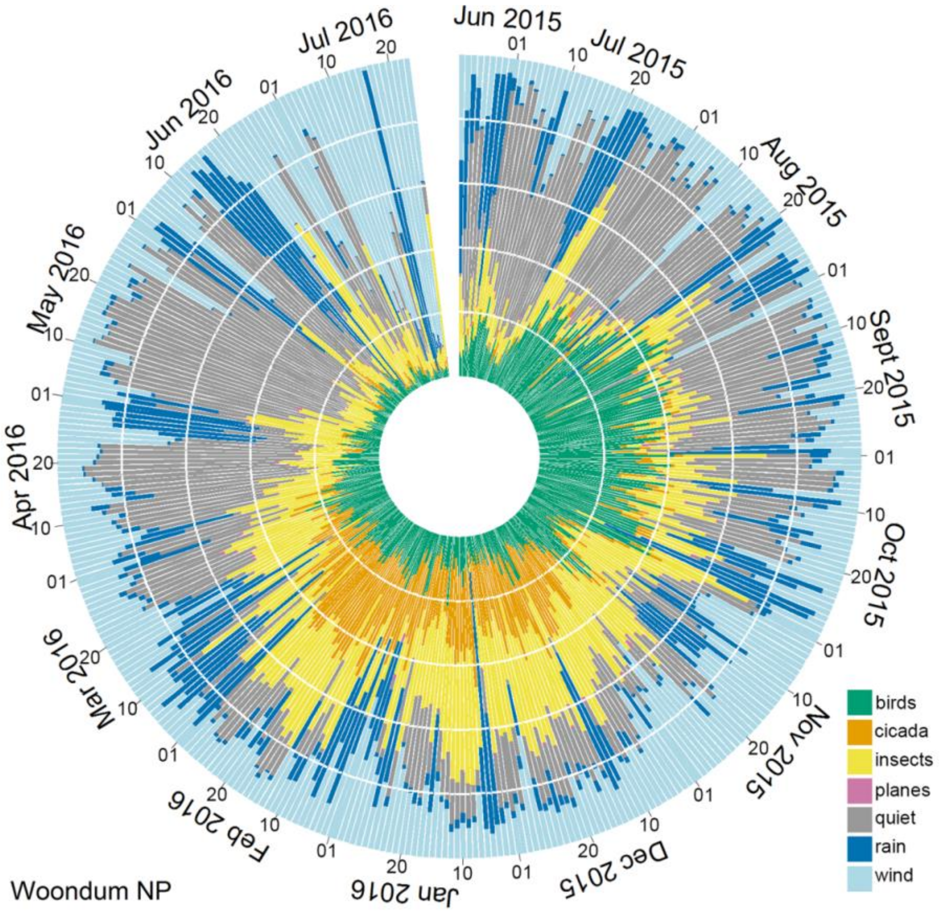

Section C: Radar Plots

The complete set of Radar Plots, one for each cluster displaying the mediods of each cluster

1. Forty-two clusters are dominated by a single acoustic class. The other clusters are mixtures of acoustic classes.
2. For a list of the contents of each cluster see page one of S2 Sample minutes.

Bird Clusters

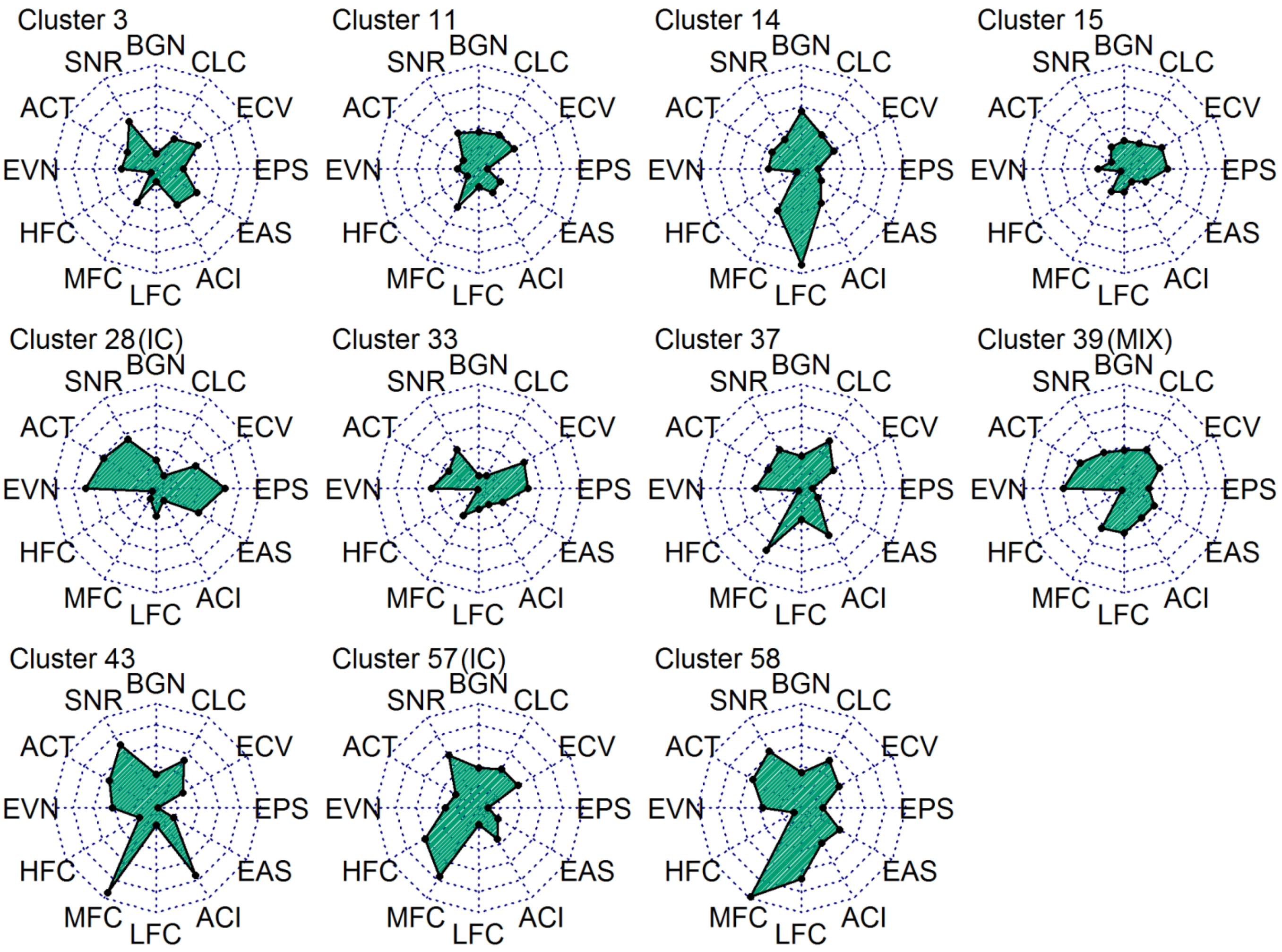

(MIX) - Cluster with more than one dominant acoustic class; (IC) - Inconsistent cluster

Plane Clusters

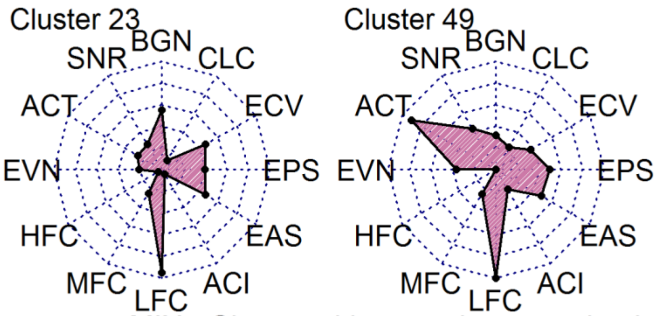

MIX - Cluster with more than one dominant acoustic class; IC - Inconsistent cluster

Insect Clusters

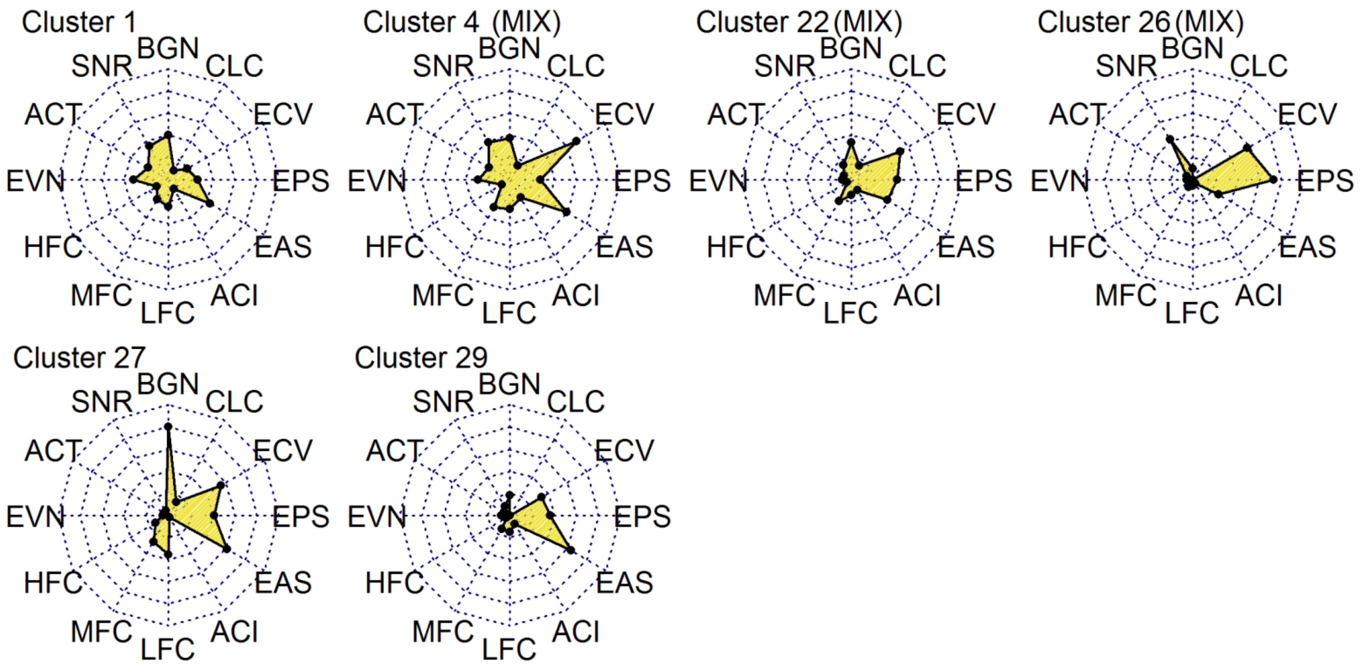

(MIX) - Cluster with more than one dominant acoustic class; (IC) - Inconsistent cluster

Cicada Clusters

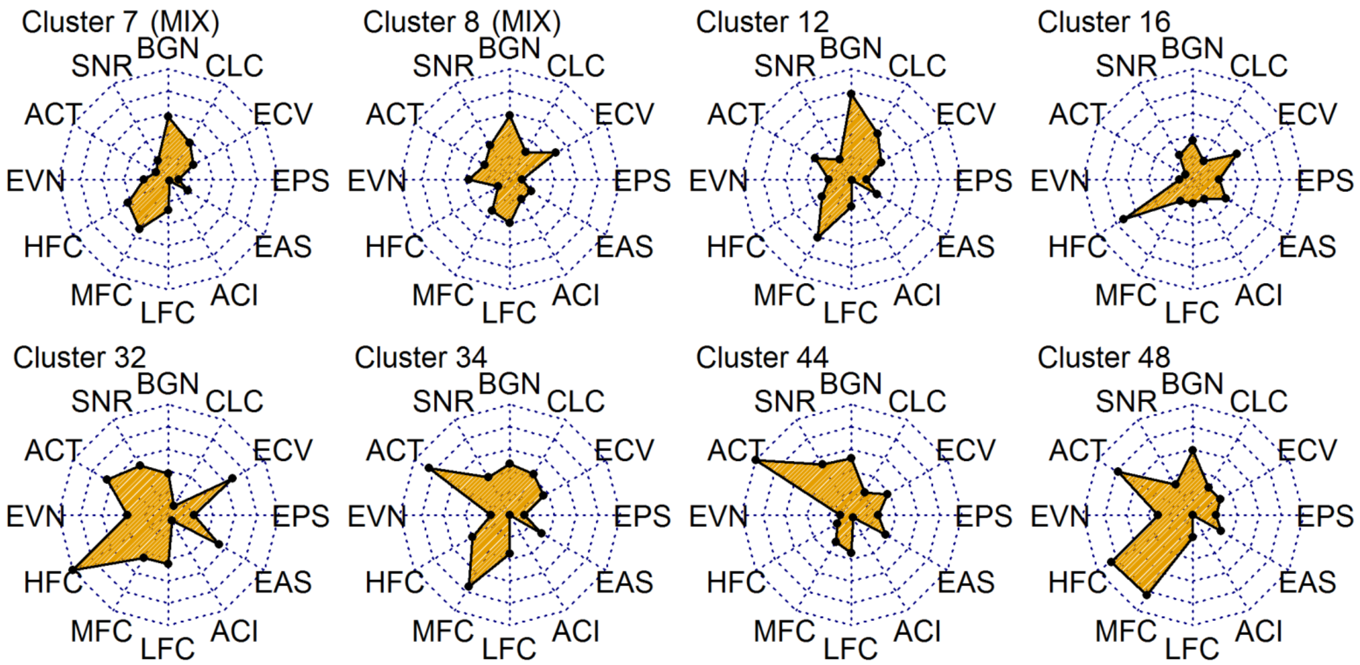

(MIX) - Cluster with more than one dominant acoustic class; (IC) - Inconsistent cluster

Rain Clusters

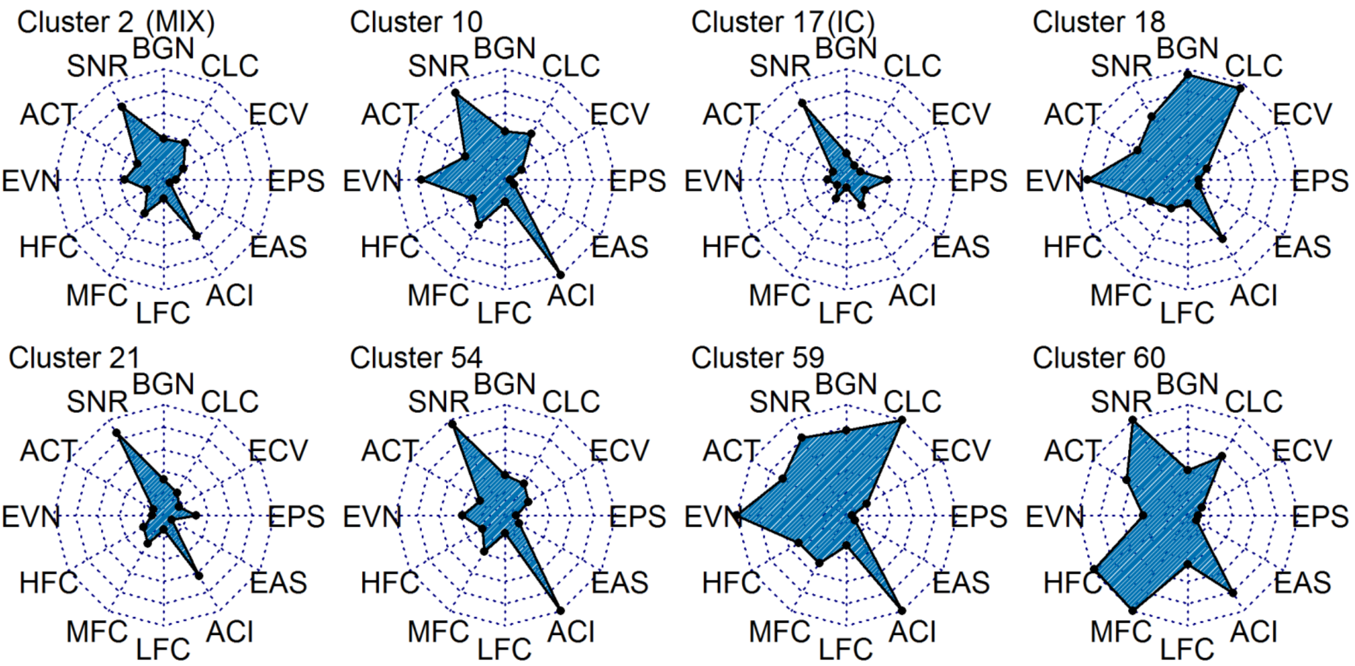

(MIX) - Cluster with more than one dominant acoustic class; (IC) - Inconsistent cluster

Quiet Clusters

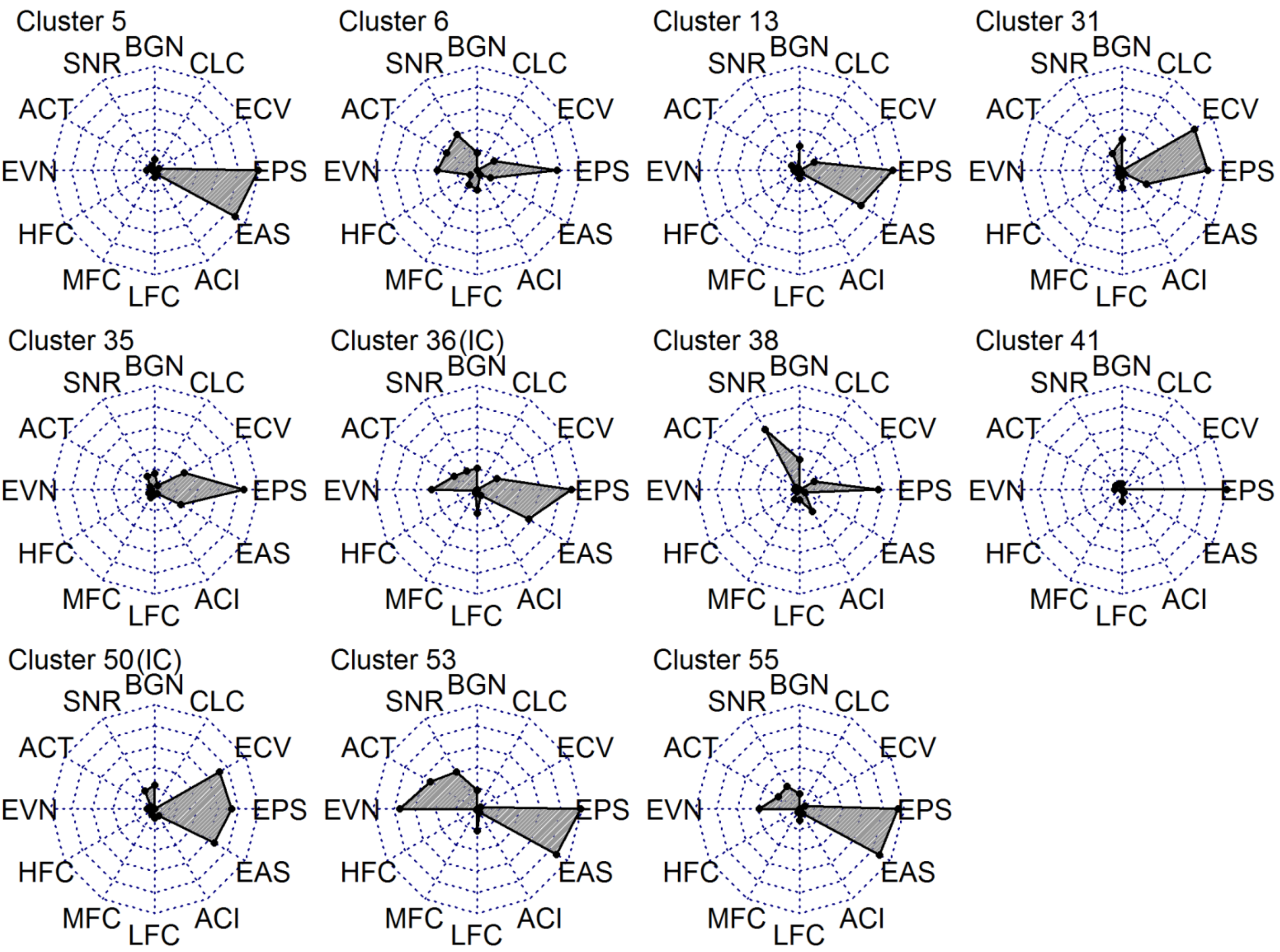

(MIX) - Cluster with more than one dominant acoustic class; (IC) - Inconsistent cluster

Wind Clusters

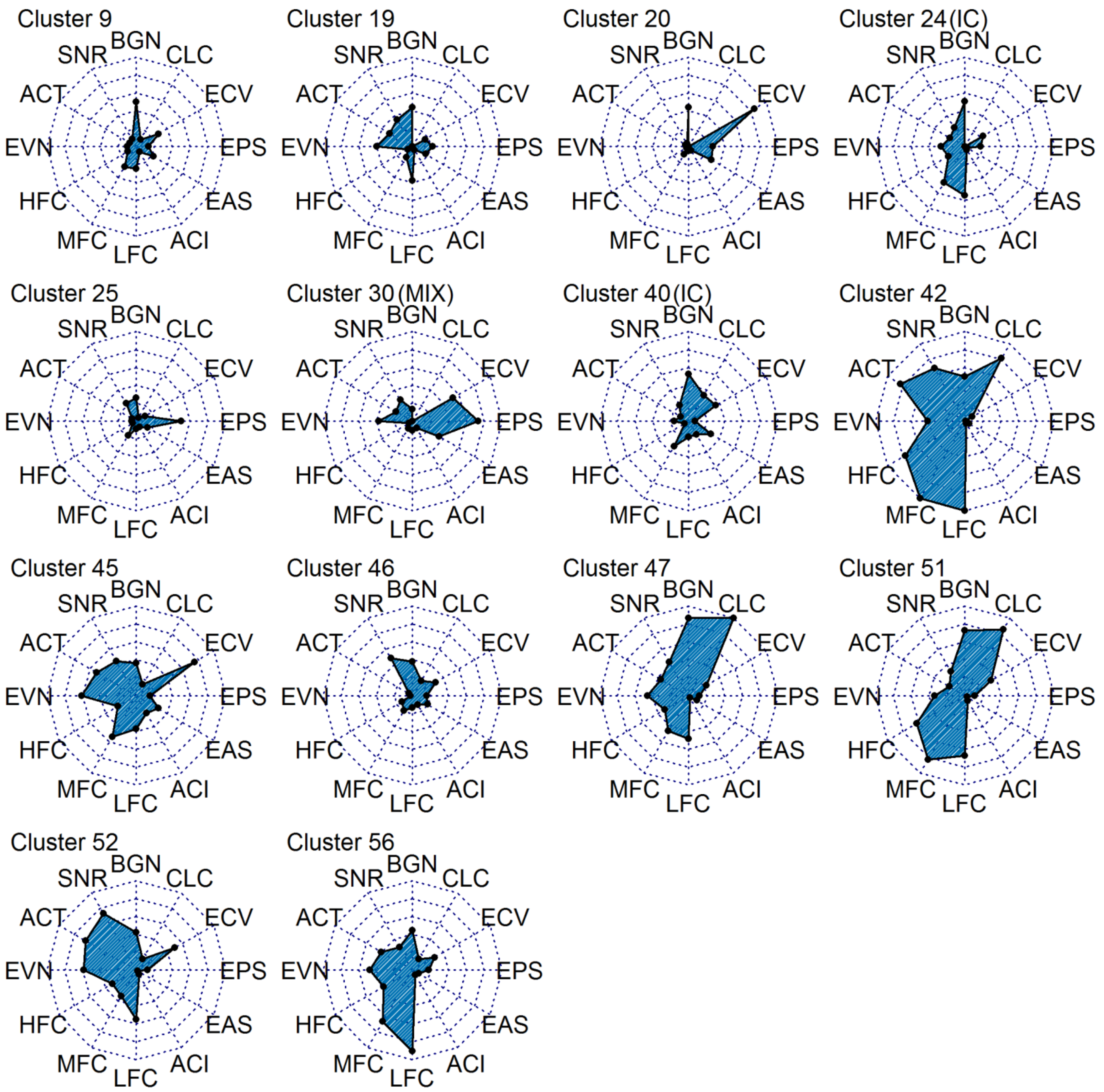

(MIX) - Cluster with more than one dominant acoustic class; (IC) - Inconsistent cluster

Section D: Boxplots

Contents: The boxplot statistics of each of the twelve summary acoustic indices for a selection of clusters in each acoustic class. The original indices were normalised between 1.5 and 98.5 percentiles and scaled [0,1].

1. BIOPHONY

BIRDS

These bird clusters have moderate values for the Acoustic Complexity Index (ACI) and low to moderate values for Cluster count (CLC). The mid-frequency cover (MFC) ranges from moderate to high as is the signal to noise ratio (SNR). The high-frequency cover (HFC) is generally low because most bird calls fall into the frequency band (482-4000 kHz) used to calculate the mid-frequency cover index. There is some elevation of low-frequency cover (LFC) in clusters 14 and 58 which is possibly due to planes.

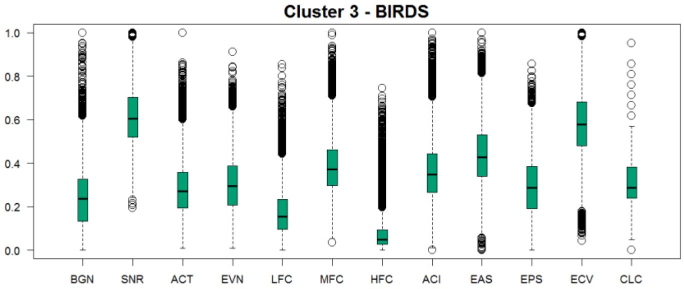

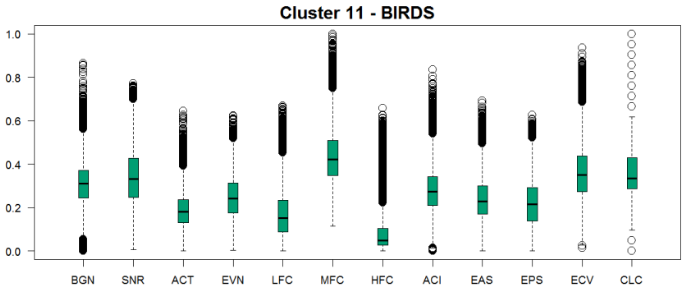

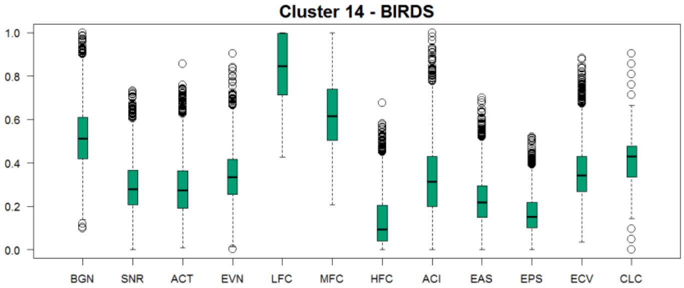

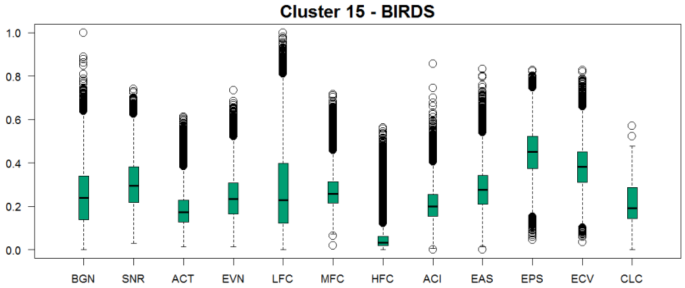

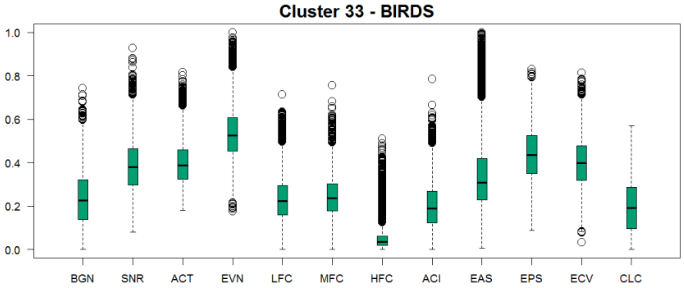

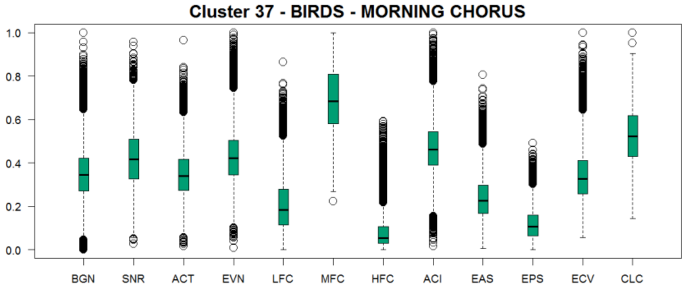

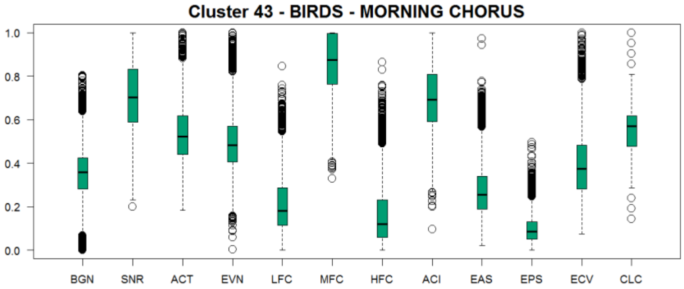

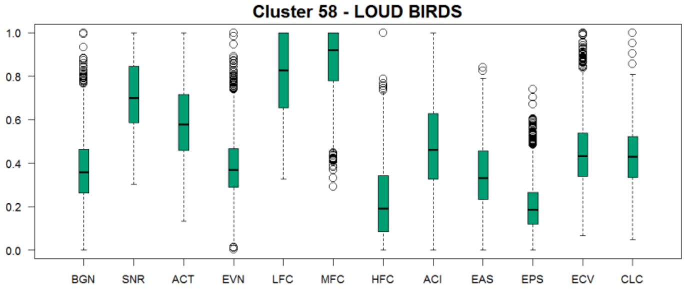

INSECTS

These insect clusters have low values for most summary indices.

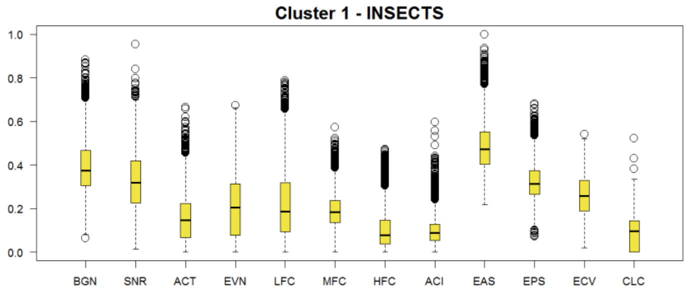

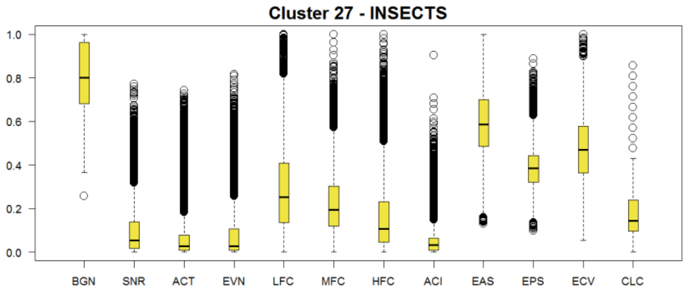

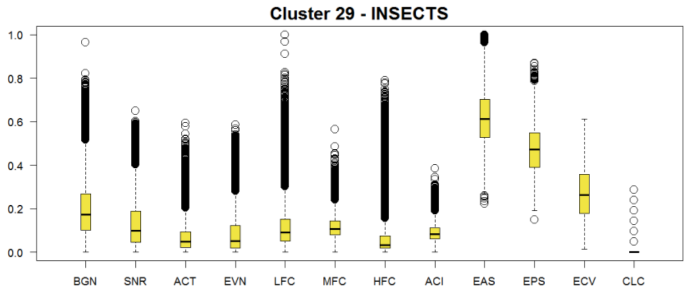

CICADAS

There is a large variation in index values amongst these cicada clusters, but consistently low ACI values across each. The broadband nature of the cicada calls in this area appear to dominate the mid and/or high frequencies increasing MFC and/or HFC.

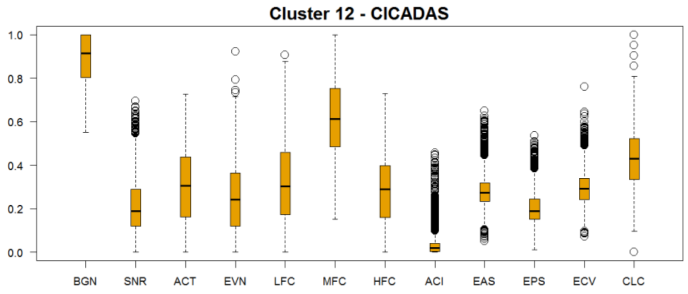

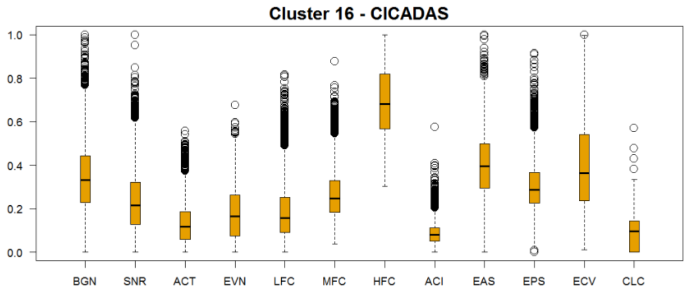

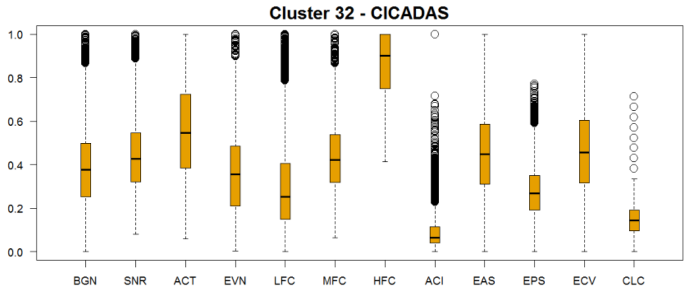

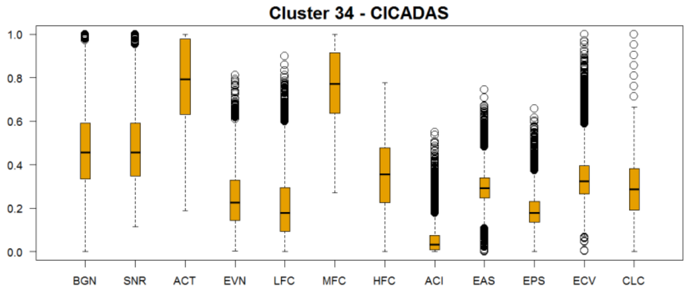

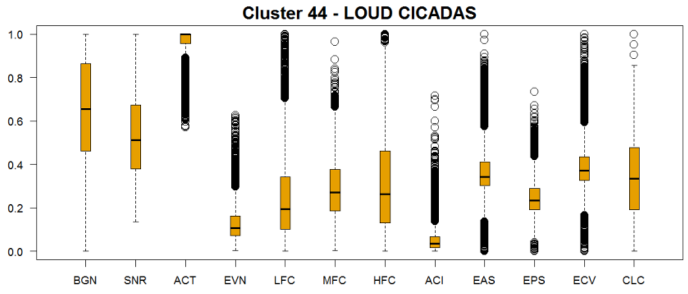

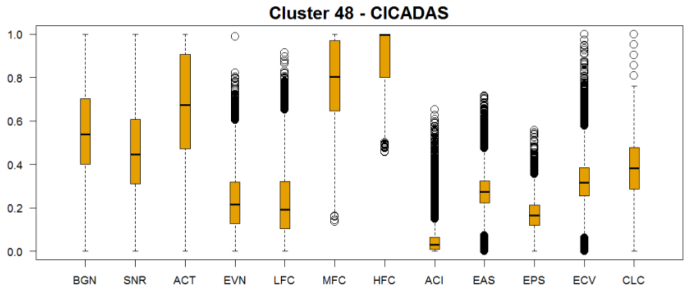

2. GEOPHONY

RAIN

The rain clusters have high to moderate SNR and ACI, due to the percussive nature of the raindrops. The three indices Background Noise (BGN), Events per Second (EVN) and Cluster Count (CLC) appear to increase with rain intensity. The three entropy indices are relatively low indicating a dispersal rather than a concentration of acoustic energy.

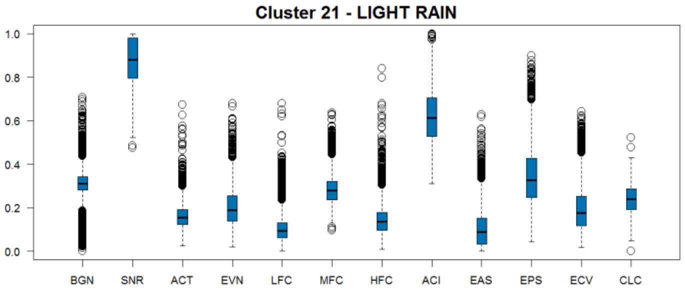

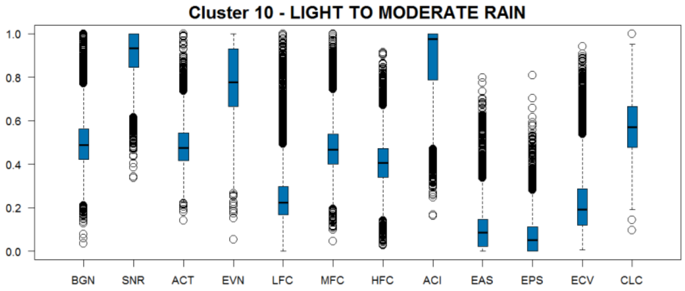

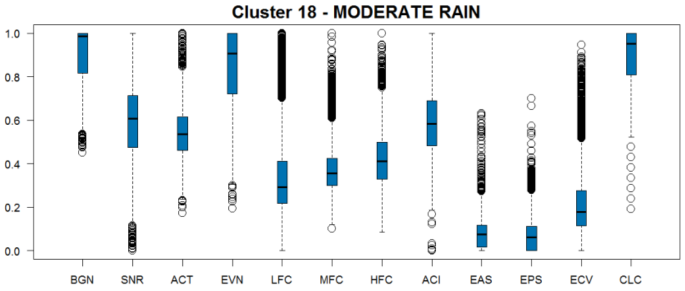

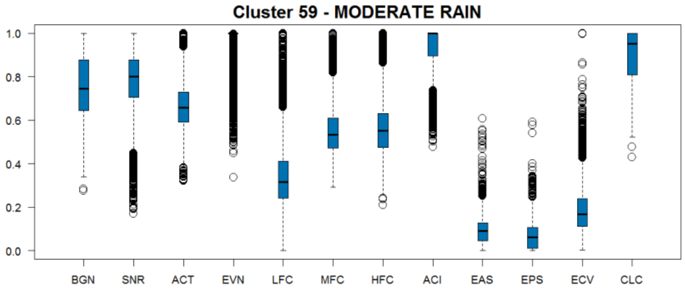

WIND

The strong wind (clusters 42, 47 and 51) have a very similar pattern of indices across the six indices BGN, ACI, EAS, EPS, ECV and CLC. The three entropy indices and ACI are relatively low. The three Frequency Cover indices (LFC, MFC and HFC) are high for the strong wind clusters (42 and 51) but not so for the very strong wind cluster, cluster 47

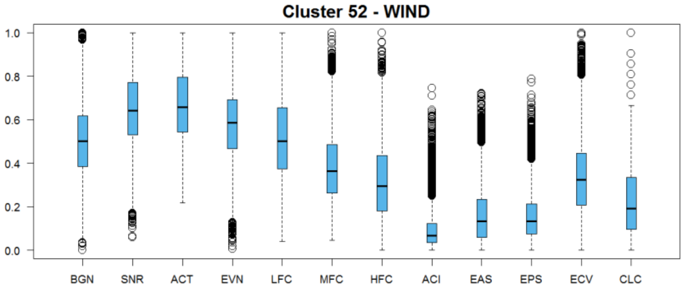

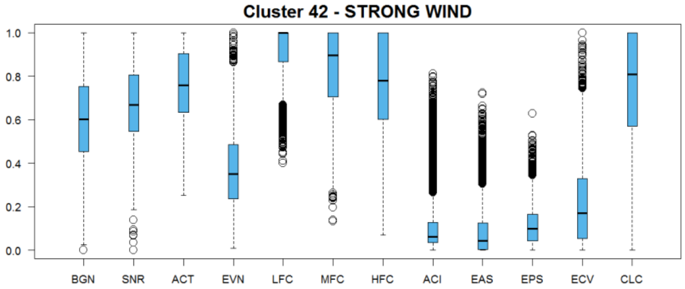

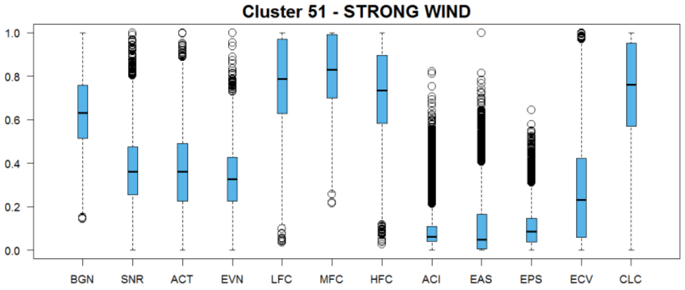

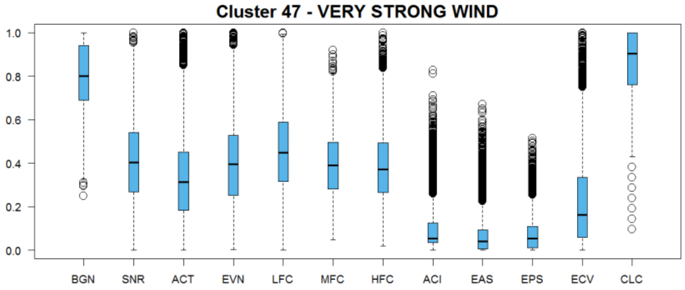

QUIET

Very low cluster count (CLC), high Entropy of Peaks Spectrum (EPS), low ACI and low mid (MFC) and high frequency cover (HFC).

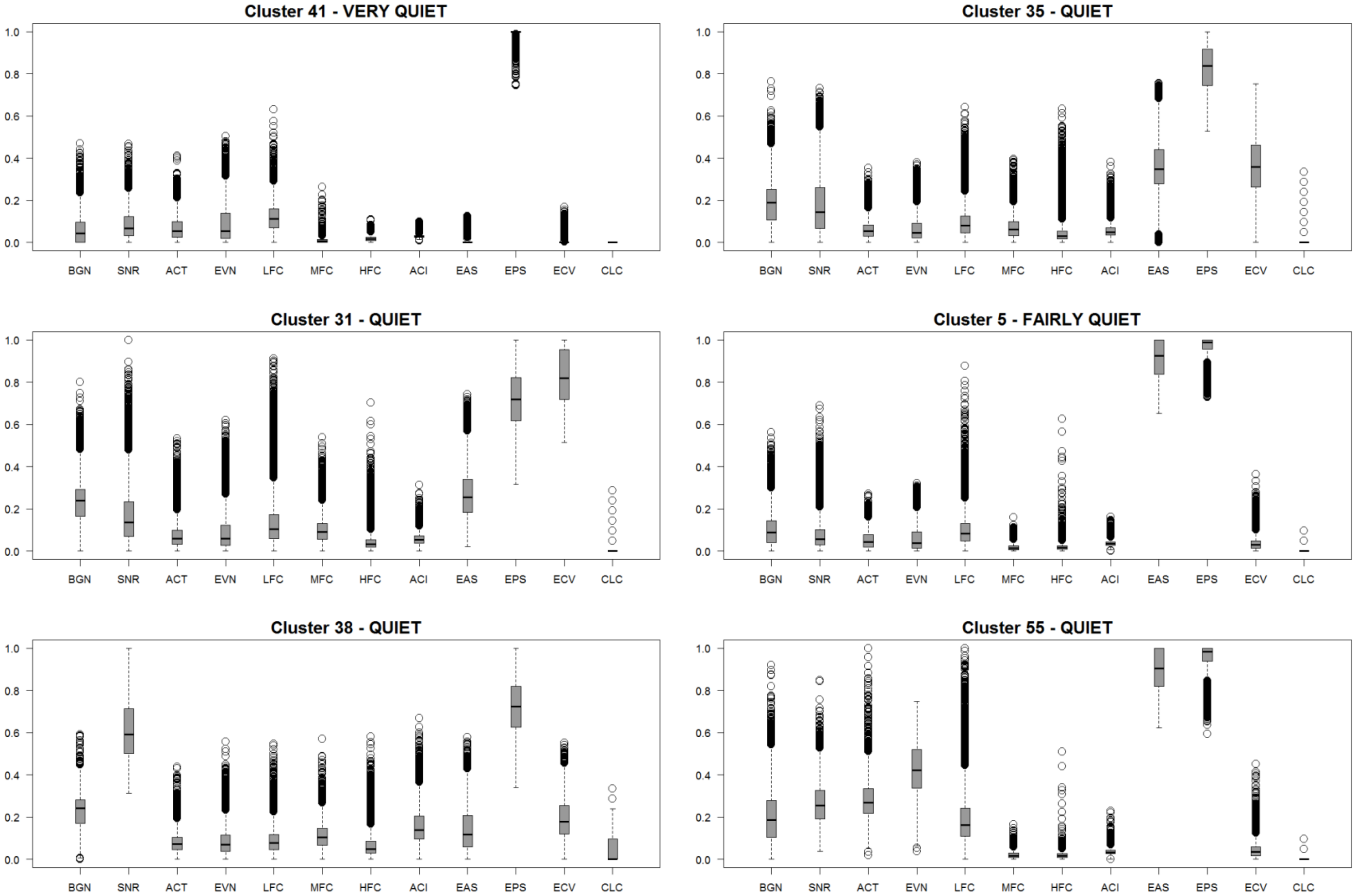

3. ANTHROPOPHONY

PLANES

The plane clusters have high low frequency cover (LFC) and a low cluster count (CLC). The three entropy indices (EAS, EPS and ECV) are moderate indicating a reasonable concentration of acoustic energy. The Acoustic Complexity Index is low because this index has a very low response to flat sounds such as planes [1]

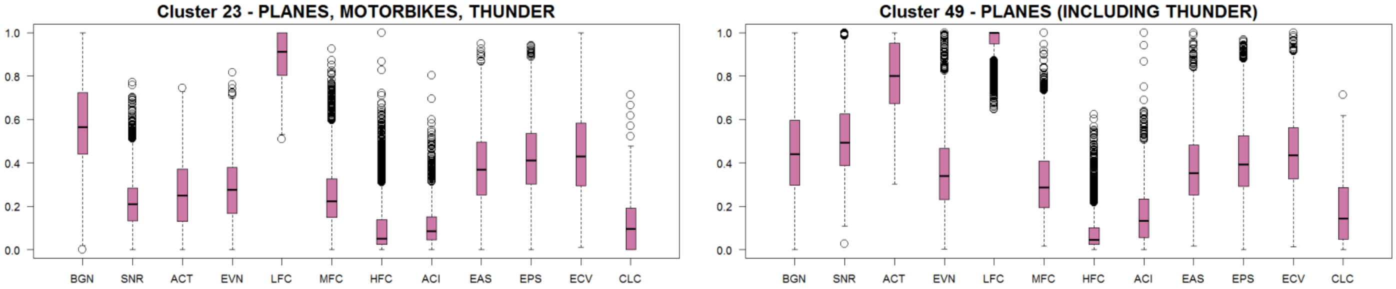

References

1. Pieretti N, Farina A, Morri D. A new methodology to infer the singing activity of an avian community: the acoustic complexity index (ACI). Ecological Indicators. 2011;11(3):868–73.
